# Synthesis of radiaannulene oligomers to model the elusive carbon allotrope 6,6,12-graphyne

**DOI:** 10.1038/s41467-019-11700-0

**Published:** 2019-08-16

**Authors:** Martin Drøhse Kilde, Adrian H. Murray, Cecilie Lindholm Andersen, Freja Eilsø Storm, Katrin Schmidt, Anders Kadziola, Kurt V. Mikkelsen, Frank Hampel, Ole Hammerich, Rik R. Tykwinski, Mogens Brøndsted Nielsen

**Affiliations:** 1 Department of Chemistry, University of Copenhagen, Universitetsparken 5, DK-2100 Copenhagen Ø, Denmark; 2 Department of Chemistry, University of Alberta, Edmonton, AB Canada T6G 2G2; 3 Department für Chemie und Pharmazie, Friedrich-Alexander-Universität Erlangen-Nürnberg (FAU), Nikolaus-Fiebiger-Straße 10, 91058 Erlangen, Germany

**Keywords:** Materials chemistry, Organic chemistry, Nanoscale materials

## Abstract

Graphyne allotropes of carbon are fascinating materials, and their electronic properties are predicted to rival those of the “wonder material” graphene. One allotrope of graphyne, having rectangular symmetry rather than hexagonal, stands out as particularly attractive, namely 6,6,12-graphyne. It is currently an insurmountable challenge, however, to design and execute a synthesis of this material. Herein, we present synthesis and electronic properties of molecules that serve as model compounds. These oligomers, so-called radiaannulenes, are prepared by iterative acetylenic coupling reactions. Systematic optical and redox studies indicate the effective conjugation length of the radiaannulene oligomers is nearly met by the length of the trimer. The HOMO-LUMO gap suggested by the series of oligomers is still, however, higher than that expected for 6,6,12-graphyne from theory, which predicts two nonequivalent distorted Dirac cones (no band gap). Thus, the radiaannulene oligomers present a suitable length in one dimension of a sheet, but should be expanded in the second dimension to provide a unique representation of 6,6,12-graphyne.

## Abstract (web summary)

6,6,12-graphyne is an intriguing synthetic allotrope of carbon that is predicted to have unique electronic properties but has not been successfully synthesized. Here, the authors prepare a series of radiaannulene oligomers that can be regarded as large segments of this elusive graphyne allotrope.

## Introduction

The promise of synthetic carbon allotropes (i.e., allotropes or derivatives not naturally occurring) lies in their lightweight, environmentally friendly, and electronically diverse properties^1,2^. As an example, graphene is a two-dimensional monolayer of graphite that consists of hexagon lattices of sp^2^-hybridized carbon atoms. Graphene and its derivatives have been termed “wonder materials” as a result of unique optical and electrical properties, many of which derived from the fact that it is a conductor, i.e., it lacks a band gap^3^. On the other hand, interest in graphene nanoribbons (GNRs) has recently exploded, in order to bridge the properties between 1D allotropes and 2D graphene. GNRs share many of the desirable properties of bulk 2D graphene, while quantum confinement in one dimension offers a defined band gap necessary for semi-conductor applications and devices^4–6^.

**Fig. 1:**
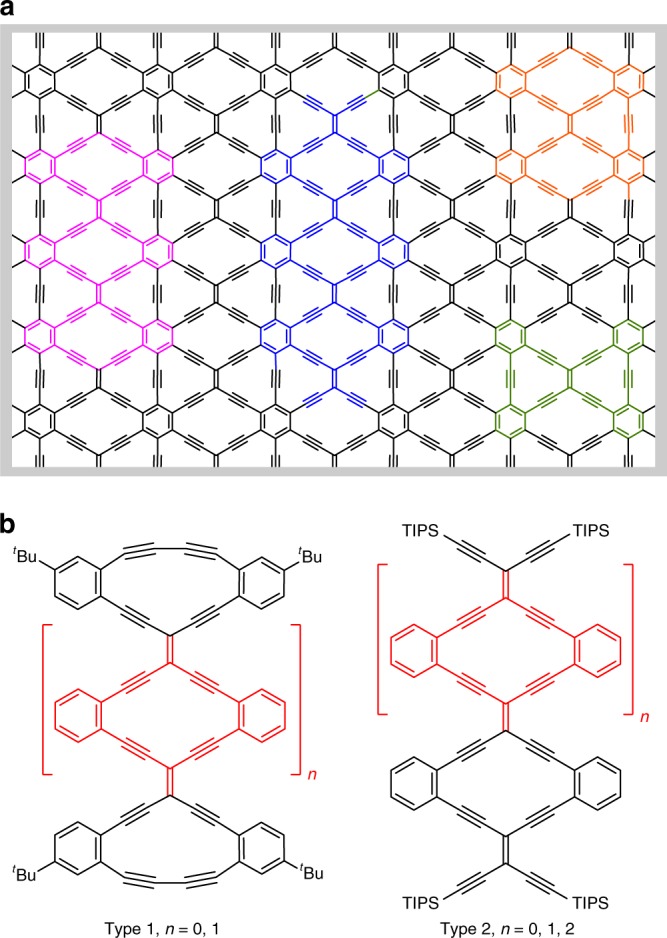
Structure of 6,6,12-graphyne. **a** Schematic representation of 6,6,12-graphyne with fragments highlighted in colors. **b** Radiaannulene oligomer model systems of Type 1 and Type 2. ^*t*^Bu = *tert*-butyl, TIPS = triisopropylsilyl

Graphynes are a class of synthetic carbon allotropes that are obtained by interspersing sp-hybridized carbon atoms into the sp^2^-carbon framework of graphene. Recent calculations have predicted that graphynes could be superior to graphene for electronic and optoelectronic applications^7–16^. Four graphyne derivatives, in particular, have attracted attention, namely α-, β-, γ-, and 6,6,12-graphyne, which differ in structure as well as the percentage of sp-hybridized carbon content versus sp^2^-carbon in their conjugated structure. For example, α-graphyne contains 75% sp-carbons, which is reduced to 55.56% for 6,6,12-graphyne. Theoretically, graphynes are predicted to exhibit Dirac cones, which means that the valence and conduction bands are shaped as upper and lower conical surfaces that meet at the so-called Dirac point (corresponding to a zero band gap)^9^. Experimentally, there are very few synthetic attempts reported for either α- or β-graphyne, although Li et al.^17^ recently found that a small fragment of α-graphyne, a so-called carbo-benzene^18^, exhibits a very high single-molecule conductance, and Maraval, Chauvin et al.^19^ have recently reported an elegant synthesis of carbo-naphthalene, a polycyclic carbo-benzenoid fragment of α-graphyne. Synthetically, most efforts have focused on γ-graphyne fragments comprised of tribenzocyclyne units, pioneered by Haley^20,21^ and Tobe^22,23^. From time-resolved microwave conductivity measurements, it was concluded that large γ-graphyne fragments could be applied as organic semiconductors^23^. Of the various graphynes that have been commonly considered to date, however, one stands out as unique, namely 6,6,12-graphyne (Fig. 1a). This material does not share the hexagonal symmetry common to graphene and other graphynes. Rather, 6,6,12-graphyne features rectangular symmetry, and, according to theoretical studies, it should feature two self-doped nonequivalent Dirac cones, i.e., electrons are present as charge carriers in one Dirac cone, while holes are present in the other^8^. For these reasons, direction-dependent electronic conduction is expected for this allotrope of graphyne. In addition, the carrier mobility of 6,6,12-graphyne is predicted to be even larger than in graphene^10^. Finally, segments of 6,6,12-graphyne, graphyne nanoribbons (GyNRs), are predicted to exhibit unique transport properties for electronic devices^24^.

Despite the remarkable properties predicted by theoretical calculations, the synthesis of either 6,6,12-graphyne or significant segments of its structure (exhibiting quantum confinement) remains elusive. For this reason, we initiated synthetic attempts toward segments of this allotrope. Examining the structure of 6,6,12-graphyne, one of the core repeat units can be described as a hybrid between a dehydrobenzannulene and an expanded radialene, a structure known generally as a radiaannulene^25^. Based on this motif, we identify two series of oligomers to help explore the potential properties of 6,6,12-graphyne (Fig. 1b). Type 1 radiaanulene oligomers feature edges that are closed by a butadiyne segment (i.e., the exocyclic C = C is removed at each end of the oligomer, representing a potentially stable edge structure), and Type 2 radiaanulene oligomers contain edges that are open as functionalized tetraethynylethene (TEE) units (representing the basic repeat units). Herein, we present the synthesis and properties of oligomers of Type 1 and Type 2. In addition, macrocyclic subunits (a bis-dehydrobenzannulene—highlighted in green in Fig. 1a—and radiaannulenes) are identified and synthesized in order to support comparisons as a function of molecular size.

## Results

### Synthesis

**Fig. 2:**
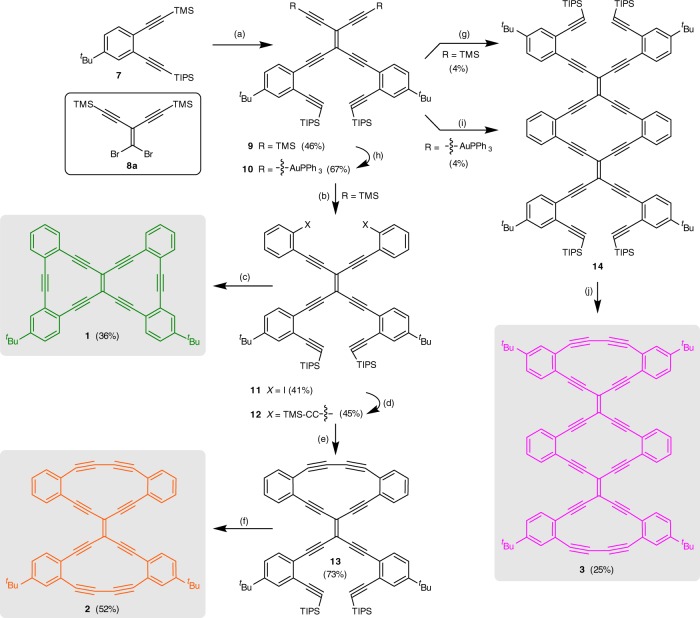
Stepwise synthesis of 6,6,12-graphyne fragment **1** and Type 1 radiaannulenes **2** and **3**. Reagents and conditions: **a** (i) K_2_CO_3_, THF/MeOH, (ii) **8a**, Cat. I, Et_3_N/THF, ))), 40 °C. **b** 1,2-Diiodobenzene, K_2_CO_3_, Cat. I, THF/Et_3_N/MeOH, ))), 40 °C. **c** (i) TBAF, THF/H_2_O, (ii) Cat. I, THF/Et_3_N. **d** TMS-acetylene, Cat. I, THF/Et_3_N, ))), 35 °C. **e** (i) K_2_CO_3_, THF/MeOH, (ii) Cat II, O_2_, 4 Å MS, CH_2_Cl_2_. **f** (i) TBAF, THF/H_2_O, (ii) Cat II, O_2_, 4 Å MS, CH_2_Cl_2_. **g** (i) K_2_CO_3_, THF/MeOH, (ii) **11**, Cat. I, THF/Et_3_N/MeOH, MW, 75 °C. **h** ClAuPPh_3_, NaOMe, THF/MeOH. (i) **11**, Cat. I, THF/Et_3_N/MeOH, MW, 75 °C. **j** (i) TBAF, THF/H_2_O, (ii) Cat II, O_2_, 4 Å MS, CH_2_Cl_2_. Cat. I = Pd(PPh_3_)_2_Cl_2_/CuI, Cat. II = CuCl, TMEDA = *N*,*N*,*N’*,*N’*-tetramethylethylenediamine, ^*t*^Bu = *tert*-butyl, Ph = phenyl, TMS = trimethylsilyl, TIPS = triisopropylsilyl, TBAF = tetrabutylammonium fluoride, MS = molecular sieves, MW = microwave, ))) = sonication (ultrasound)

**Fig. 3:**
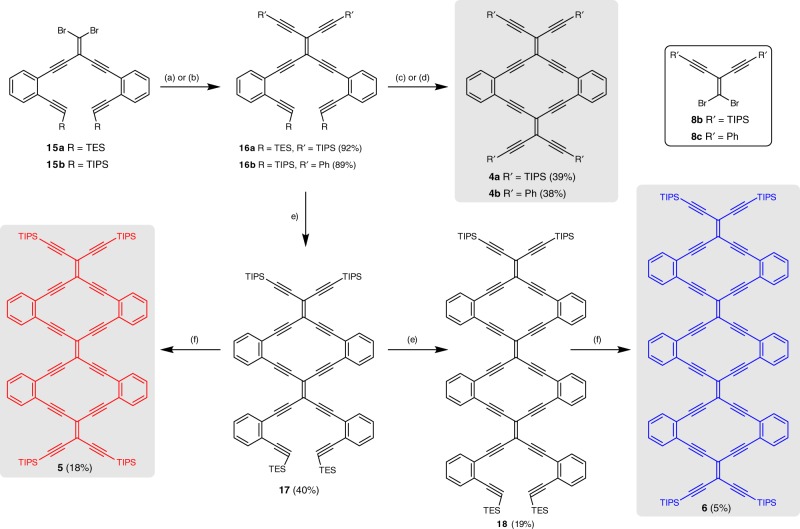
Stepwise synthesis of 6,6,12-graphyne fragments **4a, b** and Type 2 radiaannulenes **5** and **6**. Reagents and conditions: **a** R = TES: TIPS-acetylene, Cat. III, Et_3_N, 50 °C. **b** R = TIPS: phenylacetylene, Cat. III, THF/(*i*Pr)_2_NH, 60 °C. **c** R = TES, R’ = TIPS: (i) *t*BuOK, THF/MeOH, 40 °C, (ii) **8b**, Cat. III, THF/(*i*Pr)_2_NH, 60 °C. **d** R = TIPS, R’ = Ph: (i) TBAF, THF, 0 °C, (ii) **8c**, Cat. III, PhMe/(*i*Pr)_2_NH, 50 °C. **e** R = TES, R’ = TIPS: (i) KOH, THF/MeOH, (ii) **15a**, Cat. III, Et_3_N, 50 °C. **f** (i) KOH, THF/MeOH, (ii) **8b**, Cat. III, Et_3_N, 50 °C. Cat. III = Pd(PPh_3_)_4_/CuI, TBAF = tetrabutylammonium fluoride, Ph = phenyl, TES = triethylsilyl, TIPS = triisopropylsilyl

The synthesis of bis-dehydroannulene **1** and radiaannulenes of Type 1 (compounds **2** and **3**) is shown in Fig. 2, while the synthesis of radiaannulenes of Type 2 (compounds **4a–b**, **5**–**6**) is presented in Fig. 3. In general, these syntheses relied on an iterative series of Pd-catalyzed Sonogashira coupling reactions between terminal alkynes and a suitable vinyl- or arylhalide (Br, I) electrophile. Furthermore, our strategy exploits the ability to selectively remove trimethylsilyl (TMS) and triethylsilyl (TES) alkyne-protecting groups before the triisopropylsilyl (TIPS) group.

The first target, bis-dehydroannulene **1**, was synthesized starting from the silyl-protected diethynylbenzene derivative **7**, which was selectively desilylated and treated with the vinyl dibromide **8a**^26,27^ under Sonogashira coupling conditions, to provide product **9**. This compound (**9**) was then elaborated to **11** via desilylation followed by a Sonogashira coupling with 1,2-diiodobenzene. With **11** in hand, a subsequent sequence of desilylation followed by intramolecular Sonogashira coupling gave annulene **1**. Compound **11** represented a common precursor for the synthesis of GyNR **2**. Cross-coupling of **11** with TMS-acetylene gave **12**, and desilylation followed by intramolecular Hay homocoupling gave **13**. A second sequence of desilylation and homocoupling then gave the dimer **2**. Noteworthy, the oxidative Hay couplings to give the butadiyne edges of the target molecules **2** and **13** were accomplished in the presence of molecular sieves (4 Å) to remove water as previously described to be beneficial for such couplings^28^.

Compound **14**, the precursor for the trimer **3**, was first prepared from **9** via desilylation and subsequent intermolecular Sonogashira coupling with **11**, but yields were low (Fig. 2; conditions g). Alternatively, triphenylphosphine Au(I) end-capped intermediate **10** could be formed from **9** and coupled with **11** under Sonogashira-like conditions. In spite of previous reports that suggested Au(I) oligoynyl complexes can be excellent substrates in cases in which the unprotected terminal alkynes exhibit limited stability^29^, an improved yield of **14** was not realized by this protocol (Fig. 2; conditions h). In a final step, trimer **3** was completed using the standard desilylation and homocoupling sequence, representing a significant segment of 6,6,12-graphyne (the magenta-colored segment in Fig. 1).

Several approaches were explored for assembly of Type 2 radiaanulene oligomers. Initially, a one-pot approach was tested, which had previously been used effectively for the synthesis of radiaannulenes^30^. While successful for the assembly of, for example, **4a** and **5**, these tactics consistently gave low yields (see Supplementary Methods, p. 24–28, for details) and were eventually abandoned in favor of a stepwise approach. The advantage of the revised method derived from a common building block to be used in the synthesis of each oligomer, namely enediyne **15a**. Sonogashira coupling of **15a** with TIPS-acetylene gave **16a**, which could be converted to radiaannulene monomer **4a** via desilylation and Sonogashira coupling with dibromoolefin **8b**^31^. An analogous sequence of reactions, starting from **15b** and phenylacetylene, gave **16b**, which underwent ring closure using dibromoolefin **8c** to give radiaannulene **4b**. Alternatively, starting with **16a**, a sequence of desilylation and cross-coupling with **15a** gave the expanded system **17**, which was then readily converted into dimer **5** via desilylation and Sonogashira coupling with **8b**. Finally, the entire expansion process was repeated, starting with **17**, to give **18** and then **6**, completing the blue-colored segment of 6,6,12-graphyne highlighted in Fig. 1. The structural assignment of **6** rests on high-resolution mass spectrometry and UV–vis spectroscopy from a small amount (μgs) of sample purified by preparative thin-layer chromatography, and the limited amount of sample precluded further characterization. The majority of the product isolated from the reaction mixture, calculated as 5% yield (3.5 mg) based on the assumption of a pure compound, decomposed upon exposure to CDCl_3_ during sample preparation for NMR analyses (see Supplementary Methods, p. 20, for full synthetic details).

The isolated Type 1 and Type 2 molecules showed reasonable solubility in common organic solvents as a result of the *t*-butyl and triisopropylsilyl groups incorporated at the periphery of the molecules. All compounds exhibited good stability, except for the trimer **6** that appeared to rapidly (and surprisingly) decompose when dissolved in CDCl_3_, which ultimately limited characterization. When macroscopic quantities were available for analyses, differential scanning calorimetry confirmed excellent thermal stability with decomposition temperatures often above 250 °C.

### X-ray crystallography

An interesting structural aspect of the Type 1 radiaannulenes is the ring strain introduced through the closing of the butadiyne segment. Unfortunately, the presence of *tert*-butyl substituents, introduced to maintain solubility, also appear to thwart the formation of X-ray quality single crystals, with only one exception. An intermediate radiaannulene was successfully analyzed by X-ray crystallography (see Supplementary Fig. 77 for details), showing that the interior angles of the butadiyne segment are only moderately strained with acetylenic bond angles from 177 to 167°.

**Fig. 4:**
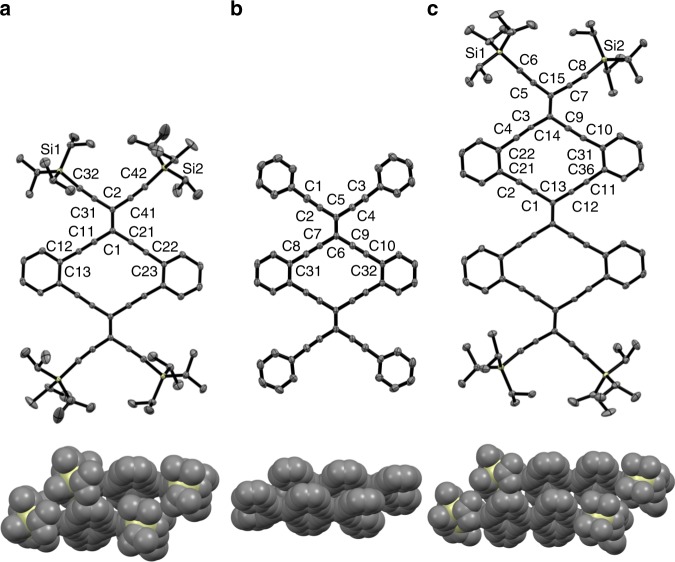
Molecular structures obtained by X-ray crystallography. ORTEP structure (top) and solid-state packing (bottom) of (**a**) radiaannulene **4a**, (**b**) radiaannulene **4b**, and (**c**) radiaannulene dimer **5**; selected bond lengths [Å] and angles [°] **4a**: C(1)–C(2) 1.378(2), C(11)–C(12) 1.201(3), C(21)–C(22) 1.199(3), C(31)–C(32) 1.206(3), C(41)–C(42) 1.202(3); C(31)–C(2)–C(41) 116.02(15), C(11)–C(1)–C(21) 117.45(15), C(1)–C(11)–C(12) 174.6(2), C(11)–C(12)–C(13) 177.8(2), C(1)–C(21)–C(22) 178.6(2), C(21)–C(22)–C(23) 176.56(19); **4b**: C(5)–C(6) 1.374(5), C(7)–C(8) 1.188(4), C(9)–C(10) 1.197(4), C(1)–C(2) 1.182(4), C(3)–C(4) 1.181(4); C(3)–C(2)–C(1) 117.4(3), C(7)–C(6)–C(9) 118.2(3), C(6)–C(7)–C(8) 178.9(4), C(7)–C(8)–C(31) 177.8(3), C(6)–C(9)–C(10) 179.3(4), C(9)–C(10)–C(32) 179.4(4); **5**: C(13)–C(13’) 1.384(3), C(1)–C(2) 1.198(3), C(11)–C(12) 1.202(3), C(14)–C(15) 1.379(3), C(3)–C(4) 1.172(3), C(9)–C(10) 1.193(3), C(5)–C(6) 1.210(3), C(7)–C(8) 1.211(3); C(5)–C(15)–C(7) 115.74(16), C(13)–C(1)–C(2) 179.30(19), C(1)–C(2)–C(21) 175.8(2), C(13)–C(12)–C(11) 176.52(18), C(12)–C(11)–C(36) 177.9(2), C(1)–C(13)–C(12) 117.91(15), C(14)–C(3)–C(4) 171.3(2), C(3)–C(4)–C(22) 177.5(2), C(14)–C(9)–C(10) 178.8(2), C(9)–C(10)–C(31) 174.2(2), C(3)–C(14)–C(9) 117.59(15); ORTEP ellipsoids at the 30% probability level, hydrogen atoms omitted for clarity. CCDCs 1894937 (**4a**), 1894939 (**4b**), 1894938 (**5**)

Single crystals of the series of Type 2 GyNRs were obtained, including radiaannulenes **4a** and **4b**, as well as dimer **5** (Fig. 4a–c). A comparison of bond lengths in the series of structures shows few discernible trends. Overall, the endocyclic alkyne bonds of all three structures are close to linear, with the only notable of exception of angle C(14)–C(3)–C(4) 171.3(2)° (**5**). The exocyclic alkylidene angles in **4a** and **5** (C(31)–C(2)–C(41) and C(5)–C(15)–C(7), respectively), at 116.0 and 115.7°, are distorted from the ideal of 120°. The corresponding endocyclic alkylidene bonds of **4a** and **5** are 117–118°, the analogous value found for both the exo- and endocyclic alkylidene angles of arylated **4b** (117.4(3) and 118.2(3), respectively). In total, these observations suggest that structure of large GyNRs might show slight distortions from ideal bond angle values at the alkylidene centers, balanced by slight bending of the alkyne units.

The benzo-fused cyclic cores of all three molecules, **4a–b** and **5** demonstrate a nearly planar structure, in spite of small deviations from ideal bond angles (Fig. 4a–c). The molecules pack in a slip-stacked fashion (see Supplementary Figs. 78–80), and intermolecular packing is dramatically affected by molecular size and terminal substitution. With intermolecular separation of 3.60 Å, radiaannulene **4a** shows the largest interplanar distance, presumably due to the large ratio of sterically demanding TIPS groups relative to the conjugated carbon structure. Increasing the size of the molecular skeleton decreases the interplanar distance to 3.38 Å in **5**. Finally, formal replacement of the TIPS groups with phenyl rings, as in **4b**, approximates the most realistic packing for 6,6,12-graphyne with intermolecular stacking of 3.27 Å, which is slightly less to that found in graphite (3.35 Å)^32^.

### Electronic absorption properties

**Fig. 5:**
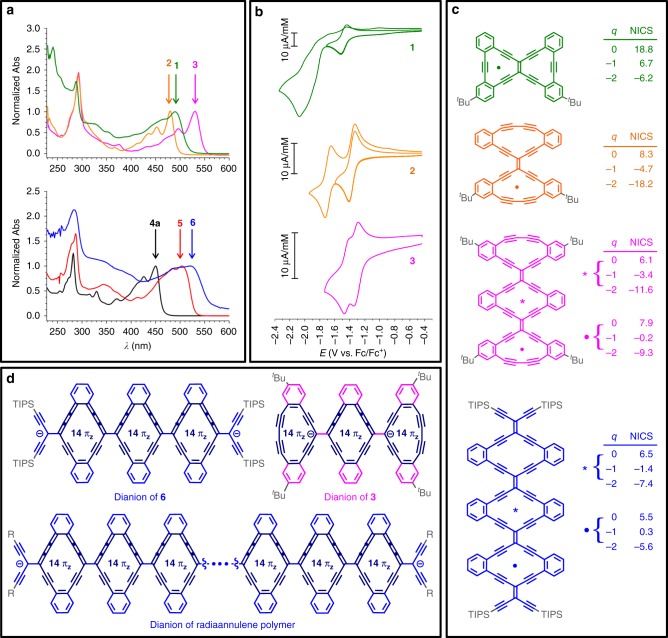
Properties of the compounds. **a** Electronic absorption spectra of **1** (green line), **2** (orange line), and **3** (pink line) in CH_2_Cl_2_ and of **4a** (black line), **5** (red line), and **6** (blue line) in THF. The spectra were normalized based on the longest-wavelength absorption maximum (note, the broadening and tailing of the low energy absorption of **6** could arise from impurities, see the text for additional discussion). **b** Cyclic voltammograms of **1** (green line), **2** (orange line), and **3** (pink line) in CH_2_Cl_2_ (+0.1 M Bu_4_NPF_6_); scan rate 0.1 V s^−1^. Fc = ferrocene. **c** Nucleus independent chemical shift, NICS(1) π_zz_, values calculated for different charge states (*q*) at 1 Å above the center of the rings marked by a solid circle and a star (B3LYP/cc-pvdz; *t*Bu and TIPS groups have been replaced by hydrogen atoms). **d** Canonical forms of the dianions of trimers **3**, **6**, and of an infinite radiaannulene graphyne nanoribbon (GyNR). ^*t*^Bu = *tert*-butyl, TIPS = triisopropylsilyl

The electronic absorption spectra of Type 1 and Type 2 molecules are shown in Fig. 5a (absorption maxima are presented in Supplementary Table 1). The absorption characteristics of bis-dehydrobenzannulene **1** and Type 1 molecules **2**–**3** are analyzed on the basis of both the longest-wavelength absorption maximum (*λ*_max_) and the HOMO-LUMO gap (i.e., *λ*_onset_, which is estimated as the intercept of the tangent line to the lowest-energy absorption with the *x*-axis). Both *λ*_max_ and *λ*_onset_ show sequentially red-shifted values: *λ*_max_, *λ*_onset_ = 480 nm, 496 nm for **2**; 490 nm, 521 nm for **1**; 530 nm, 552 nm for **3** (*λ*_onset_ = 2.50, 2.38, and 2.25 eV, respectively, for **1–3**). The bis-dehydrobenzannulene **1** and dimer **2** have similar HOMO-LUMO gaps, suggesting that structural expansion in either the vertical or horizontal direction (based on Fig. 1) has approximately the same effect, if extrapolated to small GyNRs; *λ*_max_ values of **1** and **2** are also similar to that of radiaannulene **4b** (*λ*_max_ = 483 nm). We note that neither **1** nor **2** exhibit any apparent fluorescence. The effect of the conjugation pathway is evident in the comparison of **1** and **2**. Dimer **1** (linearly conjugated) has four fewer sp-hybridized carbon atoms than dimer **2** (cross-conjugated), and yet *λ*_max_-values are comparable, while that of **1** actually appears at lower energy. DFT calculations reveal that the HOMO and LUMO of **1** (with *t*Bu groups removed for computational convenience) span the entire π-system. On the other hand, while the LUMO encompasses the complete π-system of dimer **2**, the HOMO does not include the butadiyne bridges (see Supplementary Fig. 85). Trimer **3** has the lowest HOMO-LUMO gap in this series, and, thus, expansion from **2** to **3** leads to a reduction of the HOMO-LUMO gap by 0.25 eV. Based on these three molecules, it is projected that the effective conjugation length, i.e., saturation of the electronic absorption trend, has not yet been reached for Type 1 radiaannnulene oligomers with butadiyne edges.

The lowest-energy absorption energy *λ*_max_ and HOMO-LUMO gap (*λ*_onset_) also decrease in the Type 2 series versus oligomer length: from monomer **4a** (*λ*_max_ = 450 nm, *λ*_onset_ = 466 nm), to dimer **5** (*λ*_max_ = 504 nm, *λ*_onset_ = 532 nm), to trimer **6** (*λ*_max_ = 522 nm, *λ*_onset_ = 570 nm (2.66, 2.33, 2.18 eV, respectively, for **4a**, **5–6**). While the spectral characteristics of **6** fit a trend established by **4a** and **5**, it should be noted that the broadening and tailing of the low-energy absorption could result from the presence of impurities due to the limited amount of sample (vide supra). Notably, the shift in HOMO-LUMO gap upon extension from **5** to **6** is relatively small (0.15 eV, based on *λ*_onset_), which indicates that at the stage of the trimer, the effective conjugation length for this series of 6,6,12-graphyne model compounds has been nearly reached. This premise is further supported by a comparison of HOMO-LUMO gaps of Type 1 trimer **3** and Type 2 trimer **6**, which show similar values for *λ*_onset_ (2.25 eV and 2.18 eV, respectively).

### Redox properties

The potential of 6,6,12-graphyne and of GyNRs to function as useful semiconductors is expected to correlate with their redox potentials. Electrochemical analysis of selected molecules and reference compounds by cyclic voltammetry (CV) was therefore conducted, including the series of **1–3** (Table 1 and Fig. 5b). Upon reduction of **1**–**3**, a well-defined one-electron transfer takes place leading to the reversible formation of the radical anion; the peak heights are of similar size for the compounds. Upon further reduction, the dianion is formed, which for **2** and **3** is stable within the experimental timeframe (while a follow-up reaction occurs for the dianion of **1**, probably protonation with residual water, forming products that are reduced at a similar potential as where the radical anion is reduced to the dianion). Compounds **2** and **3** exhibit the smallest reduction potentials, with trimer **3** being 40 mV easier to reduce than dimer **2**, which again is 110 mV easier to reduce than the bis-dehydrobenzannulene **1**. A similar influence of macrocyclic oligomerization is observed when proceeding from Type 2 radiaannulene **4a** (−1.50 V vs. Fc/Fc^+^) to dimer **5** (−1.33 V vs. Fc/Fc^+^), and from radiaannulene **17** (−1.60 V vs. Fc/Fc^+^) to dimer **18** (−1.46 V vs. Fc/Fc^+^), i.e., monomer-to-dimer differences of 170 mV and 140 mV, respectively (voltammograms were measured under slightly different conditions, see Supplementary Fig. 83).

**Table 1:** Electrochemical data—reductions^a^

Compound	*E*_1_°’ [V]	Peak height for *E*_1_°’ [μA mM^–1^]	*E*_2_°’ [V]	*E*_2_^p^ [V]
**1**	−1.48	12.8	–	−2.07
**2**	−1.37	14.1	−1.69	−1.73
**3**	−1.33	10.1	−1.44	−1.47
**1** **4**	−1.61	9.1	–	−2.04

*E°’   *reduction potential*, E*^p^   peak position

^a^Measurements were performed by cyclic voltammetry of compounds dissolved in CH_2_Cl_2_ (Bu_4_NPF_6_) using a glassy carbon working electrode and Fc/Fc^+^ as the external reference

It is clear that the size of the π-system plays a role for the acceptor strength. Thus, the first reduction potential of **2** and **3** differs by only 40 mV, while the second reduction differs by as much as 250 mV; this is reasonable based on electrostatic repulsion, in which the two negative charges of the dianion can be further separated in **3** than in the smaller **2**. Moreover, the facile reduction of the radiaannulene oligomers is attributed to the inherent acceptor strength of the TEE unit, which has been documented in the literature^33,34^. Notably, the radiaannulenes are significantly stronger acceptors than tribenzocyclyne^35^ (−2.21 V vs. Fc/Fc^+^) that can be seen as a macrocyclic fragment of γ-graphyne.

Oxidations of Type 1 molecules occur irreversibly, and the data are therefore more difficult to interpret. Oxidations occurred for **1**–**3** at peak potentials of 1.13 V, 1.10 V, and 0.82 V (vs. Fc/Fc^+^), respectively. For trimer **3**, this would correspond to an electrochemical HOMO-LUMO gap of 2.15 eV, which is slightly lower than the optical gap of 2.25 eV (based on *λ*_onset_).

In summary, the electrochemical studies show that radiaannulene oligomers of both Type 1 and Type 2 are excellent electron acceptors, and we predict that 6,6,12-graphyne would stand out as a particularly good electron acceptor in comparison with other graphyne allotropes.

### Nucleus independent chemical shift (NICS) calculations

To explore the remarkable ability of the molecules to accommodate electrons, a calculational study was explored using NICS^36^. According to Hückel’s rule of aromaticity, the radiaannulene cores are 14 π_z_-aromatic upon reduction. NICS(1) π_zz_ values are listed in Fig. 5c for the various charge states, in which NICS(x) designates an evaluation of aromaticity at a distance of x Å above the molecule. Upon reduction of trimers **3** and **6** to their dianions, the NICS values transform from positive to negative, indicating gain in aromaticity of all three rings. As shown in Fig. 5d, canonical forms with three 14 π_z_-aromatic rings can be drawn for these dianions^37^. In fact, for an infinite oligomer, a two-electron reduction could afford an entirely aromatized structure, as illustrated in Fig. 5d. Extrapolating from this observation suggests that a neutral GyNR would also begin to build up diradical character at the edges, stabilized by the resultant aromatic character imparted to the core. This is analogous to observed behavior of GNRs^38^. It should be noted, however, that this “aromatization” disturbs the benzenoid character of the system, and resonance forms with intact benzene rings (Clar sextets) are expected to contribute as well to the resonance hybrid. Interestingly, the NICS calculations reveal that, upon two-electron reduction, the rings of **3** gain more aromaticity than the rings of **6**. The efficient π-electron delocalization skeleton of **3** upon reduction is confirmed by geometry calculations, which show a distinct decrease in bond length alternation through shortening of single bonds and lengthening of double and triple bonds, as schematically suggested by the resonance form shown in Fig. 5d (see Supplementary Fig. 84 and Supplementary Table 8).

Calculations also allow an estimation of molecular size for the radiaanulene oligomers. Trimer **3** has calculated length of 1.7 nm (distance calculated from center of the two butadiyne edges), and for trimer **6** the length is estimated as 2.6 nm (the distance calculated between distal carbon atoms of the terminal alkyne units bearing the TIPS groups).

## Discussion

In conclusion, radiaannulene oligomers that represent significant sections of 6,6,12-graphyne have been synthesized by iterative acetylenic cross-coupling reactions and studied in either solution or the solid state. Only the Type 2 trimer shows limited stability in solution (chloroform), while the Type 1 trimer was stable and shows particularly easy reduction to mono- and dianions. When proceeding from neutral trimer to its dianion (**3** and **6**), the generation of three aromatic rings is suggested from NICS calculations. For an oligomer composed of *n* radiaannulene rings, the same number *n* of aromatic octadehydro[14]annulene rings could be generated upon two-electron reduction. In fact, we speculate that a long GyNR may be described also in the neutral state as a sequence of 14π-aromatic rings, with an unpaired electron at each of the edges (diradical). X-ray crystallography of Type 2 mono- and dimers predict a planar framework for 6,6,12-graphyne, as would be expected, in spite of the potential of small deviations from ideal interatomic angles, most notably the alkylidene enediyne angles which appear to tend toward 118°. Furthermore, the interplanar packing for 6,6,12-graphyne (≤3.27 Å) is predicted to be slightly less than that of graphene (3.35 Å).

From systematic optical studies and *λ*_onset_ values, we predict the HOMO-LUMO gap of an infinitely long GyNR to be not much smaller than 2.2 eV. This experimentally predicted HOMO-LUMO gap is significantly larger than that predicted from theoretical models of 6,6,12-graphyne, for which two Dirac points are expected (i.e., no band gap). A comparison of the two types of oligomers reveals clear edge effects in the electronic properties, but in an infinite polymer, such effects are expected to be eliminated.

The synthetic protocols that have been developed in this study would allow for vertical extension to longer GyNRs. It seems more relevant, however, to target expansion in the horizontal direction. UV–vis absorption studies show a lower HOMO-LUMO gap for bis-dehydrobenzannulene **1** than for the Type 1 dimer **2**, despite the larger π-system of the latter. Thus, fusing radiaannulene oligomers along the lateral edges would introduce the bis-dehydrobenzannulene fragment (Fig. 1a), leading to increased electronic delocalization as expected for extended GyNRs. Concurrently, the question remains, how many repeat units are required in the horizontal direction to make a GyNR analogue of 6,6,12-graphyne? The answer to this is not yet known and will clearly challenge current synthetic methods to introduce the additional functionality needed to allow expansion of the GyNR in two dimensions. Indeed, the complicated, rectangular symmetry of 6,6,12-graphyne calls for a classical synthetic approach rather than the otherwise convenient on-surface polymerization of suitable molecular precursors that has attracted attention in recent years for synthesis of GNRs^4–6^ and recently also for 30,30,30-graphyne^39^.

## Methods

### Synthesis, optical and electrochemical studies

The syntheses of all target molecules and precursors are provided in Supplementary Methods, which also gives details on the optical and electrochemical studies.

### Computations

Geometry optimizations on neutral molecules, radial anions, and dianions were performed on structures where the *t*Bu and TIPS groups had been substituted for hydrogen atoms at the B3LYP/cc-pVDZ level of theory (an optimization of **14** with TIPS groups, but without *t*Bu groups, was also performed). Frontier molecular orbitals are shown in Supplementary Fig. 85, and were obtained at the B3LYP//cc-pVDZ//CAM-B3LYP/6-31 g(d, p) level of theory.

## Supplementary information

Supplementary Information

## Data Availability

List of figures that have associated raw data: Fig. 4 (the X-ray crystallographic coordinates for structures reported in this study have been deposited at the Cambridge Crystallographic Data Centre (CCDC), under deposition numbers CCDCs 1894937 (**4a**), 1894939 (**4b**), 1894938 (**5**); these data can be obtained free of charge from The Cambridge Crystallographic Data Centre via www.ccdc.cam.ac.uk/data_request/cif); Fig. 5 (output files from computations are provided in Supplementary Methods under “Computational study”).
