# Supplementary material for: Synthesis of radiaannulene oligomers to model the elusive carbon allotrope 6,6,12-graphyne
Source: Nat Commun. 2019 Aug 16;10:3714. doi: 10.1038/s41467-019-11700-0 (PMC6697750; doi:10.1038/s41467-019-11700-0)
Supplement: Supplementary file 1 — Supplementary Information [file 41467_2019_11700_MOESM1_ESM.pdf]

Synthesis of graphyne nanoribbons to model the elusive carbon allotrope 6,6,12-graphyne

Martin Drøhse Kilde,[a] Adrian H. Murray,[b] Cecilie Lindholm Andersen,[a] Freja Eilsø Storm,[a] Katrin Schmidt,[c] Anders Kadziola,[a] Kurt V. Mikkelsen,[a] Frank Hampel,[c] Ole Hammerich,[a] Rik R. Tykwinski,[b,]* Mogens Brøndsted Nielsen [a,]*

a) Department of Chemistry, University of Copenhagen, Universitetsparken 5, DK-2100 Copenhagen Ø, Denmark. E-mail: mbn@chem.ku.dk

b) Department of Chemistry, University of Alberta, Edmonton, AB, Canada T6G 2G2.E-mail: rik.tykwinski@ualberta.ca

c) Department für Chemie und Pharmazie, Friedrich-Alexander-Universität Erlangen-Nürnberg (FAU), Nikolaus-Fiebiger-Straße 10, 91058 Erlangen, Germany

Supplementary Information

TABLE OF CONTENTS

Supplementary Methods ………………………………………….. 2

General methods - synthesis and characterization ……….……… 2

Synthesis – protocols …………………………………….……...…. 3

Spectra ………………………………….…………………… 29

X-Ray crystallographic analysis …………………………...….... 96

UV–Vis absorption spectra ………………………..…………….… 102

Cyclic voltammetry ……………………………………………...…. 103

Computational study ………………………...………….…...……... 106

Supplementary References …………………………………..… 130

Supplementary Methods

General methods – synthesis and characterization

All reagents and solvents were obtained from commercial suppliers and used as received unless otherwise stated. Anhydrous THF was collected from an IT (Innovative Technology) installation of the model PS-MD-05 or distilled from sodium/benzophenone. Hexanes, CH_2Cl_2, and MeCN were either distilled from CaH_2 or used as HPLC grade solvents. $(i\text{Pr})_2NH$ was distilled from NaOH pellets; ethyl formate was distilled from molecular sieves (4Å); Et_3N was typically distilled from NaOH pellets. All air and moisture sensitive reactions were carried out under an inert atmosphere (either nitrogen or argon gas). Purification was carried out by column chromatography on silica gel or alumina, or using biobeads for size exclusion chromatography. Thin-layer chromatography (TLC) was carried out using commercially available aluminum sheets pre-coated with silica gel with a fluorescence indicator and visualized under UV light at 254 or 365 nm. ^{1}H and ^{13}C NMR spectra were recorded on either 300 MHz, 400 MHz or 500 MHz instruments. ^{31}P NMR spectra were recorded on a 500 MHz instrument equipped with a broad-band probe. ^{13}C-NMR data are reported with 2 decimals when recorded on instrument with cryoprobe. Chemical shift values are quoted in ppm and coupling constants (J) in Hz. ^{1}H and ^{13}C NMR spectra are referenced against residual solvent peaks. MALDI spectra were recorded using either dithranol as matrix or *trans*-2-[3-(4-*tert*butylphenyl)-2-methyl-2-propenylidene]malononitrile (DCTB). Samples for ESI mass spectrometry were dissolved in $ClCH_2CH_2Cl$, and a 3:1 MeOH/toluene mixture was used as the carrier solvent. Samples for APPI mass spectrometry were dissolved in THF/MeOH. Crystallographic analysis was performed using a Bruker D8-venture diffractometer using Kα (Mo) radiation or using a Nonius KappaCCS diffractometer. UV–Vis absorption measurements were performed in a 1 cm path-length cuvette, and the neat solvent was used as baseline; sh = shoulder.

Synthesis – protocols

Synthesis - starting materials and molecules presented in Figure 2 (*in article*)

Synthesis of building block S4

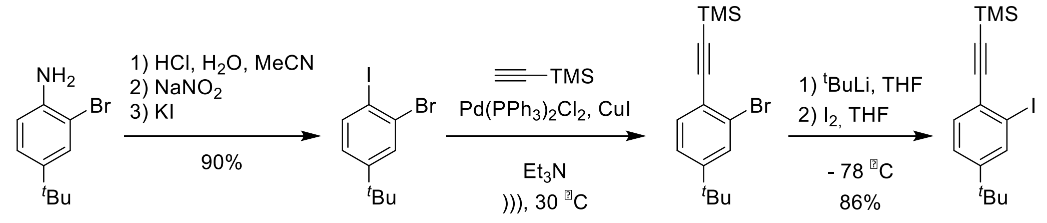

S1 **S2** **S3** **S4**

2-Bromo-4-*tert*-butyliodobenzene (S2)

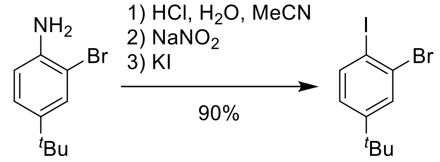

S1 **S2**

Modified relative to literature procedure.[1]

To a solution of 2-bromo-4-*tert*-butylaniline **S1** (25 g, 110 mmol) in MeCN (320 mL) was added aq. HCl (60 mL conc. HCl in 200 mL water), then the mixture was cooled to 0 °C, and a solution of $NaNO_2$ (9.6 g, 140 mmol) in water (200 mL) was added. The reaction mixture was stirred at 0 °C for 1 h after which a solution of KI (29 g, 180 mmol) in water (200 mL) was added carefully. The temperature was voluntarily raised to rt overnight, and the mixture was then poured into a saturated aq. solution of $Na_2S_2O_3$ (400 mL) and extracted with CH_2Cl_2 (4 x 200 mL). The combined organics was dried with $MgSO_4$, filtered, and the solvents were removed *in vacuo*. Purification by distillation (126–129 °C, 0.46 mbar) afforded **S2** (31.3 g, 92.3 mmol, 84%) as an orange oil. Less pure fractions (138–140 °C, 0.46 mbar) was also collected (2.1 g, 6.2 mmol, 6%) as a dark orange oil, which had sufficient purity for further use. ^{1}H NMR (500 MHz, $CDCl_3$) δ 7.75 (d, J = 8.3 Hz, 1H), 7.63 (d, J = 2.3 Hz, 1H), 7.02 (dd, J = 8.3, 2.3 Hz, 1H), 1.29 (s, 9H) ppm. ^{13}C NMR (126 MHz, $CDCl_3$) δ 153.57, 139.93, 130.21, 129.66, 126.12, 97.21, 34.86, 31.16 ppm.

2-Bromo-4-*tert*-butyl(trimethylsilylethynyl)benzene (S3)

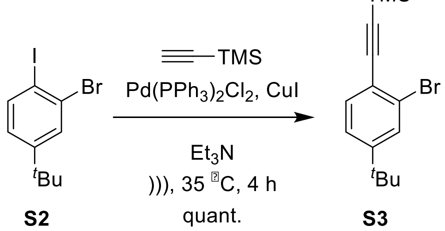

S2 **S3**

To an argon flushed solution of **S2** (7.07 g, 20.9 mmol) in Et_3N (150 mL) were added trimethylsilylacetylene (3.0 mL, 22 mmol), $Pd(PPh_3)_2Cl_2$ (150 mg, 0.21 mmol), and CuI (80 mg, 0.42 mmol). The reaction mixture was subjected to ultrasonication, 35 °C. After 4h, TLC (heptanes) showed no starting material (*Note: In the case when starting material **S2** remained, additional trimethylsilylacetylene was added, and the reaction was carefully followed by TLC analysis*). When judged complete, the reaction mixture was filtered through a silica plug (SiO_2 0.43–0.63 µm, heptanes as eluent). The filtrate was concentrated *in vacuo*, which gave crude **S3** (6.9 g, quantitative) as a viscous dark oil with a reddish glow. ^{1}H NMR (500 MHz, $CDCl_3$) δ 7.56 (d, J = 1.8 Hz, 1H), 7.40 (d, J = 8.1 Hz, 1H), 7.24 (dd, J = 8.2, 1.9 Hz,

1H), 1.28 (s, 9H), 0.26 (s, 9H) ppm. ^{13}C NMR (126 MHz, $CDCl_3$) *δ* 153.68, 133.32, 129.60, 125.76, 124.28, 122.37, 103.40, 98.77, 35.03, 31.15, 0.04 ppm. GC-MS (EI): *m/z* = 308 ([$M^{•+}$]; $C_{15}H_{21}{}^{79}BrSi^+$), 310 ([$M^{•+}$]; $C_{15}H_{21}{}^{81}BrSi^+$). HRMS (APCI): *m/z* = 308.05901 [$M^{•+}$], calcd. for ($C_{15}H_{21}{}^{79}BrSi^{•+}$): 308.05904.

2-Iodo-4-*tert*-butyl(trimethylsilylethynyl)benzene (S4)

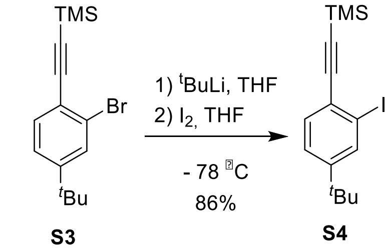

To a stirred solution of crude **S3** (20.9 mmol) in dry THF (150 mL) kept at –78 °C was added *t*BuLi (30 mL, 51 mmol, 1.7 M, 2.4 equiv) in pentane dropwise over the course of 10 min. The resulting dark red reaction mixture was then stirred at –78 °C for 2 h, and then a solution of iodine (26.5 g, 104 mmol, 5 equiv) in THF (80 mL) was added dropwise over the course of 10 min, and the resulting purple solution was stirred overnight while allowed to slowly reach rt. The reaction mixture was diluted with Et_2O (200 mL) and washed with a saturated aq. solution of $Na_2S_2O_3$ (2 x 200 mL), water (200 mL), and brine (200 mL). The combined organic phases were dried with $MgSO_4$, filtered and concentrated *in vacuo*. The resulting residue was filtered through a silica plug (SiO_2 0.43–0.63 µm, heptanes as eluent) to afford **S4** (6.42 g, 18.0 mmol, 86%) as a dark oil. ^{1}H NMR (500 MHz, $CDCl_3$) *δ* 7.82 (d, *J* = 1.9 Hz, 1H), 7.38 (d, *J* = 8.2 Hz, 1H), 7.29 (dd, *J* = 8.2, 1.9 Hz, 1H), 1.28 (s, 9H), 0.27 (s, 9H) ppm. ^{13}C NMR (126 MHz, $CDCl_3$) *δ* 153.51, 135.95, 132.41, 126.86, 125.14, 106.84, 101.57, 97.97, 34.86, 31.16, 0.02 ppm. GC–MS (EI): *m/z* = 356 [$M^{•+}$]. HRMS (APCI): *m/z* = 356.04515 [$M^{•+}$], calcd. for ($C_{15}H_{21}ISi^{•+}$): 356.04517.

Compound **S5**

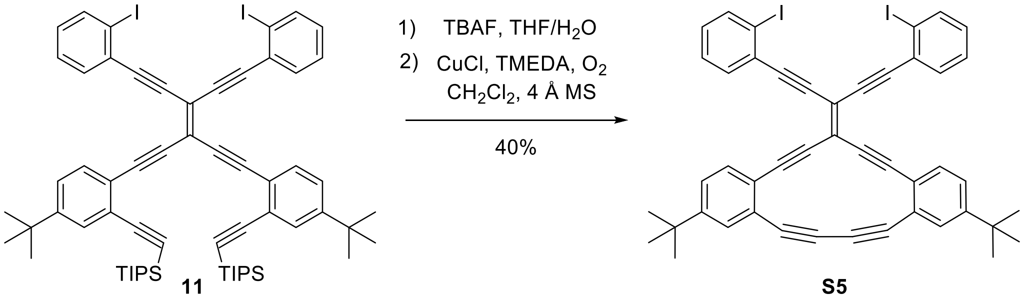

To a solution of **11** (62 mg, 0.054 mmol) in THF (15 mL) and 3 drops of H_2O was added a solution of TBAF (1M in THF, 0.2 ml, 0.2 mmol). The mixture was stirred at rt for 2 h, until quantitative conversion was detected by TLC (SiO_2 20% CH_2Cl_2/heptane). The reaction mixture was passed through a short plug of silica (SiO_2, CH_2Cl_2) and concentrated *in vacuo* until the total volume was ca. 10 mL. The obtained solution of the desilylated product was diluted with CH_2Cl_2 (200 mL), and 4 Å molecular sieves (8 mg), a solution of TMEDA (0.2 ml, 1.3 mmol), and CuCl (20 mg, 0.10 mmol) in CH_2Cl_2 (10 mL) were added. The reaction mixture was stirred vigorously for 72 h under open air, and then it was passed through a short plug of silica (0.43–0.63 µm SiO_2, CH_2Cl_2) and concentrated *in vacuo*. Size exclusion column chromatography (Biobeads, S–X3, CH_2Cl_2) gave compound **S5** (18 mg, 40%) as a yellow oil. R_f = 0.30 (20% CH_2Cl_2/heptane). ^{1}H NMR (500 MHz, $CDCl_3$) *δ* 7.93 (dd, *J* = 8.0, 0.9 Hz, 2H), 7.64 (dd, *J* = 7.7, 1.6 Hz, 2H), 7.53 (d, *J* = 8.2 Hz, 2H), 7.41–7.38 (m, 4H), 7.36 (dd, *J* = 8.2, 2.0 Hz, 2H), 7.08 (ddd, *J* = 8.0, 7.5, 1.6 Hz, 2H), 1.31 (s, 18H) ppm. ^{13}C NMR (126 MHz, $CDCl_3$) *δ* 152.68, 139.13, 133.27, 131.97, 130.27, 129.62, 128.02, 126.55, 126.43, 126.35, 125.44, 117.73, 116.15, 101.40, 101.07, 98.47, 92.70, 90.83, 87.46, 81.18, 35.12, 31.10 ppm. HRMS (MALDI+ FT–ICR, dithranol): *m/z* = 969.52047 [$(M + Na)^+$], calcd. for ($C_{68}H_{74}Si_2Na^+$): 969.52213.

Single crystals suitable for X-ray crystallographic analysis were grown by slow evaporation of a solution of **S5** from a solution of CH_2Cl_2 overlayed with an excess of heptanes at rt.

Di-dehydrobenzannulene 1

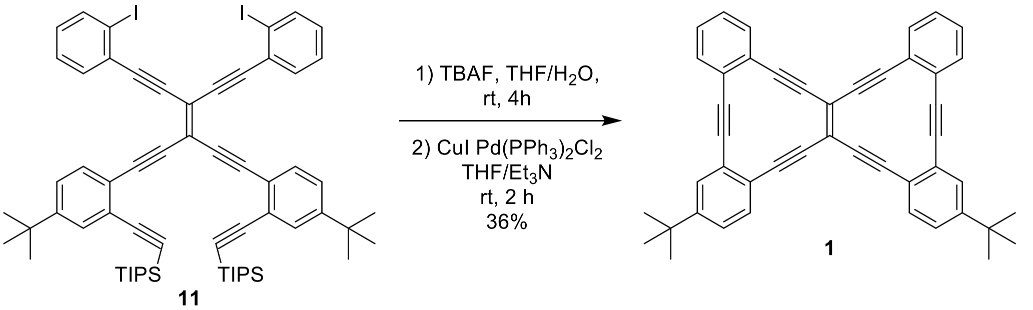

To a solution of **11** (110 mg, 0.095 mmol) in THF (15 mL) and 3 drops of H_2O was added a solution of TBAF (1M in THF, 0.4 mL, 0.4 mmol). The mixture was stirred at rt for 4 h, until quantitative conversion was detected by TLC (SiO_2 20% CH_2Cl_2/heptane). The reaction mixture was passed through a short plug of silica (SiO_2, CH_2Cl_2) and concentrated *in vacuo* until the total volume was ca. 5 mL. To the solution of the desilylated compound were added THF (10 mL) and Et_3N (10 mL), and the solution was thoroughly deoxygenated with argon. Then $Pd(PPh_3)_2Cl_2$ (33 mg, 0.047 mmol) and CuI (10 mg,0.053 mmol) were added, and the reaction mixture was stirred at rt for 2h. The reaction mixture was passed through a plug of silica (SiO_2 0.43–0.63 μm, CH_2Cl_2). Purification by flash column chromatography (SiO_2 0.43–0.63 μm, 15% CH_2Cl_2/heptanes, loading on column: CS_2) and size exclusion column chromatography (Biobeads, S-X3, CH_2Cl_2) gave **1** (20 mg, 0.034 mmol, 36%) as an orange solid. R_f = 0.27 (15% CH_2Cl_2/heptane). UV–Vis (CH_2Cl_2): λ_{max} (ε / $M^{-1}cm^{-1}$) 241 (110000), 288 (72700), 313 sh (27400), 351 sh (17900), 470 (31700), 490 (39700) nm. ^1H NMR (500 MHz, $CDCl_3$) δ 7.93 (dd, J = 8.0, 0.9 Hz, 2H), 7.64 (dd, J = 7.7, 1.6 Hz, 2H), 7.53 (d, J = 8.2 Hz, 2H), 7.41–7.38 (m, 4H), 7.36 (dd, J = 8.2, 2.0 Hz, 2H), 7.08 (ddd, J = 8.0, 7.5, 1.6 Hz, 2H), 1.31 (s, 18H) ppm. ^{13}C NMR (126 MHz, $CDCl_3$) δ 152.69, 139.14, 133.28, 131.98, 130.27, 129.63, 128.03, 126.56, 126.43, 126.36, 125.45, 117.75, 116.16, 101.41, 101.08, 98.48, 92.71, 90.84, 87.47, 81.19, 35.13, 31.11 ppm. HRMS (MALDI+ FT-ICR, dithranol): m/z = 584.24912 [$M^{\bullet+}$], calcd. for ($C_{46}H_{32}^{\bullet+}$): 584.24985.

Di-radiaannulene 2

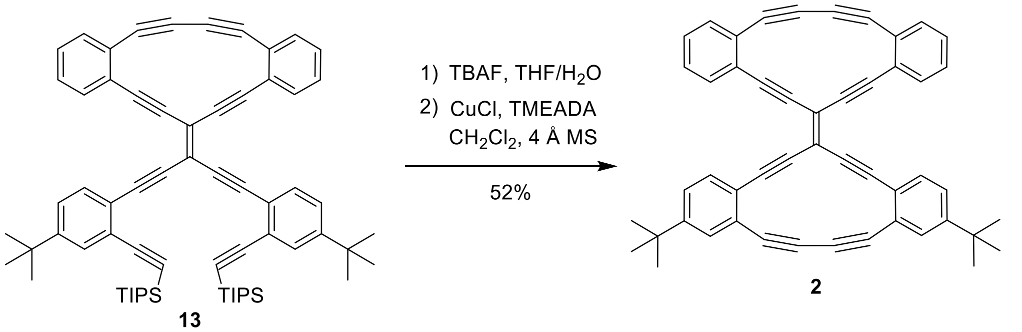

To a solution of **13** (46 mg, 0.049 mmol) in THF (15 mL) and 3 drops of H_2O was added a solution of TBAF (1M in THF, 0.1 mL, 0.1 mmol). The mixture was stirred at rt for 4 h, until quantitative conversion was detected by TLC (SiO_2 20% CH_2Cl_2/heptane). The reaction mixture was passed through a short plug of silica (SiO_2, CH_2Cl_2) and concentrated *in vacuo* until the total volume was ca. 10 mL. The obtained solution of the desilylated product was diluted with CH_2Cl_2 (100 mL), and 4 Å molecular sieves (8 mg), a solution of TMEDA (0.2 mL, 1.3 mmol), and CuCl (20 mg, 0.10 mmol) in CH_2Cl_2 (10 mL) were added. The reaction mixture was stirred vigorously for 40 h under open air, whereafter it was passed through a short plug of silica (0.43–0.63 μm SiO_2, CH_2Cl_2) and concentrated *in vacuo*. Size exclusion column chromatography (Biobeads,

S-X3, CH_2Cl_2) gave compound **2** (16 mg, 0.025 mmol, 52%) as an orange/brown solid. R_f = 0.31 (20% CH_2Cl_2/heptane). UV–Vis (CH_2Cl_2): λ_{max} (ε / $M^{-1}cm^{-1}$) 292 (100000), 367 (7600), 415 sh (13000), 436 (25200), 453 (33600), 480 (53800) nm. ^{1}H NMR (500 MHz, $CDCl_3$) δ 7.61–7.56 (m, 2H), 7.52 (d, J = 8.2 Hz, 2H), 7.43 (dd, J = 8.2, 2.0 Hz, 2H), 7.42–7.36 (m, 6H), 7.35–7.30 (m, 2H), 1.33 (s, 18H) ppm. ^{13}C NMR (126 MHz, $CDCl_3$) δ 152.86, 131.45, 131.33, 129.62, 129.33, 129.07, 129.00, 126.63, 126.57, 126.42, 125.59, 125.48, 117.88, 116.27, 99.36, 98.59, 93.38, 92.85, 87.43, 87.10, 81.70, 81.26, 35.16, 31.12 ppm. HRMS (MALDI+ FT-ICR, dithranol): m/z = 632.24871 [$M^{\bullet+}$], calcd. for ($C_{50}H_{32}^{\bullet+}$): 632.24985.

Tri-radiaannulene 3

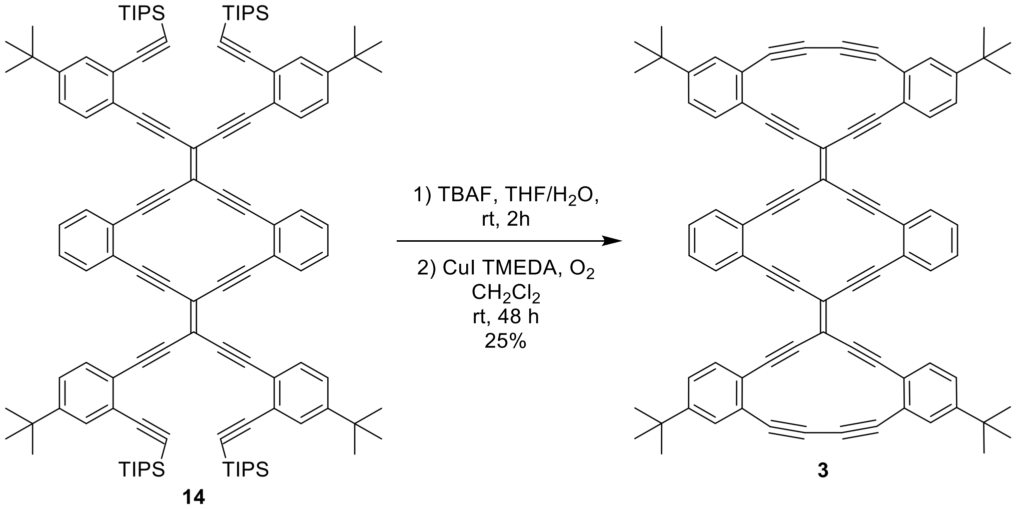

To a solution of **14** (35 mg, 0.021 mmol) in THF (20 mL) and 3 drops of H_2O was added a solution of TBAF (1M in THF, 0.2 ml, 0.2 mmol). The mixture was stirred at rt for 2 h, until quantitative conversion was detected by TLC (SiO_2 30% CH_2Cl_2/heptane). The reaction mixture was passed through a short plug of silica (SiO_2 0.43–0.63 µm, CH_2Cl_2 as eluent) and concentrated *in vacuo* until the total volume was ca. 50 mL. The obtained solution of the desilylated product was diluted with CH_2Cl_2 (100 mL), and 4 Å molecular sieves (8 mg), a solution of TMEDA (0.2 ml, 1.3 mmol), and CuCl (20 mg, 0.10 mmol) in CH_2Cl_2 (10 mL) were added. The reaction mixture was stirred vigorously for 48 h under open air, whereafter it was passed through a short plug of silica (0.43–0.63 µm SiO_2, CH_2Cl_2 as eluent) and concentrated *in vacuo*. Size exclusion column chromatography (Biobeads, S-X3, CH_2Cl_2) gave compound **3** (5.4 mg, 0.005 mmol, 25%) as a red solid. R_f = 0.22 (30% CH_2Cl_2/heptane). UV–Vis (CH_2Cl_2): λ_{max} (ε / $M^{-1}cm^{-1}$) 293 (107000), 375 (11900), 433 sh (9100), 479 sh (22700), 496 (33000), 530 (55700) nm. ^{1}H NMR (500 MHz, $CDCl_3$) δ 7.71 (dd, J = 5.8, 3.4 Hz, 4H), 7.55 (d, J = 8.0 Hz, 4H), 7.48 (dd, J = 5.8, 3.4 Hz, 4H), 7.46–7.42 (m, 8H), 1.34 (s, 36H) ppm. ^{13}C NMR (126 MHz, $CDCl_3$) δ 152.80, 132.27, 131.15, 129.10, 126.68, 126.58, 126.49, 125.73, 125.43, 117.66, 116.57, 98.93, 98.25, 92.96, 92.52, 87.46, 81.25, 35.17, 31.13 ppm. HRMS (MALDI+ FT-ICR, dithranol): m/z = 1016.43792 [$M^{\bullet+}$], calcd. for ($C_{80}H_{56}^{\bullet+}$): 1016.43765.

4-*Tert*-butyl-2-(triisopropylsilylethynyl)(trimethylsilylethynyl)benzene (7)

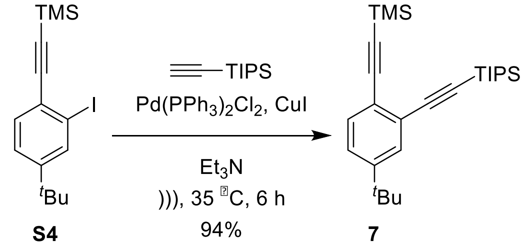

To an argon flushed solution of **S4** (5.04 g, 14.1 mmol) in Et_3N (150 mL) were added triisopropylsilylacetylene (3.8 mL, 17 mmol), $Pd(PPh_3)_2Cl_2$ (220 mg, 0.31 mmol), and CuI (95 mg, 0.50 mmol). The reaction mixture was subjected to ultrasonication, 35 °C. After 6 h, TLC (heptanes) showed no starting material(*Note: In the case when starting material **S4** remained, additional triisopropylsilylacetylene was added, and the reaction was carefully followed by TLC analysis*). When judged complete, the reaction mixture was filtered through a silica plug (SiO_2 0.43–0.63 µm, heptanes as eluent). Purification by flash column chromatography (SiO_2 0.43–0.63 µm, heptanes, loading on column:heptanes) gave **7** (5.48 g, 13.3 mmol, 94%) as a yellow oil. ^{1}H NMR (500 MHz, $CDCl_3$) δ 7.44 (d, J = 2.0 Hz, 1H), 7.39 (d, J = 8.3 Hz, 1H), 7.26–7.24 (m, 1H), 1.29 (s, 9H), 1.16 (s, 21H), 0.23 (s, 9H) ppm. ^{13}C NMR (126 MHz, $CDCl_3$) δ 151.50, 132.82, 129.77, 125.52, 125.47, 122.93, 105.95, 103.77, 97.39, 94.08, 34.81, 31.15, 18.97, 11.48, 0.15 ppm. HRMS (MALDI+ FT-ICR, dithranol): *m/z* = 433.27161 [(M + Na)$^+$], calcd. for ($C_{26}H_{42}Si_2Na^+$): 433.27173.

Compound **9**

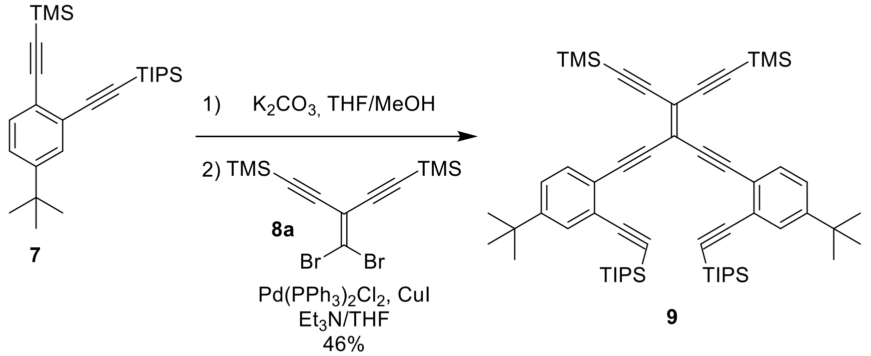

To a stirred solution of **7** (1.58 g, 3.85 mmol) in THF (15 mL) and MeOH (15 mL) was added K_2CO_3 (2.2 g, 16 mmol). The mixture was stirred at rt for 1 h, whereupon TLC (heptanes as eluent) revealed quantitative desilylation. The mixture was filtered through a short silica plug (SiO_2 0.43–0.63 µm, heptanes as eluent). Et_3N (30 mL) was added to the organic phase, which was concentrated in *vacuo* until only Et_3N was left. The desilylated compound was directly subjected to the next step. To the solution of the desilylated compound in Et_3N were added **8a** (422 mg, 1.12 mmol) and THF (30 mL), and the solution was thoroughly deoxygenated with argon. Then $Pd(PPh_3)_2Cl_2$ (87 mg, 0.12) and CuI (37 mg, 0.19 mmol) were added, and the reaction mixture was subjected to ultrasonication, 35 °C, for 16 h. Then it was passed through a plug of silica (SiO_2 0.43–0.63 µm, CH_2Cl_2 as eluent). Purification by flash column chromatography (SiO_2 0.43–0.63 µm, 5% CH_2Cl_2/heptanes to 10% CH_2Cl_2/heptane, loading on column: heptanes) gave **9** (462 mg, 0.517 mmol, 46% based on **8a**) as a yellow oil, which solidified upon standing. R_f = 0.26 (5% CH_2Cl_2/heptane). UV–Vis (CH_2Cl_2): λ_{max} (ε / $M^{-1}cm^{-1}$) 241 (73500), 268 sh (36400), 318 (15600), 349 sh (20000), 375 (27500), 395 (31400) nm. ^{1}H NMR (500 MHz, $CDCl_3$) δ 7.47 (d, J = 1.9 Hz, 2H), 7.40 (d, J = 8.2 Hz, 2H), 7.28 (dd, J = 82, 1.9 Hz, 2H), 1.32 (s, 18H), 1.06 (s, 42H), 0.19 (s, 18H) ppm. ^{13}C NMR (126 MHz, $CDCl_3$) δ 151.88, 132.28, 129.44, 125.78, 125.21, 122.95, 120.77, 117.24, 105.45, 104.82, 101.83, 98.09, 95.08, 89.71, 34.89, 31.17, 18.84, 11.46, –0.05 ppm. HRMS (MALDI+ FT-ICR, dithranol): *m/z* = 915.55417 [(M + Na)$^+$], calcd. for ($C_{58}H_{84}Si_4Na^+$): 915.55423.

Compound **10**

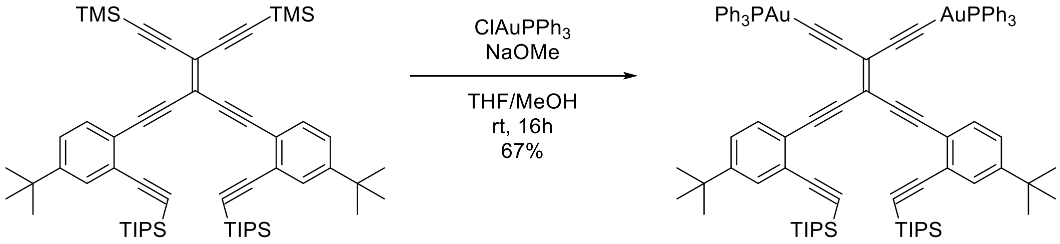

9 **10**

To a solution of **9** (98 mg, 0.11 mmol) and $ClAuPPh_3$ (108 mg, 0.22 mmol) in a mixture of THF (6 mL) and MeOH (5 mL) under argon atmosphere was added a solution of NaOMe/MeOH (0.35 mL, 0.46 mmol – stock solution: 450 mg Na in 15 mL MeOH). The reaction mixture was stirred at rt for 16 h and became a darker yellow color. The reaction mixture was cooled to 0 °C, and MeOH (35 mL) was added, which resulted in a yellow powder precipitating. The powder was collected on a P3 filter with gravity filtration, washed with MeOH (50 mL) and dried with a stream of N_2 after which **10** as a fine yellow solid (122 mg, 0.073 mmol, 67%) was collected. ^{1}H NMR (500 MHz, C_6D_6) δ 7.78 (d, J = 8.3 Hz, 2H), 7.61 (d, J = 1.7 Hz, 2H), 7.22–7.15 (m 12H), 7.02–6.97 (m, 6H), 6.94–6.89 (m, 12H), 6.81 (dd, J = 8.3, 1.7 Hz, 2H), 1.34–1.28 (m, 42H), 1.02 (s, 18H) ppm. ^{13}C NMR (126 MHz, C_6D_6) δ 150.07, 149.00 (d, $J_{P,C}$ = 147 Hz), 134.59 (d, $J_{P,C}$ = 14 Hz)), 133.38, 131.13 (d, $J_{P,C}$ = 2 Hz), 130.68 (d, $J_{P,C}$ = 55 Hz), 129.15 (d, $J_{P,C}$ = 2 Hz), 125.94, 125.82, 125.32, 124.97, 113.81, 107.06, 104.18 (d, $J_{P,C}$ = 28 Hz), 95.25, 94.87, 93.81, 34.43, 31.01, 19.30, 11.95 ppm (one signal missing due to overlap at 129.15 ppm – identified by HSQC NMR). ^{31}P NMR (121 MHz, $CDCl_3$) δ 41.77 ppm. HRMS (MALDI+ FT-ICR, dithranol): m/z = 1665.59372 [(M + H)$^+$], calcd. for ($C_{88}H_{97}Au_2P_2Si_2^+$): 1665.59296.

Compound **11**

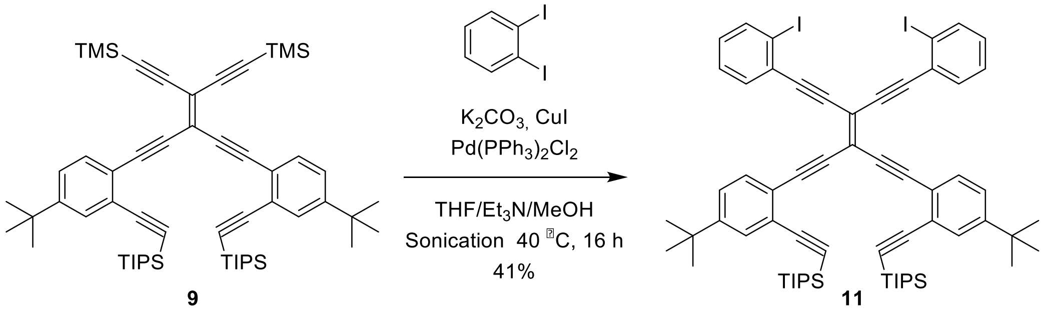

9 **11**

To an argon flushed solution of **9** (360 mg, 0.403 mmol) and 1,2-diiodobenzene (2.5 g, 7.6 mmol) in THF (15 mL), Et_3N (15 mL) and MeOH (10 mL) were added $Pd(PPh_3)_2Cl_2$ (140 mg, 0.20 mmol), CuI (40 mg, 0.21 mmol) and K_2CO_3 (350 mg, 2.5 mmol). The reaction mixture was subjected to ultrasonication, 40 °C, for 16 h. Then the reaction mixture was passed through a plug of silica (SiO_2 0.43–0.63 μm, CH_2Cl_2). Purification by flash column chromatography (SiO_2 0.43–0.63 μm, 10% CH_2Cl_2/heptanes, loading on column:heptanes) gave **11** (189 mg, 0.164 mmol, 41%) as a yellow oil. R_f = 0.23 (10% CH_2Cl_2/heptane). ^{1}H NMR (500 MHz, $CDCl_3$) δ 7.84 (dd, J = 7.9, 1.2 Hz, 2H), 7.51 (dd, J = 7.7, 1.6 Hz, 2H), 7.48 (d, J = 1.9 Hz, 2H), 7.46 (d, J = 8.3 Hz, 2H), 7.29 (dd, J = 8.3, 1.9 Hz, 2H), 7.26 (dd, J = 7.7, 1.2 Hz, 2H), 6.99 (td, J = 7.9, 1.2 Hz, 2H), 1.32 (s, 18H), 1.06 (s, 42H) ppm. ^{13}C NMR (126 MHz, $CDCl_3$) δ 152.01, 138.93, 133.47, 132.47, 129.88, 129.76, 129.52, 127.80, 125.89, 125.35, 122.98, 118.49, 116.96, 105.44, 100.94, 100.33, 98.37, 95.39, 90.88, 90.22, 34.93, 31.18, 18.86, 11.50 ppm. HRMS (MALDI+ FT-ICR, dithranol): m/z = 1175.33121 [(M + Na)$^+$], calcd. for ($C_{64}H_{74}I_2Si_2Na^+$): 1175.33106.

Compound **12**

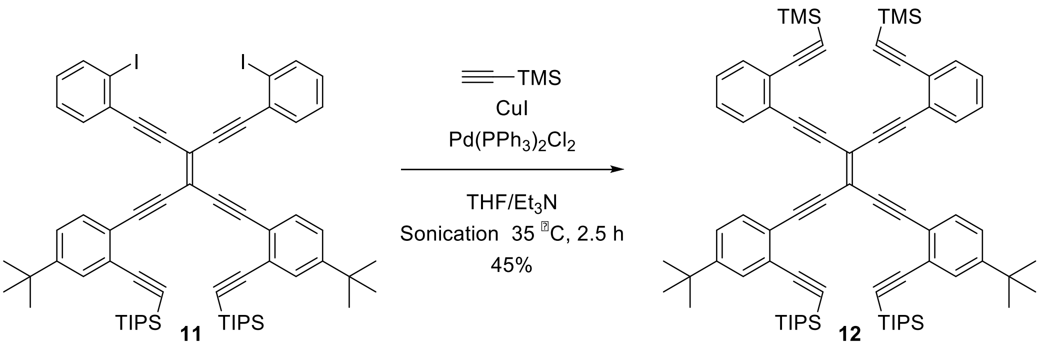

To an argon flushed solution of **11** (114 mg, 0.099 mmol) in THF (10 mL) and Et_3N (10 mL) were added $Pd(PPh_3)_2Cl_2$ (19 mg, 0.027 mmol), CuI (10 mg, 0.053 mmol) and trimethylsilylacetylene (0.20 mL, 1.4 mmol). The reaction mixture was subjected to ultrasonication, 35 °C, for 2.5 h after which it was filtered through a short silica plug (SiO_2 0.43–0.63 µm, 20% CH_2Cl_2/heptanes as eluent). Purification by flash column chromatography (SiO_2 0.43–0.63 µm 10% CH_2Cl_2/heptanes to 15% CH_2Cl_2/heptanes, loading on column: heptanes) gave **12** (49 mg, mmol, 45%) as a yellow oil. UV–Vis (CH_2Cl_2): λ_{max} (ε / $M^{-1}cm^{-1}$) 238 (91600), 252 sh (66000), 315 (22300), 326 (22700), 409 sh (27900), 424 (29000) nm. ^1H NMR (500 MHz, $CDCl_3$) δ 7.48–7.45 (m, 6H), 7.43 (d, J = 8.3 Hz, 2H), 7.24 (dd, J = 8.3, 2.0 Hz, 2H), 7.21 (dd, J = 6.7, 1.4 Hz, 2H), 7.18 (dd, J = 7.5, 1.4 Hz, 2H), 1.31 (s, 18H), 1.10–0.98 (m, 42H), 0.14 (s, 18H) ppm. ^{13}C NMR (126 MHz, $CDCl_3$) δ 151.84, 132.80, 132.70, 132.14, 129.44, 128.33, 128.02, 126.07, 125.77, 125.62, 125.37, 122.93, 118.91, 117.65, 105.48, 103.11, 99.62, 98.12, 97.23, 95.08, 91.17, 90.23, 34.89, 31.17, 18.83, 11.46, –0.05 ppm. HRMS (MALDI+ FT-ICR, dithranol): *m/z* = 1092.62524 [$M^{\bullet+}$], calcd. for ($C_{74}H_{92}Si_4^{\bullet+}$): 1092.62706.

Compound **13**

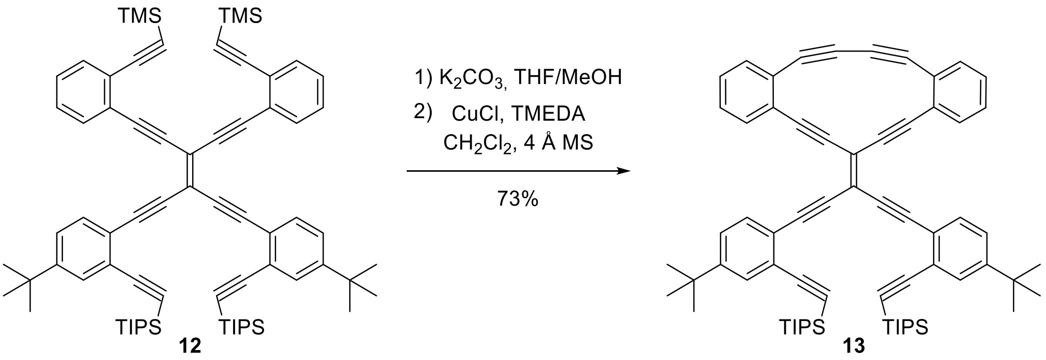

To a solution of **12** (74 mg, 0.068 mmol) in THF (20 mL) and MeOH (20 mL) was added K_2CO_3 (210 mg, 1.52 mmol). The mixture was stirred at rt for 3 h, until quantitative conversion was detected by TLC (SiO_2 15% CH_2Cl_2/heptane). The reaction mixture was passed through a short plug of silica (SiO_2, CH_2Cl_2) and concentrated *in vacuo* until the total volume was ca. 10 mL. The obtained solution of the desilylated product was diluted with CH_2Cl_2 (100 mL), and then 4 Å molecular sieves and a solution of TMEDA (0.2 mL, 1.3 mmol) and CuCl (20 mg, 0.10 mmol in CH_2Cl_2 (10 mL) were added. The reaction mixture was stirred vigorously for 14 h under open air, and then it was passed through a short plug of silica (0.43–0.63 µm SiO_2, CH_2Cl_2) and concentrated *in vacuo*. Size exclusion column chromatography (Biobeads, S-X3, CH_2Cl_2) gave compound **13** (47 mg, 73%) as a yellow solid. R_f = 0.34 (15% CH_2Cl_2/heptane). UV–Vis (CH_2Cl_2): λ_{max} (ε / $M^{-1}cm^{-1}$) 279 sh (29400), 292 (48700), 305 sh (31800), 364 (8400), 438 sh (23900), 456 (27900) nm. ^1H NMR (500 MHz, $CDCl_3$) δ 7.55–7.51 (m, 4H), 7.47–7.43 (m, 2H), 7.38–7.35 (m, 4H), 7.29–7.25 (m, 4H), 1.35 (s, 18H), 1.07–1.02 (m, 42H) ppm. ^{13}C NMR (126 MHz, $CDCl_3$) δ 152.22, 132.24, 131.81, 129.76, 129.71, 129.08, 128.96, 128.64, 125.85, 125.50, 125.32, 122.79, 118.70, 117.20, 105.30,

99.11, 97.52, 95.45, 93.29, 90.23, 87.08, 81.54, 34.98, 31.20, 18.81, 11.49 ppm. HRMS (MALDI+ FT-ICR, dithranol): *m/z* = 969.52047 [$(M + Na)^+$], calcd. for ($C_{68}H_{74}Si_2Na^+$): 969.52213.

Compound **14**

Method 1:

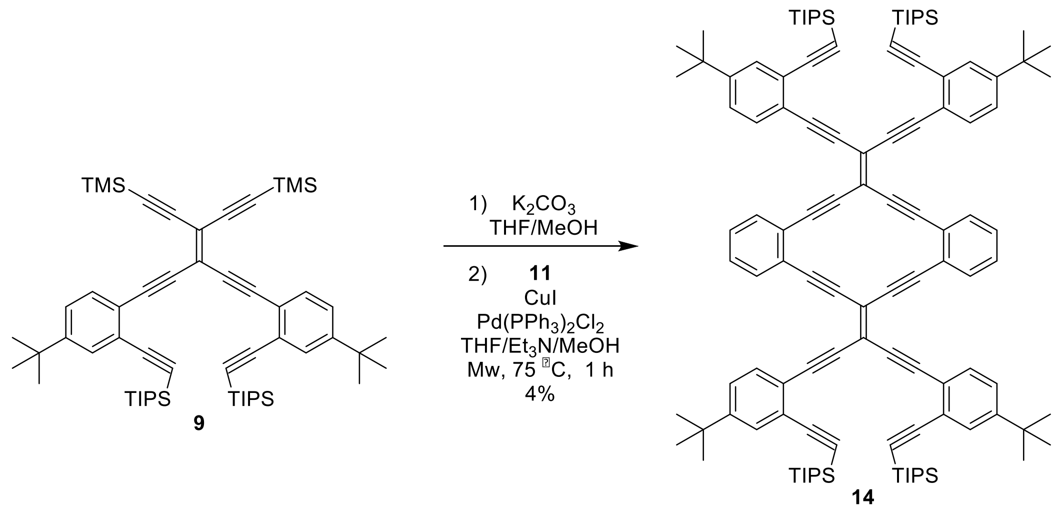

To a stirred solution of **9** (80 mg, 0.090 mmol) in THF (10 mL) and MeOH (10 mL) was added K_2CO_3 (50 mg, 0.36 mmol). The mixture was stirred at rt for 1 h, whereupon TLC (10% CH_2Cl_2/heptanes as eluent) revealed quantitative desilylation. The mixture was filtered through a short silica plug (SiO_2 0.43–0.63 µm, CH_2Cl_2 as eluent) and the solvents evaporated to a minimum amount (4 mL). The desilylated compound was directly subjected to the next step. To the solution of the desilylated compound were added **11** (103 mg, 0.90 mmol), THF (8 mL), Et_3N (8 mL) and MeOH (0.5 mL), and the solution was thoroughly deoxygenated with argon. Then $Pd(PPh_3)_2Cl_2$ (28 mg, 0.040 mmol) and CuI (8 mg, 0.04 mmol) were added, and the reaction mixture was subjected to heating using microwave radiation at 75 °C for 1 h. Then the reaction mixture was passed through a plug of silica (SiO_2 0.43–0.63 µm, CH_2Cl_2 as eluent). Purification by flash column chromatography (SiO_2 0.43–0.63 µm, 15% CH_2Cl_2/heptanes, loading on column: CS_2) gave **14** (6 mg, 0.004 mmol, 4%) as a dark yellow film. R_f = 0.31 (20% CH_2Cl_2/heptane). UV–Vis (CH_2Cl_2): λ_{max} (ε / $M^{-1}cm^{-1}$) 275 sh (38900), 289 (53800), 320 sh (19400), 351 sh (8500), 431 (23200), 454 (25500) nm. ^{1}H NMR (500 MHz, $CDCl_3$) δ 7.55–7.49 (m, 8H), 7.45 (dd, J = 5.8, 3.3 Hz, 4H), 7.34 (dd, J = 8.2, 2.1 Hz, 4H), 7.20 (dd, J = 5.8, 3.3 Hz, 4H), 1.34 (s, 36), 1.04 (s, 84H) ppm. ^{13}C NMR (126 MHz, $CDCl_3$) δ 152.05, 132.46, 132.22, 129.67, 128.58, 125.80, 125.61, 125.46, 122.94, 118.09, 117.69, 105.36, 98.40, 97.36, 95.40, 92.17, 90.38, 34.96, 31.21, 18.83, 11.49 ppm. IR 2959 (s), 2942 (s), 2864 (s), 2185 (w), 2158 (w), 1594 (w), 1485 (m), 1462 (m), 1395 (w), 1364 (w), 1336 (w), 1260 (w), 1200 (w), 1132 (w), 1069 (w), 1018 (w), 996 (w), 924 (m), 883 (m), 831 (w), 756 (m), 711 (m), 676 (m), 666 (m), 643 (m) cm^{-1}. HRMS (MALDI+ FT-ICR, dithranol): *m/z* = 1645.99864 [$M^{\bullet+}$], calcd. for ($C_{116}H_{140}Si_4^{\bullet+}$): 1646.00602.

Compound **14**

Method 2:

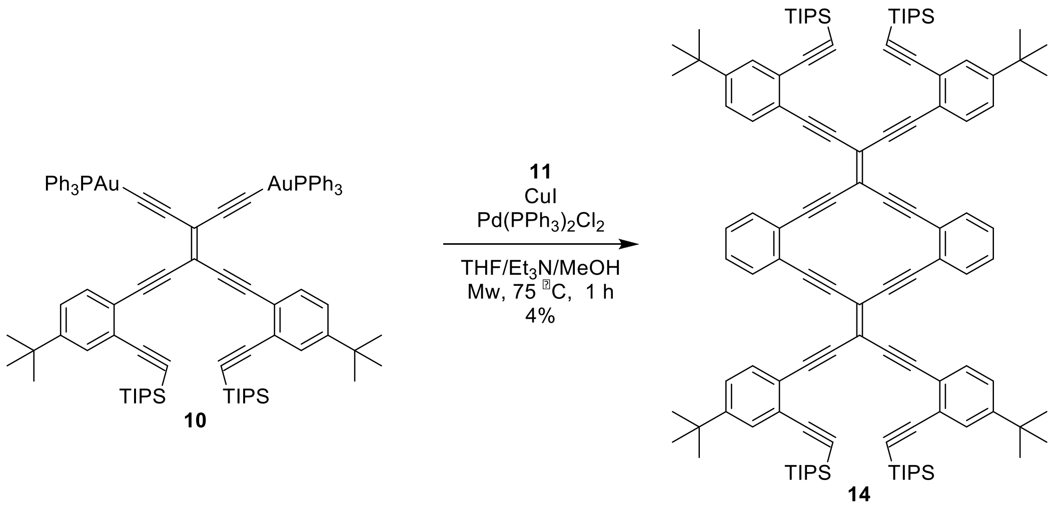

To an argon flushed solution of **10** (88 mg, 0.053 mmol) and **11** (61 mg, 0.053 mmol) in THF (8 mL), Et_3N (8 mL) and MeOH (0.5 mL) were added $Pd(PPh_3)_2Cl_2$ (28 mg, 0.040 mmol) and CuI (8 mg, 0.042 mmol), and the reaction mixture was subjected to heating using microwave radiation at 75 °C for 1 h. Then the reaction mixture was passed through a plug of silica (SiO_2 0.43–0.63 μm, CH_2Cl_2 as eluent). Purification by flash column chromatography (SiO_2 0.43–0.63 μm, 15% CH_2Cl_2/heptanes, loading on column: CS_2) gave **14** (3 mg, 0.002 mmol, 4%) as a dark yellow film.

Synthesis – starting materials and molecules presented in Figure 3 (*in article*)

Synthesis of building block S10

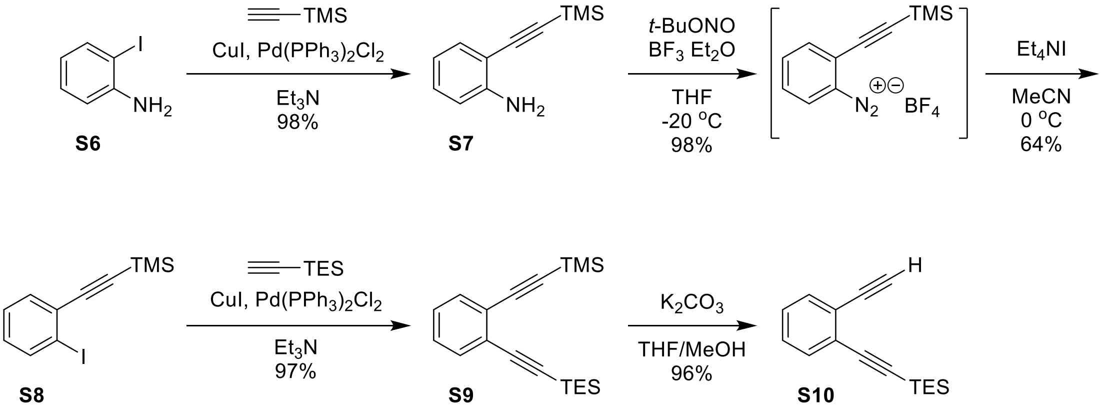

2-(Trimethylsilylethynyl)aniline S7

Synthesized according to literature procedure.[2]

2-(Trimethylsilylethynyl)iodobenzene (S8)

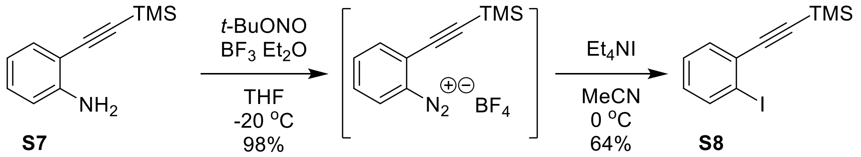

A solution of **S7** (11.973 g, 63.239 mmol) in dry THF (100 mL) was added dropwise to BF_3•Et_2O (32 mL) at –20 °C and stirred for 20 min. To this solution was added dropwise a solution of *t*BuONO (26 mL, 22 g, 220 mmol) dissolved in dry THF (50 mL). The reaction was stirred for 1 h, after which the diazonium salt was precipitated by addition of Et_2O (300 mL). The supernatant was carefully decanted, the solid was dissolved in MeCN (150 mL), and cooled to 0 °C. To this solution was added Et_4NI (20.696 g, 80.482 mmol) in small portions. The reaction was warmed to rt, stirred for 30 min, and quenched via the addition of saturated aq. $Na_2S_2O_3$ (200 mL). The aq. phase was extracted with hexanes (2 x 100 mL). The combined organic phase was washed with H_2O (100 mL) and saturated aq. NaCl (100 mL), dried with $MgSO_4$, filtered, and the solvent removed in *vacuo*. Column chromatography (silica gel, hexanes) afforded **S8** (12.091 g, 64%) as an orange oil. Spectroscopic data match those previously reported.[3]

2-(Trimethylsilylethynyl)(triethylsilylethynyl)benzene (S9)

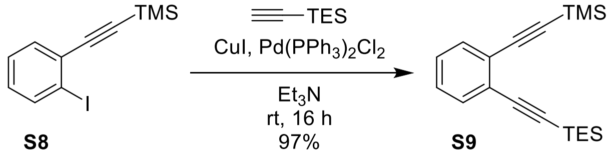

To a solution of **S8** (0.883 g, 2.94 mmol), $Pd(PPh_3)_2Cl_2$ (0.103 g, 0.147 mmol), and CuI (0.055 g, 0.29 mmol) in deoxygenated Et_3N (8 mL) was added triethylsilylacetylene (1.3 mL, 1.0 g, 7.2 mmol). The flask was sealed under argon with a rubber septum and stirred at rt for 16 h. The reaction was quenched via the addition of saturated aq. NH_4Cl (25 mL). The aq. phase was extracted with Et_2O (2 x 25 mL). The combined organic phase was washed with H_2O (20 mL) and saturated aq. NaCl (20 mL), dried with $MgSO_4$, filtered, and the solvent removed in *vacuo*. Column chromatography (silica gel, hexanes) afforded **S9** (0.894 g, 97%) as a yellow oil. R_f = 0.3 (hexanes). IR (CH_2Cl_2 cast film): 3061 (w), 2954 (s), 2911 (m), 2874 (m), 2159 (m), 1476 (m). ^{1}H NMR (300 MHz, $CDCl_3$) δ 7.48–7.41 (m, 2H), 7.23–7.20 (m, 2H), 1.05 (t, J = 7.8 Hz, 9H), 0.68 (q, J = 7.8 Hz, 6H), 0.24 (s, 9H) ppm. ^{13}C NMR (75 MHz, $CDCl_3$) δ 132.64, 132.57 128.0, 127.9, 125.8, 125.6, 104.4, 103.3, 98.3, 96.0, 7.6, 4.4, 0.0 ppm. EI HRMS: m/z = 312.1724, calcd. for ($C_{19}H_{28}Si_2^+$): 312.1729.

2-(Triethylsilylethynyl)ethynylbenzene (S10)

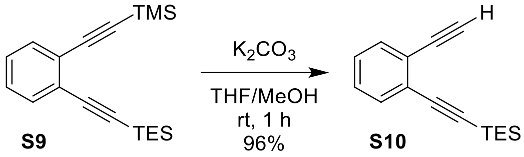

To a solution of **S9** (9.994 g, 31.81 mmol) in THF (5 mL) and MeOH (25 mL) was added K_2CO_3 (4.421 g, 31.99 mmol). The solution was stirred for 1 h after which the reaction was quenched via the addition of saturated aq. NH_4Cl (50 mL). The aq. phase was extracted with Et_2O (2 x 50 mL). The combined organic phase was washed with H_2O (50 mL) and saturated aq. NaCl (50 mL), dried with $MgSO_4$, filtered, and the solvent removed in vacuo. Column chromatography (silica gel, 10:1 hexanes/CH_2Cl_2) afforded **S10** (7.306, 96%) as an orange oil. R_f = 0.25 (10:1 hexanes/CH_2Cl_2). IR (neat film): 3305 (m), 3062 (w), 2954 (s), 2910 (m), 2874 (s), 2158 (s), 1474 (m). ^{1}H NMR (400 MHz, $CDCl_3$) δ 7.50–7.43 (m, 2H), 7.29–7.20 (m,

2H), 3.25 (s, 1H), 1.05 (t, J = 7.8 Hz, 9H), 0.67 (q, J = 7.9 Hz, 6H) ppm. ^{13}C NMR (75 MHz, $CDCl_3$) δ 132.4, 132.3, 128.3, 128.0, 126.4, 125.0, 104.2, 96.4, 82.1, 81.0, 7.5, 4.4 ppm. MS (EI): *m/z* (%) = 240.1 ([$M^{•+}$], 30), 211.1 ([M – Et]$^+$, 100). HRMS (EI): *m/z* = 240.1332 ([$M^{•+}$]), calcd. for ($C_{16}H_{20}Si^{•+}$): 240.1334.

Synthesis of compound S12

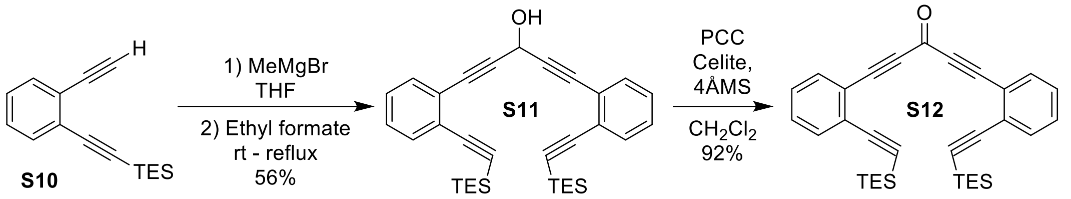

Compound **S11**

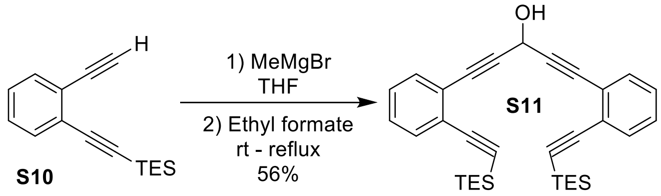

To a solution of **S10** (7.306 g, 30.39 mmol) in dry THF (150 mL) was added MeMgBr (9.2 mL, 28 mmol, 3.0 M in Et_2O). The solution was stirred for 30 min at rt, and ethyl formate (1.0 mL, 0.92 g, 12 mmol) was then added. The solution was gently heated to refluxed and stirred under argon for 1 h. The solution was cooled to rt and the reaction quenched via the addition of saturated aq. NH_4Cl (150 mL). The aq. phase was extracted with Et_2O (2 x 100 mL). The combined organic phase was washed with H_2O (50 mL) and saturated aq. NaCl (50 mL), dried with $MgSO_4$, filtered, and the solvent removed in *vacuo*. Column chromatography (silica gel, 7:1 hexanes/EtOAc) afforded **S11** (3.522 g, 56%) as a yellow oil. R_f = 0.20 (5:1 hexanes/EtOAc). IR (CH_2Cl_2 cast film): 3550–3300 (br, m), 3061 (w), 2955 (s) 2935 (s) 2911 (s) 2875 (s), 2234 (w), 2158 (m) 1478 (m), 1442 (m) ^{1}H NMR (300 MHz, $CDCl_3$) δ 7.47–7.45 (m, 4H), 7.26–7.24 (m, 4H), 5.56 (bs, 1H), 2.39 (bs, 1H), 1.03 (t, J = 8.0 Hz, 18H), 0.64 (q, J = 8.0 Hz, 12H) ppm. ^{13}C NMR (125 MHz, $CDCl_3$) δ 132.2, 132.1, 128.3, 127.9, 125.9, 124.7, 104.1, 96.3, 89.5, 83.1, 53.3, 7.5, 4.3 ppm. HRMS (EI): *m/z* = 508.2620, calcd. for ($C_{33}H_{40}OSi_2^{•+}$): 508.2619.

Compound **S12**

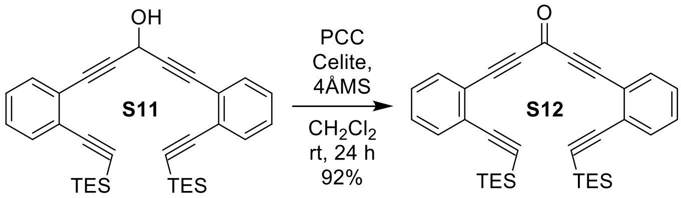

To a solution of **S11** (3.522 g, 6.922 mmol) in dry, deoxygenated CH_2Cl_2 (150 mL) was added 4Å mol. sieves (5 g), Celite (5 g) and PCC (2.249 g, 10.43 mmol). The solution was stirred for 24 h at rt. The reaction was quenched via a plug through silica gel with CH_2Cl_2. Solvent removal in *vacuo* afforded **S12** (3.226 g, 92%) as a yellow oil. R_f = 0.40 (5:1 hexanes/EtOAc). IR (CH_2Cl_2 cast film): 3063 (w), 2955 (s) 2935 (s) 2911 (s) 2875 (s), 2217 (s), 2185 (s) 2160 (m), 1622 (s), 1477 (m) ^{1}H NMR (500 MHz, $CDCl_3$) δ 7.56 (ddd, J = 7.7 Hz, 1.4 Hz, 0.6 Hz, 2H), 7.51 (ddd, J = 7.8 Hz, 1.3 Hz, 0.6 Hz, 2H), 7.4 (td, J = 7.7 Hz, 1.4 Hz, 2H), 7.30 (td, J = 7.3 Hz, 1.4 Hz, 2H), 0.99 (t, J = 7.9 Hz, 18H), 0.64 (q, J = 7.9 Hz, 12H) ppm. ^{13}C

NMR (125 MHz, $CDCl_3$) δ 160.3, 133.1, 132.5, 130.3, 128.0, 127.3, 122.5, 103.0, 98.6, 92.0, 89.6, 7.5, 4.2 ppm. HRMS (MALDI, DCTB): m/z = 507.2534 [$(M + H)^+$], calcd. for ($C_{33}H_{39}OSi_2^+$): 507.2534.

Synthesis of building block S16

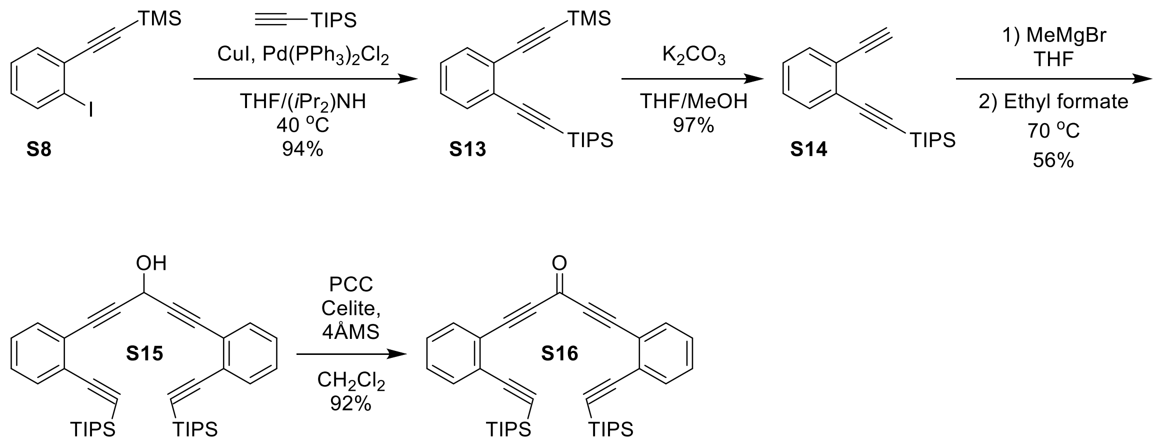

2-(Triisopropylsilylethynyl)-(trimethylsilylethynyl)benzene (S13)

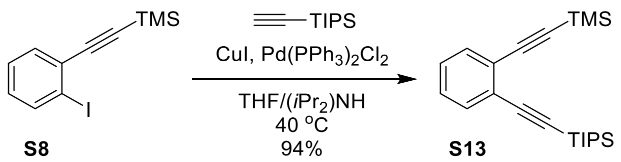

Synthesized according to literature procedure.[4]

2-(Triisopropylsilylethynyl)ethynylbenzene S14

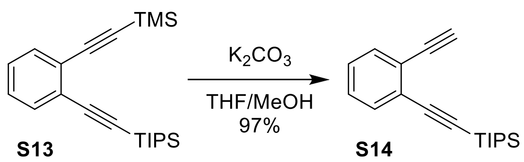

Synthesized according to literature procedures.[4,5]

Compound **S15**

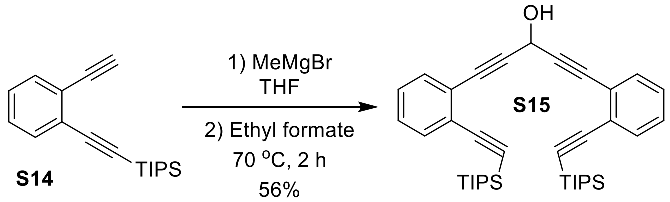

To a solution of **S14** (2.4 g, 8.5 mmol) in dry THF (35 mL) was added MeMgBr (3.0 M in Et_2O, 1.4 mL, 4.3 mmol). The reaction mixture was stirred for 45 min and ethyl formate (0.14 mL, 0.13 g, 1.7 mmol) was added. The reaction mixture was heated to 70 °C and stirred for 2 h. The reaction mixture was allowed to cool to rt, Et_2O (20 mL), and saturated aq. NH_4Cl (20 mL) were added. The organic layer was separated, washed with saturated aq. NH_4Cl (2 x 25 mL), H_2O (2 x 25 mL), brine (2 x 25 mL), dried ($MgSO_4$), filtered, and the solvent removed in *vacuo*. After column chromatography (silica, hexanes/CH_2Cl_2 10:1 to 1:1) **S15** was obtained (0.566 g, 56%) as a yellow oil and more than 85% of the unreacted **S14** starting material were recovered. R_f = 0.33 (silica, CH_2Cl_2/hexanes 1:1). IR (ATR) 3597−3212 (br w), 3060 (w), 2940 (m), 2890 (m), 2862 (s), 2156 (m), 1462 (m) cm^{-1}. ^{1}H NMR (300 MHz, $CDCl_3$) δ 7.49−7.46 (m, 4H), 7.28−7.23 (m, 4H), 5.60 (d, J = 5.3 Hz, 1H), 2.48 (d, J = 6.5 Hz, 1H), 1.15 (m, 42H) ppm. ^{13}C NMR (75 MHz, $CDCl_3$) δ 132.5, 132.4, 128.3, 127.9, 126.1, 124.7, 104.9, 95.2, 89.4, 83.3, 53.4, 18.7, 11.2 ppm. HRMS (APPI): *m/z* = 592.3531 [$M^{\bullet+}$], calcd. for ($C_{39}H_{52}OSi_2^{\bullet+}$): 592.3557.

Compound **S16**

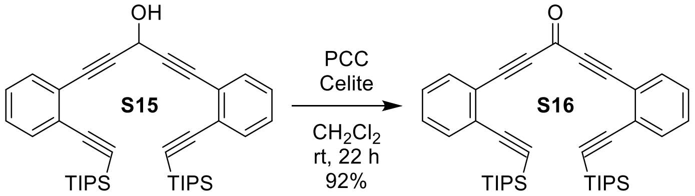

A solution of **S15** (4.6 g, 7.8 mmol), PCC (2.9 g, 13 mmol), and celite (7.9 g) in CH_2Cl_2 (500 mL) was stirred until TLC analysis no longer indicated the presence of **S15** (22 h). The reaction mixture was filtered through a plug of silica (CH_2Cl_2), the solvent was evaporated in *vacuo*, and the product **S16** was obtained as an orange oil (4.24 g, 92%). R_f = 0.59 (silica, CH_2Cl_2/hexanes 1:1). IR (ATR) 3062 (w), 2940 (m), 2890 (m), 2862 (s), 2213 (m), 2183 (m), 2157 (m), 1621 (s), 1462 (m) cm^{-1}. ^{1}H NMR (300 MHz, $CDCl_3$) δ 7.57−7.51 (m, 4H), 7.40−7.23 (m, 4H), 1.08 (s, 42H) ppm. ^{13}C NMR (75 MHz, $CDCl_3$) δ 160.3, 133.2, 132.8, 130.2, 128.0, 127.3, 122.5, 103.8, 97.4, 91.9, 89.6, 18.6, 11.2 ppm. HRMS (APPI): *m/z* = 591.3466 [$(M + H)^+$], calcd. for ($C_{39}H_{51}OSi_2^+$): 591.3473.

Compound **S17**

Method 1:

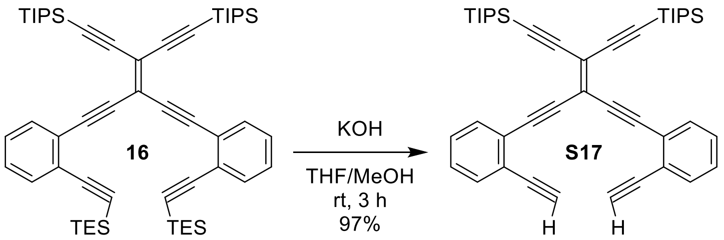

To a solution of **16** (62 mg, 0.079 mmol) in THF (3 mL) and MeOH (8 mL) was added aq. KOH (0.2 mL, 0.2 mmol, 1.0 M). The reaction was stirred at rt for 3 h, and then quenched via the addition of saturated

aq. NH_4Cl (15 mL). The aq. phase was extracted with Et_2O (2 x 10 mL). The combined organic phase was washed with H_2O (10 mL) and saturated aq. NaCl (10 mL), dried with $MgSO_4$, filtered, and the solvent removed *in vacuo*. Column chromatography (silica gel, 4:1 hexanes/CH_2Cl_2) afforded **S17** (49 mg, 97%) as an amber oil. UV–Vis (THF): λ_{max} (ε / $M^{-1}cm^{-1}$) 294 (17500), 314 (16800), 368 (31400), 384 (31300) nm. IR (ATR) 3295 (w), 3055 (w), 2942 (m), 2893 (m), 2864 (m), 2185 (w), 2140 (w), 1462 (m) cm^{-1}. ^1H NMR (300 MHz, $CDCl_3$) δ 7.50−7.45 (m, 4H), 7.30−7.24 (m, 4H), 3.31 (s, 2H), 1.08 (s, 42H) ppm. ^{13}C NMR (75 MHz, $CDCl_3$) δ 132.4, 132.0, 128.5, 128.2, 125.8, 124.9, 117.8, 117.5, 103.9, 102.4, 96.8, 90.6, 82.0, 81.7, 18.6, 11.2 ppm. HRMS (ESI): *m/z* = 659.3491 [$(M + Na)^+$], calcd. for ($C_{44}H_{52}NaSi_2^+$): 659.3500.

Method 2:

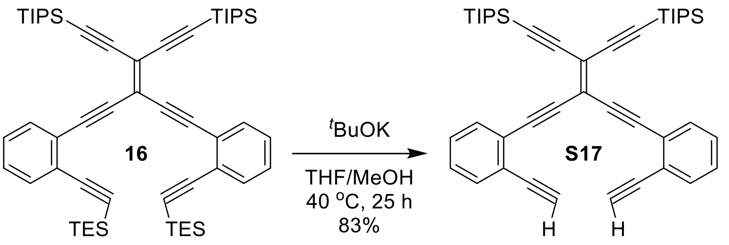

A solution of **16** (60 mg, 0.069 mmol) and *t*BuOK (78 mg, 0.69 mmol) in MeOH/THF (5:1, 6 mL) was heated to 40 °C until **16** was no longer visible using TLC analysis (25 h). The reaction mixture was allowed to cool to rt, Et_2O (30 mL), and saturated aq. NH_4Cl were added, the organic layer was separated, washed with saturated aq. NH_4Cl (2 x 30 mL), H_2O (2 x 30 mL), and brine (2 x 30 mL). The solution was dried ($MgSO_4$), filtered, and the solvent removed in *vacuo*. Column chromatography (silica, 5:1 hexanes/CH_2Cl_2) afforded **S17** (37 mg, 84%) as a brown oil. R_f = 0.43 (silica, hexanes/CH_2Cl_2 3:1).

Method 3:

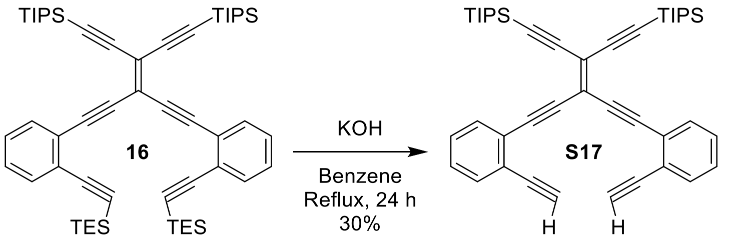

To a solution of **16** (605 mg, 0.803 mmol) in benzene (50 mL) was added KOH (452 mg, 8.06 mmol). The reaction was heated to reflux and stirred for 24 h, cooled to rt, and quenched via the addition of saturated aq. NH_4Cl (50 mL). The aq. phase was extracted with Et_2O (2 x 25 mL). The combined organic phase was washed with H_2O (15 mL) and saturated aq. NaCl (15 mL), dried with $MgSO_4$, filtered, and the solvent removed *in vacuo*. Column chromatography (silica gel, 5:1 hexanes/CH_2Cl_2) afforded **S17** (149 mg, 30%) as an amber oil.

Synthesis – molecules presented in Figure 3 (*in article*)

Mono-radiaannulene 4a

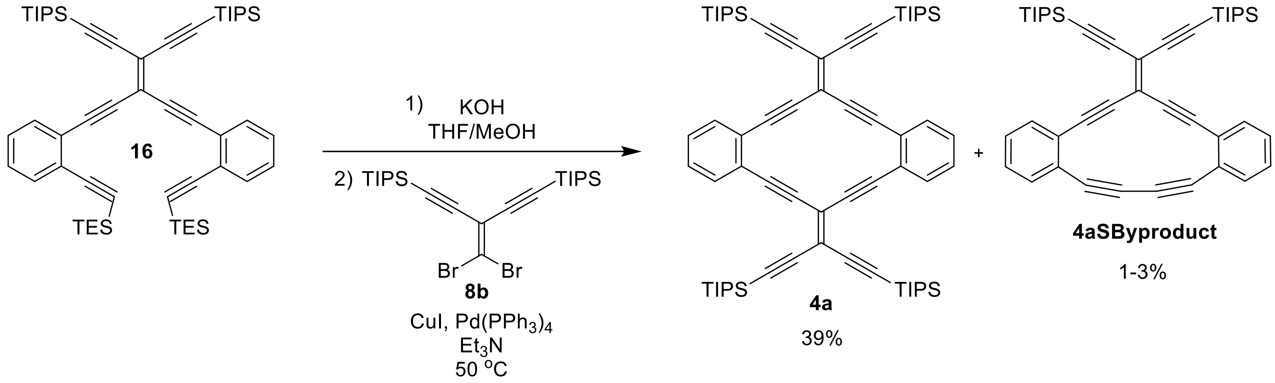

To a solution of **16** (100 mg, 0.113 mmol) in THF (2 mL) and MeOH (10 mL) was added KOH (65 mg, 1.2 mmol). The reaction was stirred at rt for 3 h and then quenched via the addition of saturated aq. NH_4Cl (20 mL). The aq. phase was extracted with Et_2O (2 x 20 mL). The combined organic phase was washed with H_2O (10 mL) and saturated aq. NaCl (10 mL), dried with $MgSO_4$, filtered, and the solvent reduced to ca. 5 mL *in vacuo*. This solution was transferred to a solution of **8b** (62 mg, 0.11 mmol) in deoxygenated, dry Et_3N (5 mL), and purged with argon for 15 min. To the reaction was added $Pd(PPh_3)_4$ (7 mg, 0.006 mmol) and CuI (3 mg, 0.02 mmol). The flask was sealed under argon with a rubber septum, heated to 50 °C, and stirred for 18 h. The solution was cooled to rt and the reaction quenched via the addition of saturated aq. NH_4Cl (25 mL). The aq. phase was extracted with Et_2O (2 x 25 mL). The combined organic phase was washed with H_2O (20 mL) and saturated aq. NaCl (20 mL), dried with $MgSO_4$, filtered, and the solvent removed *in vacuo*. Column chromatography (alumina, neutral, 4:1 to 2:1 hexanes/CH_2Cl_2) afforded a dark yellow solid. The solid was further purified by dissolving into a small amount of CH_2Cl_2 and precipitated by the addition of MeOH to yield **4a** (45 mg, 39%) as a bright yellow solid. R_f = 0.50 (4:1 hexanes/CH_2Cl_2). UV–Vis (THF): λ_{max} (ε / $M^{-1}cm^{-1}$) 282 (82700), 313 (28200), 330 (32300), 371 (22400), 426 (51800), 450 (65900) nm. IR (ATR): 2939 (m), 2890 (w), 2862 (m), 2134 (w), 1460 (m). ^1H NMR (300 MHz, CD_2Cl_2) δ 7.52–7.46 (m, 4H), 7.40–7.32 (m, 4H), 1.16 (s, 84H) ppm. ^{13}C NMR (75 MHz, CD_2Cl_2) δ 132.6, 129.2, 125.4, 118.0, 116.7, 104.5, 103.8, 97.1, 91.4, 18.8, 11.7 ppm. HRMS (MALDI, DCTB): *m/z* = 1020.6278 [$M^{•+}$], calcd. for ($C_{68}H_{92}Si_4^{•+}$): 1020.6271. DSC: decomposition, 254 °C (onset), 266 °C (peak).

Crystals suitable for X-ray crystallography by slow evaporation of solutions of **4a** in hexanes left standing in the dark at rt for several days. X-ray crystallographic data for **4a**: $C_{68}H_{92}Si_4$, M_r = 1021.78; crystal dimensions (mm) 0.3094 x 0.1924 x 0.1574; triclinic space group *P*-1 (no. 2); a = 8.4669(5) Å, b = 12.5501(7) Å, c = 15.4542(11) Å; α = 90.022(5)°, β = 96.448(6)°, γ = 97.093(5)°; V = 1619.12(17) $Å^3$; Z = 1; ρ_{calcd} = 1.048 mg mm^{-3}; μ = 1.115 mm^{-1}; λ = 1.54178 Å; T = –100 °C; $2\theta_{max}$ = 146.82°; total data collected = 9735; R_1 = 0.0503; wR_2 = 0.1407 for 337 variables, 6214 unique reflections, and 0 restraints; residual electron density = 0.714 and –0.621 e $Å^{-3}$.

The stepwise route to **4a** occasionally gave **4aSByproduct** in small amounts (1–3%), which could be isolated via column chromatography (alumina, neutral, 4:1 to 2:1 hexanes/CH_2Cl_2) as a bright yellow solid. R_f = 0.55 (4:1 hexanes/CH_2Cl_2). IR (ATR): 3064 (w), 2943 (s), 2892 (m), 2865 (s), 2211 (w), 2198 (w), 2175 (w), 2138 (w), 1469 (s). ^1H NMR (300 MHz, $CDCl_3$) δ 7.48–7.44 (m, 2H), 7.36–7.32 (m, 2H), 7.30–7.26 (m, 4H), 1.16–1.12 (m, 42H). ^{13}C NMR (75 MHz, $CDCl_3$) δ 131.3, 129.4, 129.1, 128.8, 128.7, 125.4, 118.1, 116.6, 104.3, 103.7, 97.2, 92.5, 87.0, 81.4, 18.7, 11.3. HRMS (MALDI, DCTB): *m/z* = 634.3447 [$M^{•+}$], calcd. for ($C_{44}H_{50}Si_2^{•+}$): 634.3446.

Mono-radiaannulene 4b

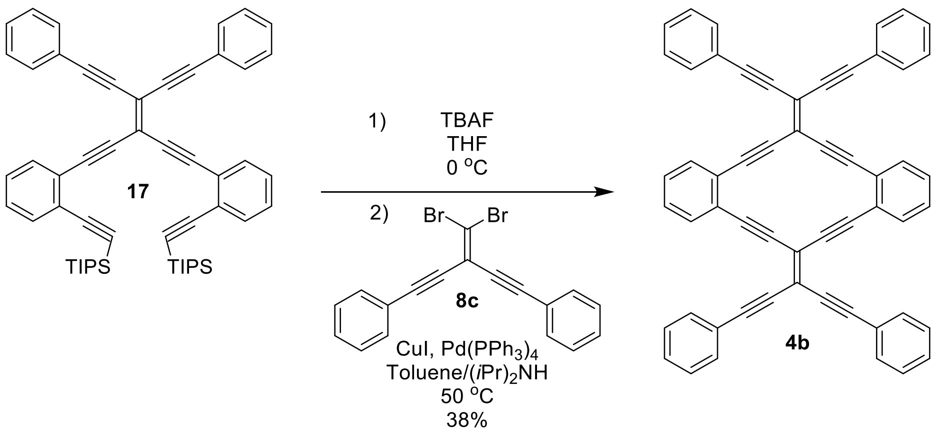

[Compound **8c** was synthesized according to literature procedure[6]]

A solution of **17** (0.10 g, 0.13 mmol) in THF (25 mL) was cooled to 0 °C and TBAF (1.0 M in THF, 0.28 mL, 0.28 mmol) was added. The reaction mixture was stirred until TLC analysis no longer indicated the presence of **17** (20 min). The solution was quenched via the addition of saturated aq. NH_4Cl (15 mL), and Et_2O (15 mL) was added. The organic layer was separated, washed with saturated aq. NH_4Cl (2 x 15 mL), H_2O (2 x 15 mL), and brine (2 x 15 mL). The solution was dried ($MgSO_4$), filtered, reduced to 2 mL, and added to a deoxygenated solution of **8c** (0.047 g, 0.12 mmol), $Pd(PPh_3)_4$ (7.3 mg, 6.4 μmol), and CuI (2.4 mg, 13 μmol) in dry (iPr)$_2$NH/dry toluene (1:1, 14 mL). The solution was stirred at 50 °C until TLC analysis no longer showed the presence of the desilylated derivative of **17** (25 h). The reaction mixture was allowed to cool to rt, CH_2Cl_2 (20 mL), and saturated aq. NH_4Cl (20 mL) were added. The organic layer was separated and washed with saturated aq. NH_4Cl (2 x 25 mL), H2O (2 x 25 mL), and brine (2 x 25 mL). The solution was dried ($MgSO_4$), filtered, and the solvent removed *in vacuo*. Column chromatography (alumina, hexanes/CH_2Cl_2 2:1), followed by recrystallization hexanes/CH_2Cl_2 (1:1) gave **4b** (34 mg, 38%) as an orange solid. R_f = 0.58 (alumina, hexanes/CH_2Cl_2 2:1). M.p. 214−217 °C. UV–Vis (THF): λ_{max} (ε / $M^{-1}cm^{-1}$) 255 (31800), 288 (90500), 306 (35100), 331 (23900), 345 (24300), 460 (sh, 41300), 483 (52600) nm. IR (ATR) 3056 (w), 2922 (m), 2852 (w), 2188 (m), 1714 (m), 1671 (m), 1594 (m), 1485 (s), 1437 (s) cm^{-1}. ^{1}H NMR (400 MHz, $C_2D_2Cl_4$) δ 7.60−7.57 (m, 8H), 7.52−7.50 (m, 4H), 7.38−7.36 (m, 12H), 7.31−7.29 (m, 4H) ppm. ^{13}C (100 MHz, CD_2Cl_2) δ 132.5, 132.1, 129.9, 129.6, 129.1, 125.4, 122.7, 97.8, 91.9, 87.8 ppm (three signals coincident or not observed). HRMS (APPI): m/z = 700.2186 [M^+], calcd. for ($C_{56}H_{28}$): 701.2191.

Single crystals suitable for X-ray crystallographic analysis were grown by slow evaporation of a solution of **4b** from a solution of CH_2Cl overlayed with an excess of hexanes at rt. X-ray crystallographic data for **4b**: $C_{56}H_{28}$, M_r = 700.78; crystal dimensions(mm) 0.203 x 0.1111 x 0.0232; monoclinic crystal system; space group $P2_1/n$; a = 15.9374(9) Å, b = 5.8425(3) Å, c = 20.8169(11) Å; β = 98.404(5)°; V = 1917.52(18) Å^3; Z = 2; ρ_{calcd} = 1.214 mg mm^{-3}; μ = 0.527 mm^{-1}; T = 173.00(14) K; $2\theta_{max}$ = 122.84°; total data collected = 4922; R_1 = 0.0845; wR_2 = 0.2574 for 2874 data, 253 variables and 0 restraints; largest difference, peak and hole = 0.30 and −0.20 e Å^{-3}.

Compound **5**

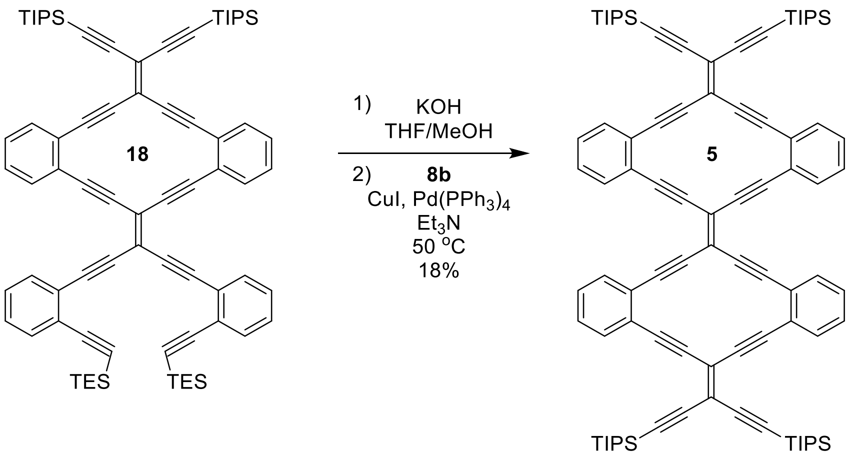

To a solution of **18** (58 mg, 0.051 mmol) in THF (4 mL) and MeOH (10 mL) was added KOH (30 mg, 0.53 mmol). The reaction was stirred at rt for 4 h and quenched via the addition of saturated aq. NH_4Cl (20 mL). The aq. phase was extracted with Et_2O (2 x 20 mL). The combined organic phase was washed with H_2O (10 mL) and saturated aq. NaCl (10 mL), dried with $MgSO_4$, filtered, and the solvent reduced to ca. 5 mL *in vacuo*. This solution was transferred to a solution of **8b** (28 mg, 0.051 mmol) in deoxygenated dry Et_3N (5 mL), and purged with argon for 15 min. To the reaction was added $Pd(PPh_3)_4$ (3 mg, 0.003 mmol) and CuI (1 mg, 0.005 mmol). The flask was sealed under argon with a rubber septum, heated to 50 °C, and stirred for 16 h. The solution was cooled to rt and the reaction quenched via the addition of saturated aq. NH_4Cl (25 mL). The aq. phase was extracted with Et_2O (2 x 25 mL). The combined organic phase was washed with H_2O (20 mL) and saturated aq. NaCl (20 mL), dried with $MgSO_4$, filtered, and the solvent removed *in vacuo*. Column chromatography (alumina, neutral, 4:1 to 2:1 hexanes/CH_2Cl_2) afforded **5** (12 mg, 18%) as a deep red solid. R_f = 0.44 (2:1 hexanes/CH_2Cl_2). Mp = 200 °C (decomp). UV–Vis (THF): λ_{max} 287, 343, 415, 485, 504 nm. IR ($CHCl_3$ cast film): 3058 (w), 2943 (s), 2892 (m), 2865 (s), 2185 (w), 2136 (w), 1485 (m), 1463 (m). ^1H NMR (400 MHz, CD_2Cl_2) δ 7.71−7.69 (m, 4H), 7.61−7.59 (m, 4H), 7.48−7.38 (m, 8H), 1.19−1.16 (m, 84H); ^{13}C NMR (100 MHz, CD_2Cl_2) δ 132.8, 132.3, 129.5, 129.4, 125.5, 125.4, 118.3, 117.2, 116.6, 104.6, 104.0, 98.4, 97.1, 92.1, 91.6, 18.9, 11.7. ^{13}C NMR (100 MHz, $CDCl_3$) δ 132.3, 131.8, 128.73, 128.67, 128.5, 125.5, 125.4, 117.8, 117.0, 116.8, 104.4, 103.0, 97.9, 96.8, 92.0, 91.5, 18.7, 11.4 ppm. HRMS (ESI): *m/z* = 1315.6759 [$(M + Na)^+$], calcd. for ($C_{90}H_{100}NaSi_4^+$): 1315.6794. DSC: decomposition, 280 °C (onset), 285 °C (peak).

Crystals suitable for X-ray crystallography by slow evaporation of solutions of **5** in hexanes left standing in the dark at rt for several days. X-ray crystallographic data for **5**: $C_{90}H_{100}Si_4$, M_r = 1294.06; crystal dimensions (mm) 0.373 x 0.1684 x 0.0756; triclinic space group *P*-1 (No. 2); a = 8.9732(7) Å, b = 13.1109(9) Å, c = 18.0827(12) Å; α = 71.321(6)°, β = 80.336(6)°, γ = 79.647(6)°; V = 1968.6(2) Å^3; Z = 1; ρ_{calcd} = 1.092 mg mm^{-3}; μ = 1.018 mm^{-1}; λ = 1.54178 Å; T = –100 °C; $2\theta_{max}$ = 147.34°; total data collected = 11687; R_1 = 0.0493; wR_2 = 0.1461 for 436 variables, 7538 unique reflections, and 0 restraints; residual electron density = 0.56 and –0.28 e Å^{-3}.

Tri-radiaannulene 6

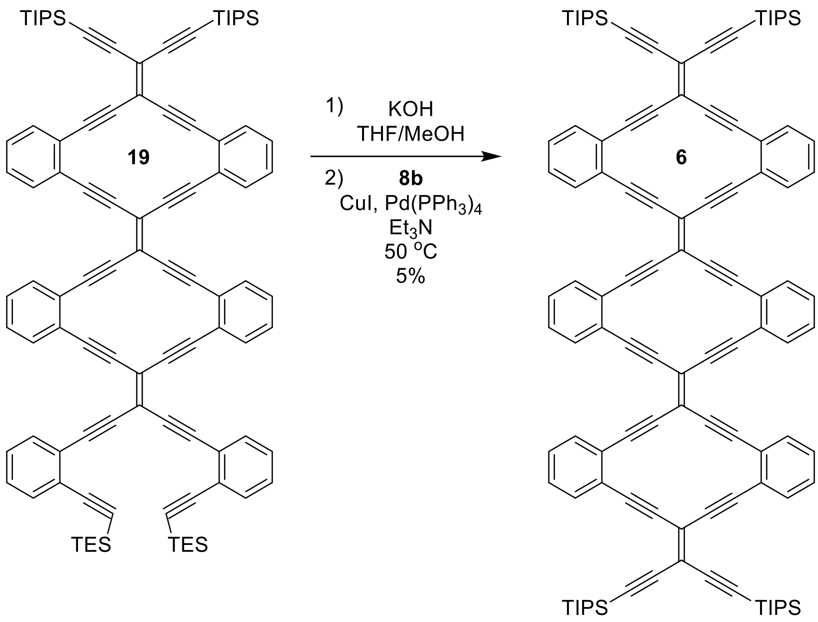

To a solution of **19** (65 mg, 0.046 mmol) in THF (5 mL) and MeOH (8 mL) was added KOH (30 mg, 0.53 mmol). The reaction mixture was stirred at rt for 4 h, and then quenched via the addition of saturated aq. NH_4Cl (25 mL). The aq. phase was extracted with CH_2Cl_2 (2 x 25 mL). The combined organic phase was washed with H_2O (20 mL) and saturated aq. NaCl (20 mL), dried with $MgSO_4$, filtered, and the solvent reduced to ca. 5 mL *in vacuo*. The solution was then transferred to a flask charged with **8b** (25 mg, 0.046 mmol) dissolved in Et_3N (5 mL) and the combined solution purged with argon for 15 min. To this solution was added $Pd(PPh_3)_4$ (3 mg, 0.003 mmol) and CuI (1 mg, 0.005 mmol). The flask was sealed under argon with a rubber septum, heated to 50 °C, and stirred for 16 h. The solution was cooled to rt and the reaction quenched via the addition of saturated aq. NH_4Cl (25 mL). The aq. phase was extracted with CH_2Cl_2 (2 x 25 mL). The combined organic phase was washed with H_2O (20 mL) and saturated aq. NaCl (20 mL), dried with $MgSO_4$, filtered, and the solvent removed *in vacuo*. Preparative TLC (silica gel, 3:2 hexanes/CH_2Cl_2) afforded **6** (3.5 mg, 5%) as a dark red solid, which decomposed when dissolved in $CDCl_3$. R_f = 0.4 (3:2 hexanes/CH_2Cl_2). UV–Vis (THF): λ_{max} 284, 490, 522 nm. MALDI HRMS (DCTB) *m/z* calcd for $C_{112}H_{108}Si_4$ [M^+] 1564.7523, found 1564.7532

The structural assignment of **6** rests on high-resolution mass spectrometry and UV-Vis spectroscopy from a small amount (mgs) of sample purified by preparative thin-layer chromatography, and the limited amount of sample precluded further characterization. The majority of the product isolated from the reaction mixture, calculated as 5% yield (3.5 mg, vide infra) based on the assumption of a pure compound, decomposed upon exposure to $CDCl_3$ during sample preparation for NMR analyses. A subsequent synthesis of **6** is not feasible based on resources available.

Compound **15a**

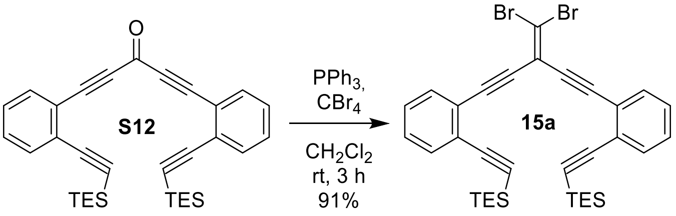

To a solution of PPh_3 (7.130 g, 27.18 mmol) and CBr_4 (4.468 g, 13.47 mmol) in dry, deoxygenated CH_2Cl_2 (90 mL) was added a solution of **S12** (3.226 g, 6.365 mmol) in dry, deoxygenated CH_2Cl_2 (10 mL).

The solution was stirred for 3 h at rt. The reaction was quenched via the addition of hexanes, followed by a plug through silica gel with hexanes. Solvent removal in *vacuo* afforded **15a** (3.849 g, 91%) as a yellow oil. R_f = 0.60 (2:1 hexanes/CH_2Cl_2). IR (CH_2Cl_2 cast film): 3061 (w), 2955 (s), 2934 (s), 2910 (s), 2874 (s), 2204 (w), 2158 (m), 1480 (m) ^{1}H NMR (400 MHz, $CDCl_3$) δ 7.50–7.47 (m, 4H), 7.29–7.23 (m, 4H), 0.99 (t, J = 7.9 Hz, 18H), 0.63 (q, J = 7.9 Hz, 12H) ppm. ^{13}C NMR (75 MHz, $CDCl_3$) δ 132.7, 132.2, 128.6, 128.0, 125.8, 124.9, 114.4, 108.7, 104.0, 97.0, 94.3, 89.2, 7.5, 4.4 ppm. HRMS (MALDI, DCTB): m/z = 662.0857 [$M^{\bullet+}$], calcd. for ($C_{34}H_{38}{}^{79}Br^{81}BrSi_2{}^{\bullet+}$): 662.0853.

Compound **15b**

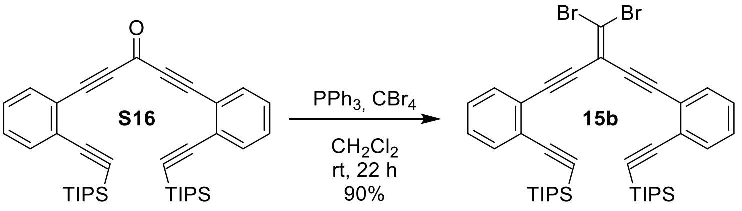

A solution of CBr_4 (5.0 g, 15 mmol) and PPh_3 (8.1 g, 31 mmol) in dry CH_2Cl_2 (120 mL) was stirred for 45 min and **S16** (4.2 g, 7.2 mmol) was added. The reaction mixture was stirred until TLC analysis no longer indicated the presence of **S16** (22 h) and then filtered through a plug of silica (hexanes). Column chromatography (silica, hexanes/CH_2Cl_2 10:1) afforded **15b** (4.86 g, 90%) as a dark orange oil. R_f = 0.58 (silica, hexanes). IR (ATR) 3059 (w), 2943 (s), 2890 (m), 2858 (s), 2202 (w), 2151 (m), 1469 (s), 1441 (m) $cm^{-1.}$ ^{1}H NMR (300 MHz, $CDCl_3$) δ 7.51−7.48 (m, 4H), 7.31−7.24 (m, 4H), 1.10 (s, 42H) ppm. ^{13}C NMR (75 MHz, $CDCl_3$) δ 132.8, 132.2, 128.6, 127.9, 125.9, 124.9, 114.5, 108.7, 104.7, 95.9, 94.3, 89.0, 18.7, 11.3 ppm. HRMS (APPI): m/z = 744.1790 [$M^{\bullet+}$], calcd for ($C_{40}H_{50}{}^{79}Br_2Si_2{}^{\bullet+}$): 744.1812.

Compound **16a**

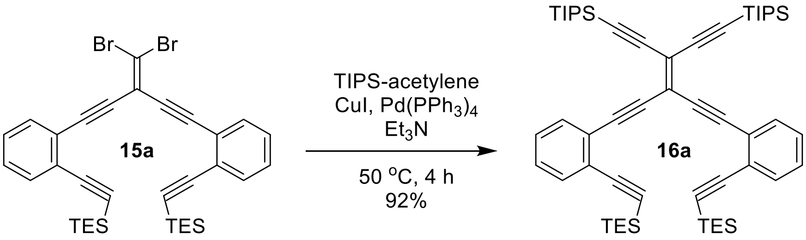

To a solution of **15a** (1.244 g, 1.877 mmol) in deoxygenated, dry Et_3N (10 mL) was added $Pd(PPh_3)_4$ (0.125 g, 0.108 mmol) and CuI (0.039 g, 0.205 mmol). The solution was purged with argon for an additional 10 min after which triisopropylsilylacetylene (2.2 mL, 1.8 g, 9.8 mmol) was added. The flask was sealed under argon with a rubber septum and stirred for 4 h at 50 °C. The solution was cooled to rt and the reaction was quenched via the addition of saturated aq. NH_4Cl (25 mL). The aq. phase was extracted with Et_2O (2 x 25 mL). The combined organic phase was washed with H_2O (20 mL) and saturated aq. NaCl (20 mL), dried with $MgSO_4$, filtered, and the solvent removed *in vacuo*. Column chromatography (silica gel, 5:1 hexanes/CH_2Cl_2) afforded **16a** (1.493 g, 92%) as a dark amber oil. R_f = 0.60 (5:1 hexanes/CH_2Cl_2). UV–Vis (THF): λ_{max} 238, 322, 362, 372, 390 nm. IR (CH_2Cl_2 cast film): 2956 (s), 2866 (s), 2159 (w), 1463 (m) ^{1}H NMR (400 MHz, $CDCl_3$) δ 7.45–7.41 (m, 4H), 7.24–7.19 (m, 4H), 1.04 (s, 42H), 0.93 (t, J = 7.9 Hz, 18H), 0.58 (q, J = 7.9 Hz, 12H) ppm. ^{13}C NMR (75 MHz, $CDCl_3$) δ 132.2, 132.1, 128.2, 127.5, 126.1, 125.7, 118.3, 117.9, 104.08, 103.99, 101.9, 97.08, 96.97, 90.2, 18.6, 11.3, 7.5, 4.4 ppm. HRMS (MALDI, DCTB): m/z = 864.5335 [$M^{\bullet+}$], calcd. for ($C_{56}H_{80}Si_4{}^{\bullet+}$): 864.5331.

Compound **16b**

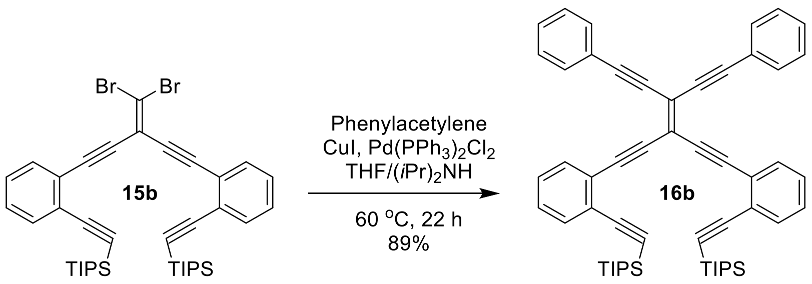

To a deoxygenated solution of **15b** (0.13 g, 0.17 mmol), $Pd(PPh_3)_2Cl_2$ (6.1 mg, 8.7 μmol), CuI (3.3 mg, 0.017 mmol), and dry $(i\text{Pr})_2NH$ (3 mL) in dry THF (8 mL) phenylacetylene (0.04 mL, 0.04 g, 0.4 mmol) was added. The reaction mixture was heated at 60 °C until TLC analysis no longer showed the presence of **15b** (22 h). The reaction mixture was allowed to cool to rt, Et_2O (20 mL), and saturated aq. NH_4Cl (20 mL) were added. The organic layer was separated and washed with saturated aq. NH_4Cl (2 x 25 mL), H_2O (2 x 25 mL), and brine (2 x 25 mL). The solution was dried ($MgSO_4$), filtered, and the solvent removed *in vacuo*. Purification via column chromatography (silica, hexanes/$CH_2Cl_2$10:1 to 4:1) afforded **16b** (0.119 g, 89%) as a brown oil. R_f = 0.42 (silica, hexanes/CH_2Cl_2 3:1). UV–Vis (THF): λ_{max} (ε / $M^{-1}cm^{-1}$) 238 (68900), 252 (47900), 303 (32000), 324 (20500), 405 (31900) nm. IR (CH_2Cl_2 cast film) 3056 (w), 2941 (m), 2890 (m), 2863 (m), 2185 (w), 1479 (m), 1462 (m), 1441 (m) cm^{-1}. ^1H NMR (300 MHz, CD_2Cl_2) δ 7.57−7.52 (m, 8H), 7.37−7.28 (m, 10H), 1.05 (s, 42H) ppm. ^{13}C NMR (75 MHz, CD_2Cl_2) δ 133.1, 132.6, 132.2, 129.5, 129.0, 128.8, 128.4, 126.3, 125.5, 122.8, 118.5, 117.7, 105.0, 99.5, 97.9, 96.5, 90.5, 87.6, 18.8, 11.7 ppm. HRMS (APPI): *m/z* = 788.4227 [$M^{\bullet+}$], calcd. for ($C_{56}H_{60}Si_2^{\bullet+}$): 788.4228.

Compound **17**

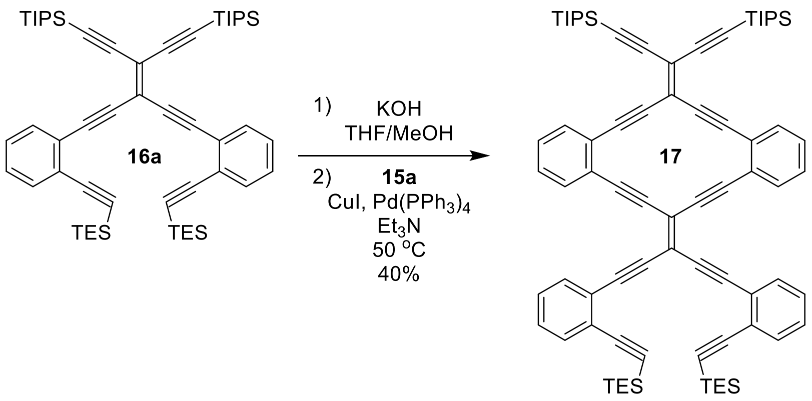

To a solution of **16a** (754 mg, 0.871 mmol) in THF (4 mL) and MeOH (15 mL) was added KOH (490 mg, 8.73 mmol). The reaction was stirred at rt for 6 h, and then quenched via the addition of saturated aq. NH_4Cl (25 mL). The aq. phase was extracted with Et_2O (2 x 25 mL). The combined organic phase was washed with H_2O (20 mL) and saturated aq. NaCl (20 mL), dried with $MgSO_4$, filtered, and the solvent reduced to ca. 5 mL *in vacuo*. The solution was then transferred to a flask charged with **15a** (574 mg, 0.866 mmol) dissolved in Et_3N (12 mL), and purged with argon for 15 min. To this solution was added $Pd(PPh_3)_4$ (53 mg, 0.046 mmol) and CuI (16 mg, 0.084 mmol). The flask was sealed under argon with a rubber septum, heated to 50 °C, and stirred for 16 h. The solution was cooled to rt and the reaction quenched via the addition of saturated aq. NH_4Cl (25 mL). The aq. phase was extracted with Et_2O (2 x 25 mL). The combined organic phase was washed with H_2O (20 mL) and saturated aq. NaCl (20 mL), dried with $MgSO_4$, filtered, and the solvent removed *in vacuo*. Column chromatography (silica gel, 3:1 hexanes/CH_2Cl_2) afforded **17** (391 mg, 40%) as a bright yellow solid. R_f = 0.33 (2:1 hexanes/CH_2Cl_2). UV–Vis (THF): λ_{max} (ε / $M^{-1}cm^{-1}$) 286 (87100), 328 (37800), 338 (35200), 388 (24800), 450 (52300), 470 (59300) nm. IR ($CHCl_3$ cast film): 3060

(w), 2955 (s), 2887 (s), 2867 (s), 2188 (w), 2157 (w), 1481 (m), 1463 (m). ^{1}H NMR (400 MHz, CD_2Cl_2) δ 7.64−7.62 (m, 2H), 7.57−7.52 (m, 6H), 7.38−7.30 (m, 8H), 1.17−1.16 (m, 42H), 0.95 (t, J = 7.9 Hz, 18H), 0.57 (q, J = 7.9 Hz, 12H) ppm. ^{13}C NMR (100 MHz, $CDCl_3$) δ 132.6, 132.1, 132.0, 128.6, 128.5, 128.4, 127.9, 126.0, 125.54, 125.50, 125.3, 118.1, 117.6, 117.2, 116.9, 104.5, 103.8, 102.8, 97.9, 97.45, 97.36, 96.9, 91.9, 91.4, 90.6, 18.7, 11.4, 7.5, 4.3 (one signal coincident or not observed) ppm. HRMS (MALDI, DCTB): m/z = 1136.5957 [$M^{\bullet+}$], calcd. for ($C_{78}H_{88}Si_4^{\bullet+}$): 1136.5958.

Compound **18**

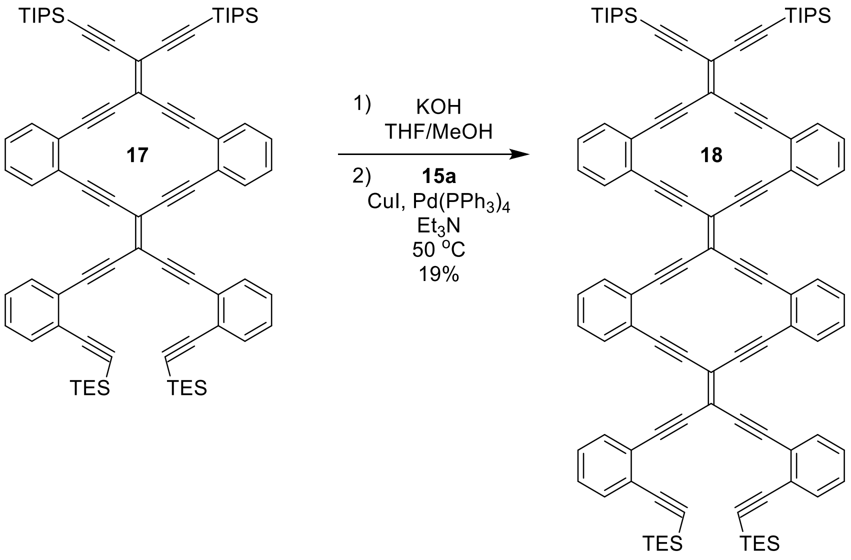

To a solution of **17** (391 mg, 0.344 mmol) in THF (7 mL) and MeOH (15 mL) was added KOH (195 mg, 3.46 mmol). The reaction was stirred at rt for 4 h, and then quenched via the addition of saturated aq. NH_4Cl (25 mL). The aq. phase was extracted with Et_2O (2 x 25 mL). The combined organic phase was washed with H_2O (20 mL) and saturated aq. NaCl (20 mL), dried with $MgSO_4$, filtered, and the solvent reduced to ca. 5 mL *in vacuo*. The solution was then transferred to a flask charged with **15a** (223 mg, 0.336 mmol) dissolved in deoxygenated, dry Et_3N (6 mL), and purged with argon for 15 min. To this solution was added $Pd(PPh_3)_4$ (20 mg, 0.017 mmol) and CuI (8 mg, 0.040 mmol). The flask was sealed with a rubber septum under argon, heated to 50 °C, and stirred for 16 h. The solution was cooled to rt and the reaction quenched via the addition of saturated aq. NH_4Cl (25 mL). The aq. phase was extracted with Et_2O (2 x 25 mL). The combined organic phase was washed with H_2O (20 mL) and saturated aq. NaCl (20 mL), dried with $MgSO_4$, filtered, and the solvent removed *in vacuo*. Column chromatography (alumina, neutral, 4:1 to 2:1 hexanes/CH_2Cl_2) afforded **18** (88 mg, 19%) as a dark red solid. R_f = 0.50 (1:1 hexanes/CH_2Cl_2). UV−Vis (THF): λ_{max} 288, 388, 494, 516 nm. IR ($CHCl_3$ cast film): 3061 (w), 2954 (s), 2889 (s), 2867 (s), 2184 (w), 2157 (w), 2138 (w), 1484 (m), 1462 (m); ^{1}H NMR (400 MHz, CD_2Cl_2) δ 7.71 (dd, J = 7.6, 2.3 Hz, 4H), 7.66−7.64 (m, 2H), 7.60−7.55 (m, 6H), 7.48−7.34 (m, 12H), 1.19−1.17 (m, 42H), 0.96 (t, J = 7.9 Hz, 18H), 0.58 (q, J = 7.9 Hz, 12H); ^{13}C NMR (100 MHz, CD_2Cl_2) δ 133.1, 133.0, 132.7, 132.5, 132.4, 132.3, 129.50, 129.47, 129.3, 129.2, 128.6, 126.4, 125.62, 125.58, 125.57, 125.52, 125.46, 118.3, 118.1, 117.7, 117.40, 117.37, 116.8, 104.7, 104.2, 103.8, 98.6, 98.5, 98.4, 98.1, 97.8, 97.3, 92.4, 92.3, 92.2, 91.6, 90.9, 18.9, 11.8, 7.7, 4.6 (one signal coincident or not observed). HRMS (MALDI, DCTB): m/z = 1408.6596 [$M^{\bullet+}$], calcd. for ($C_{100}H_{96}Si_4^{\bullet+}$): 1408.6584.

Synthesis of starting material building blocks and mono- and di-radiaannulenes via one-pot approaches and other methods

Synthesis of mono-radiaannulenes *(One-pot syntheses)*
Mono-radiaannulene 4a

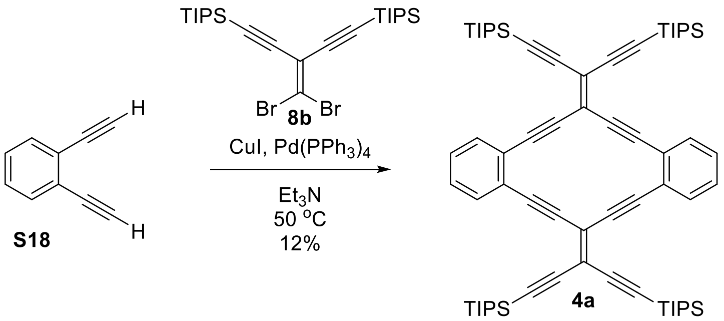

To a solution of 1,2-diethynylbenzene **S18** (171 mg, 1.36 mmol) and **8b** (736 mg, 1.35 mmol) in deoxygenated, dry Et_3N (10 mL) was added $Pd(PPh_3)_4$ (158 mg, 0.137 mmol) and CuI (52 mg, 0.273 mmol). The flask was sealed under argon with a rubber septum, heated to 50 °C, and stirred for 24 h. The solution was cooled to rt and the reaction quenched via the addition of saturated aq. NH_4Cl (30 mL). The aq. phase was extracted with Et_2O (2 x 25 mL). The combined organic phase was washed with H_2O (20 mL) and saturated aq. NaCl (20 mL), dried with $MgSO_4$, filtered, and the solvent removed *in vacuo*. Column chromatography (alumina, neutral, 4:1 to 2:1 hexanes/CH_2Cl_2) afforded a dark yellow solid. The solid was further purified by dissolving into a small amount of CH_2Cl_2 and precipitated by the addition of MeOH to yield **4a** (83 mg, 12%) as a bright yellow solid.

Synthesis of building block S22

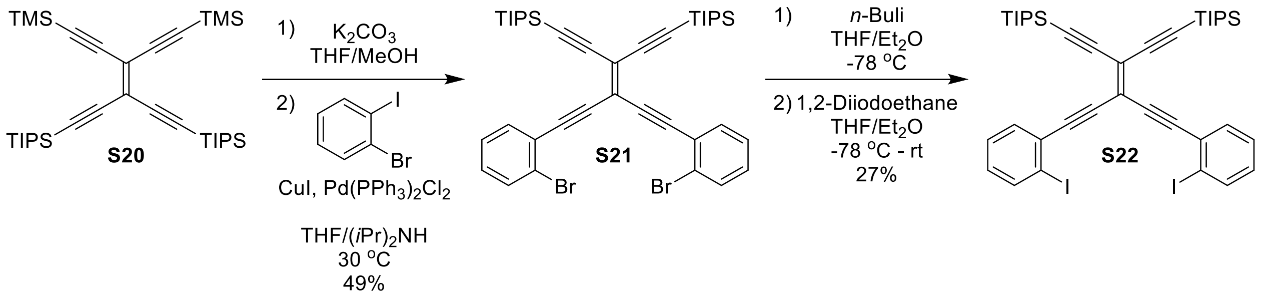

Compound **S21**

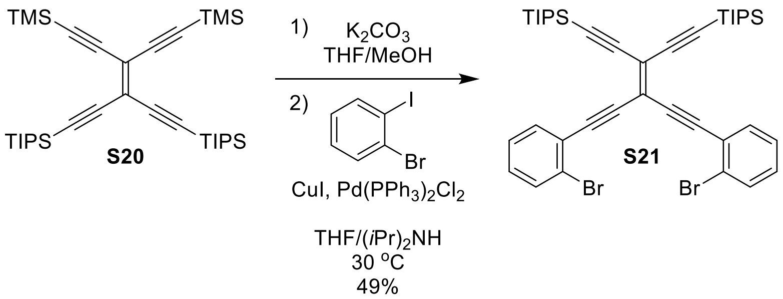

[Compound **S20** was synthesized according to literature procedure[7]]

A solution of **S20** (0.40 g, 0.69 mmol) and K_2CO_3 (0.19 g, 1.4 mmol) in MeOH/THF (4:1, 25 mL) was stirred until TLC analysis no longer showed the presence of **S20** (1 h). The solution was quenched via the addition of saturated aq. NH_4Cl (15 mL), and Et_2O (15 mL) was added. The organic layer was separated, washed with saturated aq. NH_4Cl (2 x 15 mL), H_2O (2 x 15 mL), and brine (2 x 15 mL). The solution was

dried ($MgSO_4$), filtered, reduced to 2 mL, and added to a deoxygenated solution of 2-bromoiodobenzene (0.41 g, 0.18 mL, 1.4 mmol), CuI (13 mg, 0.069 mmol), $Pd(PPh_3)_2Cl_2$ (24 mg, 0.034 mmol), and dry (iPr)$_2$NH (5 mL) in dry THF (15 mL). The reaction mixture was stirred at 30 °C until TLC analysis no longer showed the presence of the desilylated version of **S20** (3 d). To the reaction mixture Et_2O (20 mL) and saturated aq. NH_4Cl (20 mL) were added, the organic layer was separated and washed with saturated aq. NH_4Cl (2 x 25 mL), H_2O (2 x 25 mL), and brine (2 x 25 mL). The solution was dried with $MgSO_4$, filtered, and the solvent removed *in vacuo*. After purification via column chromatography (1. silica, hexanes/CH_2Cl_2 10:1, 2. alumina, cyclohexane) **S21** (0.252 g, 49%). was obtained as a yellow solid. Mp 84−86 °C. R_f = 0.59 (silica, hexanes/CH_2Cl_2 2:1). IR (ATR) 2938 (m), 2923 (m), 2860 (m), 2193 (w), 2144 (w), 1460 (m) cm^{-1}. ^{1}H NMR (300 MHz, $CDCl_3$) δ 7.57 (dd, J = 8.0, 1.0 Hz, 2H), 7.48 (dd, J = 7.7, 1.7 Hz, 2H), 7.27−7.13 (m, 4H), 1.07−1.06 (m, 42H) ppm. ^{13}C NMR (75 MHz, $CDCl_3$) δ 133.5, 132.4, 130.0, 126.8, 125.9, 124.9, 117.9, 116.9, 103.8, 102.8, 96.9, 90.8, 18.6, 11.2 ppm. HRMS (APPI): m/z = 744.1826 [$M^{\bullet+}$], calcd. for ($C_{40}H_{50}{}^{79}Br_2Si_2{}^{\bullet+}$): 744.1812.

Compound **S22**

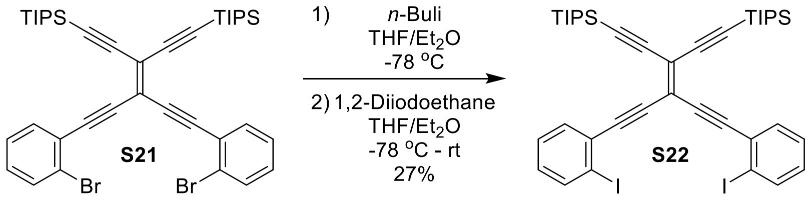

A solution of **S21** (0.33 g. 0.44 mmol) in dry THF/dry Et_2O (1:1, 20 mL) was cooled to −78 °C and nBuLi (2.5 M in hexanes, 0.72 mL, 1.8 mmol) was added. The reaction mixture was stirred for 90 min at −78 °C and 1,2-diiodoethane (0.37 g, 1.3 mmol) was added. The reaction mixture was allowed to warm to rt, and stirred for 18 h. To the reaction mixture CH_2Cl_2 (40 mL) was added, the organic layer was washed with H_2O (3 x 30 mL), dried with Na_2SO_4, filtered, and reduced *in vacuo*. Column chromatography (silica, hexanes) yielded **S22** (100 mg, 27%) as yellow orange oil. R_f = 0.45 (silica, hexanes). UV–Vis ($CHCl_3$): λ_{max} (ε / M^{-1}cm^{-1}) 295 (24900), 369 (37700), 384 (38600) nm. IR (ATR) 3061 (vw), 2940 (m), 2890 (m), 2862 (m), 2203 (w), 2138 (w), 1460 (m) cm^{-1}. ^{1}H NMR (400 MHz, CD_2Cl_2) δ 7.89 (dd, J = 8.0, 0.9 Hz, 2H), 7.51 (dd, J = 7.8, 1.6 Hz, 2H), 7.34 (dt, J = 7.6, 1.2 Hz, 2H), 7.06 (dt, J = 7.7, 1.5 Hz, 2H), 1.12−1.08 (m, 42H) ppm. ^{13}C NMR (75 MHz, $CDCl_3$) δ 138.8, 133.0, 129.9, 129.3, 127.5, 117.8, 117.0, 103.9, 102.8, 101.0, 100.4, 89.9, 18.6, 11.3 ppm. HRMS (APPI): m/z = 840.1525 [$M^{\bullet+}$], calcd. for ($C_{40}H_50I_2Si_2{}^{\bullet+}$) 840.1540.

Mono-radiaannulene 4a

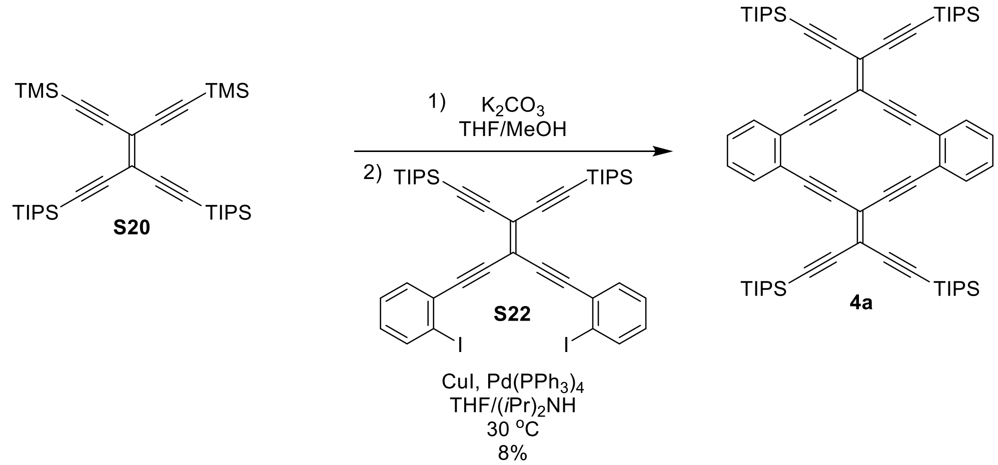

A solution of **S20** (0.10 g, 0.17 mmol) and K_2CO_3 (51 mg, 0.37 mmol) in MeOH/THF (4:1, 25 mL) was stirred until TLC analysis no longer indicated the presence of **S20** (2 h). The solution was quenched via the addition of saturated aq. NH_4Cl (15 mL), and Et_2O (15 mL) was added. The organic layer was separated,

washed with saturated aq. NH_4Cl (2 x 15 mL), H_2O (2 x 15 mL), and brine (2 x 15 mL). The solution was dried with $MgSO_4$, filtered, reduced to 1 mL, and added to a deoxygenated solution of **S20** (0.13 g, 0.16 mmol), $Pd(PPh_3)_4$ (9.2 mg, 8.0 µmol), CuI (3.0 mg, 16 µmol), and dry (iPr)$_2$NH (3 mL) in dry THF (10 mL). The solution was heated at 30 °C until TLC analysis no longer showed the presence of the desilylated species of **S20** (3 d). The reaction mixture was allowed to cool to rt, Et_2O (20 mL), and saturated aq. NH_4Cl (20 mL) were added. The organic layer was separated and washed with saturated aq. NH_4Cl (2 x 25 mL), H_2O (2 x 25 mL), and brine (2 x 25 mL). The solution was dried ($MgSO_4$), filtered, and the solvent removed *in vacuo*. Column chromatography (alumina, hexanes/CH_2Cl_2 4:1 to 2:1), and recrystallization from CH_2Cl_2/MeOH provided **4a** (13 mg, 8%) as a yellow-brown solid.

Synthesis of di-radiaannulene 5 (*One-pot synthesis*)

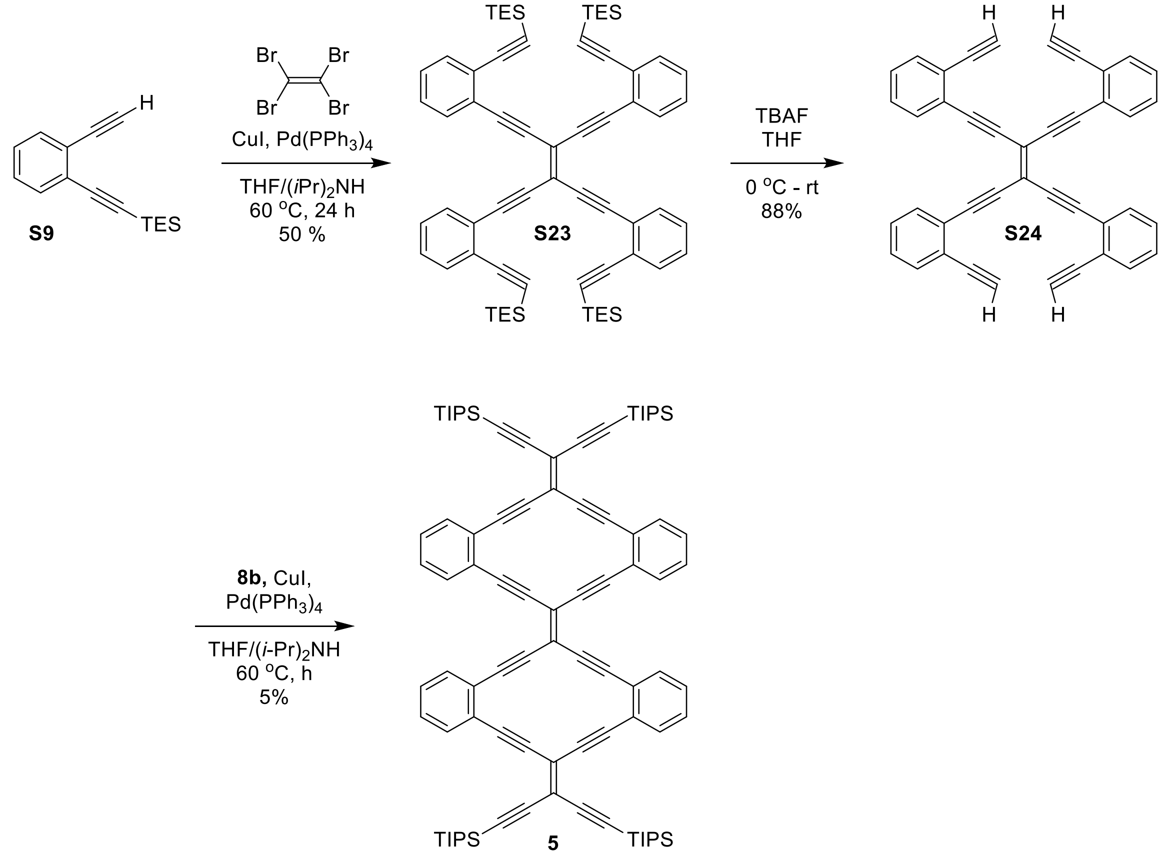

Compound **S23**

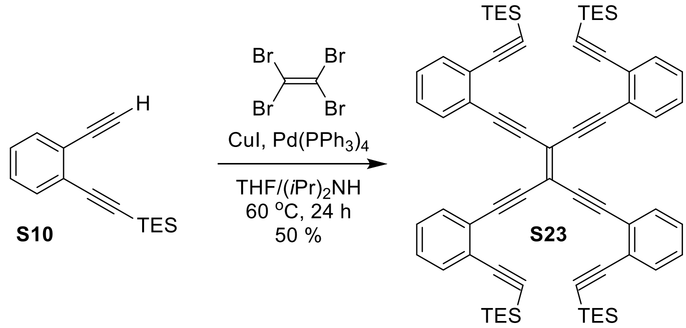

To a solution of **S10** (126 mg, 0.524 mmol) and tetrabromoethene (40 mg, 0.12 mmol) in THF (2 mL) and (iPr)$_2$NH (1 mL) was added $Pd(PPh_3)_4$ (10 mg, 0.008 mmol) and CuI (1 mg, 0.005 mmol). The flask was sealed under argon with a rubber septum, heated to 60 °C, and stirred for 24 h. The solution was cooled to rt and the reaction quenched via the addition of saturated aq. NH_4Cl (10 mL). The aq. phase was extracted with

Et_2O (2 x 15 mL). The combined organic phase was washed with H_2O (10 mL) and saturated aq. NaCl (10 mL), dried with $MgSO_4$, filtered, and the solvent removed in *vacuo*. Column chromatography (silica gel, 5:1 hexanes/CH_2Cl_2) afforded **S23** (56 mg, 50%) as a bright yellow/orange oily solid. R_f = 0.53 (2:1 hexanes/CH_2Cl_2). IR (ATR): 2951 (s), 2908 (m), 2871 (s), 2193 (w), 2154 (w), 1476 (m). ^{1}H NMR (400 MHz, $CDCl_3$) δ 7.49–7.45 (m, 8H), 7.25–7.16 (m, 8H), 0.93 (t, J = 7.9 Hz, 36H), 0.56 (q, J = 7.9 Hz, 24H) ppm. ^{13}C NMR (75 MHz, $CDCl_3$) δ 132.6, 132.4, 128.3, 127.8, 125.8, 125.6, 118.3, 103.9, 97.6, 97.0, 90.7, 7.5, 4.3 ppm. HRMS (ESI): m/z = 1003.4893 [$(M + Na)^+$], calcd. for ($C_{66}H_{76}NaSi_4^+$): 1003.4916.

Compound **S24**

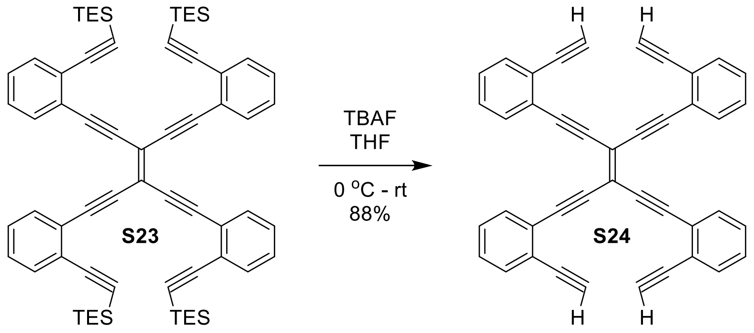

To a solution of **S23** (104 mg, 0.106 mmol) THF (10 mL) was TBAF (0.50 mL, 0.50 mmol, 1.0 M in THF) added at 0 °C. The solution was stirred at 0 °C for 10 min, warmed to rt, and stirred for an additional 20 min. The reaction was quenched via the addition of saturated aq. NH_4Cl (20 mL). The aq. phase was extracted with Et_2O (2 x 15 mL). The combined organic phase was washed with H_2O (10 mL) and saturated aq. NaCl (10 mL), dried with $MgSO_4$, filtered, and the solvent removed in *vacuo*. The yellow solid was dissolved in minimum amount of CH_2Cl_2 and precipitated by the addition of hexanes to yield **S24** (49 mg, 88%) as a bright yellow solid. R_f = 0.25 (2:1 hexanes/CH_2Cl_2). Mp. = 150–155 °C (decomp). IR (ATR): 3295 (m), 3280 (m), 3062 (w), 2209 (w), 2184 (w), 1475 (m), 1440 (m). ^{1}H NMR (400 MHz, $CDCl_3$) δ 7.57–7.49 (m, 8H), 7.33–7.28 (m, 8H), 3.18 (s, 4H) ppm. ^{13}C NMR (100 MHz, $CDCl_3$) δ 132.6, 132.5, 128.7, 128.4, 125.8, 124.9, 117.3, 97.4, 91.1, 82.2, 81.6 ppm. MS (MALDI, DCTB): m/z = 524 [$M^{\bullet+}$]. HRMS (ESI): m/z = 547.1456 [$(M + Na)^+$], calcd. for ($C_{42}H_{20}Na^+$): 547.1457.

Di-radiaannulene 5

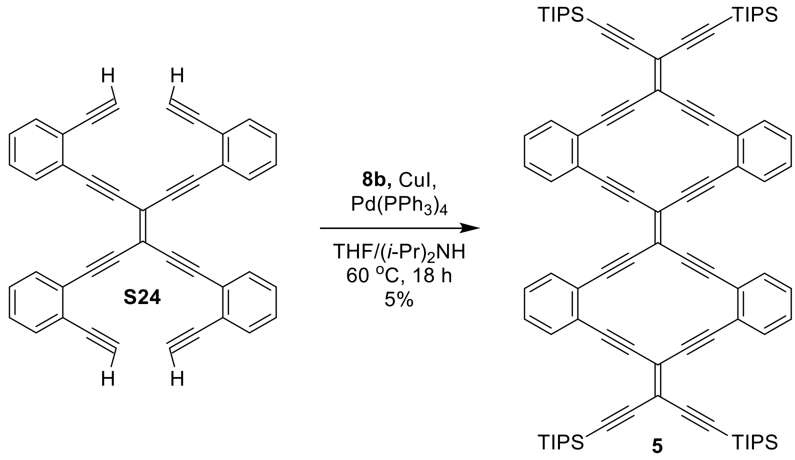

To a solution of **S24** (80 mg, 0.15 mmol) and **8b** (166 mg, 0.30 mmol) in deoxygenated, dry THF (15 mL) and (iPr)$_2$NH (15 mL) was added $Pd(PPh_3)_4$ (37 mg, 0.032 mmol) and CuI (6 mg, 0.03 mmol). The flask was sealed under nitrogen with a rubber septum, heated to 60 °C, and stirred for 18 h. The solution was cooled to rt and the reaction quenched via the addition of saturated aq. NH_4Cl (30 mL). The aq. phase was

extracted with Et_2O (2 x 30 mL). The combined organic phase was washed with H_2O (25 mL) and saturated aq. NaCl (25 mL), dried with $MgSO_4$, filtered, and the solvent removed in *vacuo*. Column chromatography (alumina, neutral, 5:1 to 2:1 hexanes/CH_2Cl_2) afforded a dark red solid. The solid was further purified by dissolving into a small amount of THF and precipitated by the addition of MeOH and cooling to –78 °C to yield **5** (10 mg, 5%) as a deep red solid.

Spectra

2-Bromo-4-*tert*-butyliodobenzene (S2)

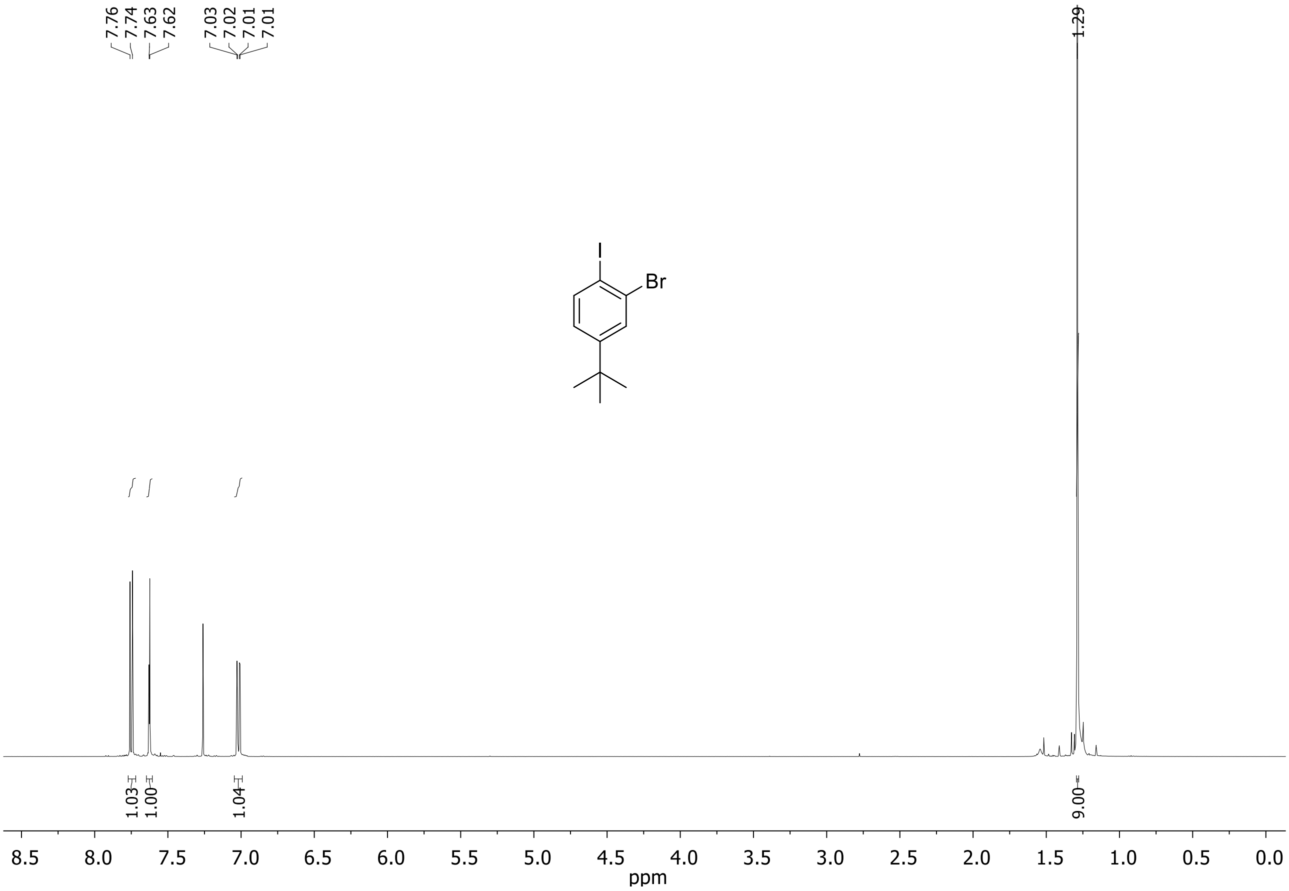

Supplementary Figure 1. ^{1}H-NMR spectrum of **S2** in $CDCl_3$ (500 MHz).

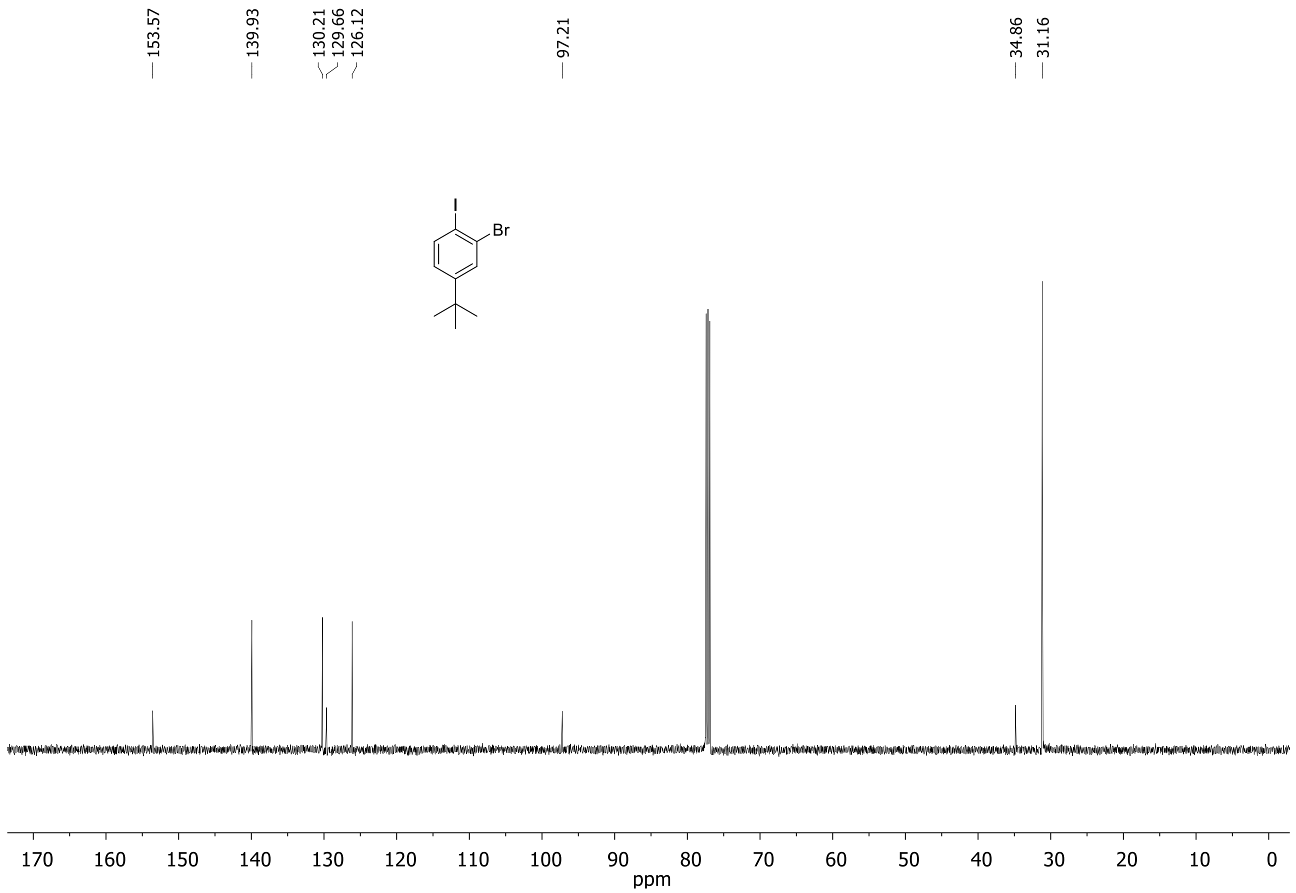

Supplementary Figure 2. ^{13}C spectrum of **S2** in $CDCl_3$ (126 MHz).

2-Bromo-4-*tert*-butyl(trimethylsilylethynyl)benzene (S3)

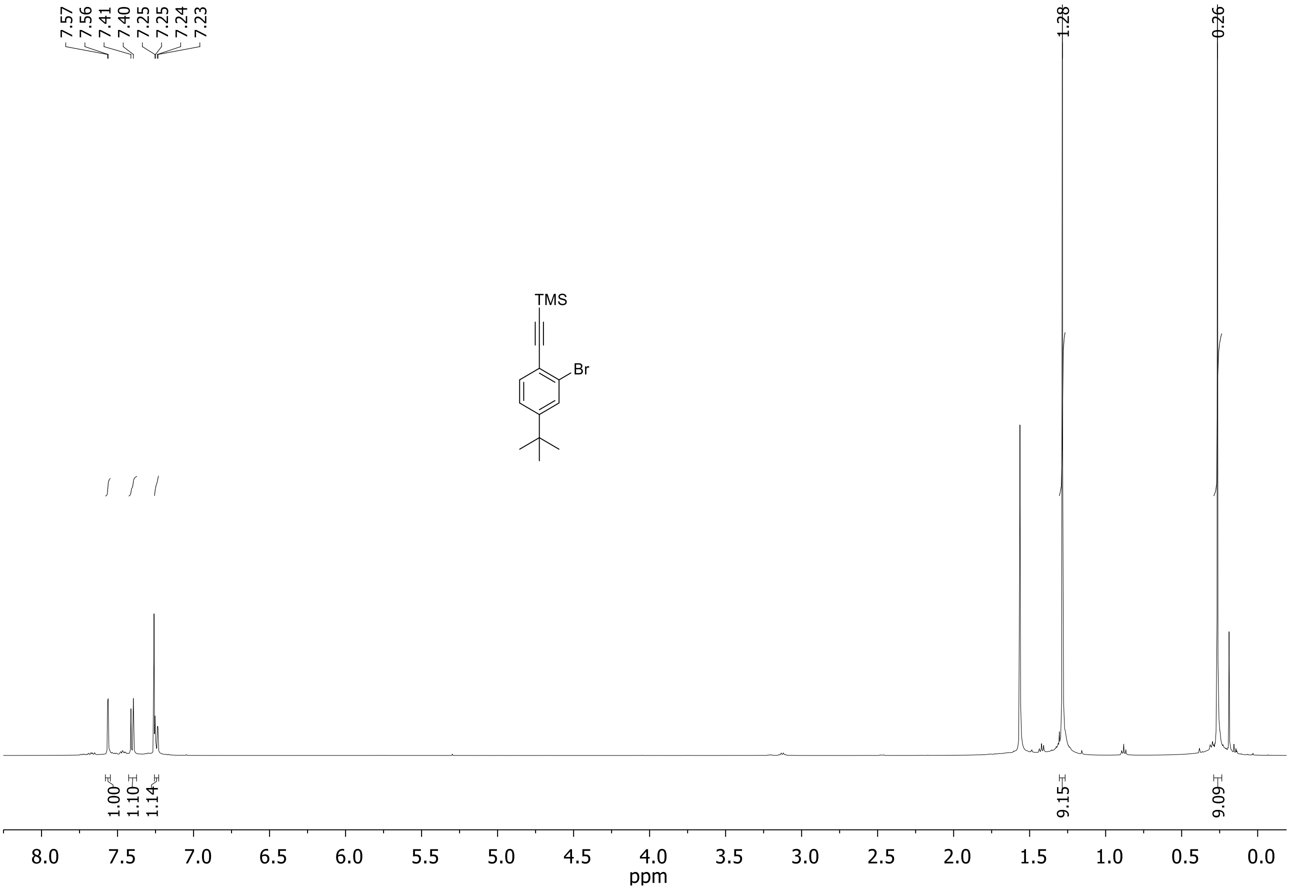

Supplementary Figure 3. ^{1}H-NMR spectrum of **S3** in $CDCl_3$ (500 MHz).

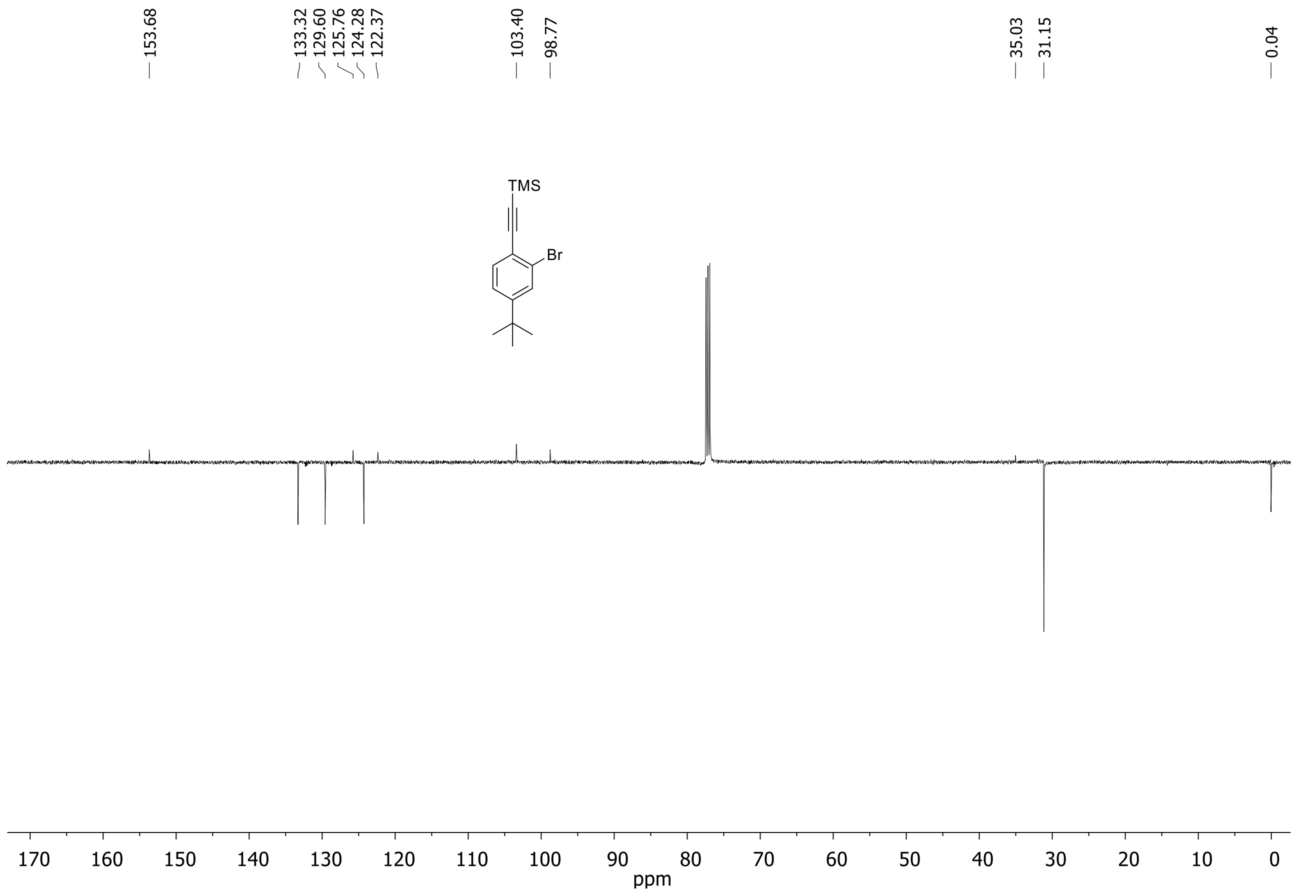

Supplementary Figure 4. ^{13}C-APT spectrum of **S3** in $CDCl_3$ (126 MHz).

2-Iodo-4-*tert*-butyl(trimethylsilylethynyl)benzene (S4)

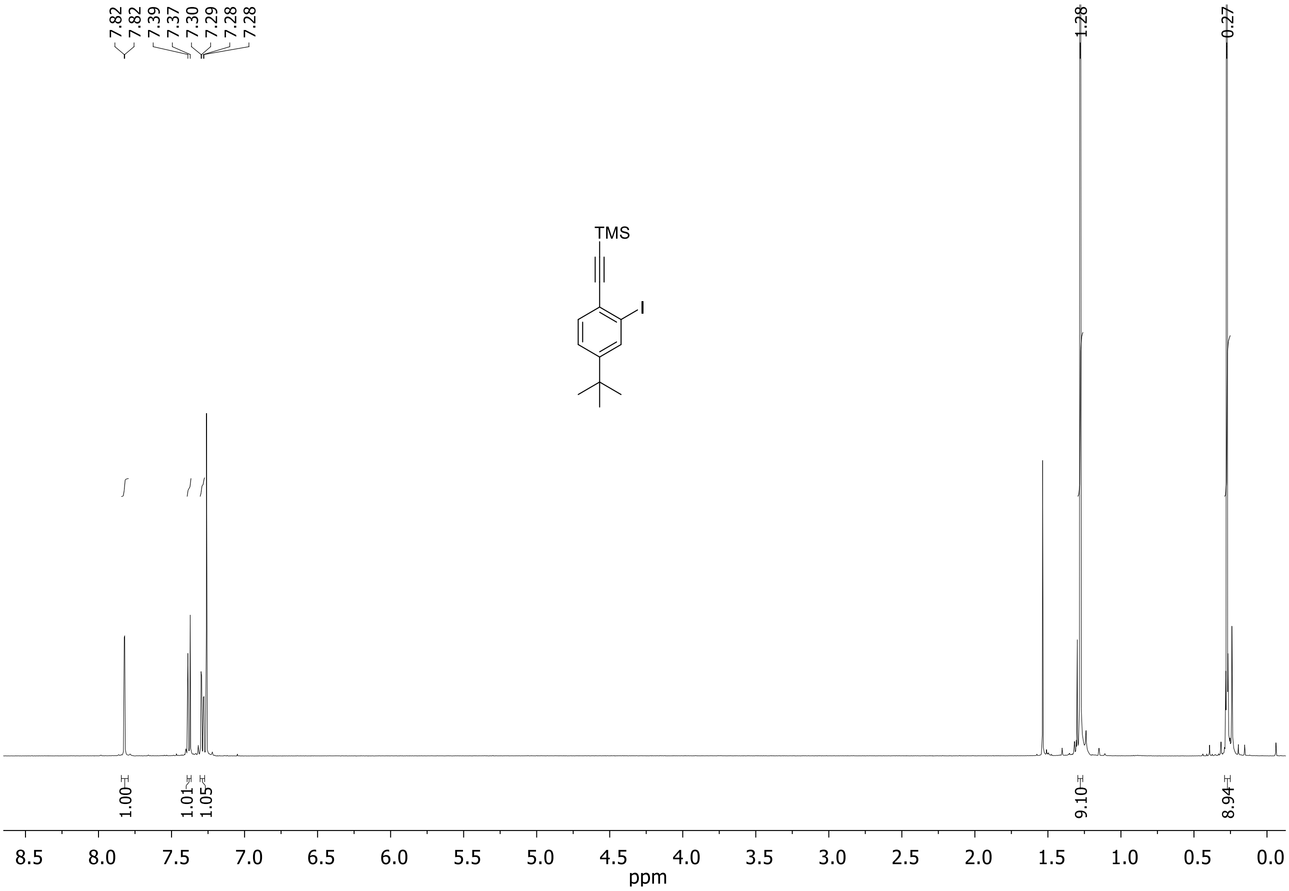

Supplementary Figure 5. ^{1}H-NMR spectrum of **S4** in $CDCl_3$ (500 MHz).

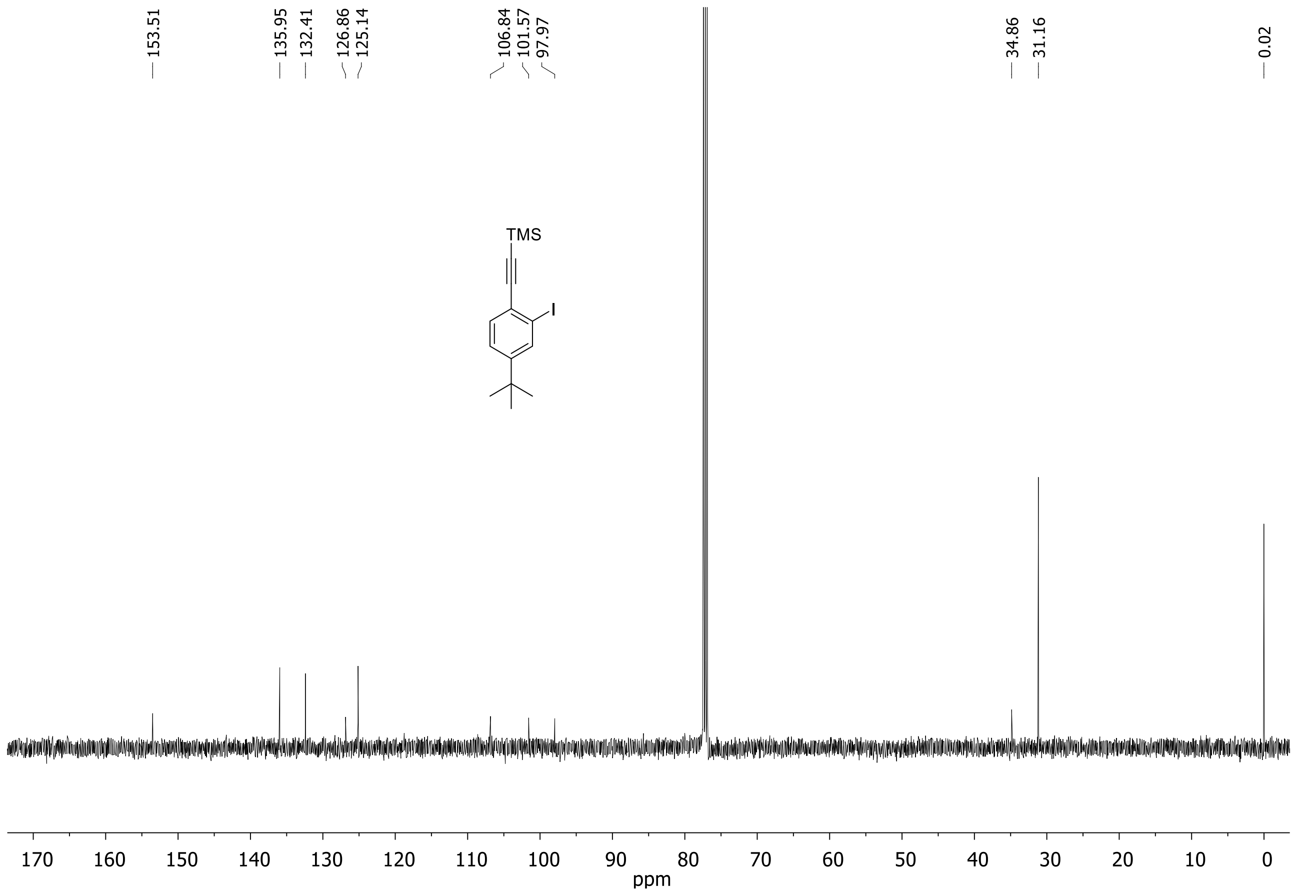

Supplementary Figure 6. ^{13}C spectrum of **S4** in $CDCl_3$ (126 MHz).

Compound **S5**

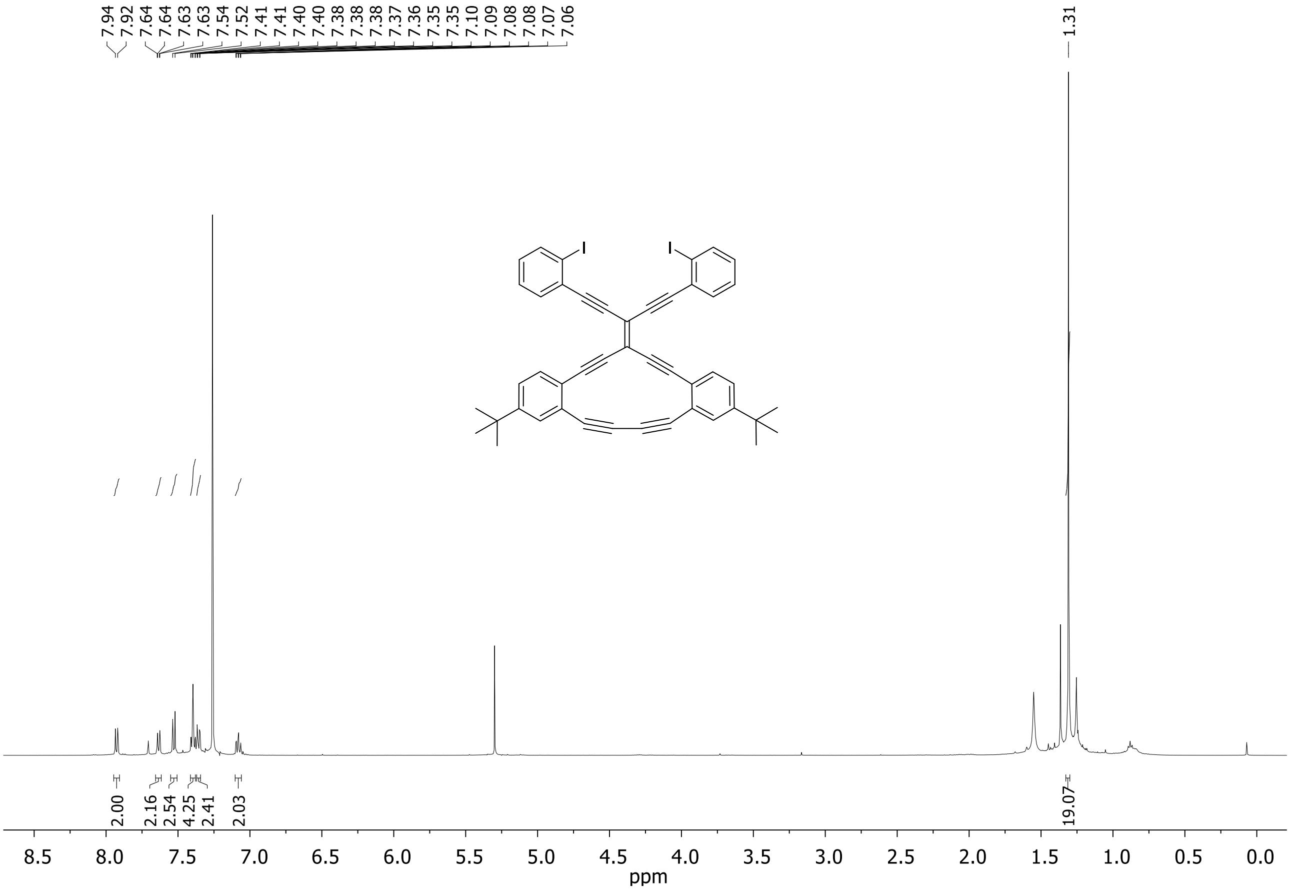

Supplementary Figure 7. ^{1}H-NMR spectrum of **S5** in $CDCl_3$ (500 MHz).

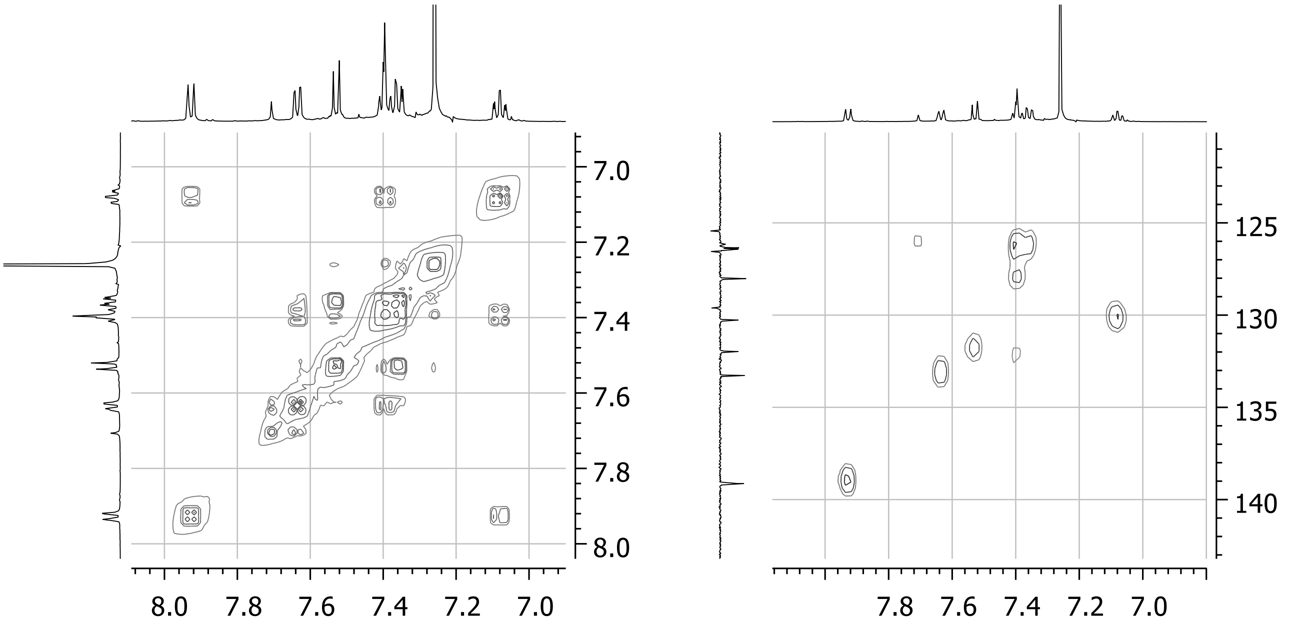

Supplementary Figure 8. COSY and (right) ^{1}H / ^{13}C-APT HSQC spectra of **S5** in $CDCl_3$ (500 / 126 MHz).

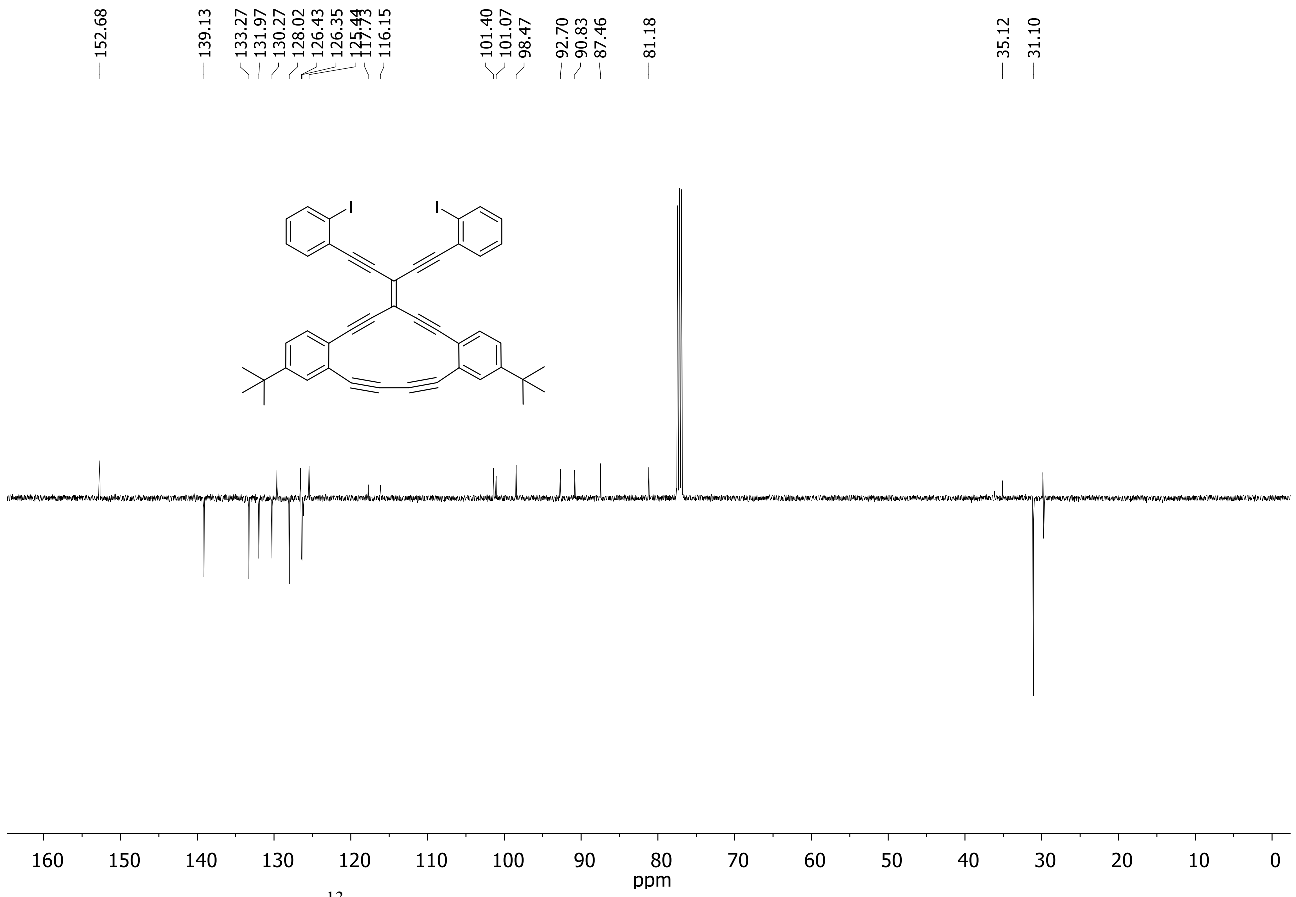

Supplementary Figure 9. ^{13}C-APT spectrum of **S5** in $CDCl_3$ (126 MHz).

Di-dehydrobenzannulene 1

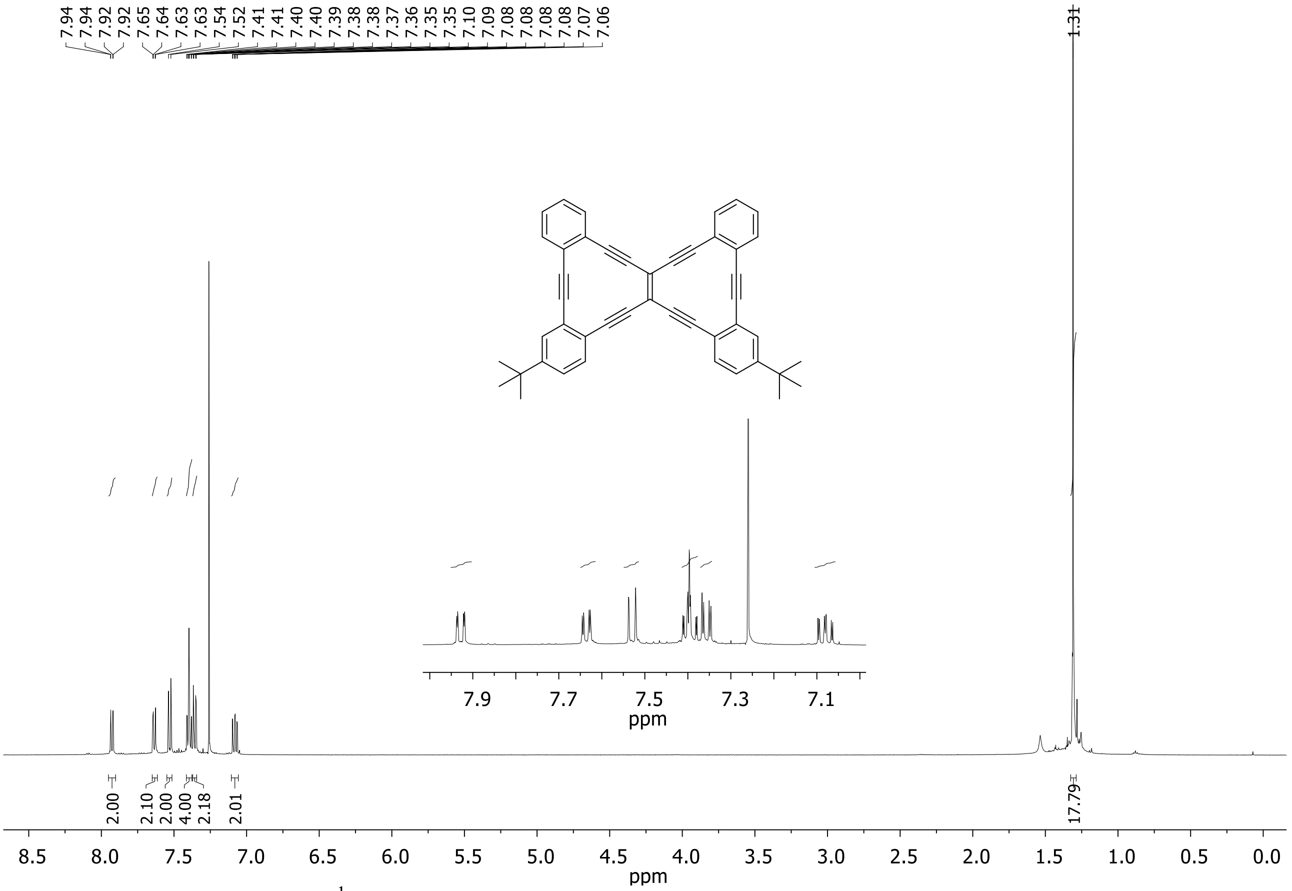

Supplementary Figure 10. ^{1}H-NMR spectrum of **1** in $CDCl_3$ (500 MHz).

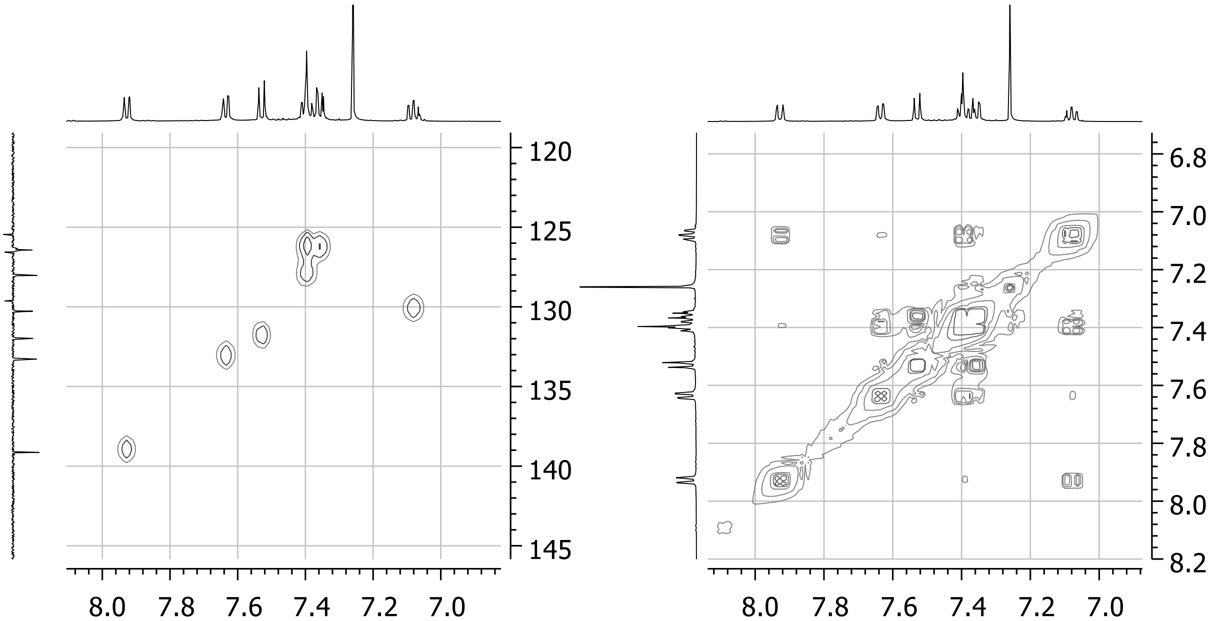

Supplementary Figure 11. COSY and (right) ^{1}H / ^{13}C-APT HSQC spectra of **1** in $CDCl_3$ (500 / 126 MHz).

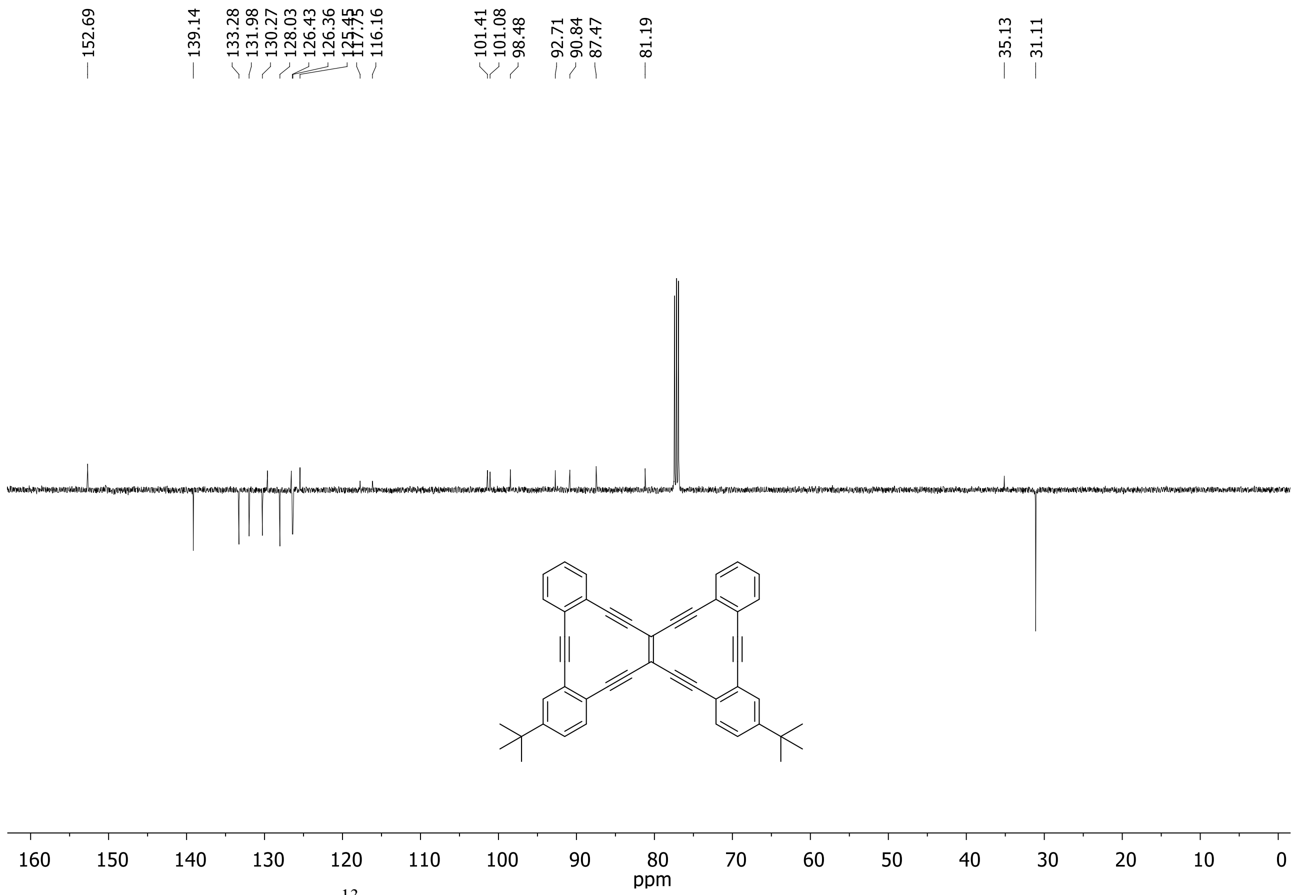

Supplementary Figure 12. ^{13}C-APT spectrum of **1** in $CDCl_3$ (126 MHz).

Di-radiaannulene 2

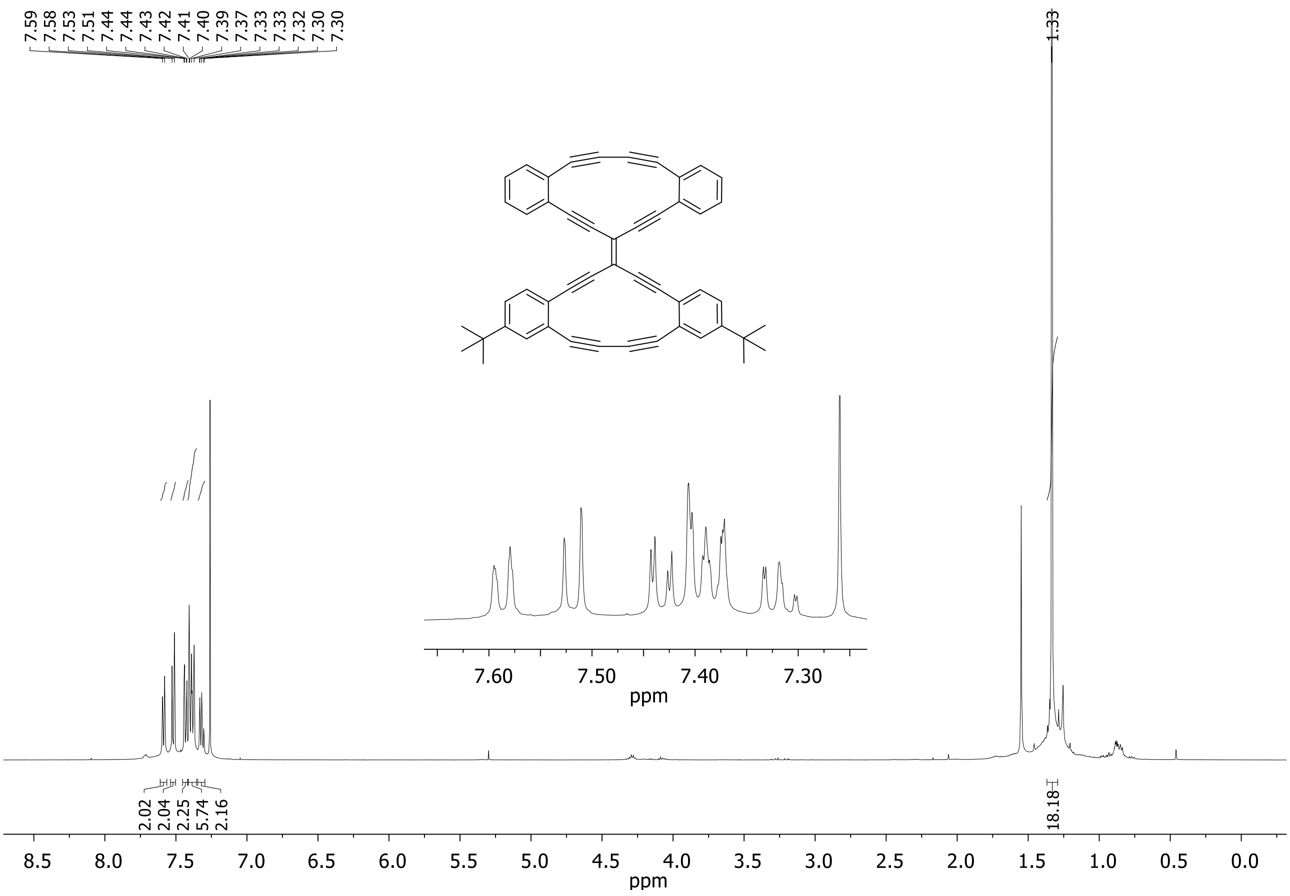

Supplementary Figure 13. ^{1}H-NMR spectrum of **2** in $CDCl_3$ (500 MHz).

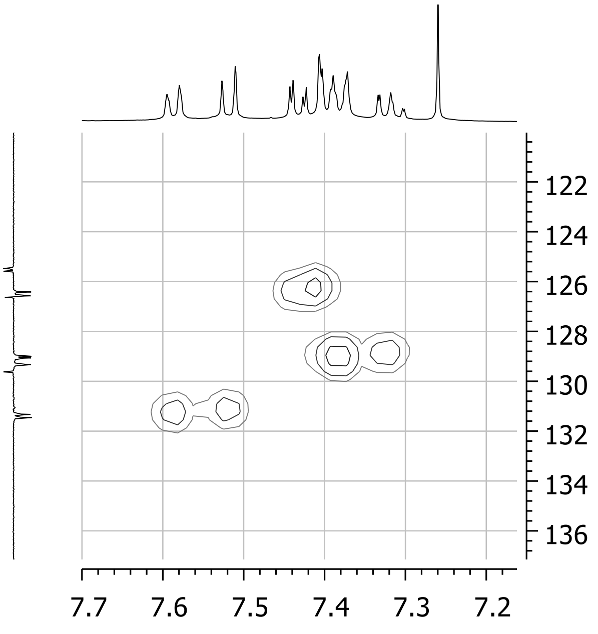

Supplementary Figure 14. ^{1}H / ^{13}C-APT HSQC spectrum of **2** in $CDCl_3$ (500 / 126 MHz).

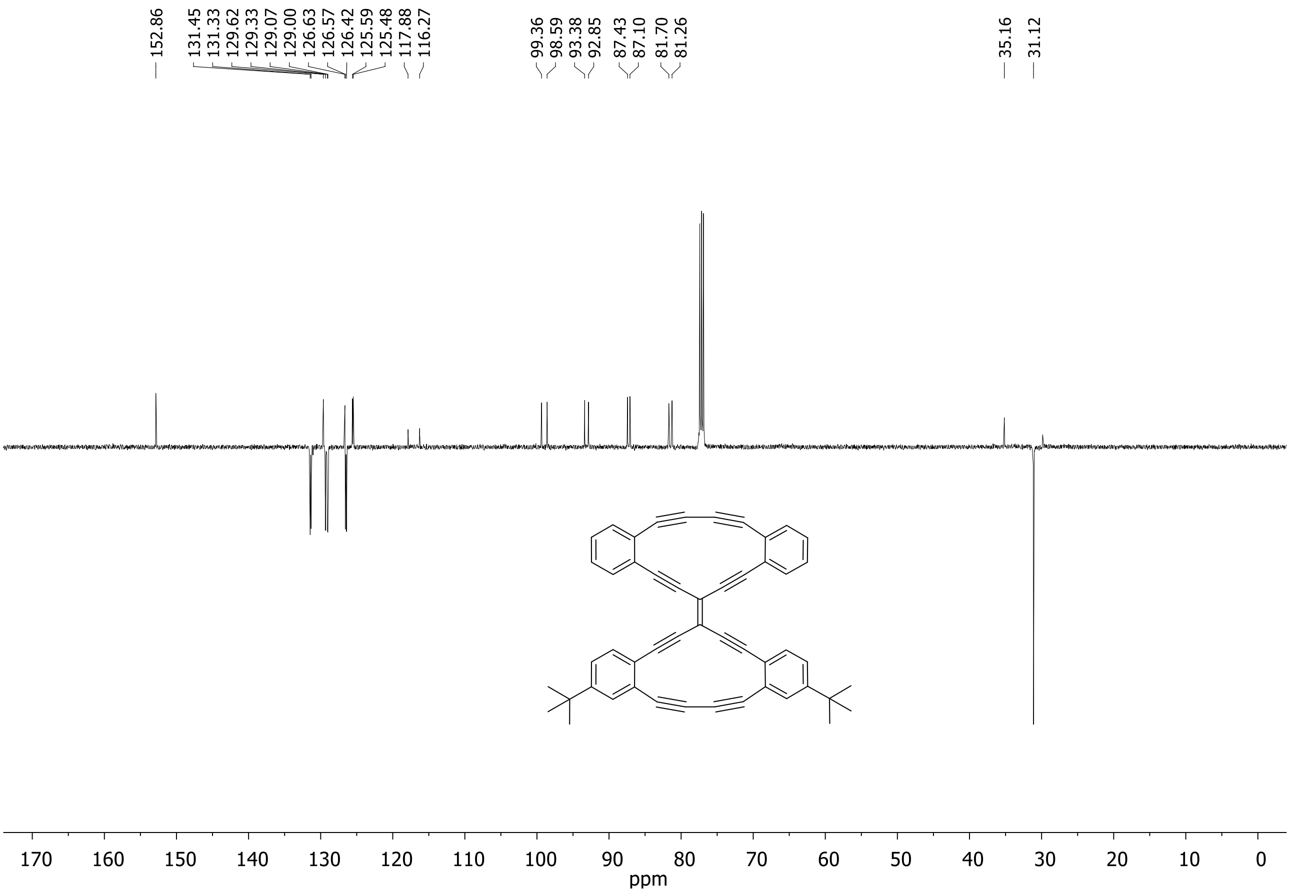

Supplementary Figure 15. ^{13}C-APT spectrum of **2** in $CDCl_3$ (126 MHz).

Tri-radiaannulene 3

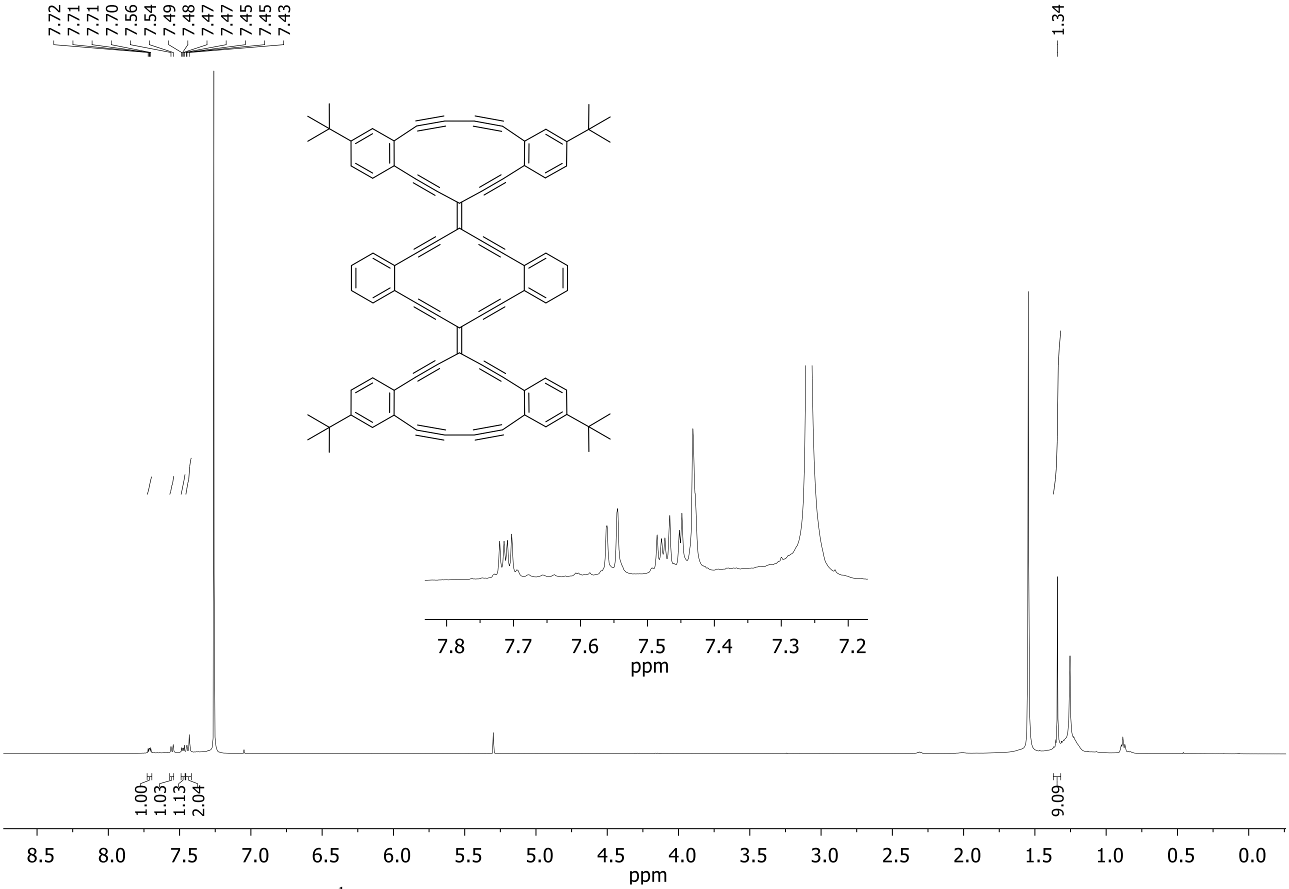

Supplementary Figure 16. ^{1}H-NMR spectrum of **3** in $CDCl_3$ (500 MHz).

The signals at 0.88 and 1.25 ppm are assigned to a small amount of grease. The NMR spectrum is recorded from a saturated solution (the compound has limited solubility), and therefore an up-concentration of residual grease relative to the compound likely occurs.

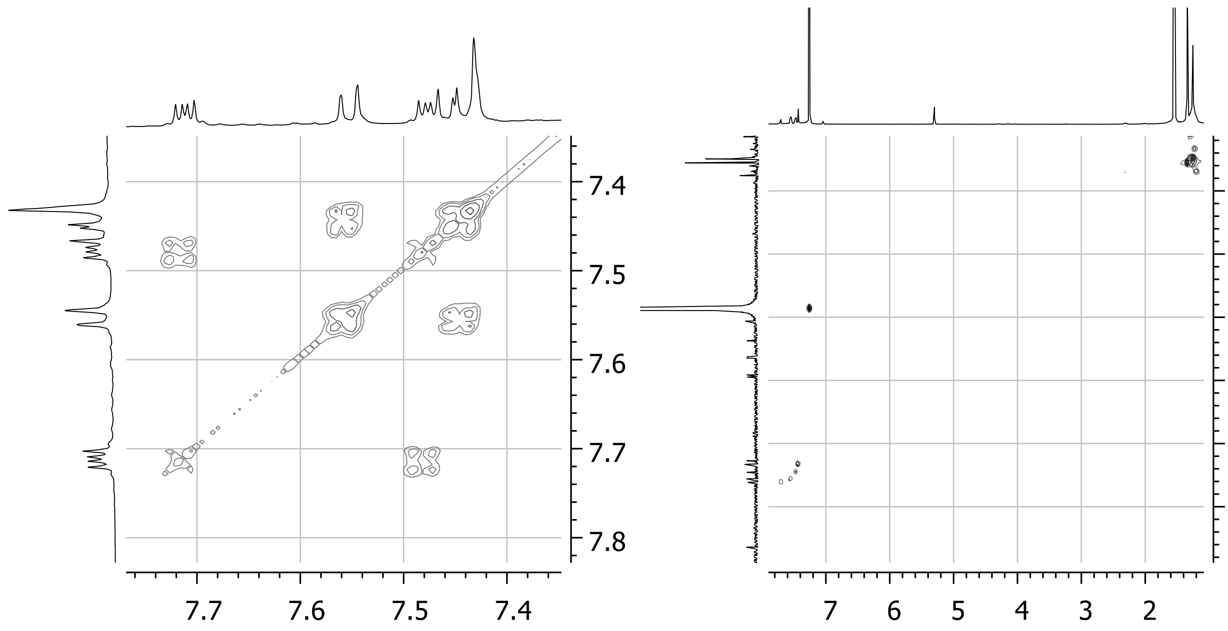

Supplementary Figure 17. COSY and (right) ^{1}H / ^{13}C-APT HSQC spectra of **3** in $CDCl_3$ (500 / 126 MHz).

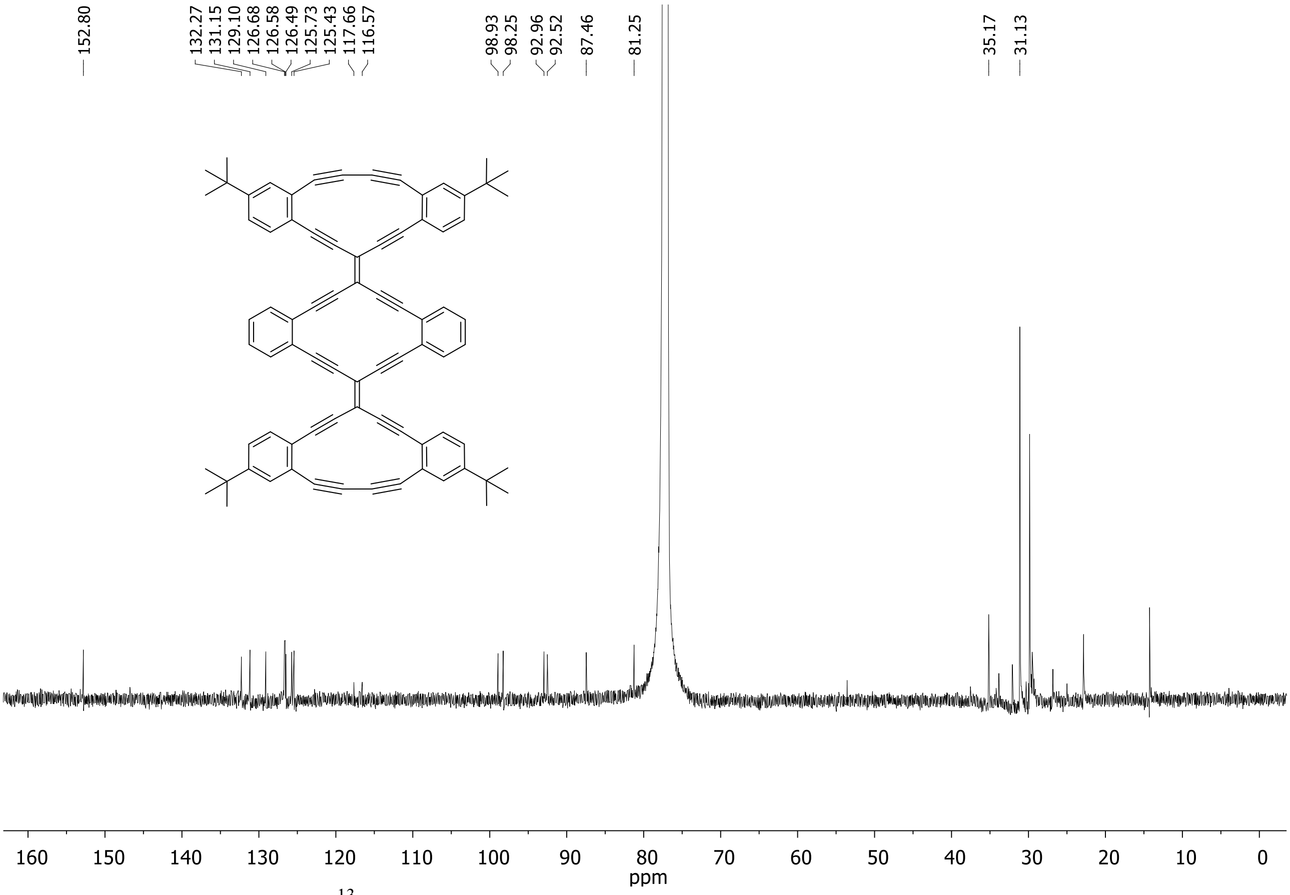

Supplementary Figure 18. ^{13}C-NMR spectrum of **3** in $CDCl_3$ (126 MHz).

4-*tert*-Butyl-2-(triisopropylsilylethynyl)(trimethylsilylethynyl)benzene (7)

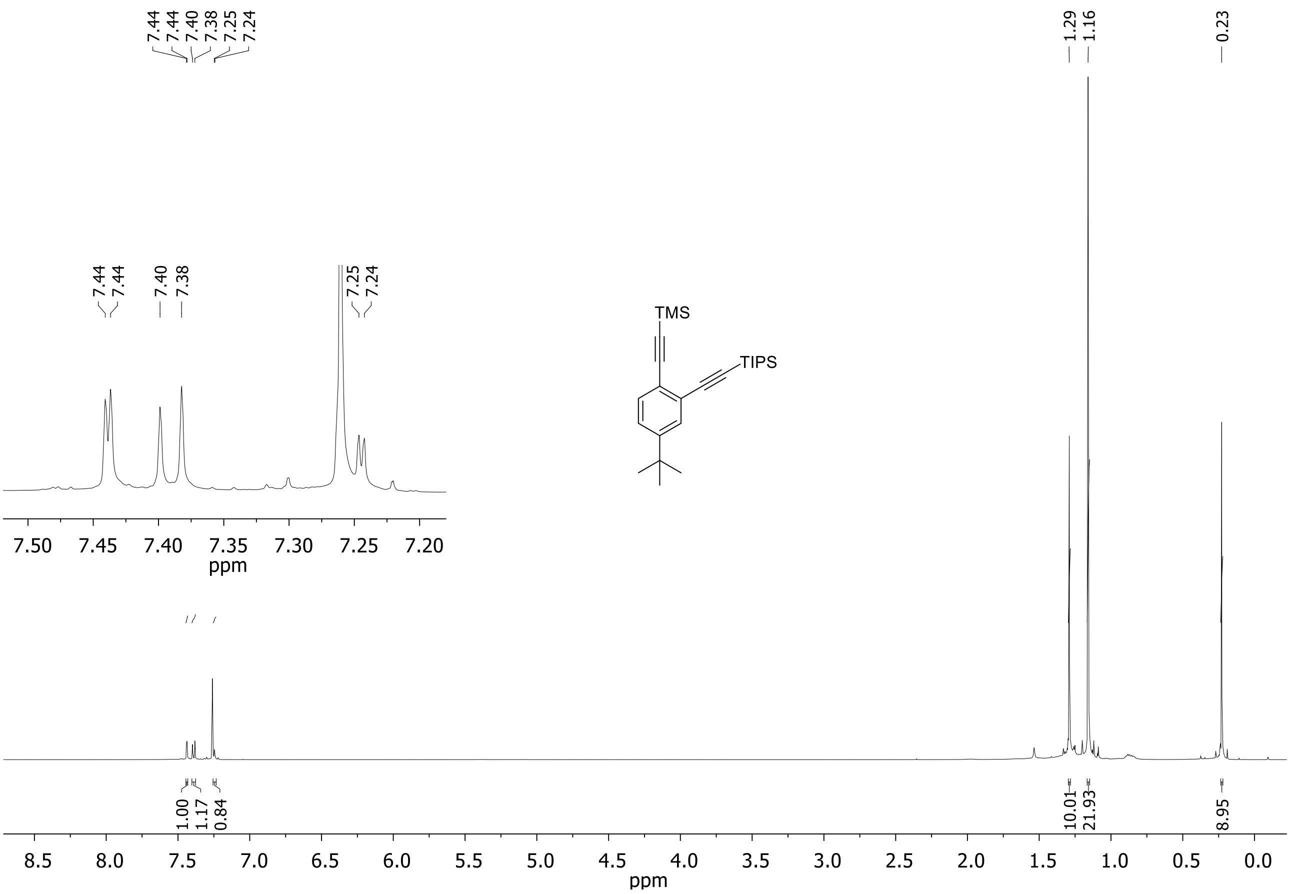

Supplementary Figure 19.^{1}H-NMR spectrum of **7** in $CDCl_3$ (500 MHz).

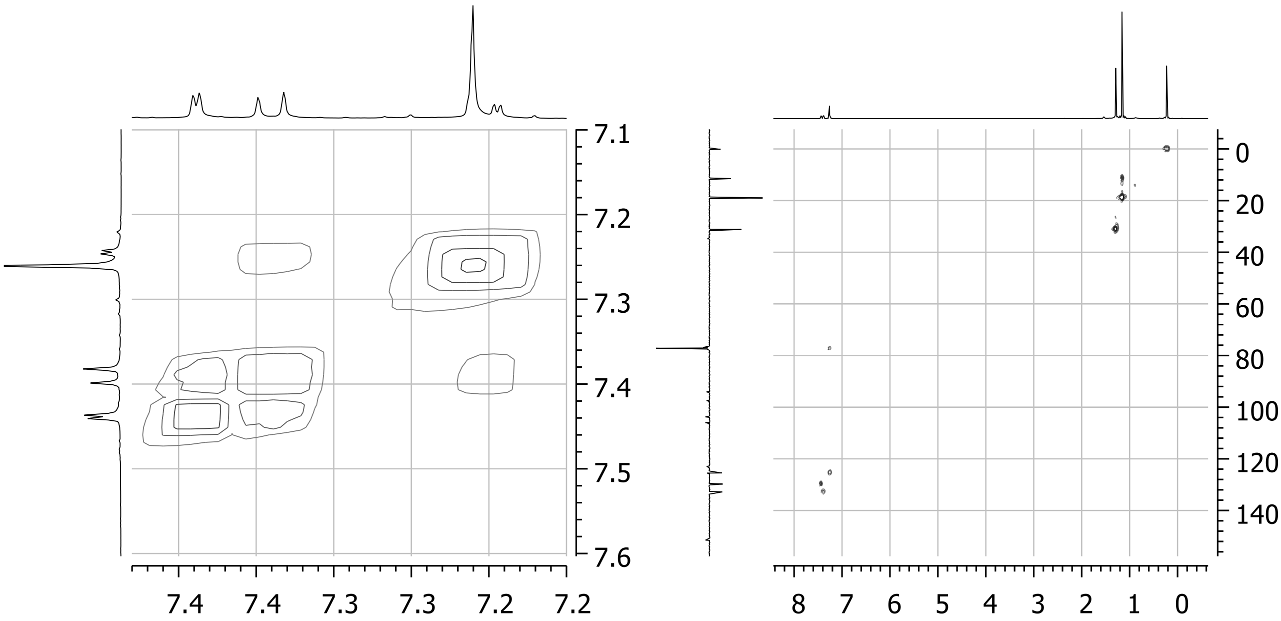

Supplementary Figure 20. COSY and (right) ^{1}H / ^{13}C-APT HSQC spectra of **7** in $CDCl_3$ (500 / 126 MHz).

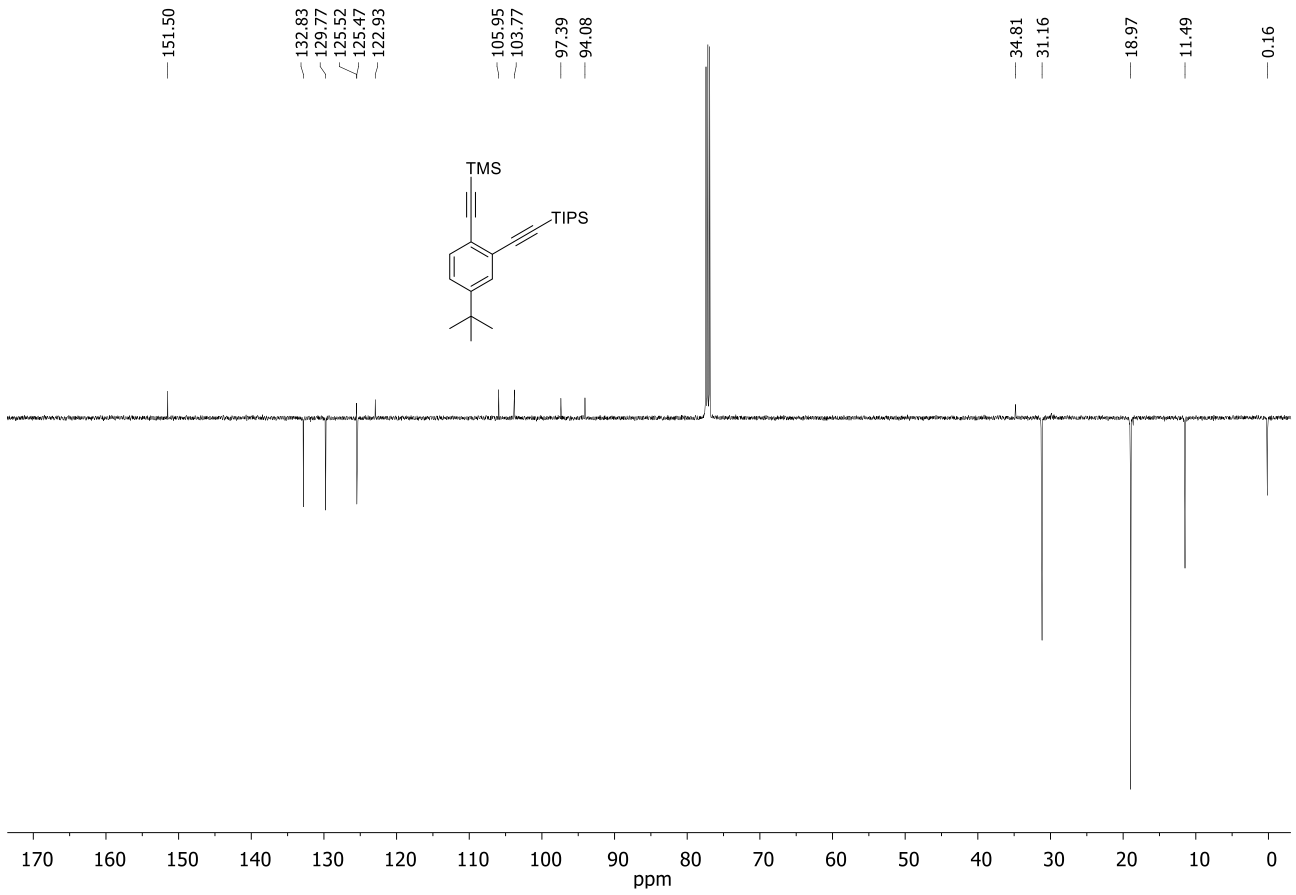

Supplementary Figure 21. ^{13}C-APT spectrum of **7** in $CDCl_3$ (126 MHz).

Compound **9**

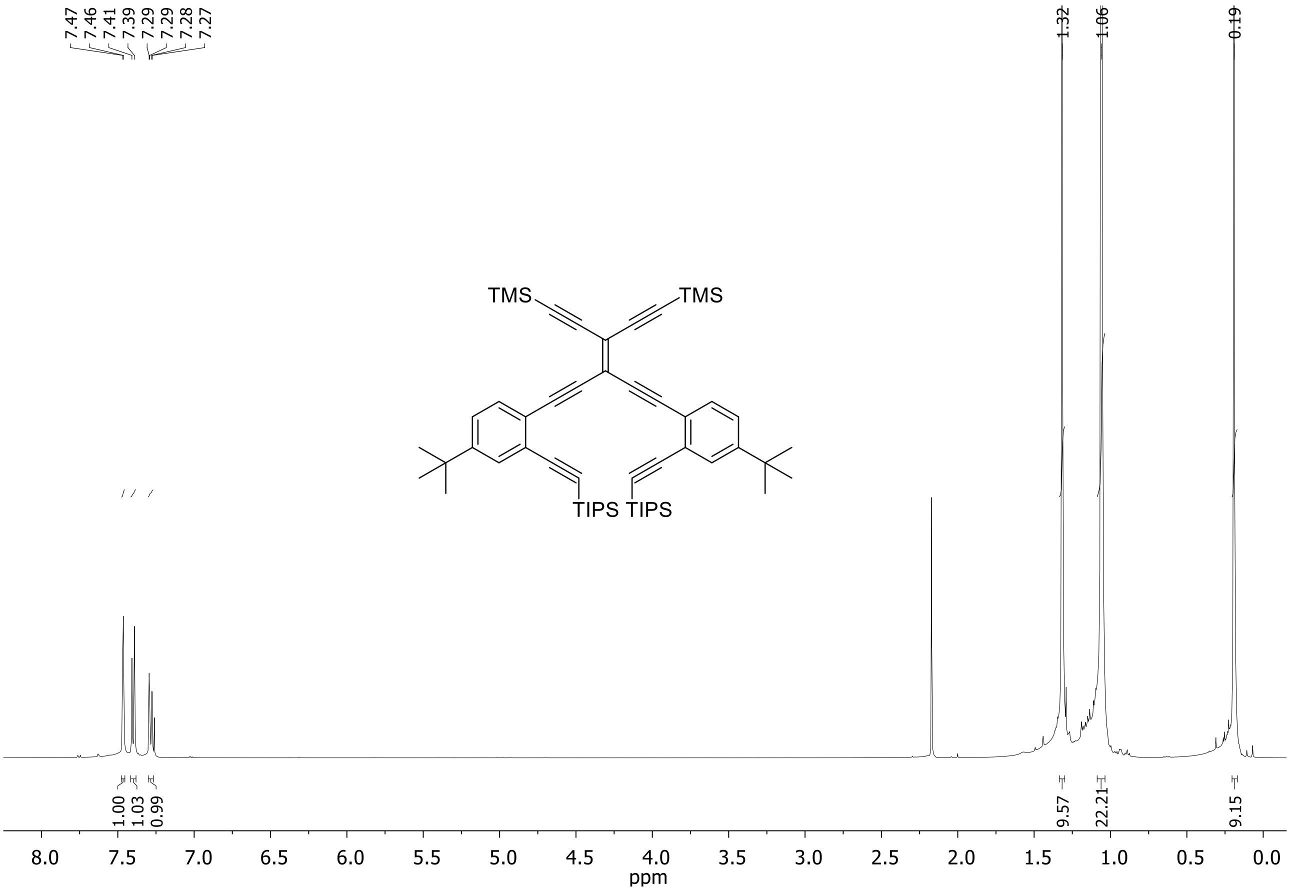

Supplementary Figure 22. ^{1}H-NMR spectrum of **9** in $CDCl_3$ (500 MHz).

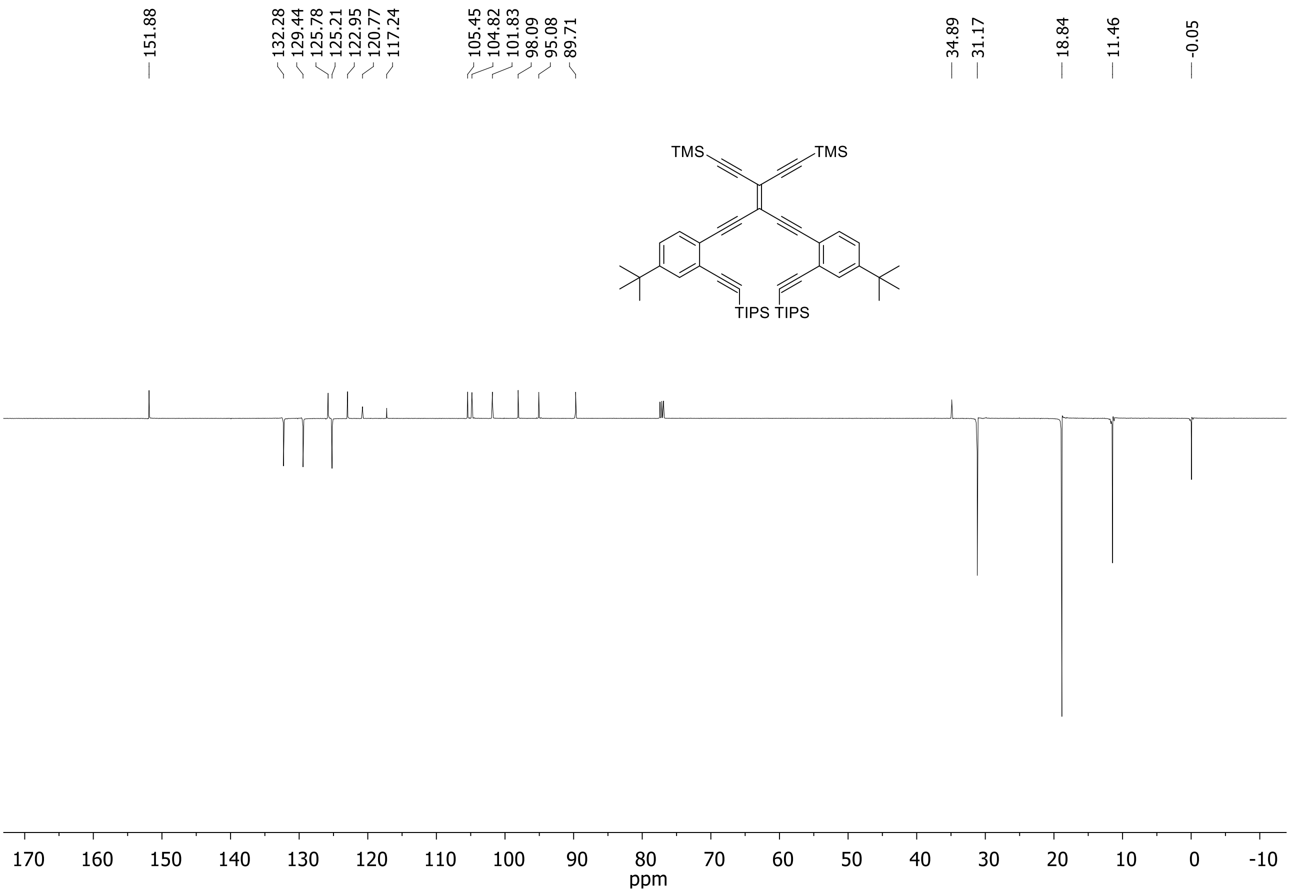

Supplementary Figure 23. ^{13}C-APT spectrum of **9** in $CDCl_3$ (126 MHz).

Compound **10**

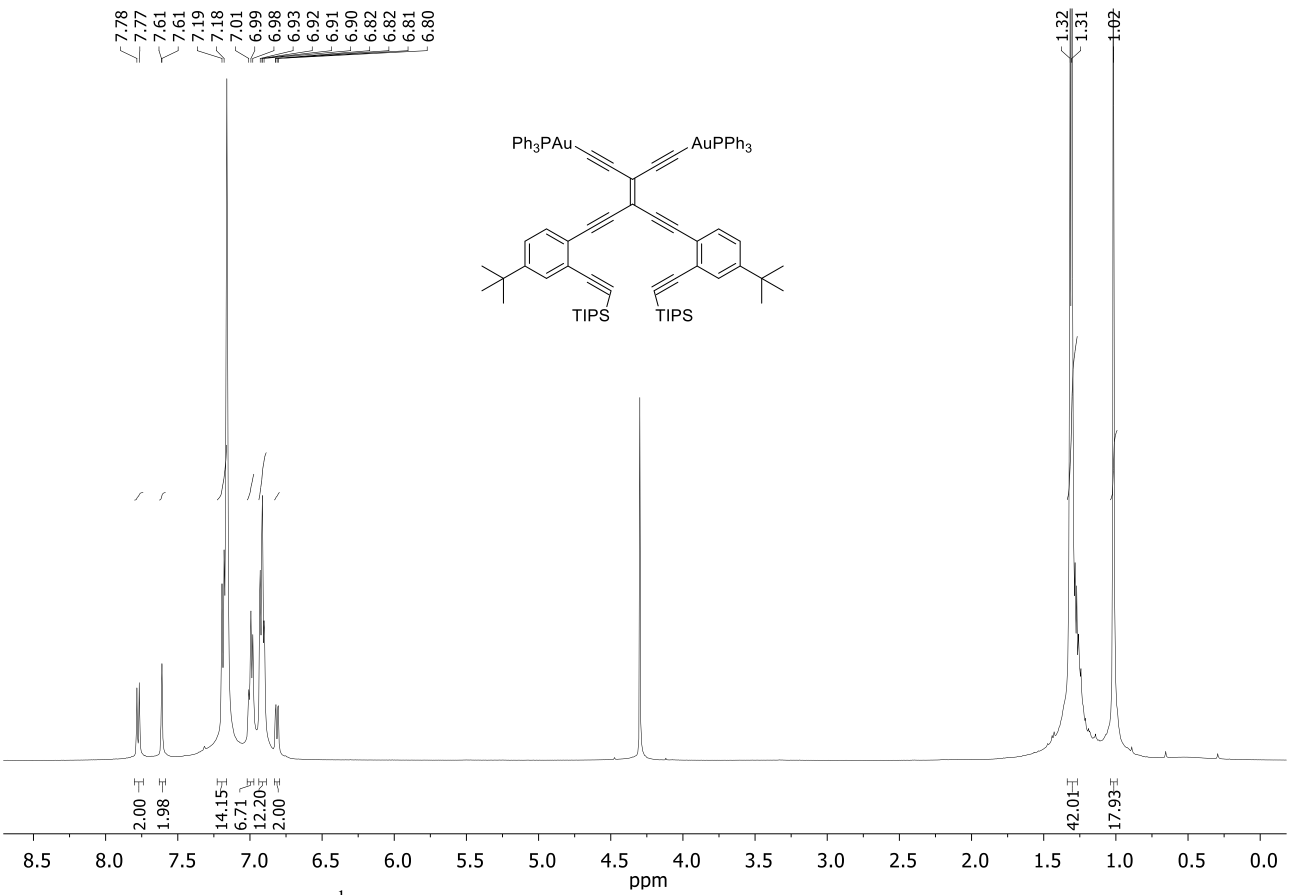

Supplementary Figure 24. ^{1}H-NMR spectrum of **10** in $CDCl_3$ (500 MHz).

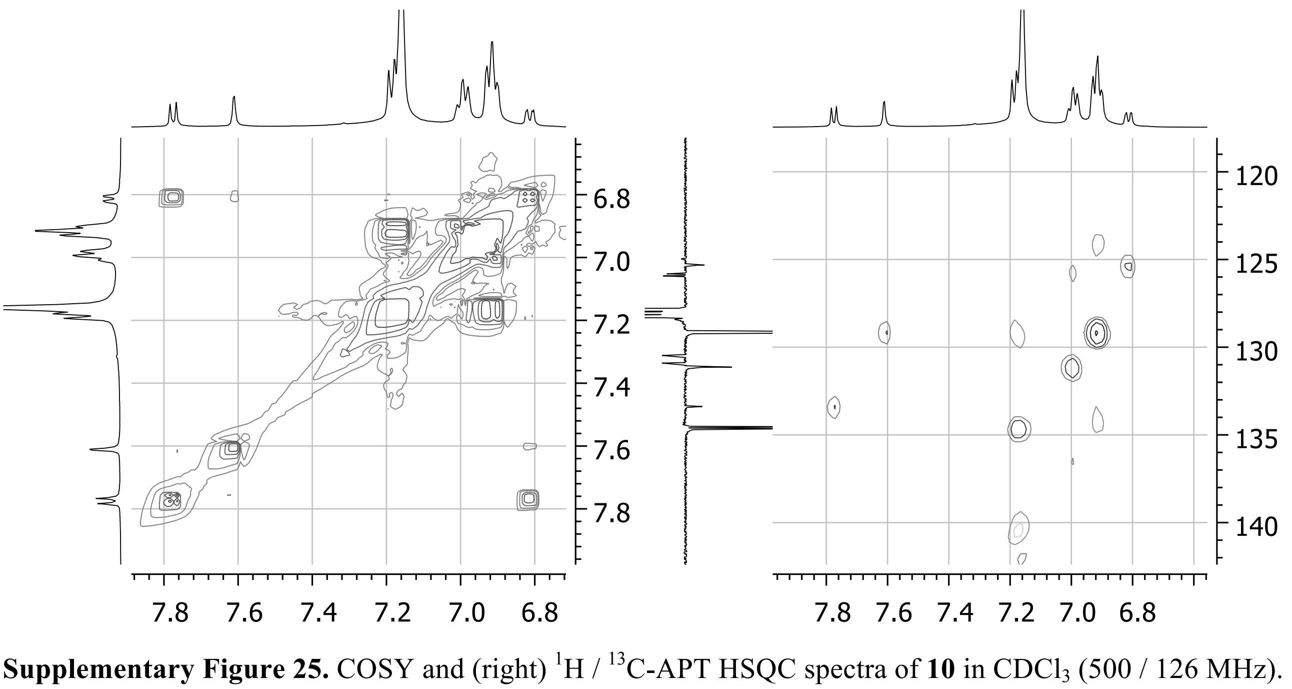

Supplementary Figure 25. COSY and (right) ^{1}H / ^{13}C-APT HSQC spectra of **10** in $CDCl_3$ (500 / 126 MHz).

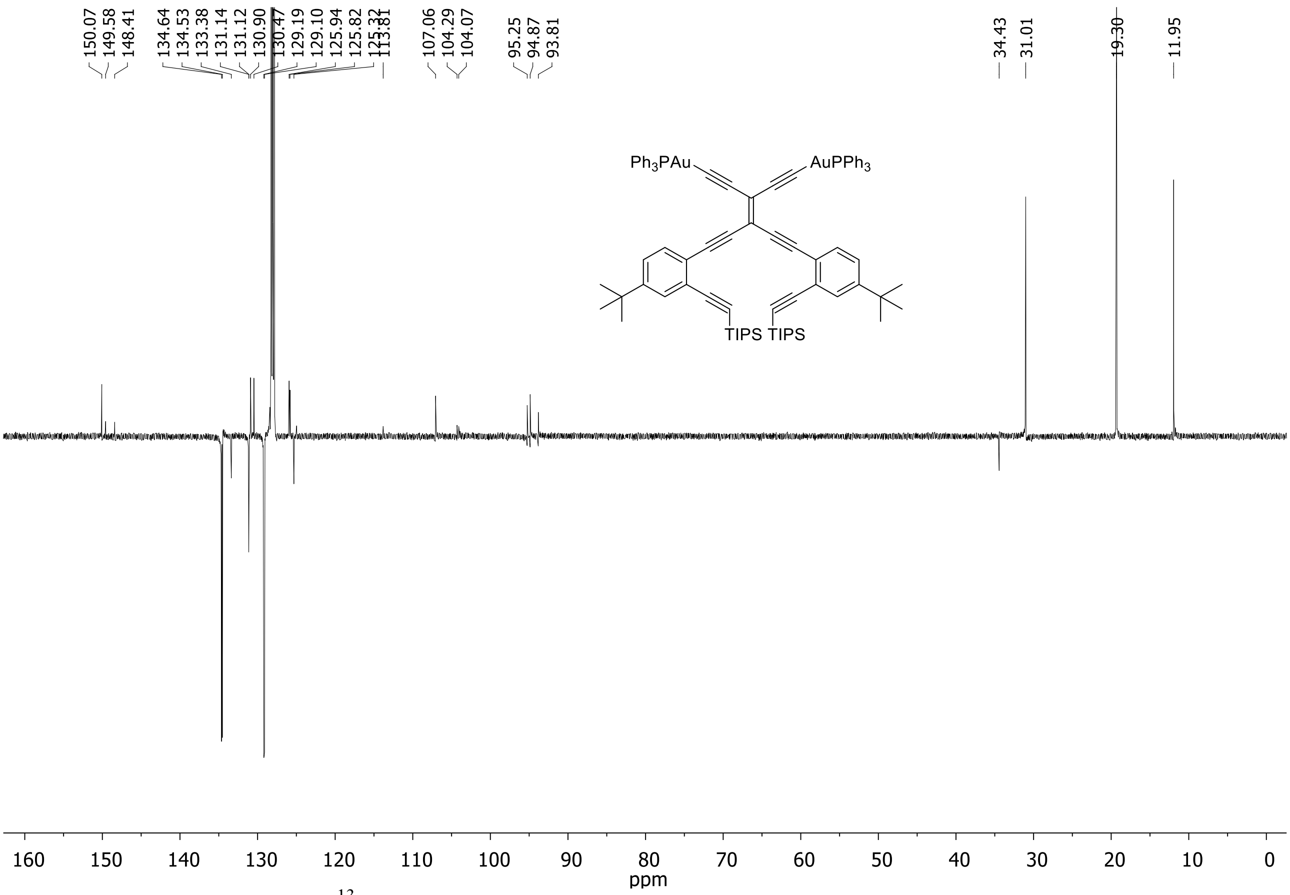

Supplementary Figure 26. ^{13}C-APT spectrum of **10** in $CDCl_3$ (126 MHz).

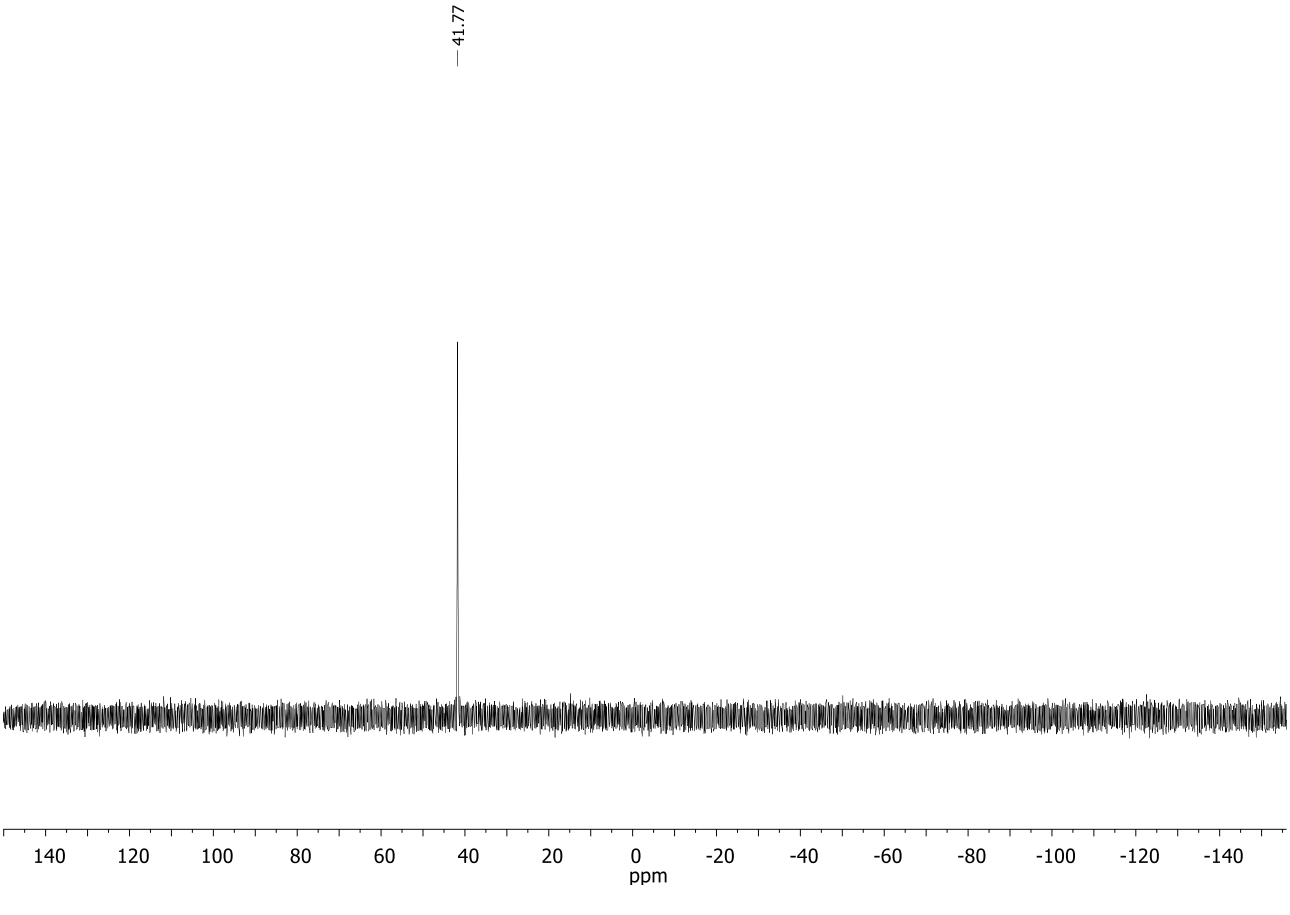

Supplementary Figure 27. ^{31}P-NMR spectrum of **10** in $CDCl_3$ (121 MHz).

Compound **11**

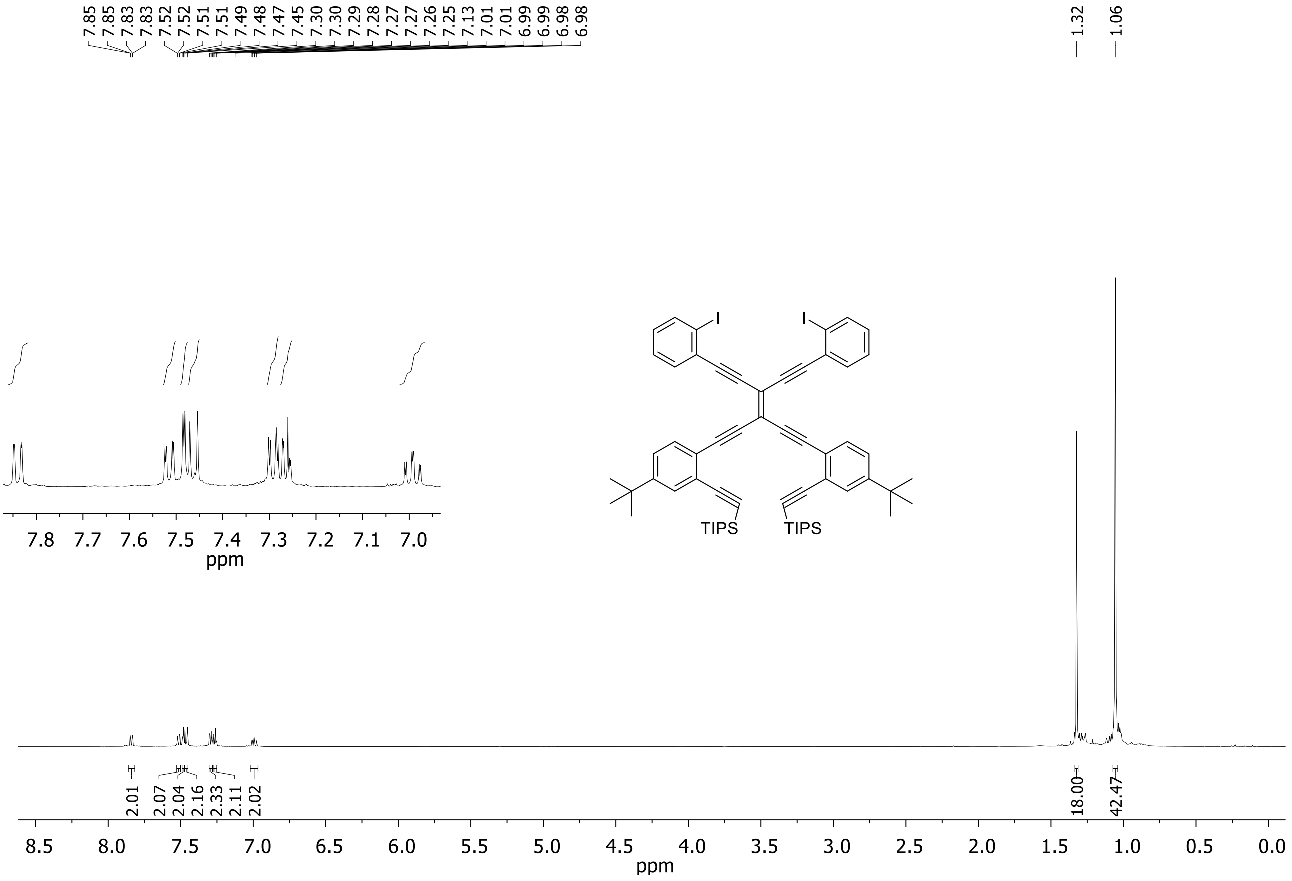

Supplementary Figure 28. ^{1}H-NMR spectrum of **11** in $CDCl_3$ (500 MHz).

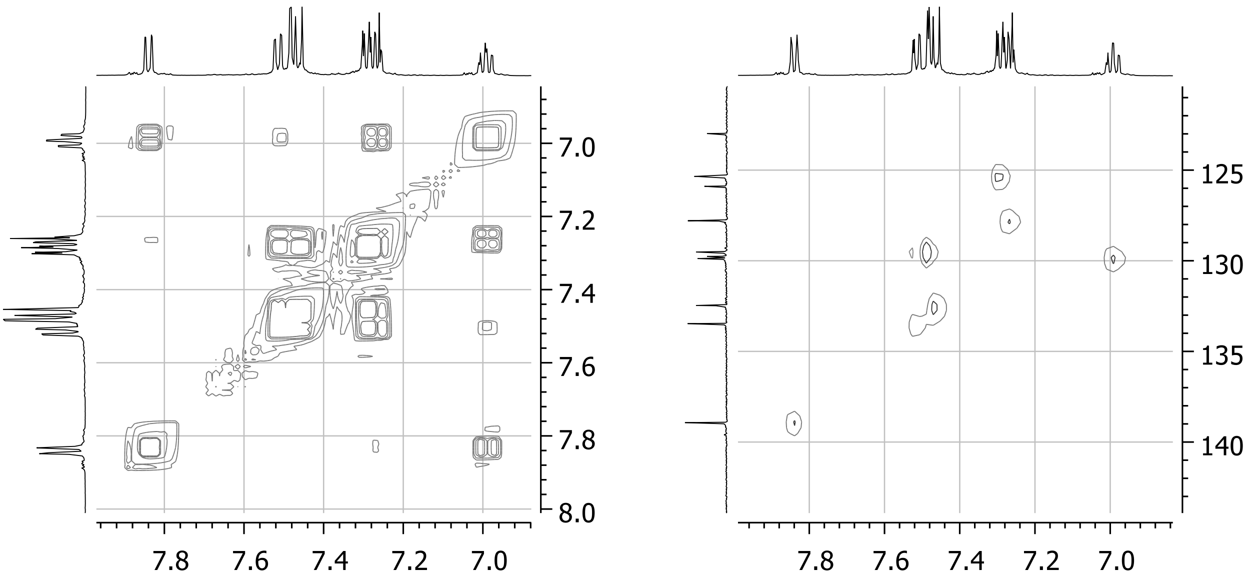

Supplementary Figure 29. COSY and (right) ^{1}H / ^{13}C-APT HSQC spectra of **11** in $CDCl_3$ (500 / 126 MHz).

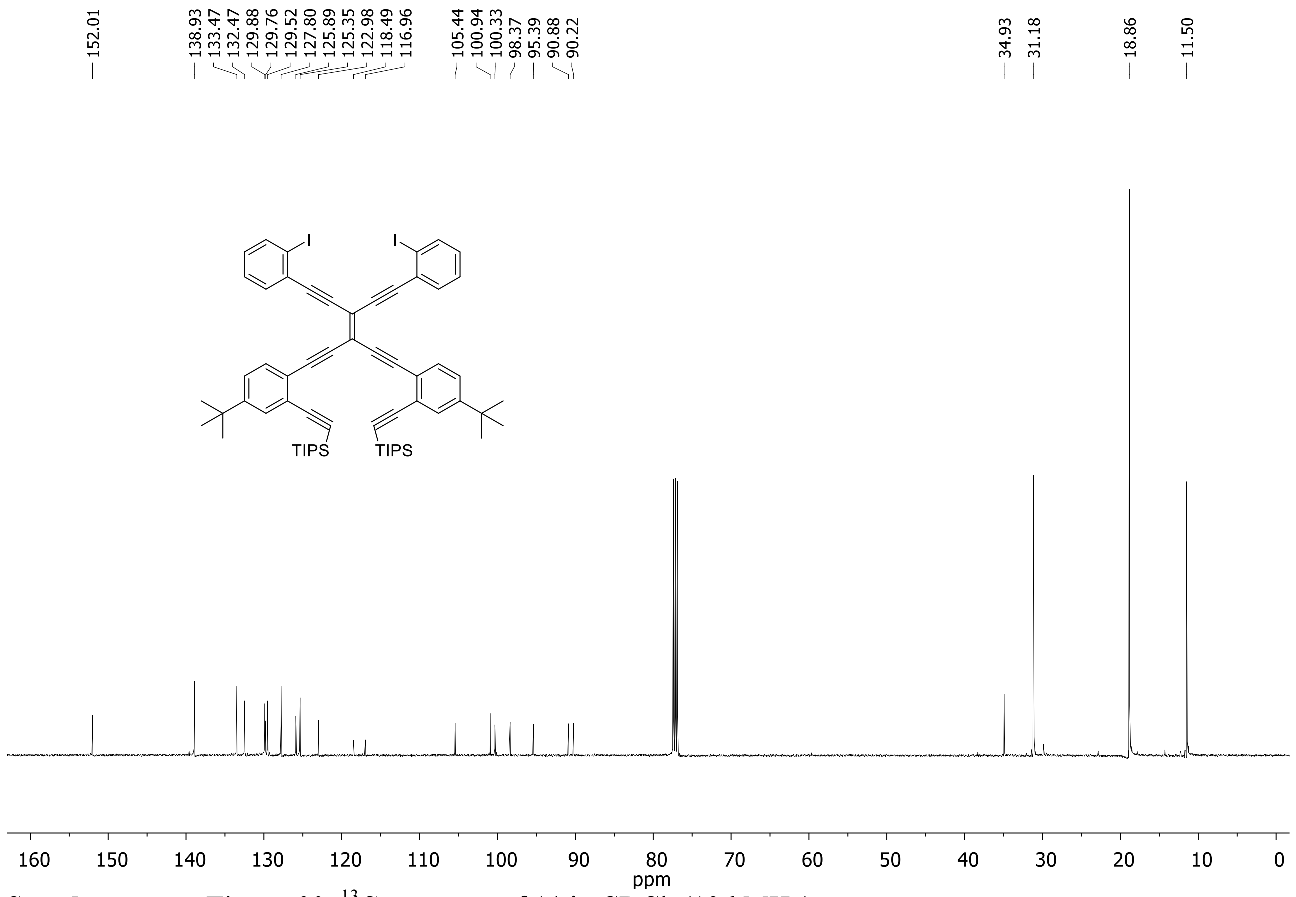

Supplementary Figure 30. ^{13}C spectrum of **11** in $CDCl_3$ (126 MHz).

Compound **12**

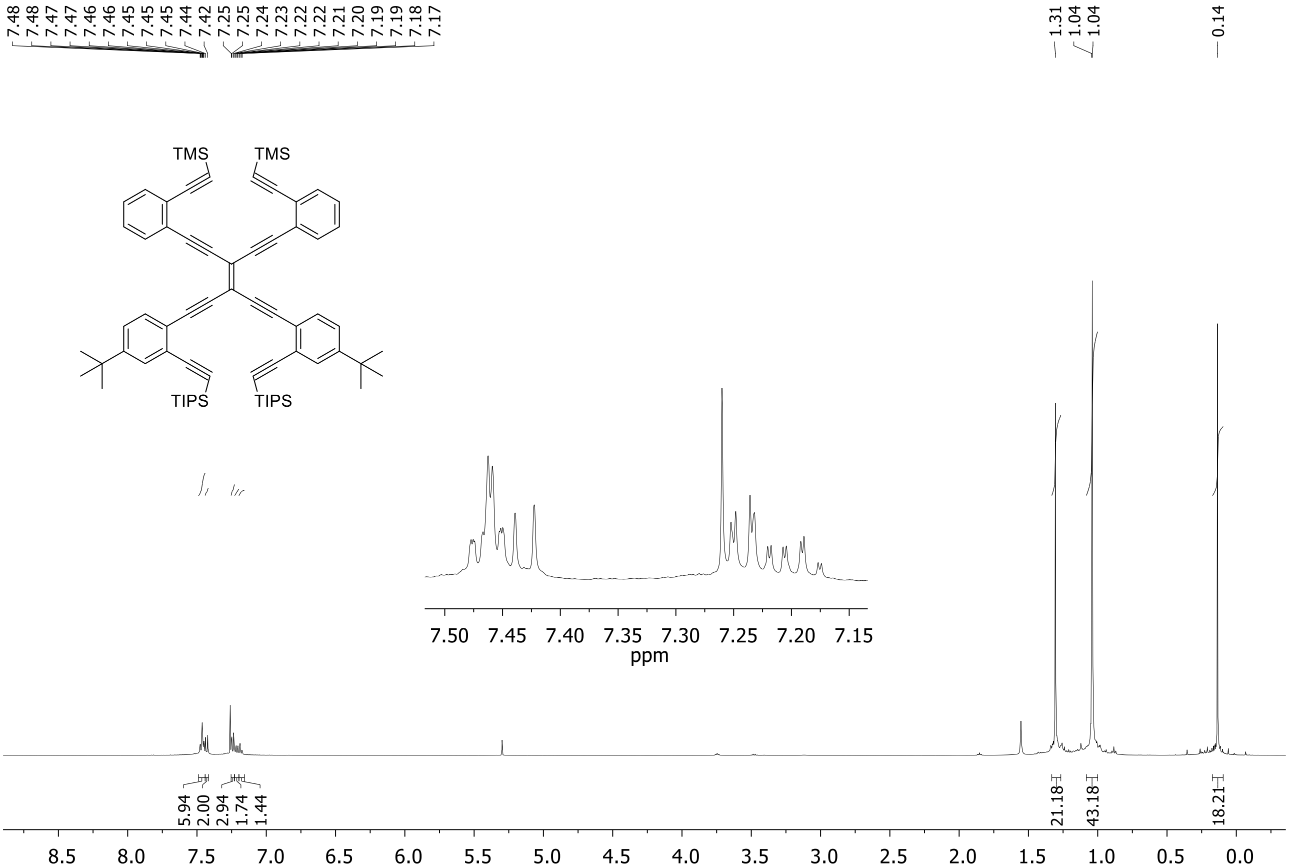

Supplementary Figure 31. ^{1}H-NMR spectrum of **12** in $CDCl_3$ (500 MHz).

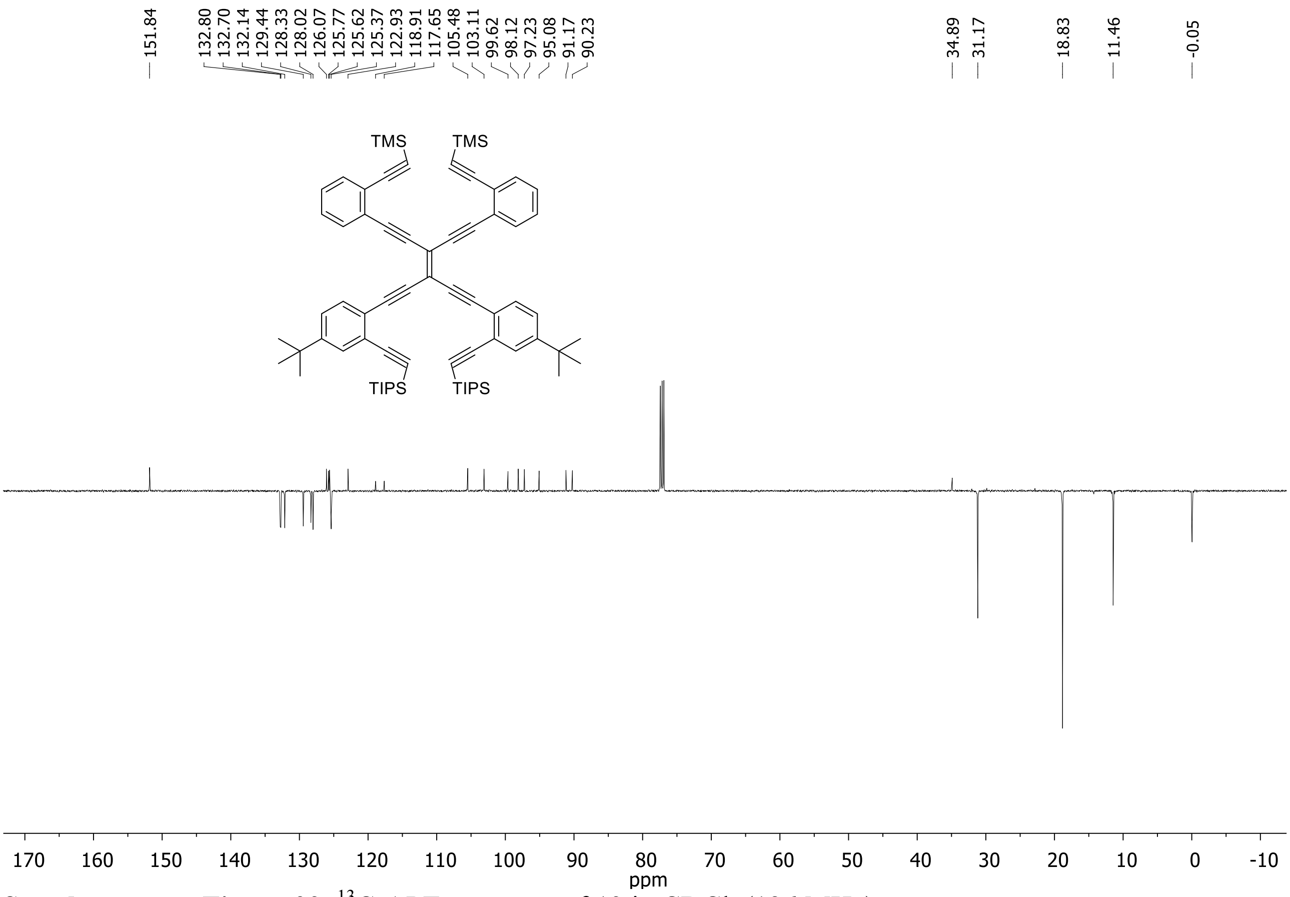

Supplementary Figure 32. ^{13}C-APT spectrum of **12** in $CDCl_3$ (126 MHz).

Compound **13**

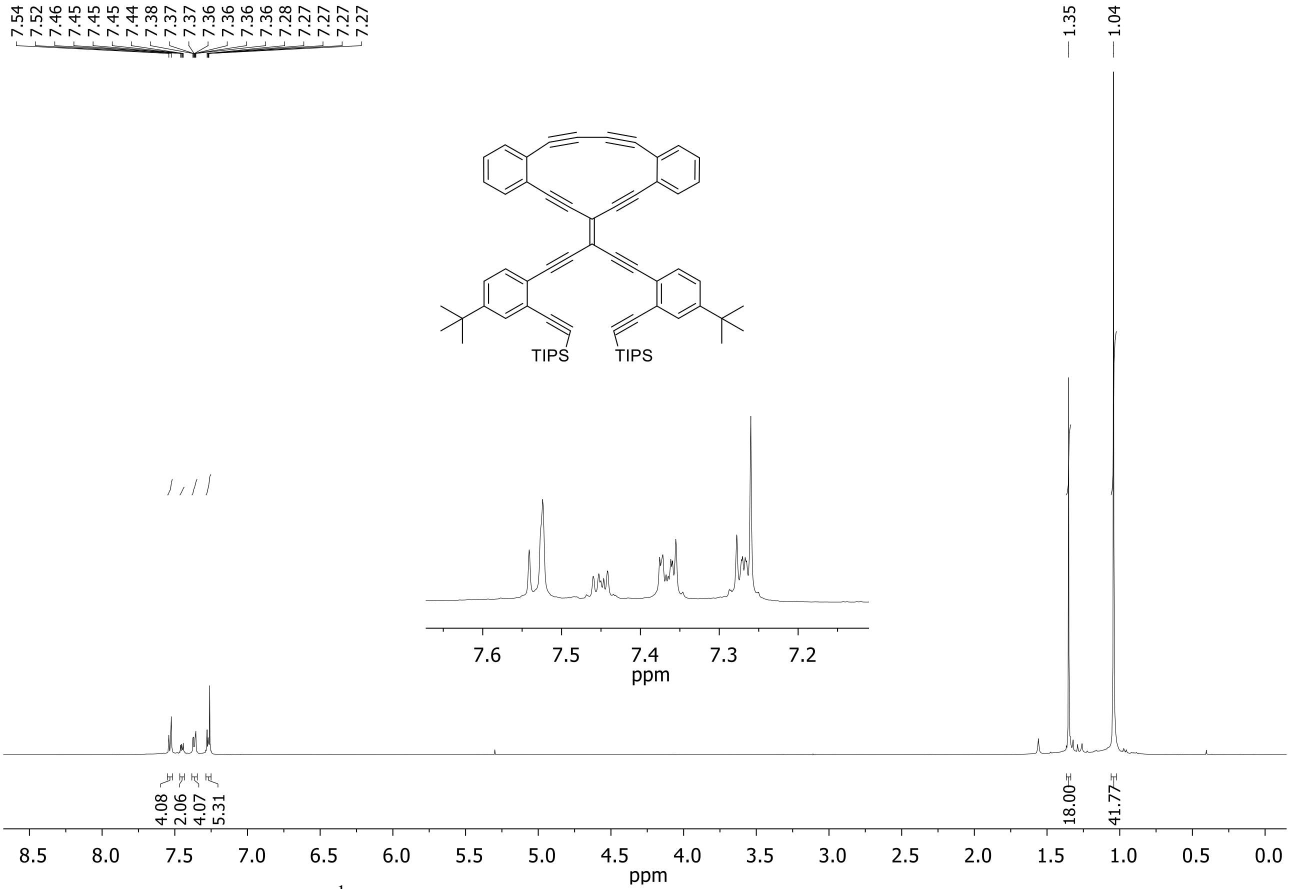

Supplementary Figure 33. ^{1}H-NMR spectrum of **13** in $CDCl_3$ (500 MHz).

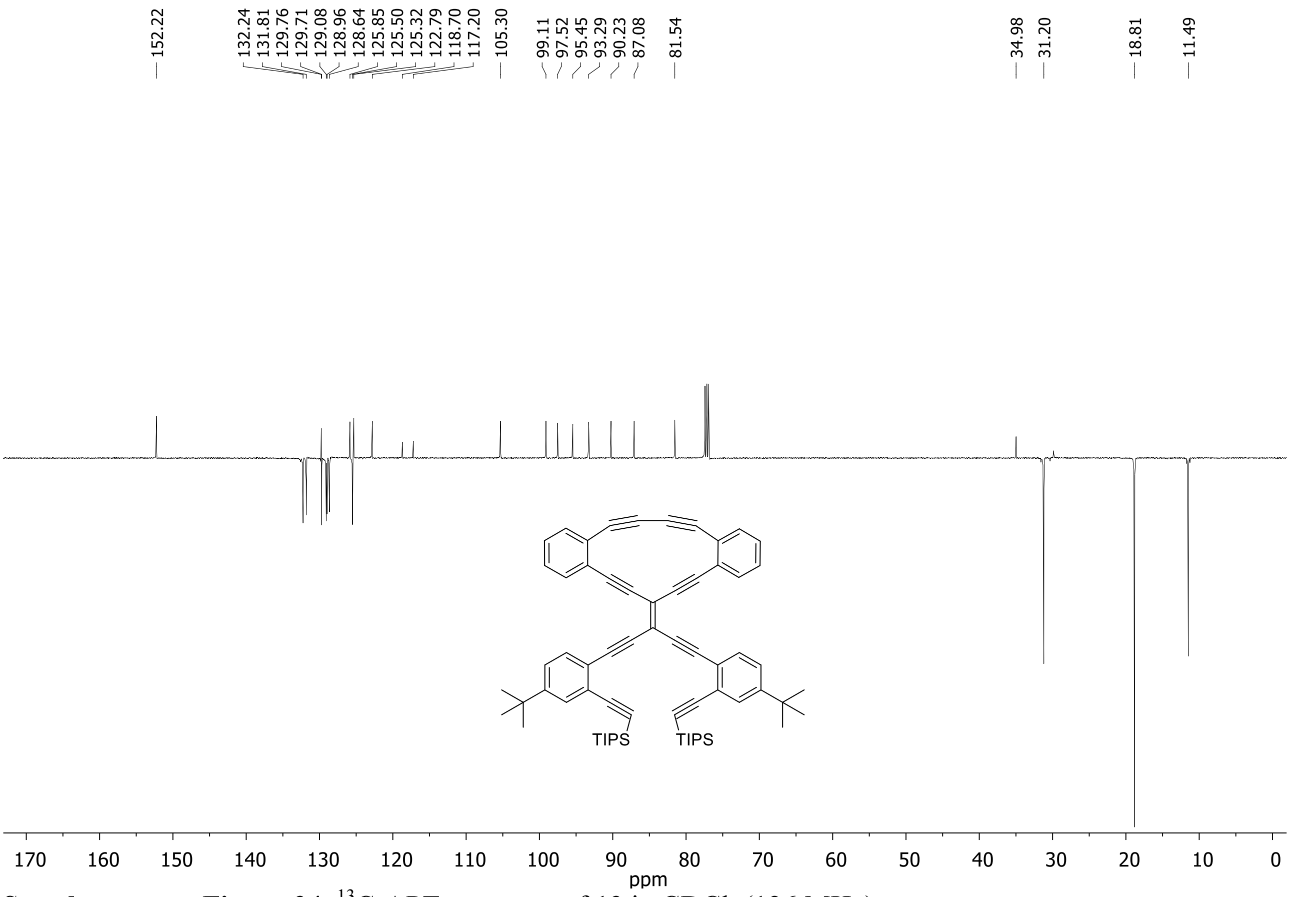

Supplementary Figure 34. ^{13}C-APT spectrum of **13** in $CDCl_3$ (126 MHz).

Compound **14**

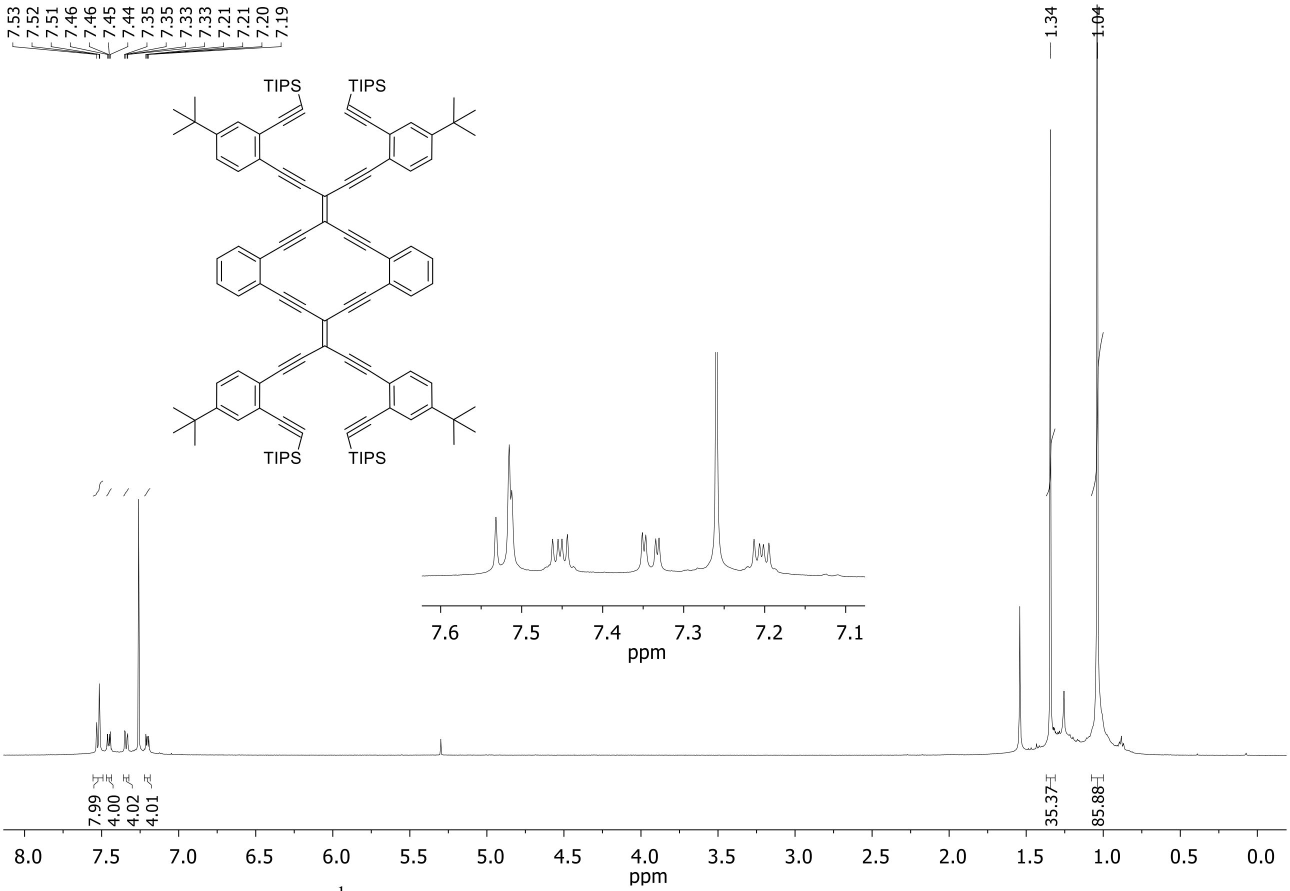

Supplementary Figure 35. ^{1}H-NMR spectrum of **14** in $CDCl_3$ (500 MHz).

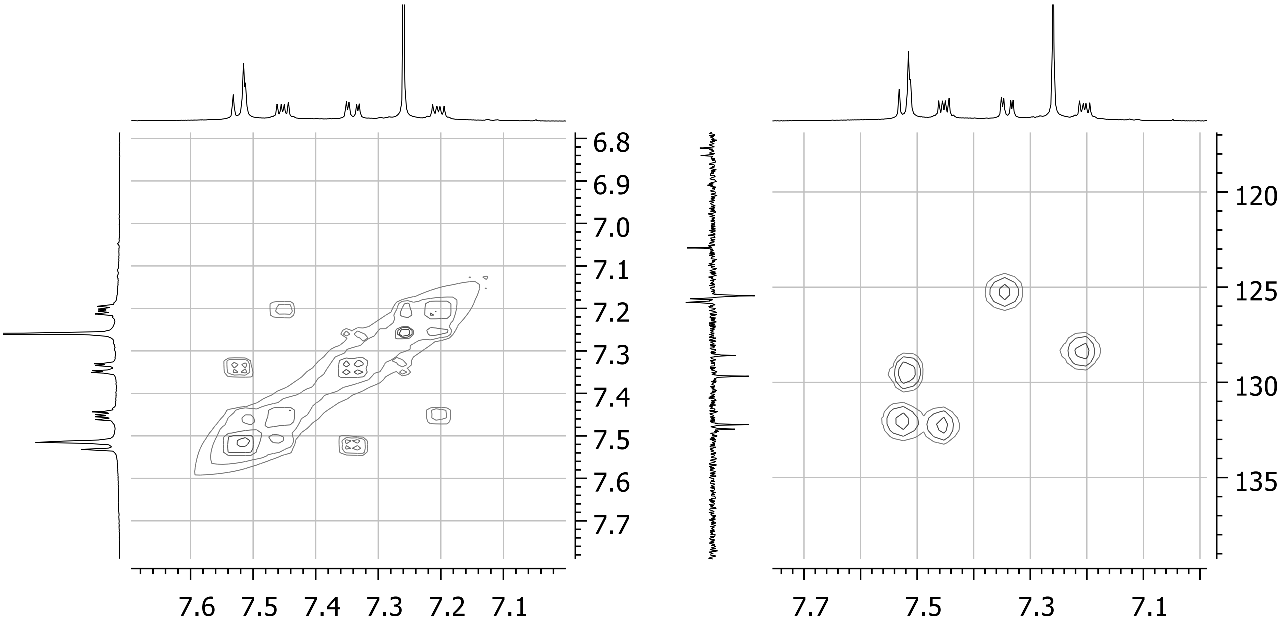

Supplementary Figure 36. COSY and (right) ^{1}H / ^{13}C-APT HSQC spectra of **14** in $CDCl_3$ (500 / 126 MHz).

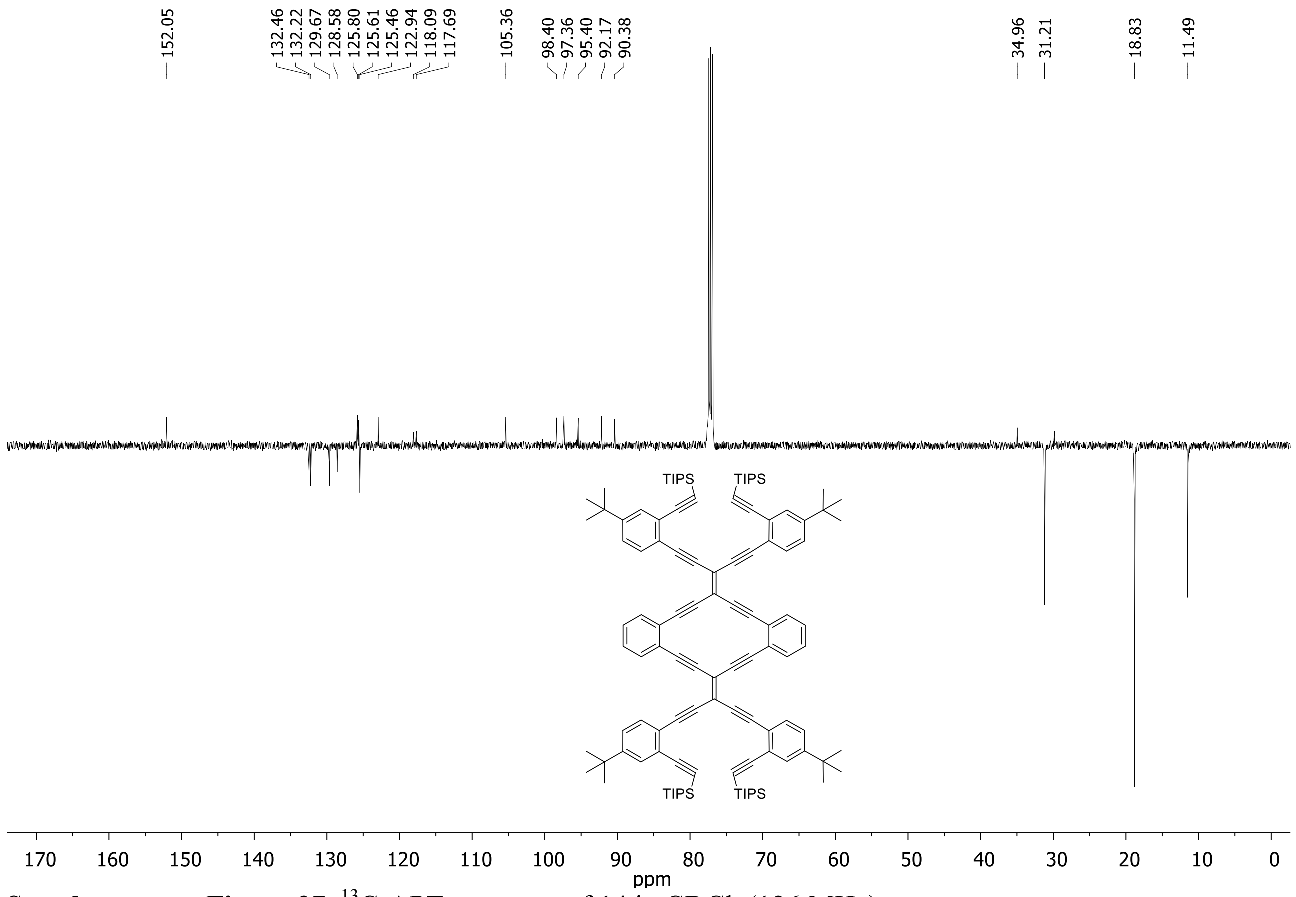

Supplementary Figure 37. ^{13}C-APT spectrum of **14** in $CDCl_3$ (126 MHz).

2-(Trimethylsilylethynyl)(triethylsilylethynyl)benzene (S9)

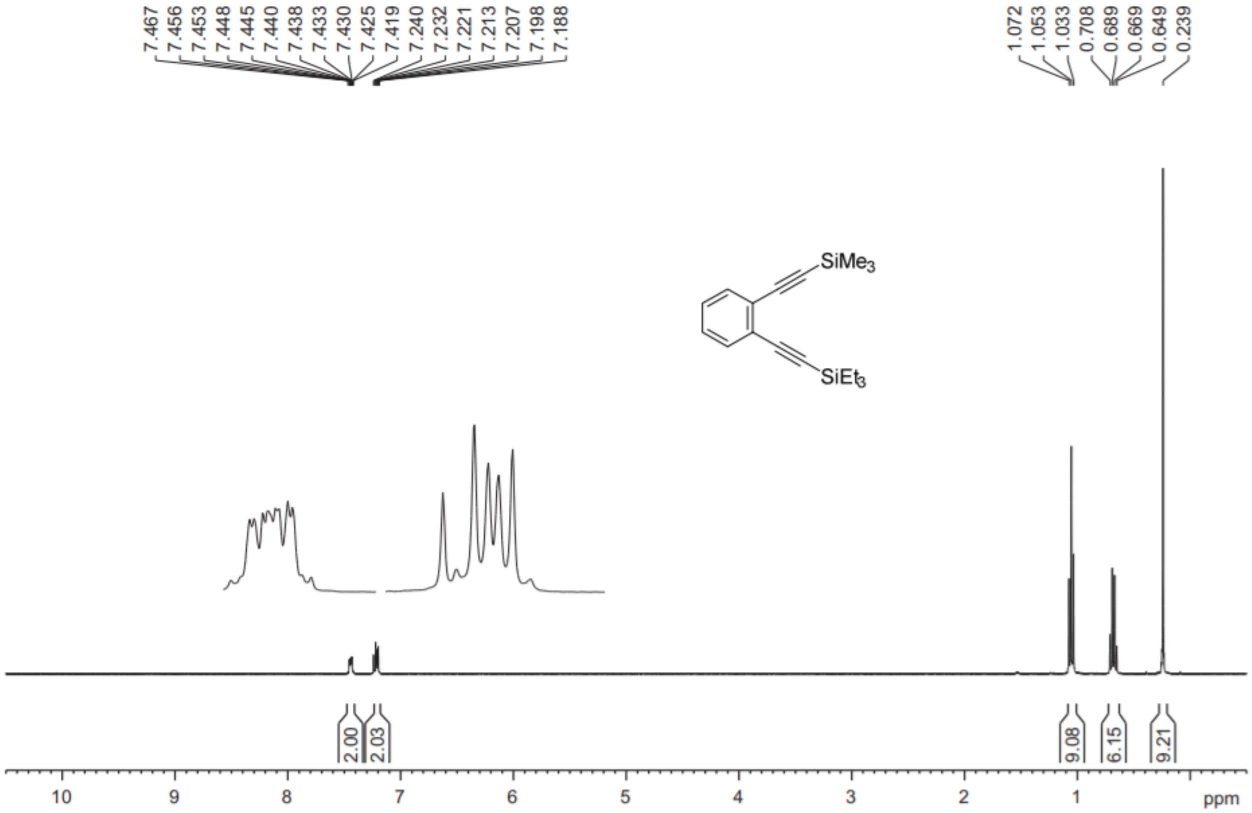

Supplementary Figure 38. ^{1}H-NMR spectrum of **S9** in $CDCl_3$ (300 MHz).

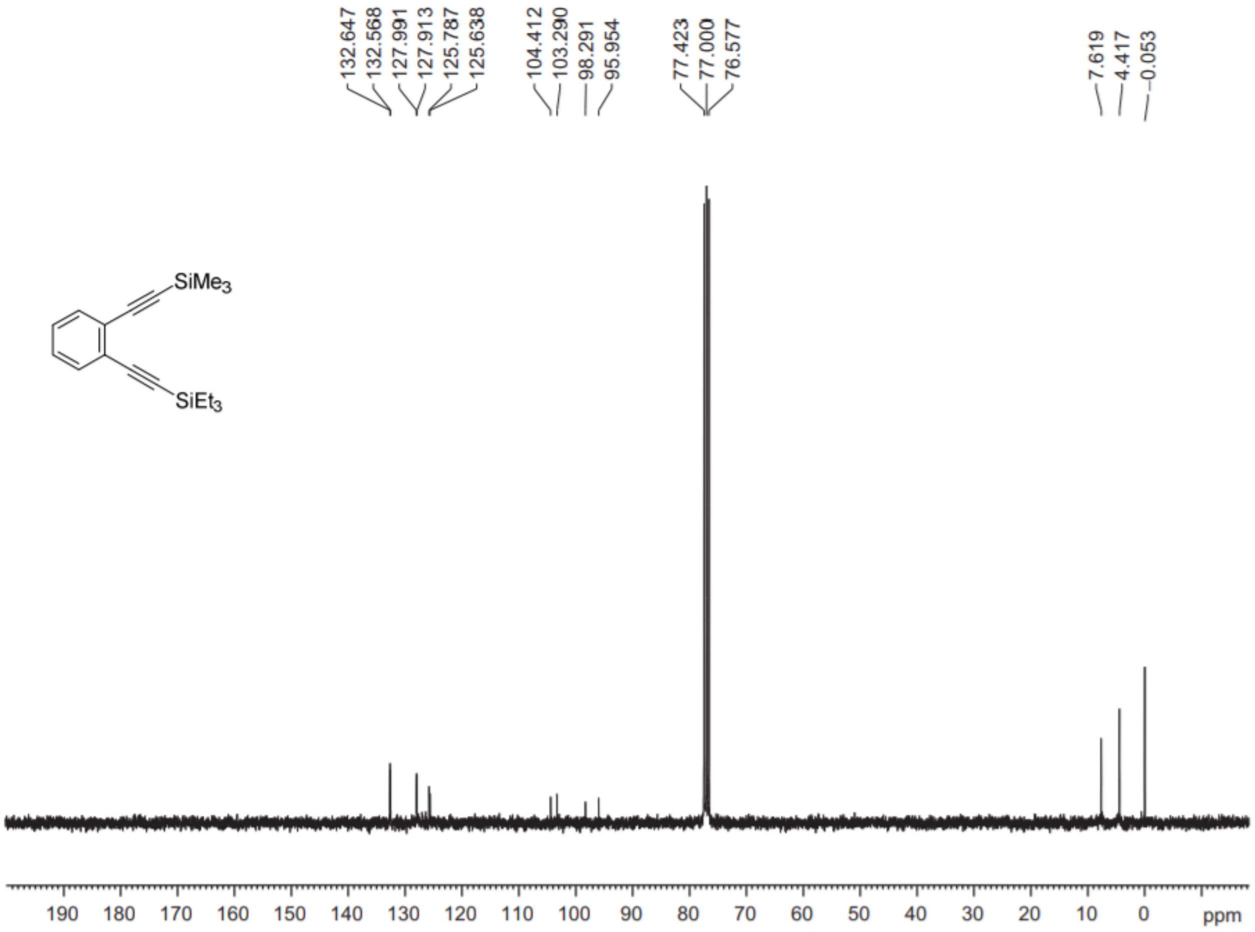

Supplementary Figure 39. ^{13}C spectrum of **S9** in $CDCl_3$ (75 MHz).

2-(Trimethylsilylethynyl)ethynylbenzene (S10)

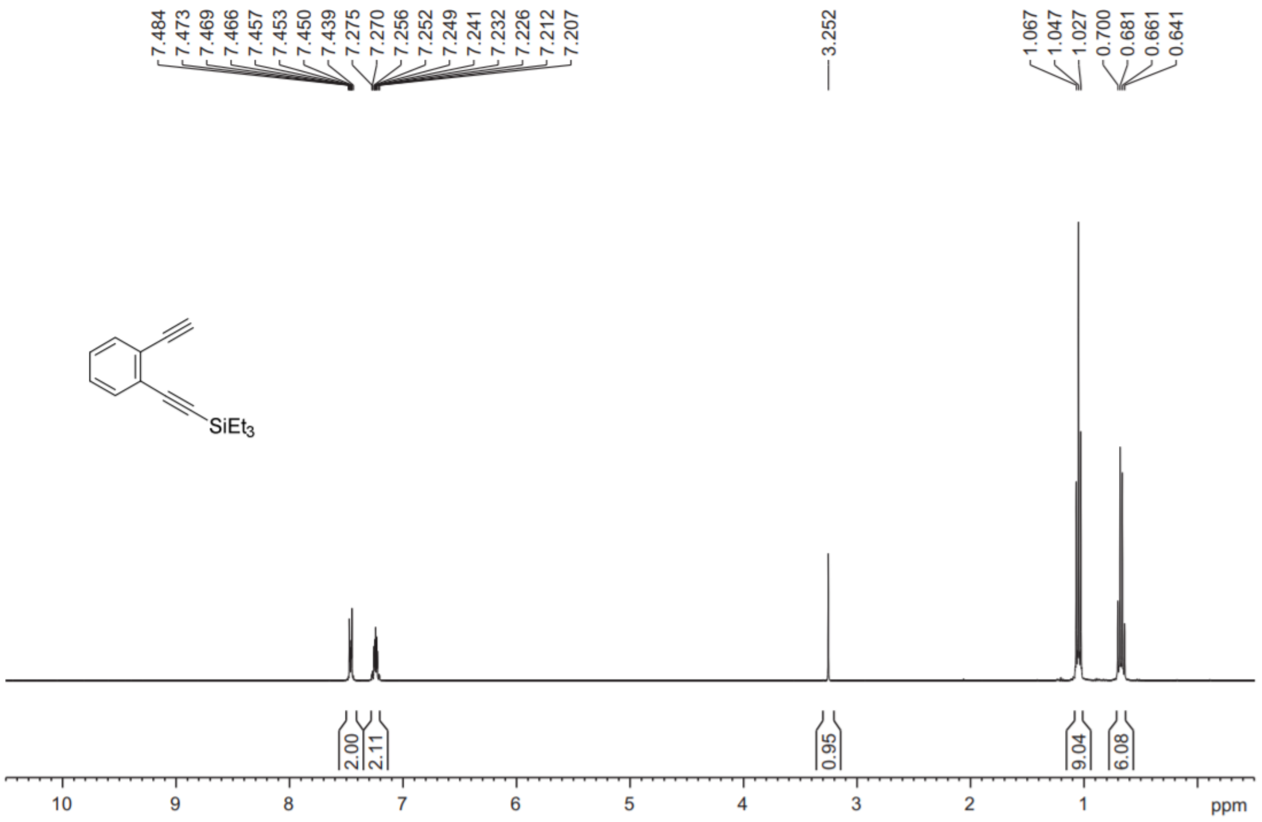

Supplementary Figure 40. ^{1}H-NMR spectrum of **S10** in $CDCl_3$ (300 MHz).

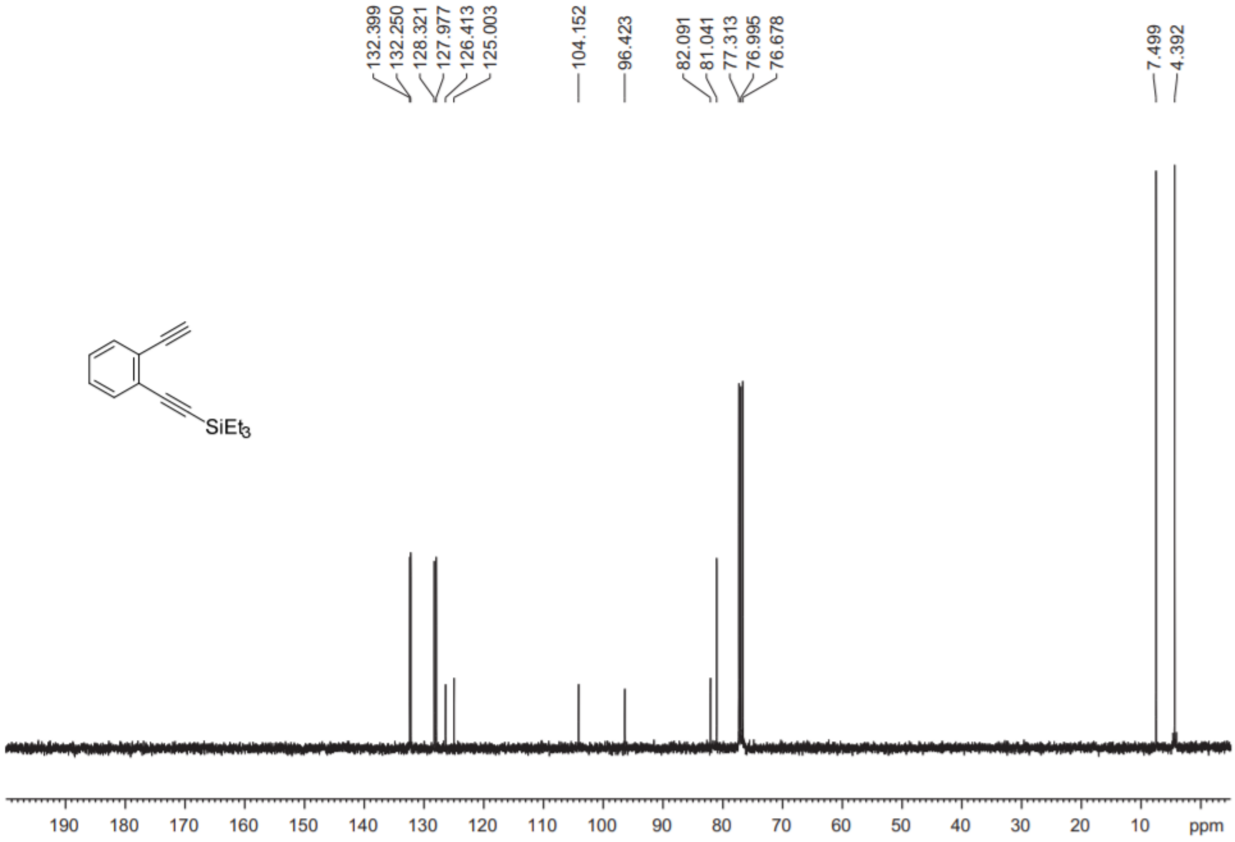

Supplementary Figure 41. ^{13}C-NMR spectrum of **S10** in $CDCl_3$ (75 MHz).

Compound **S11**

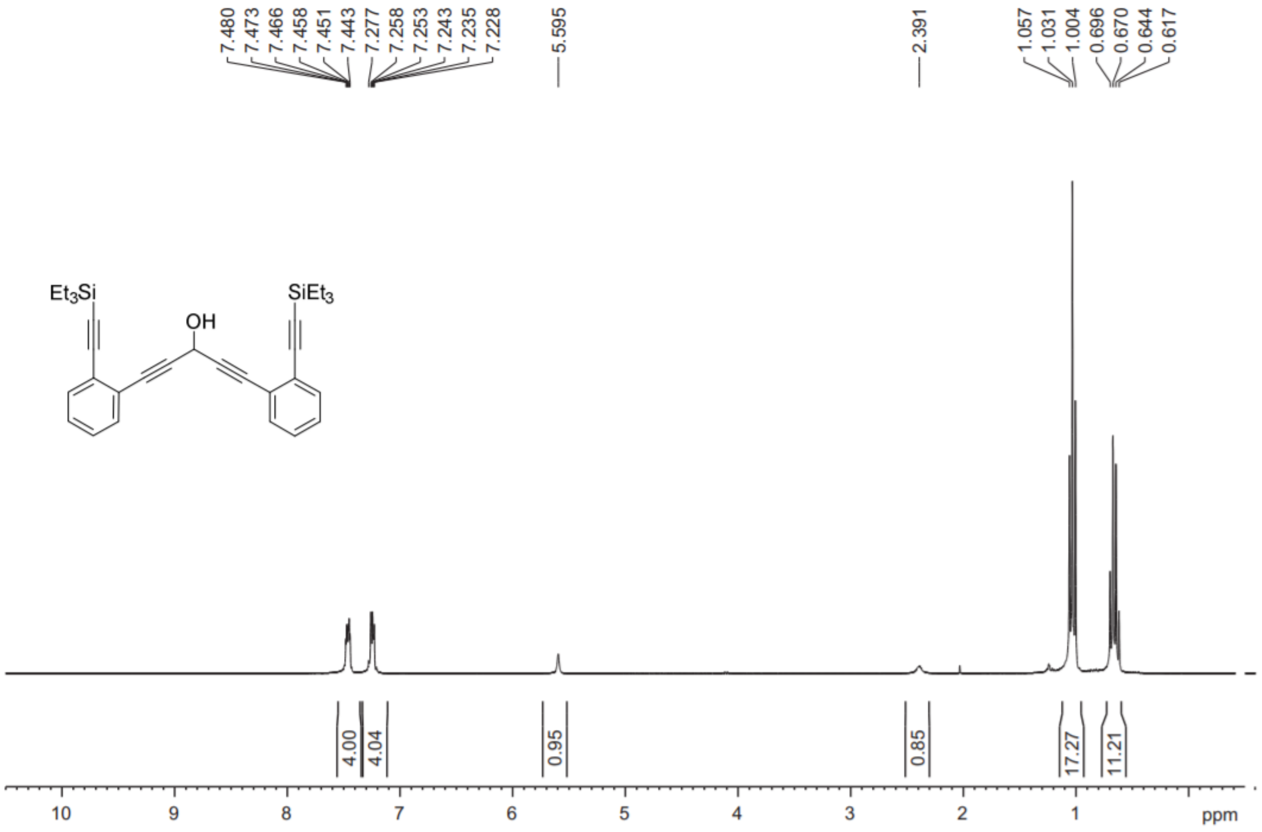

Supplementary Figure 42. ^{1}H-NMR spectrum of **S11** in $CDCl_3$ (300 MHz).

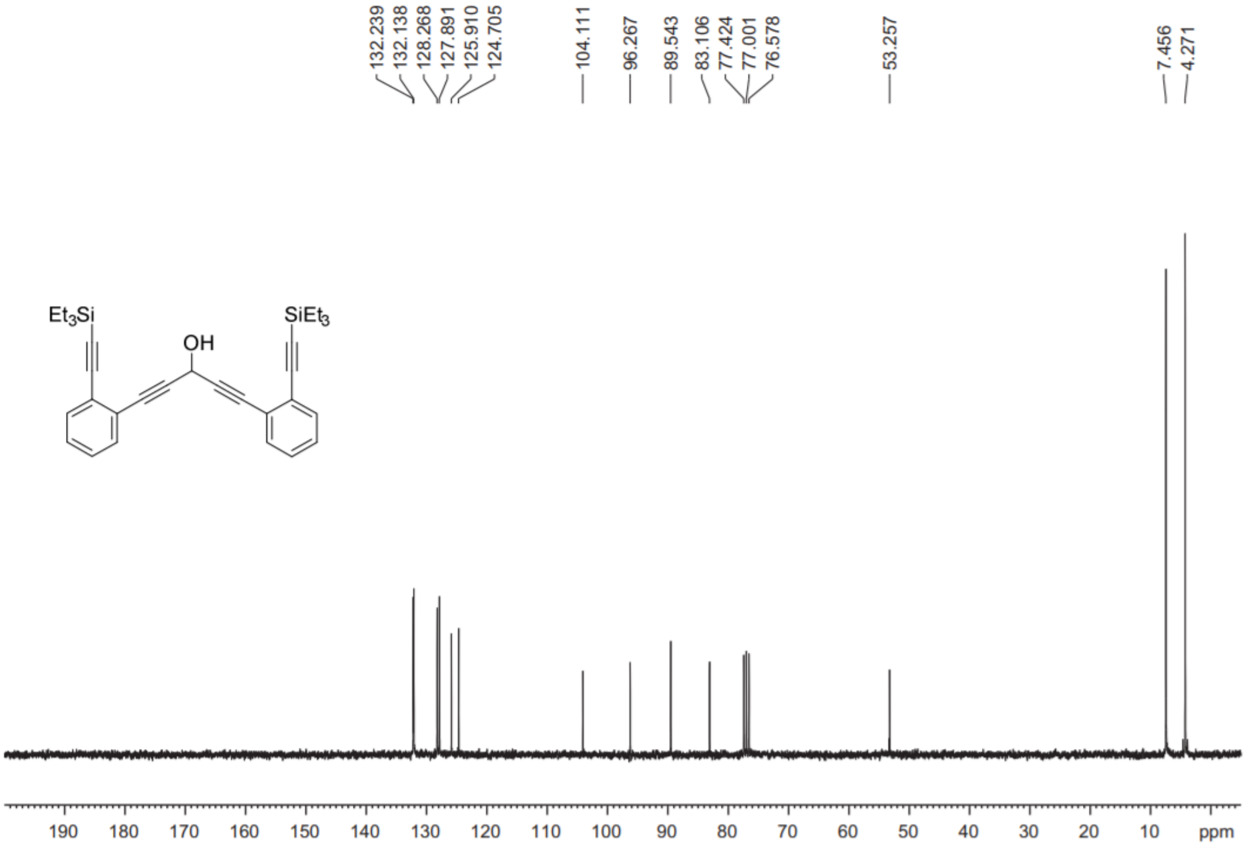

Supplementary Figure 43. ^{13}C-NMR spectrum of **S11** in $CDCl_3$ (75 MHz).

Compound **S16**

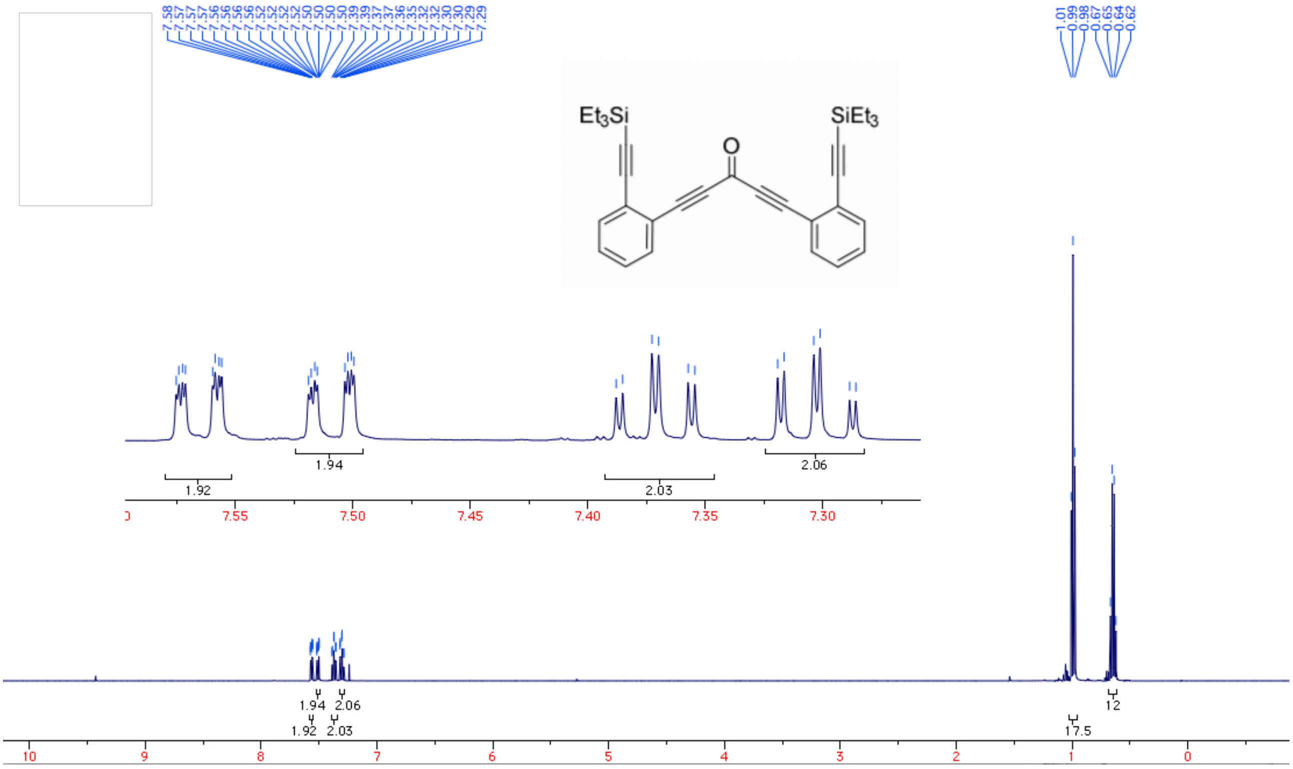

Supplementary Figure 44. ^{1}H-NMR spectrum of **S16** in $CDCl_3$ (500 MHz).

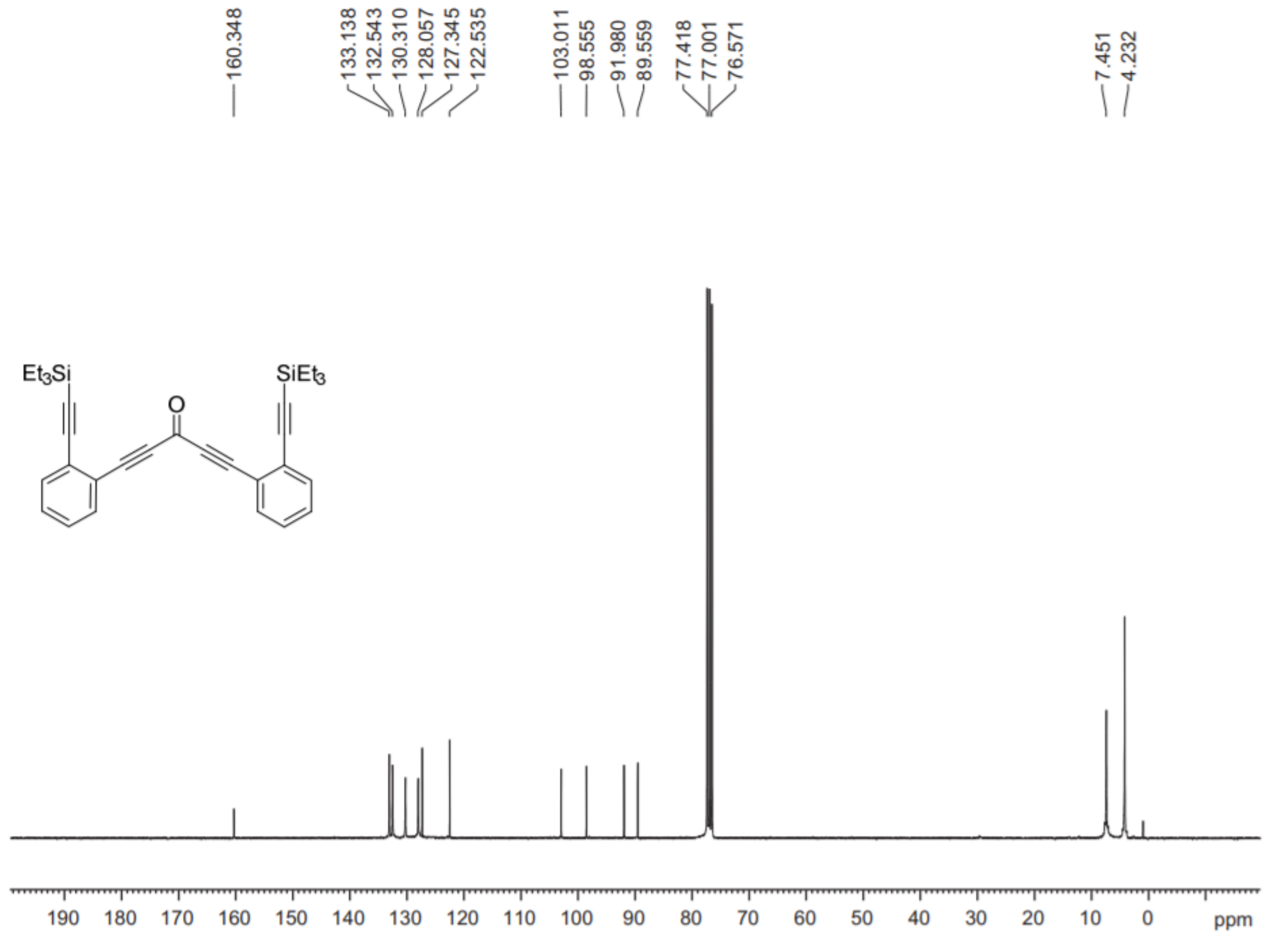

Supplementary Figure 45. ^{13}C-NMR spectrum of **S16** in $CDCl_3$ (75 MHz).

Compound **S17**

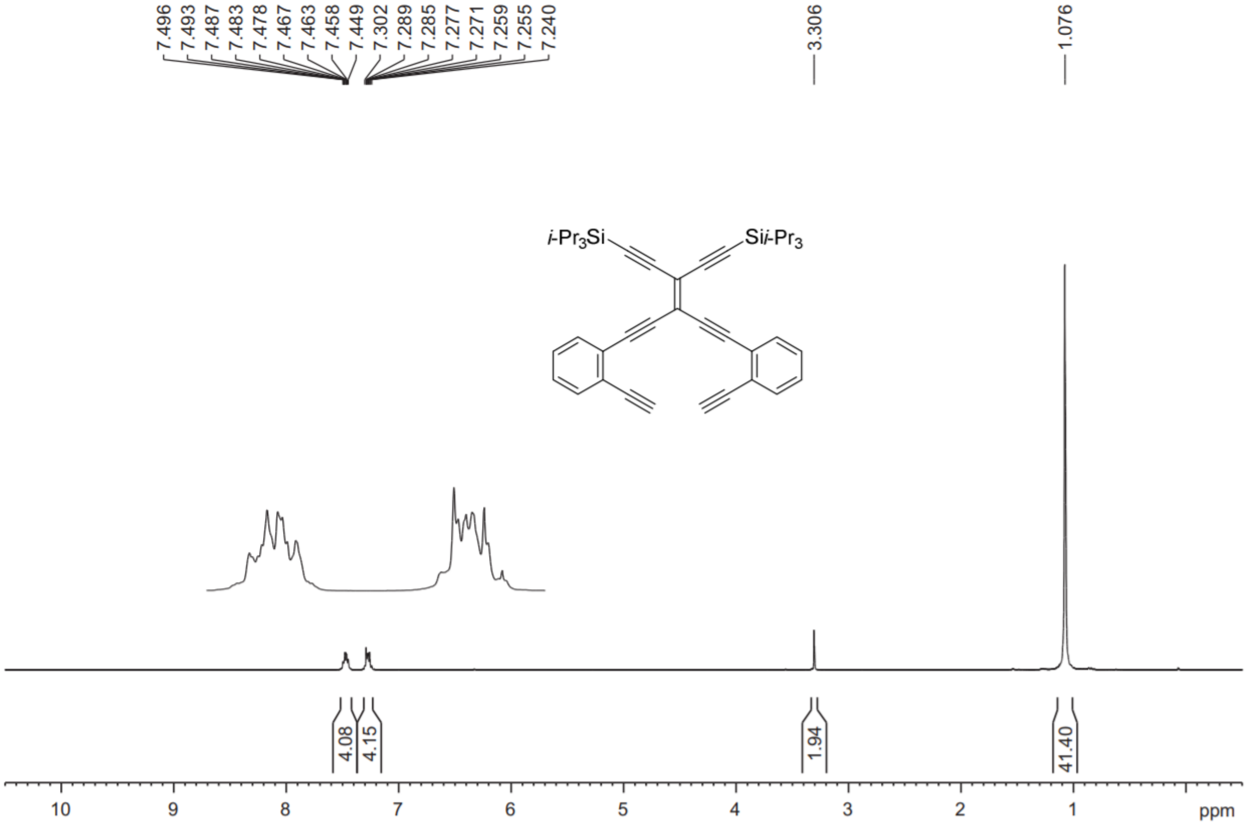

Supplementary Figure 46. ^{1}H-NMR spectrum of **S17** in $CDCl_3$ (300 MHz).

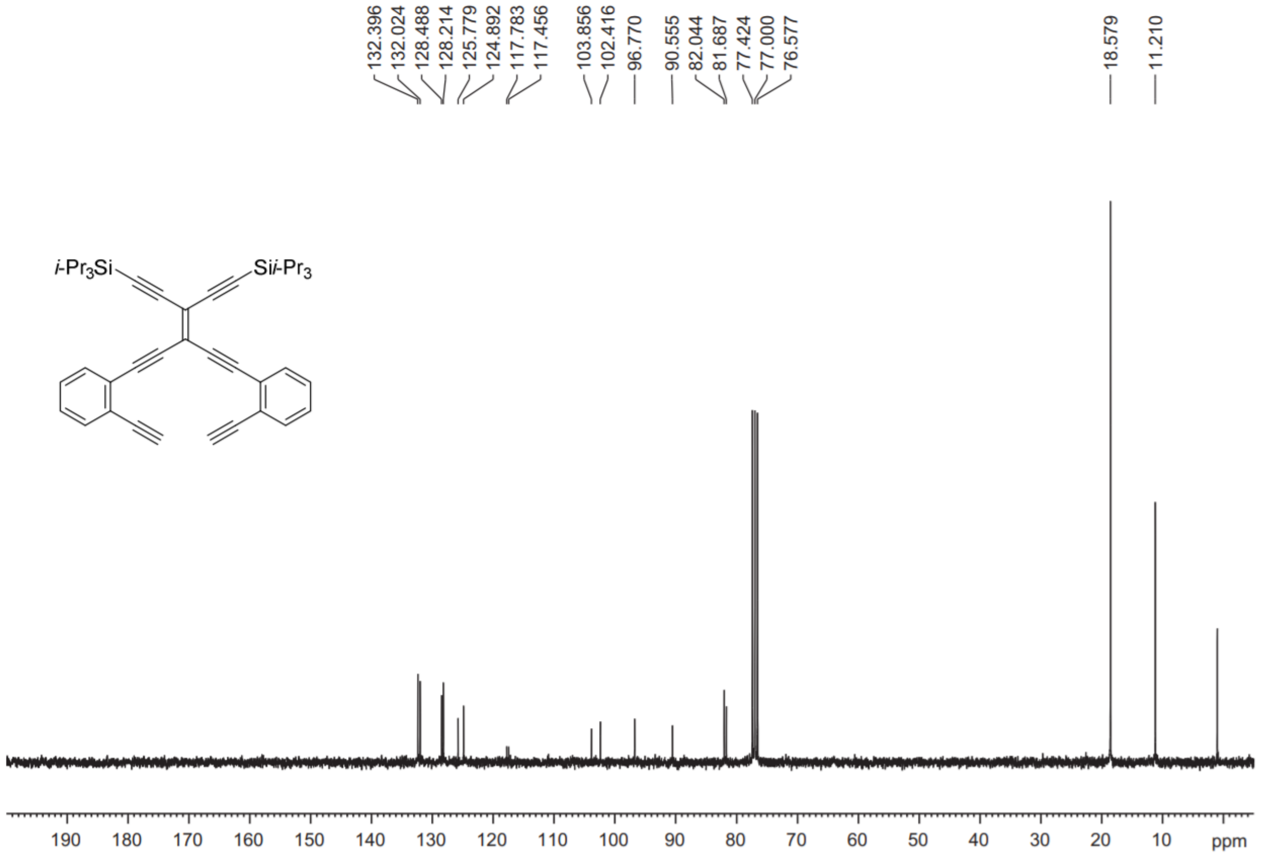

Supplementary Figure 47. ^{13}C-NMR spectrum of **S17** in $CDCl_3$ (75 MHz).

Compound **S21**

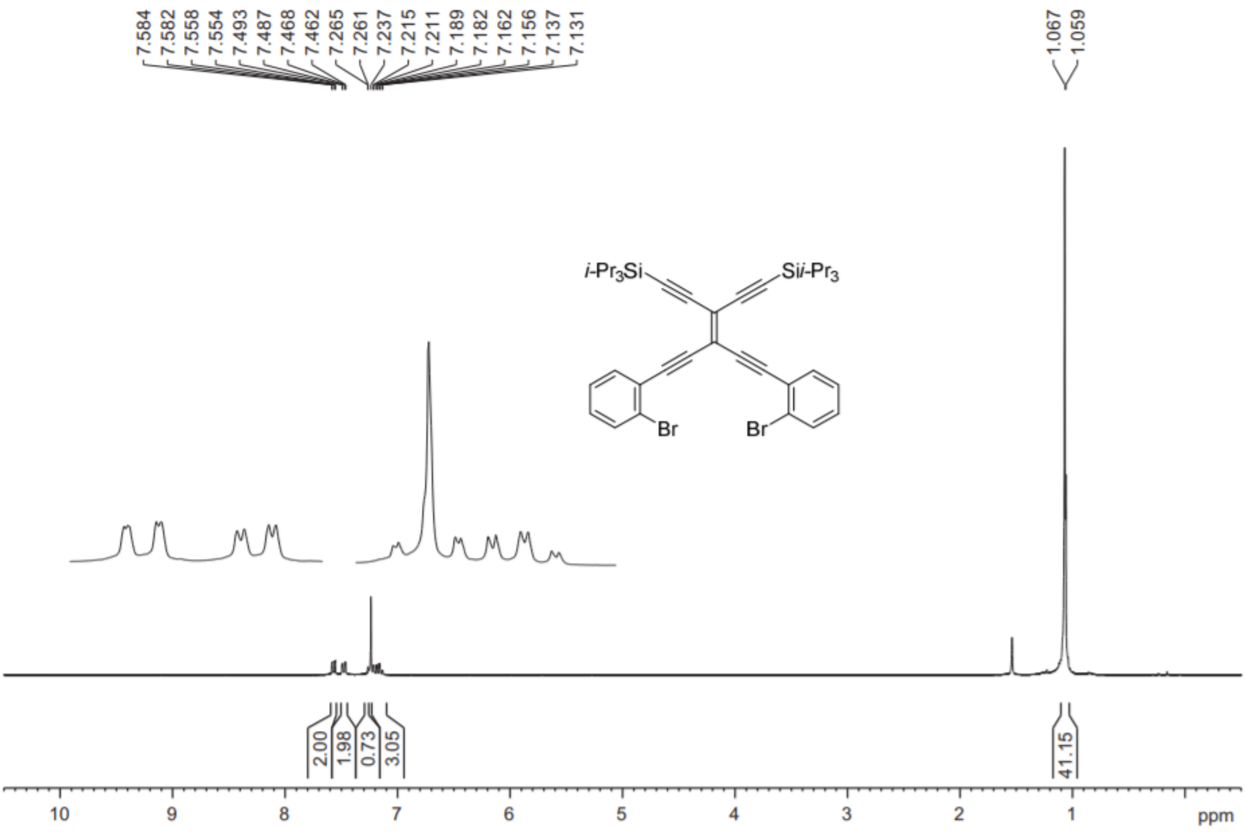

Supplementary Figure 48. ^{1}H-NMR spectrum of **S21** in $CDCl_3$ (300 MHz).

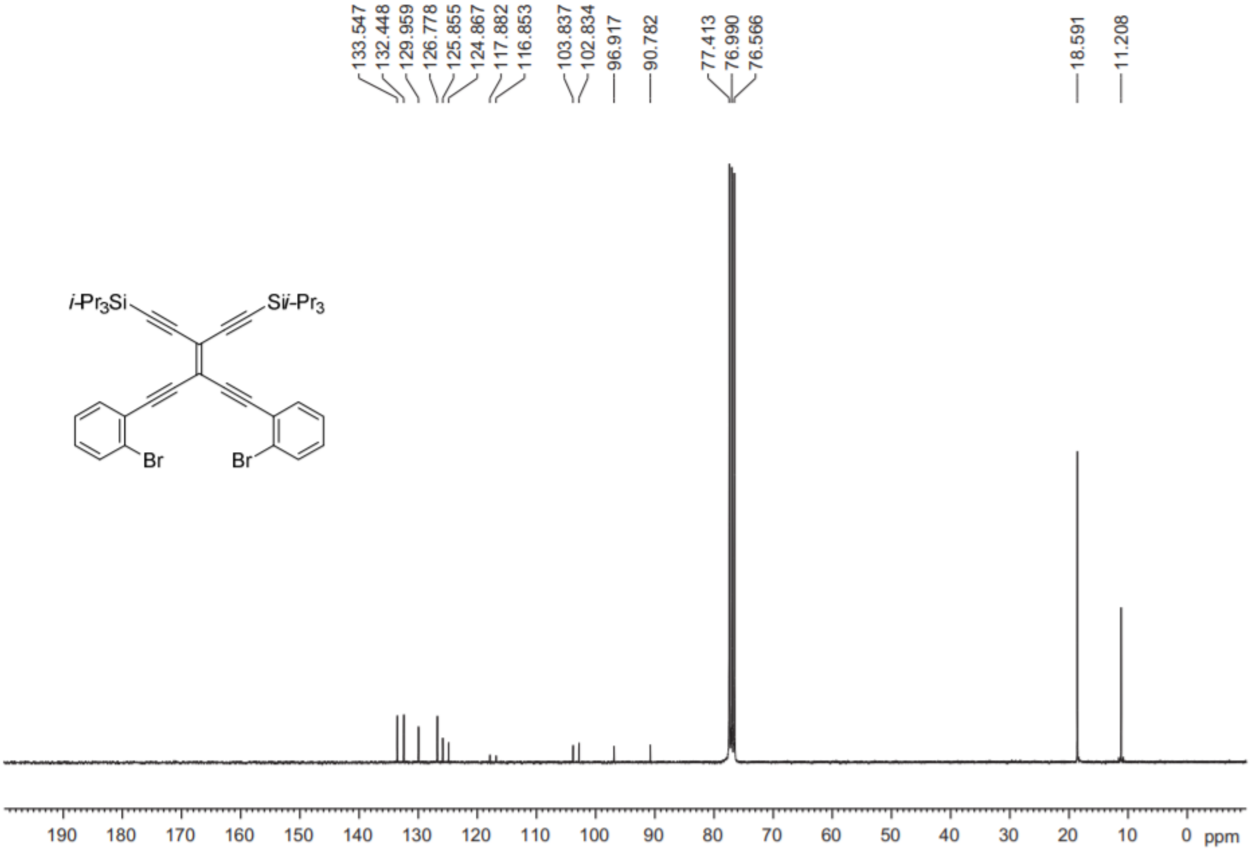

Supplementary Figure 49. ^{13}C-NMR spectrum of **S21** in $CDCl_3$ (75 MHz).

Compound **S22**

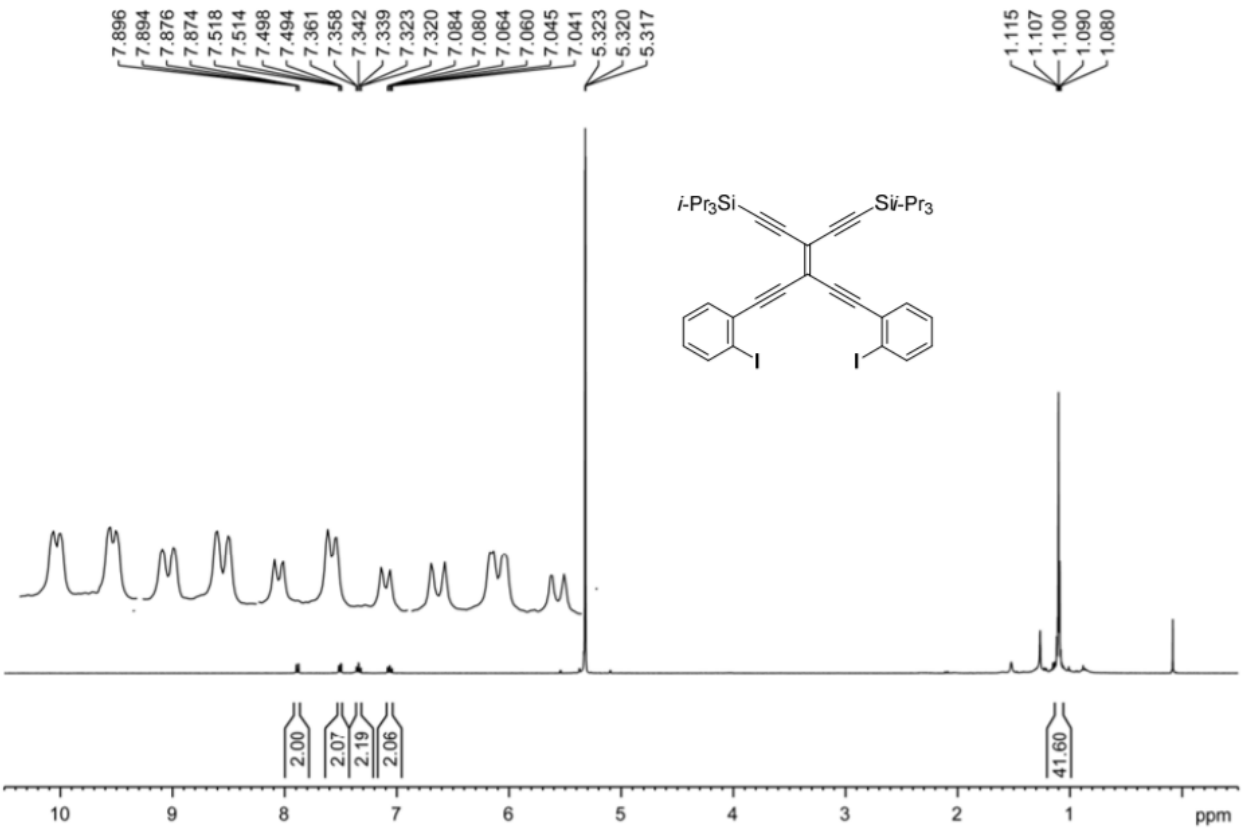

Supplementary Figure 50. ^{1}H-NMR spectrum of **S22** in $CDCl_3$ (300 MHz).

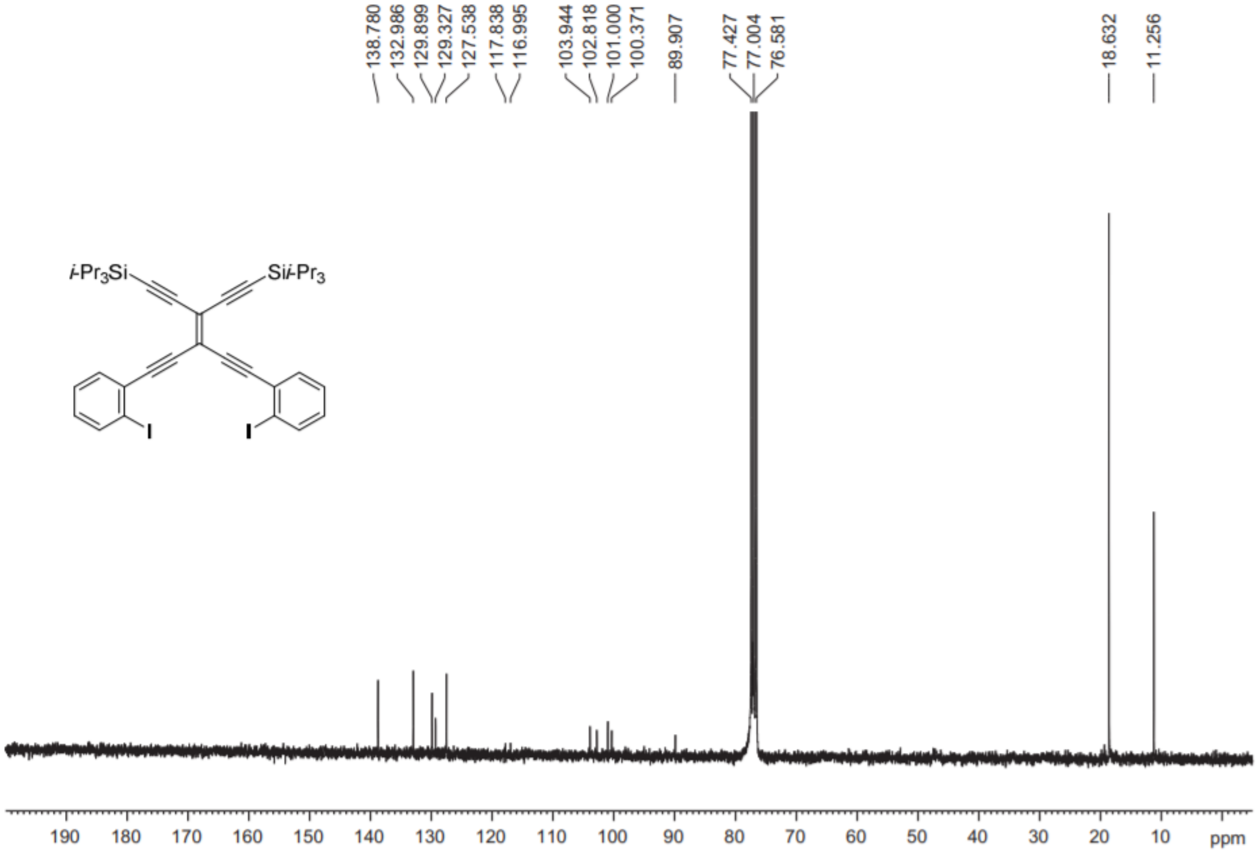

Supplementary Figure 51. ^{13}C-NMR spectrum of **S22** in $CDCl_3$ (75 MHz).

Compound **S23**

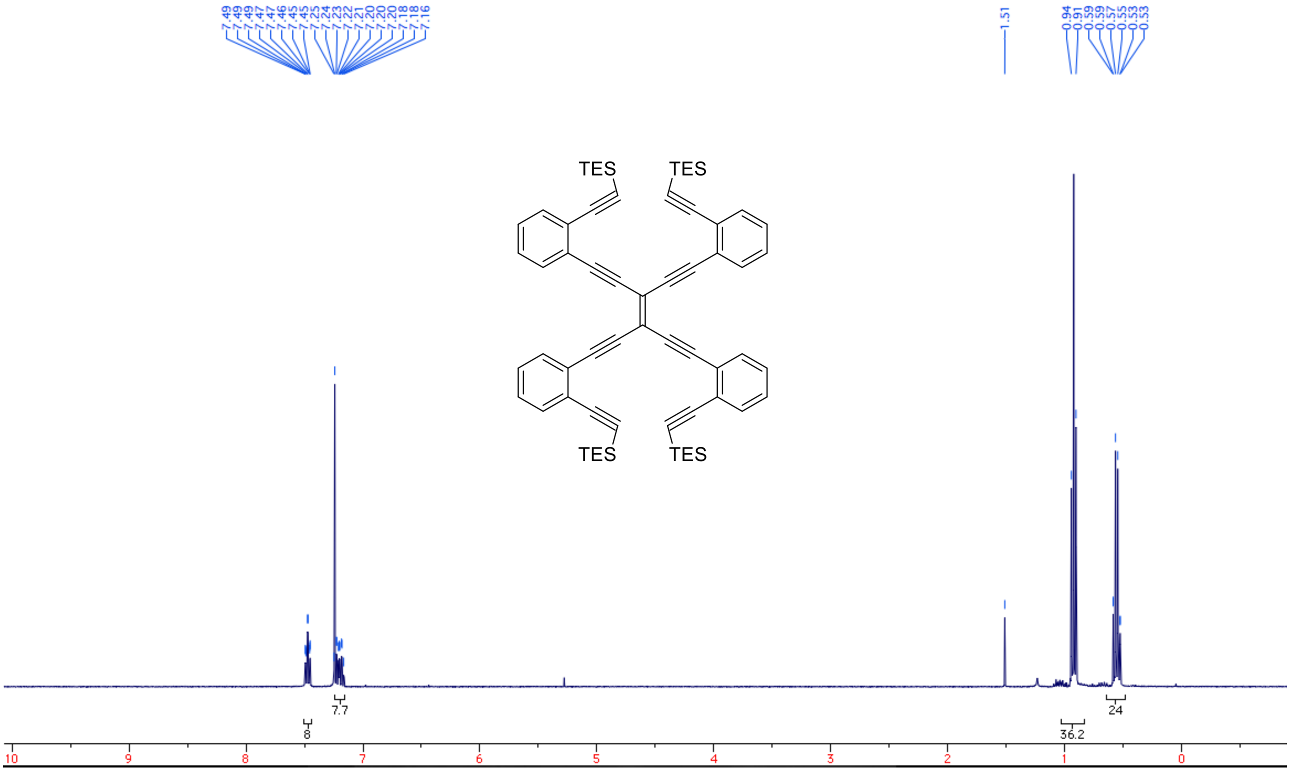

Supplementary Figure 52. ^{1}H-NMR spectrum of **S23** in $CDCl_3$ (300 MHz).

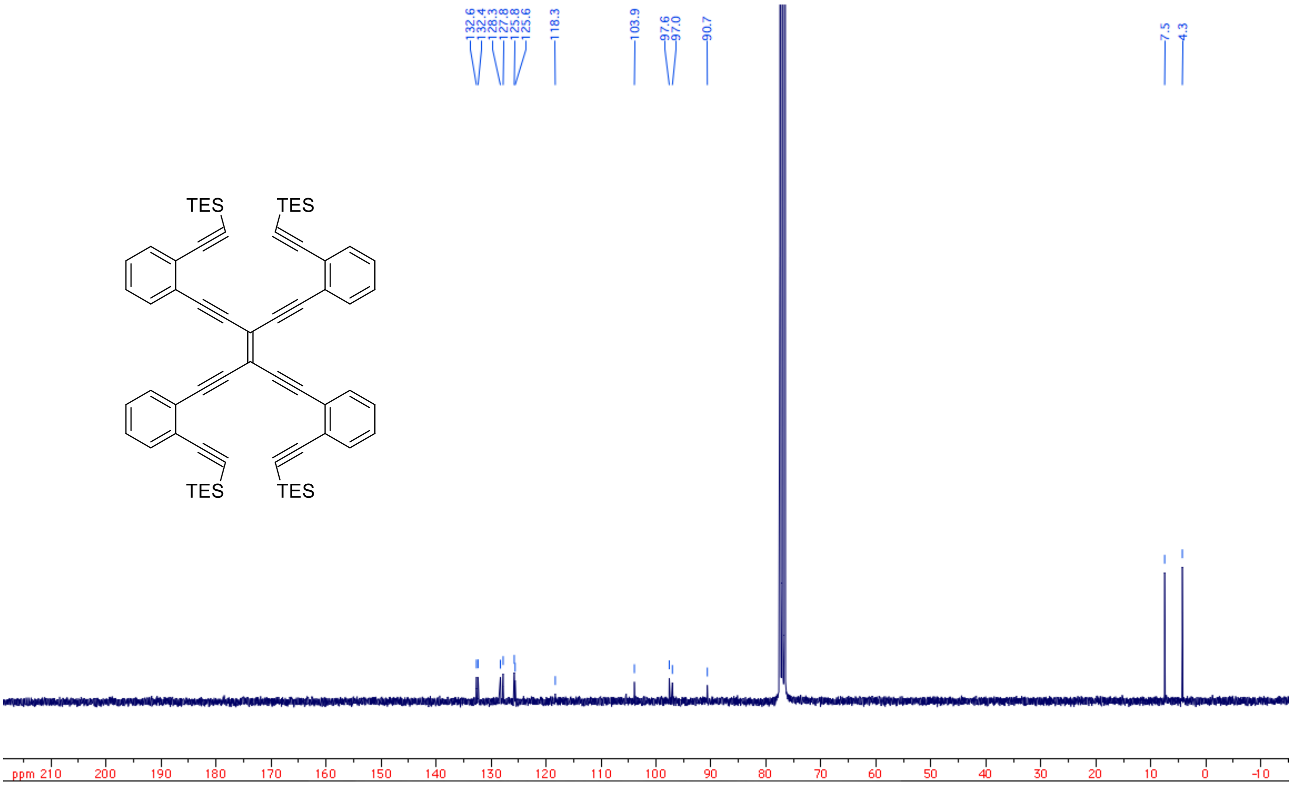

Supplementary Figure 53. ^{13}C-NMR spectrum of **S23** in $CDCl_3$ (75 MHz).

Compound **S24**

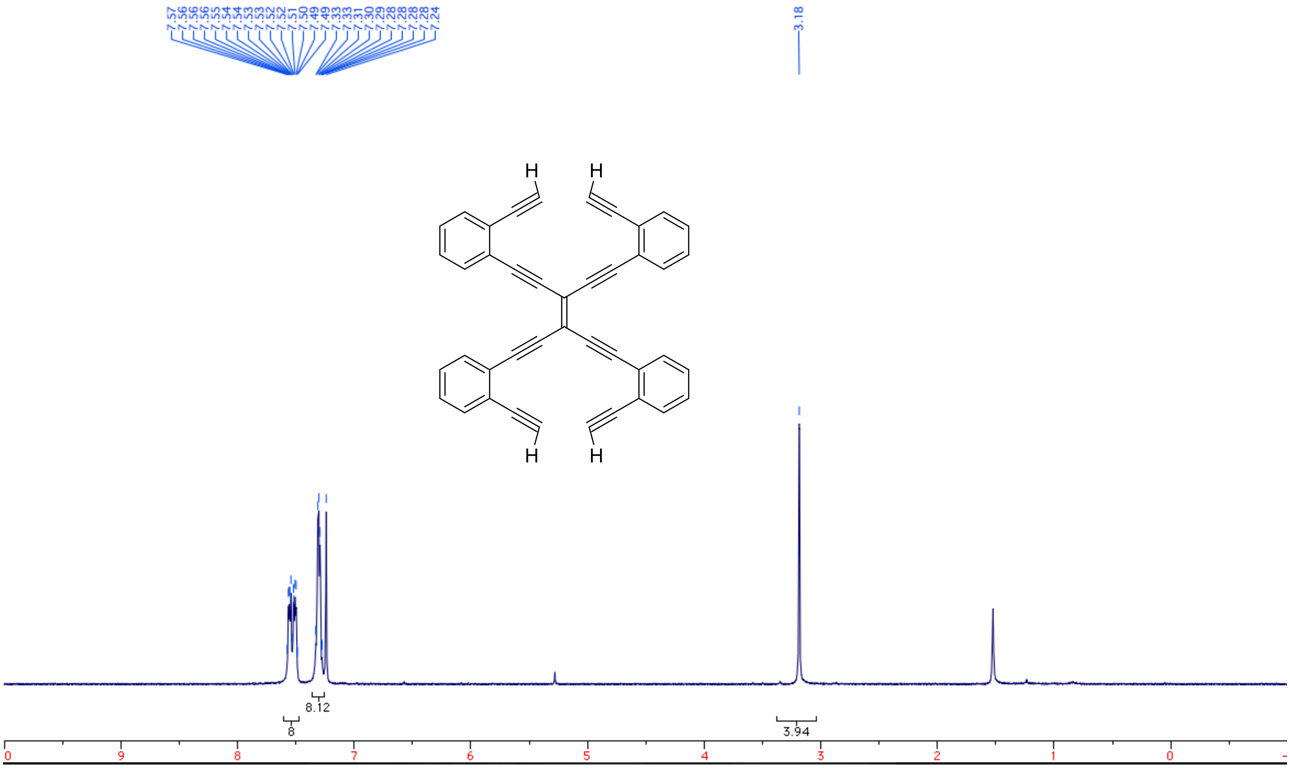

Supplementary Figure 54. ^{1}H-NMR spectrum of **S24** in $CDCl_3$ (300 MHz).

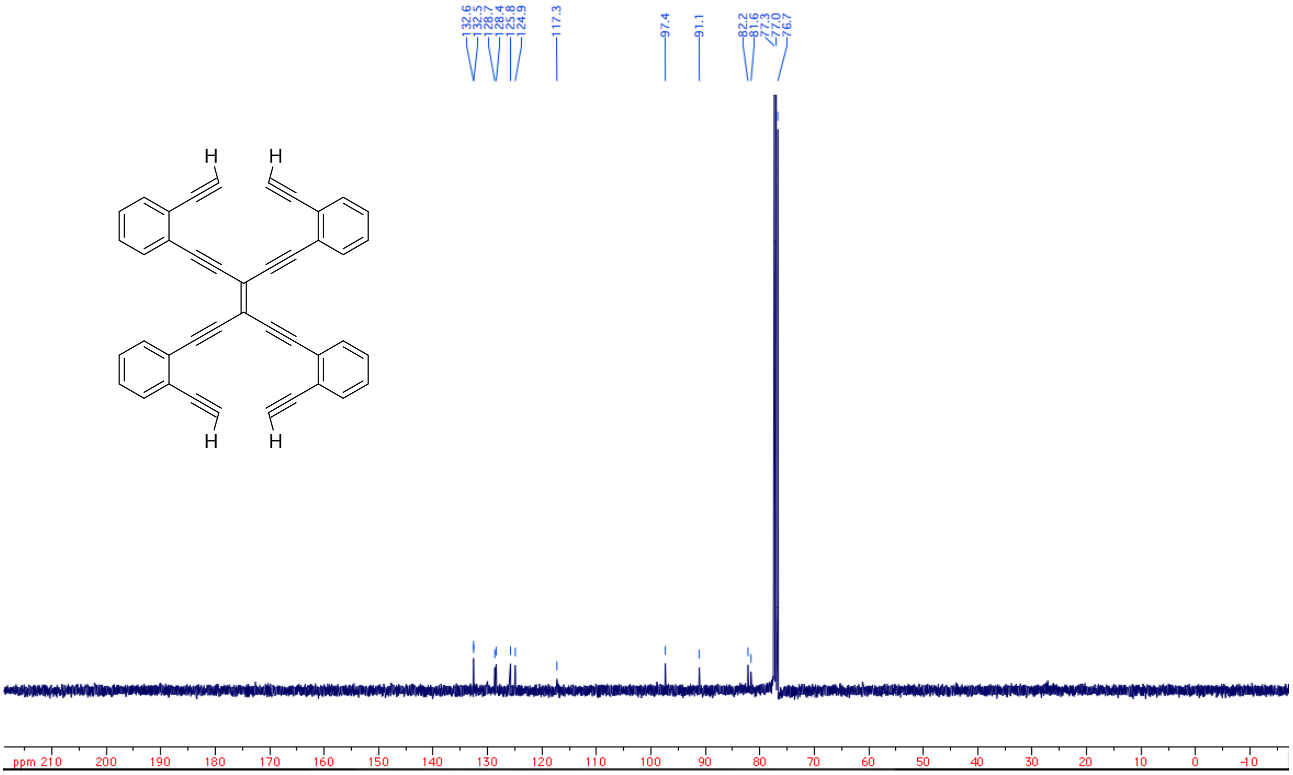

Supplementary Figure 55. ^{13}C-NMR spectrum of **S24** in $CDCl_3$ (75 MHz).

Mono-radiaannulene 4a

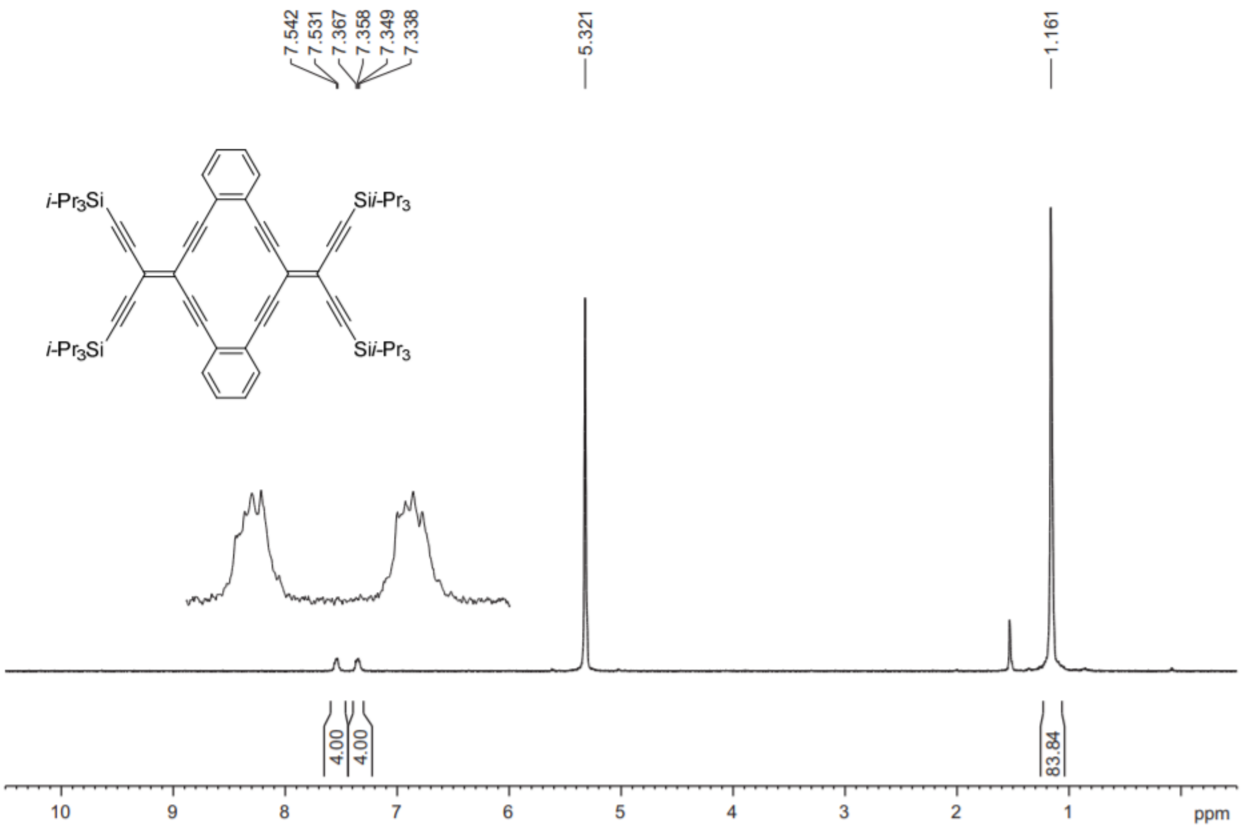

Supplementary Figure 56. ^{1}H-NMR spectrum of **4a** in CD_2Cl_2 (300 MHz).

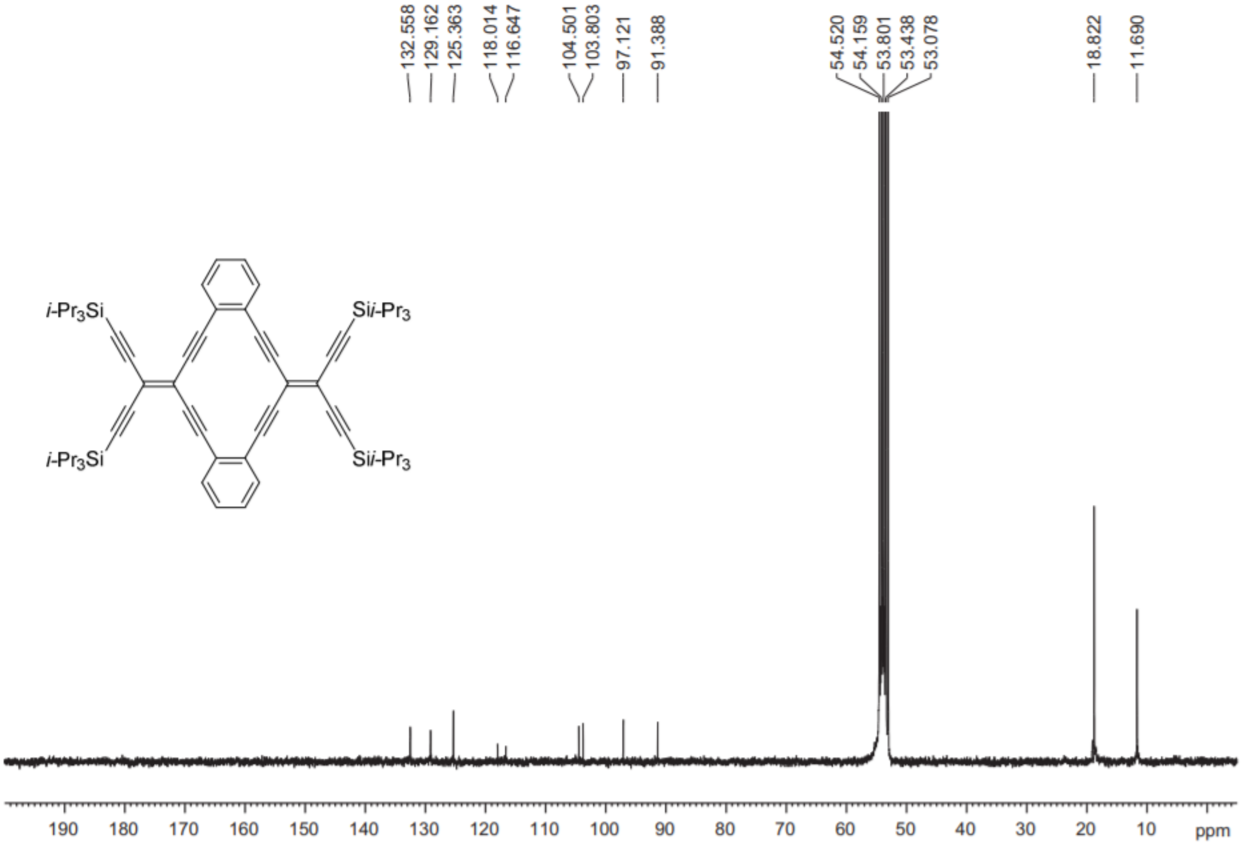

Supplementary Figure 57. ^{13}C-NMR spectrum of **4a** in CD_2Cl_2 (75 MHz).

Compound **4aSByproduct**

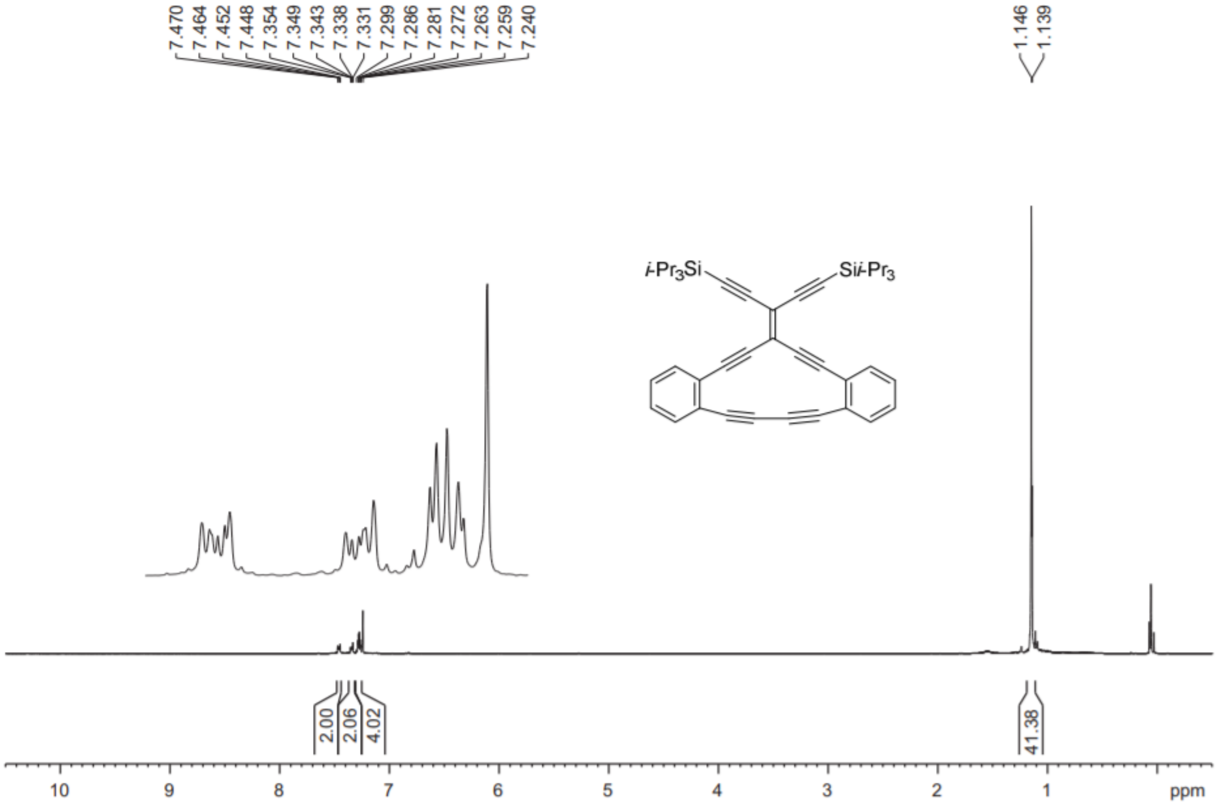

Supplementary Figure 58. ^{1}H-NMR spectrum of **4aSByproduct** in $CDCl_3$ (300 MHz).

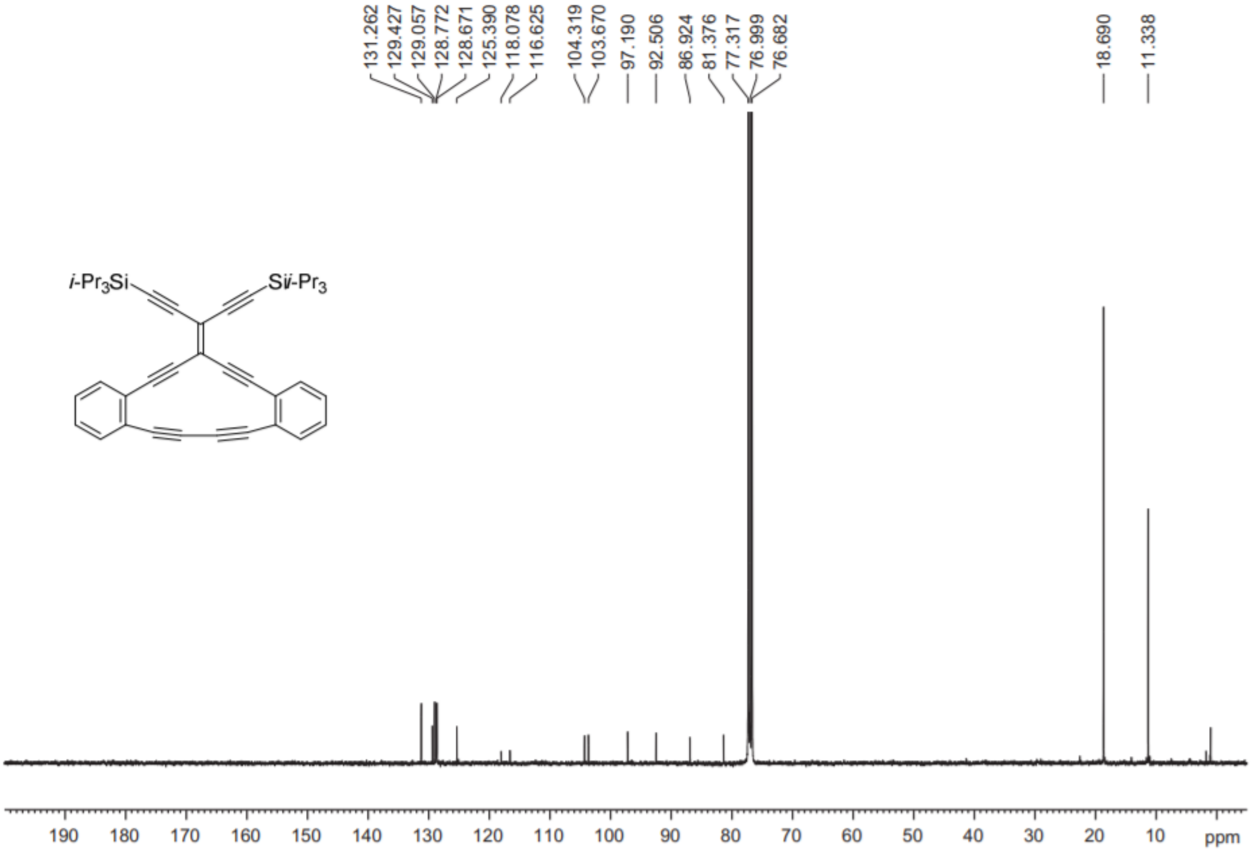

Supplementary Figure 59. ^{13}C-NMR spectrum of **4aSByproduct** in $CDCl_3$ (75 MHz).

Mono-radiaannulene 4b

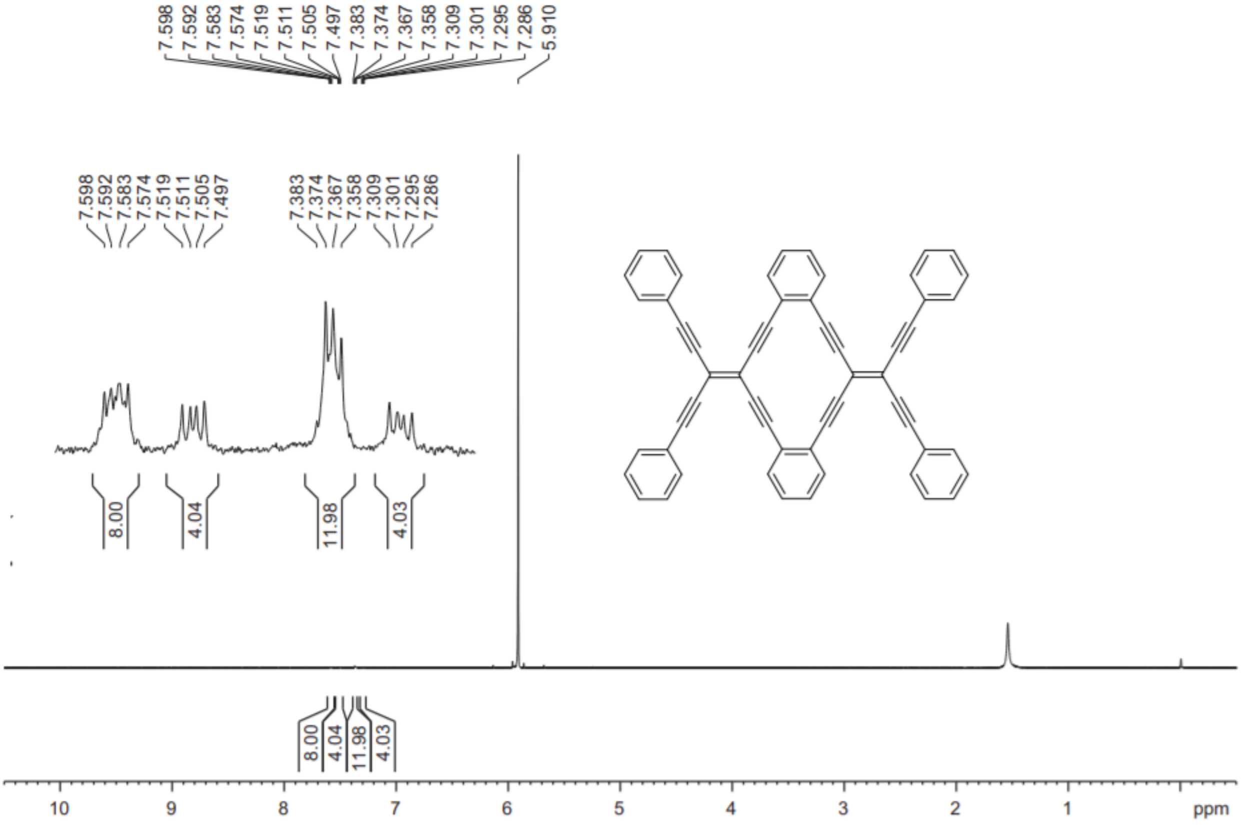

Supplementary Figure 60. ^{1}H-NMR spectrum of **4b** in $C_2D_2Cl_4$ (400 MHz).

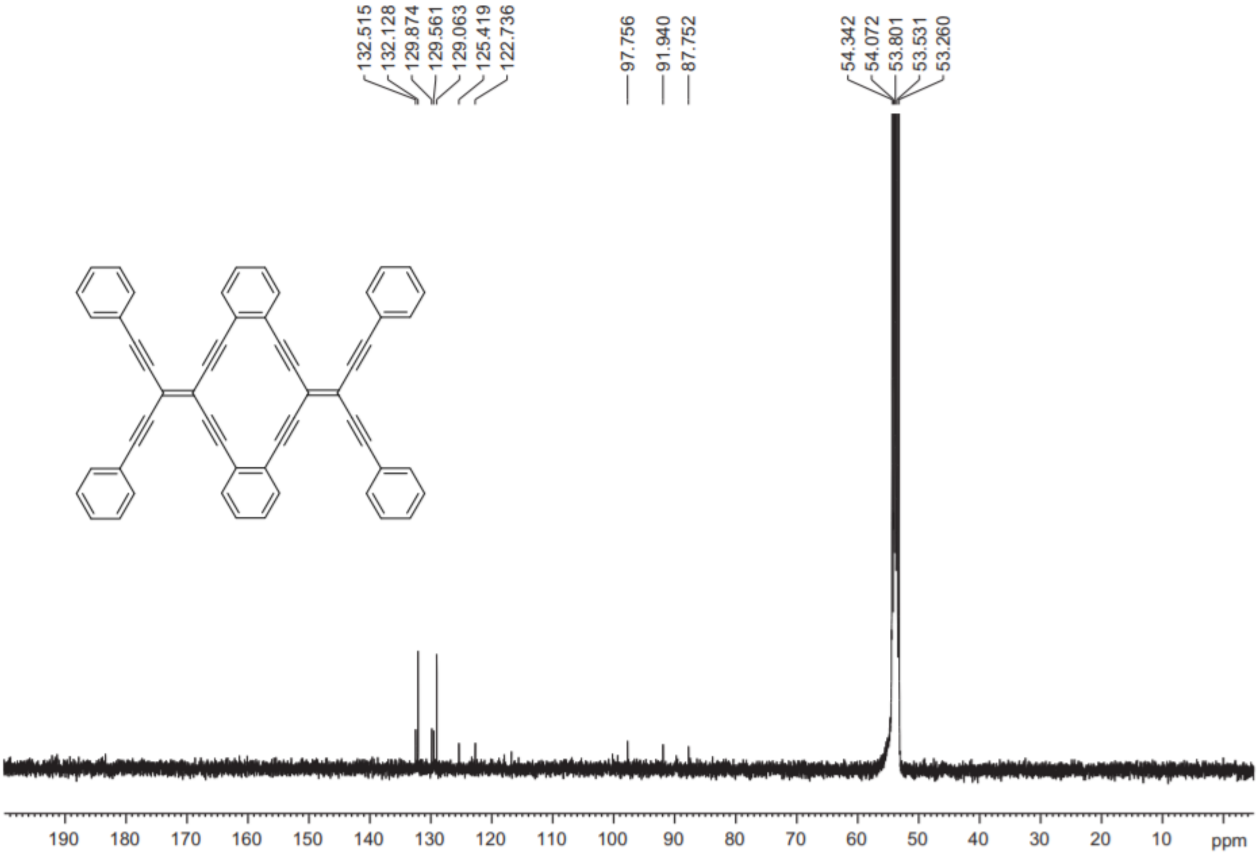

Supplementary Figure 61. ^{13}C-NMR spectrum of **4b** in $C_2D_2Cl_4$ (100 MHz).

Di-radiaannulene 5

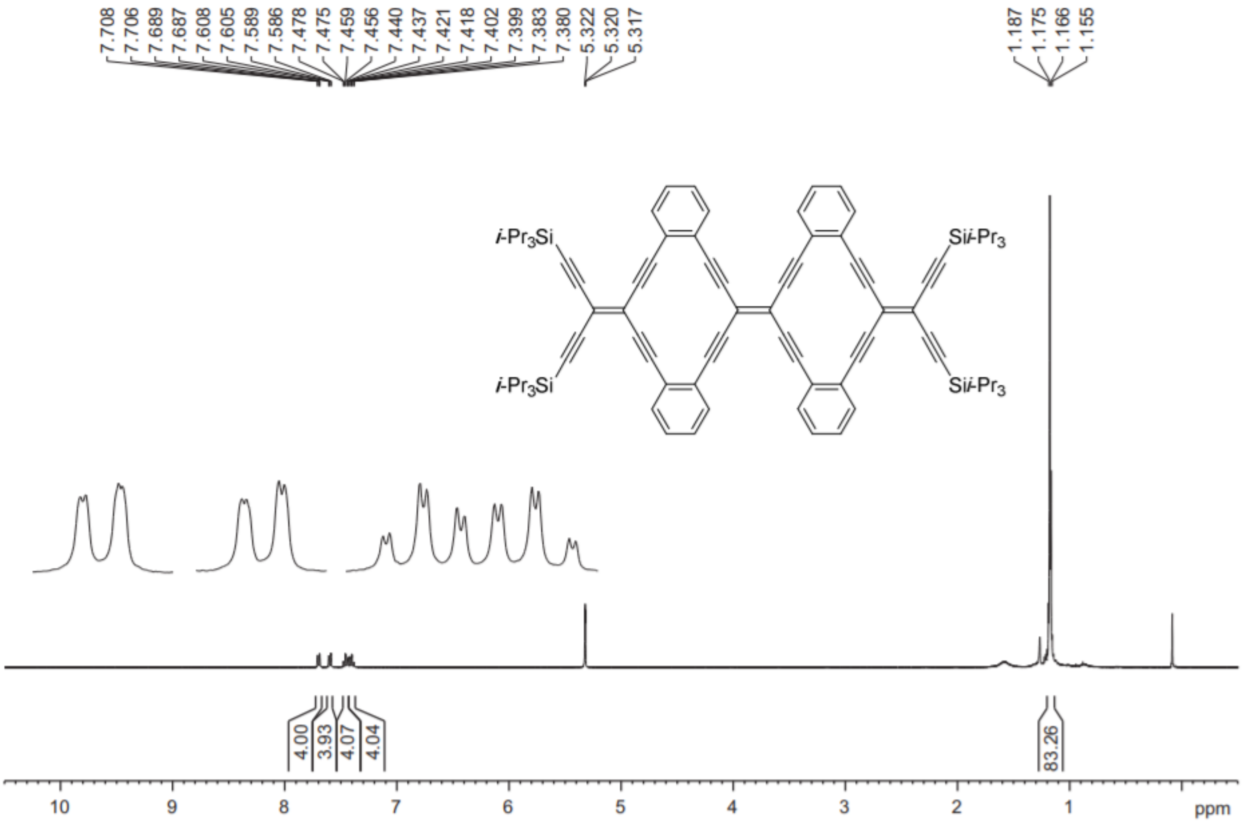

Supplementary Figure 62. ^{1}H-NMR spectrum of **5** in CD_2Cl_2 (400 MHz).

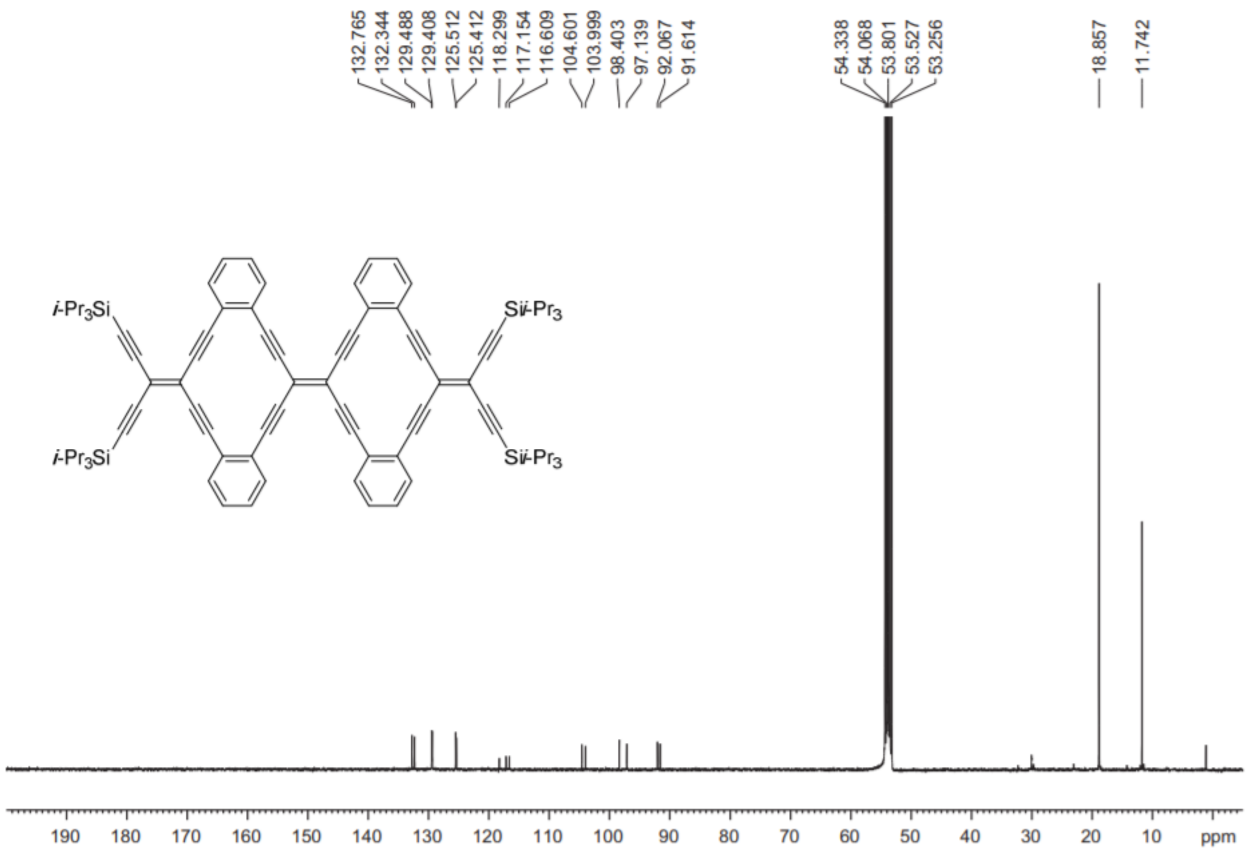

Supplementary Figure 63. ^{13}C-NMR spectrum of **5** in CD_2Cl_2 (75 MHz).

Tri-radiaannulene 6

a)

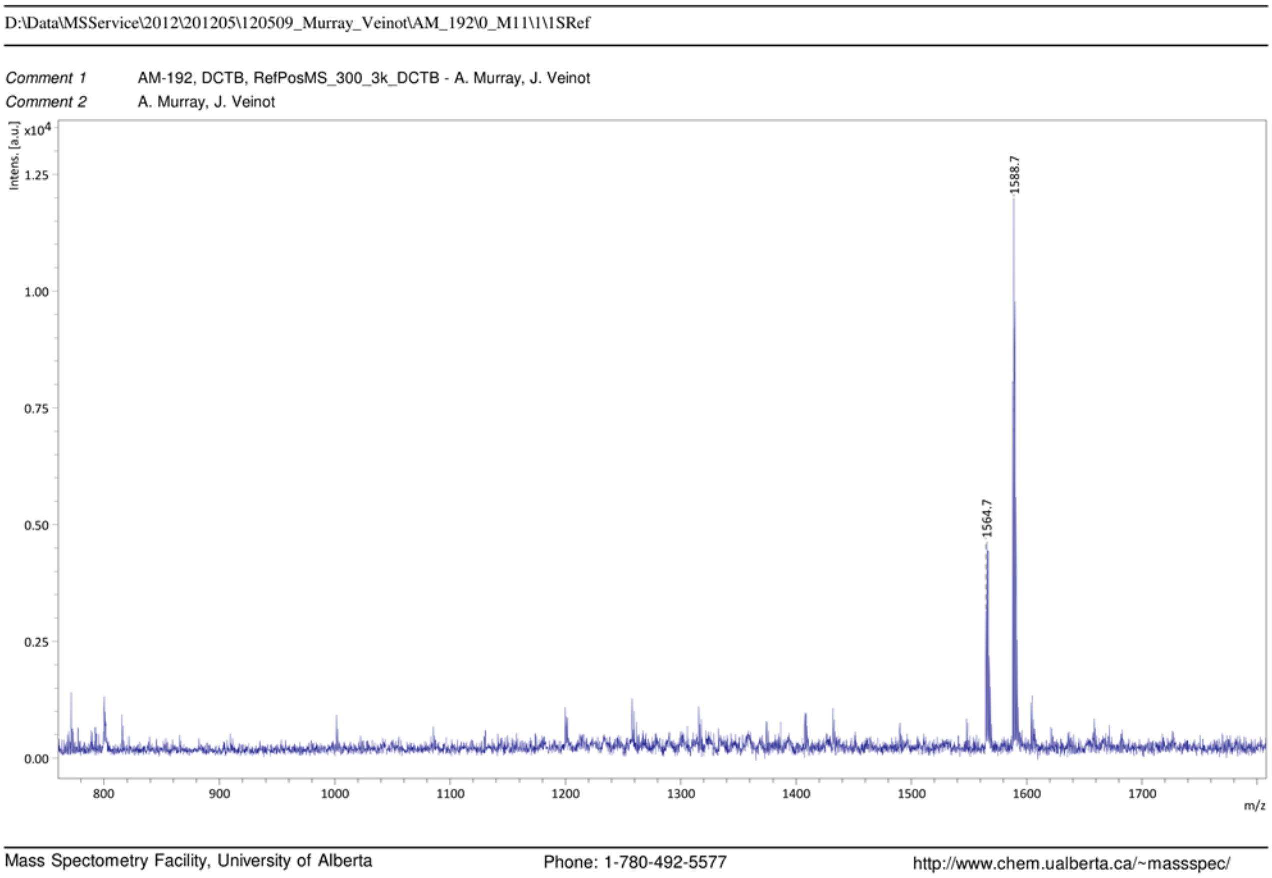

b)

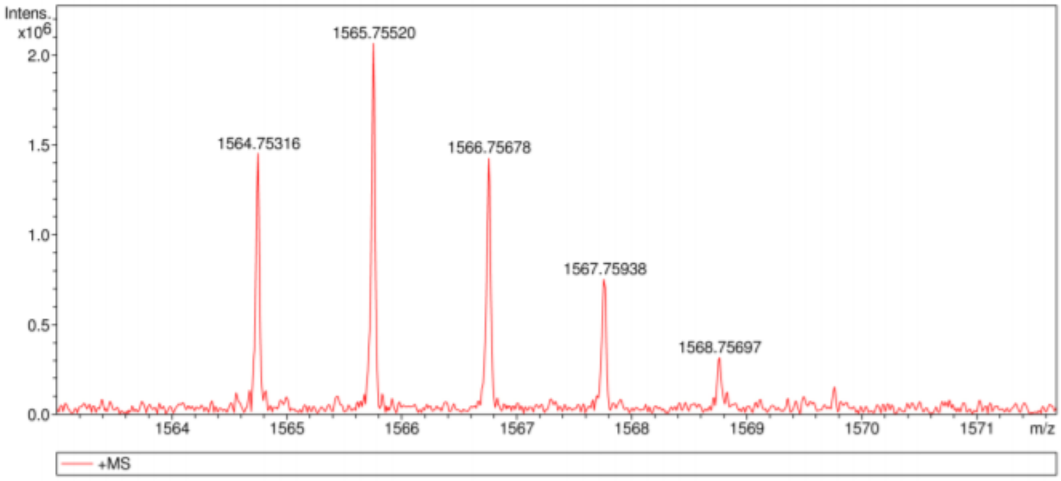

Sum Formula	Sigma	m/z	Err [ppm]	Mean Err [ppm]	Err [mDa]	rdb	N Rule	e⁻
C 112 H 108 Si 4	0.064	1564.75226	-0.57	-0.11	-0.89	63.00	ok	odd

Supplementary Figure 64. a) Low resolution MS of **6**, showing signals of [M$^+$] (*m/z* 1564.7) and [M+ Na$^+$] (m/z 1588.7); b) HRMS MALDI spectrum of **6** ([M$^+$]).

Compound **15a**

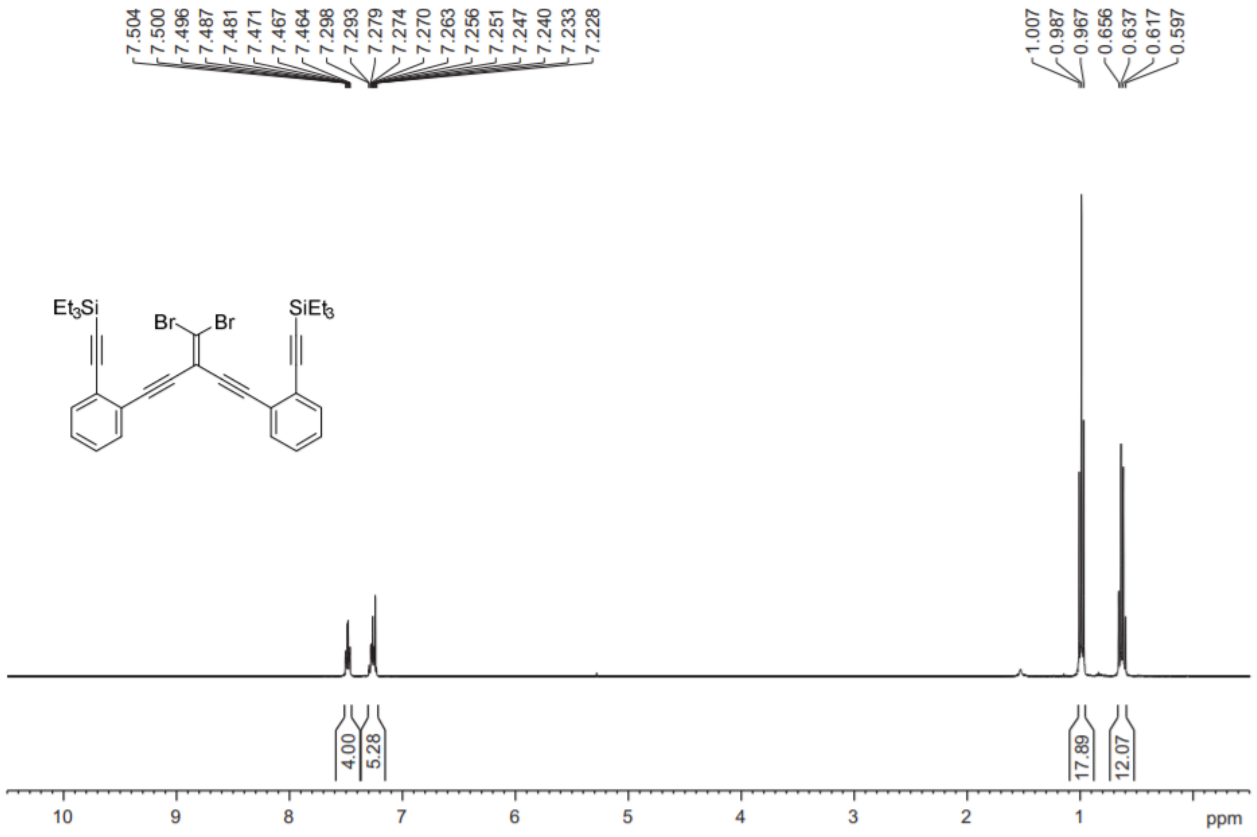

Supplementary Figure 65. ^{1}H-NMR spectrum of **15a** in $CDCl_3$ (300 MHz).

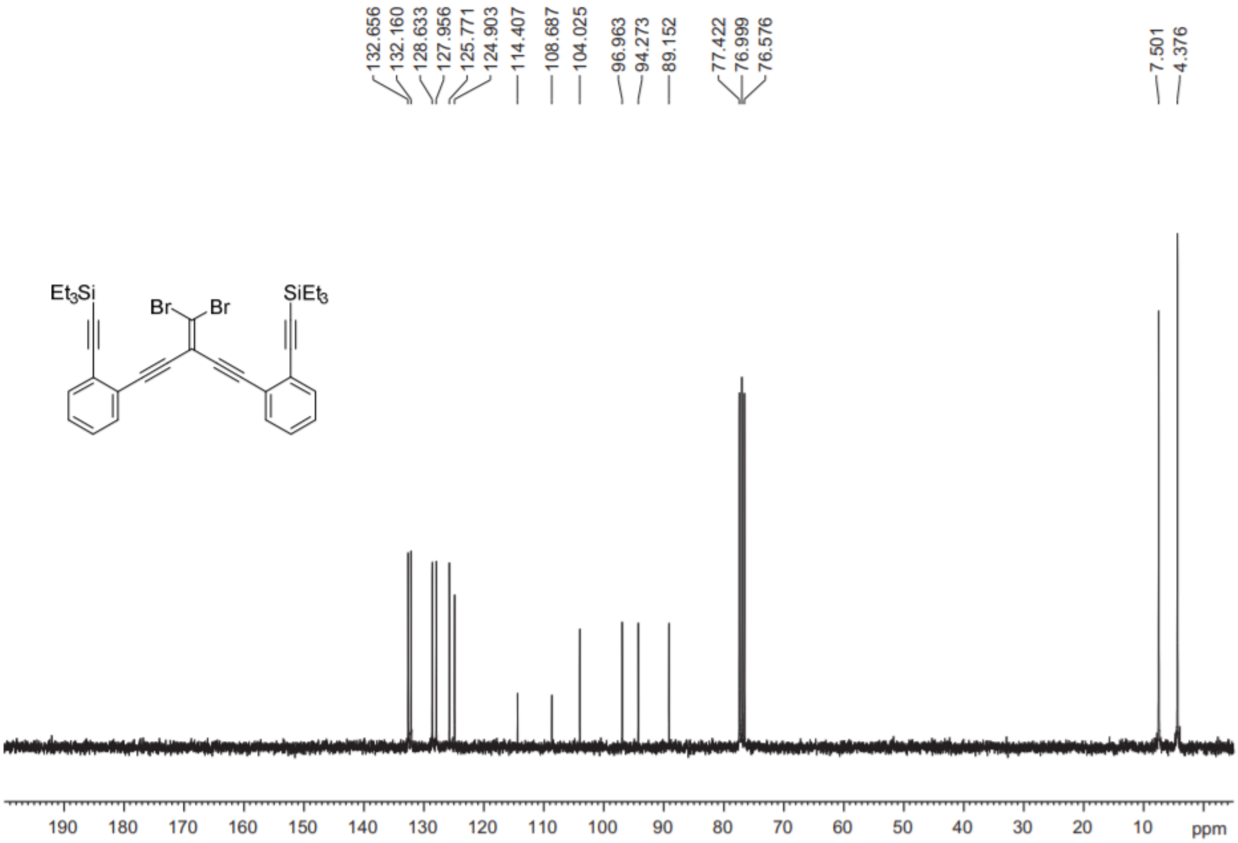

Supplementary Figure 66. ^{13}C-NMR spectrum of **15a** in $CDCl_3$ (75 MHz).

Compound **15b**

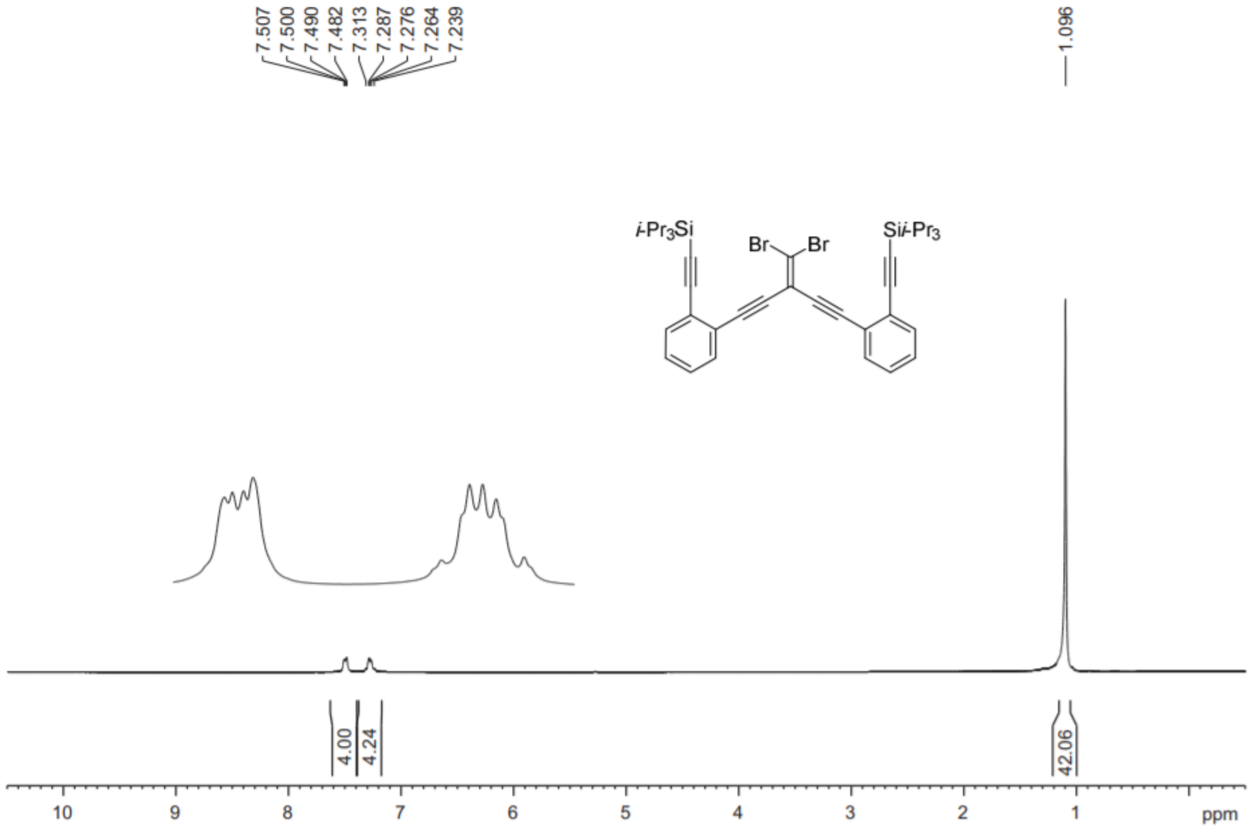

Supplementary Figure 67. ^{1}H-NMR spectrum of **15b** in $CDCl_3$ (300 MHz).

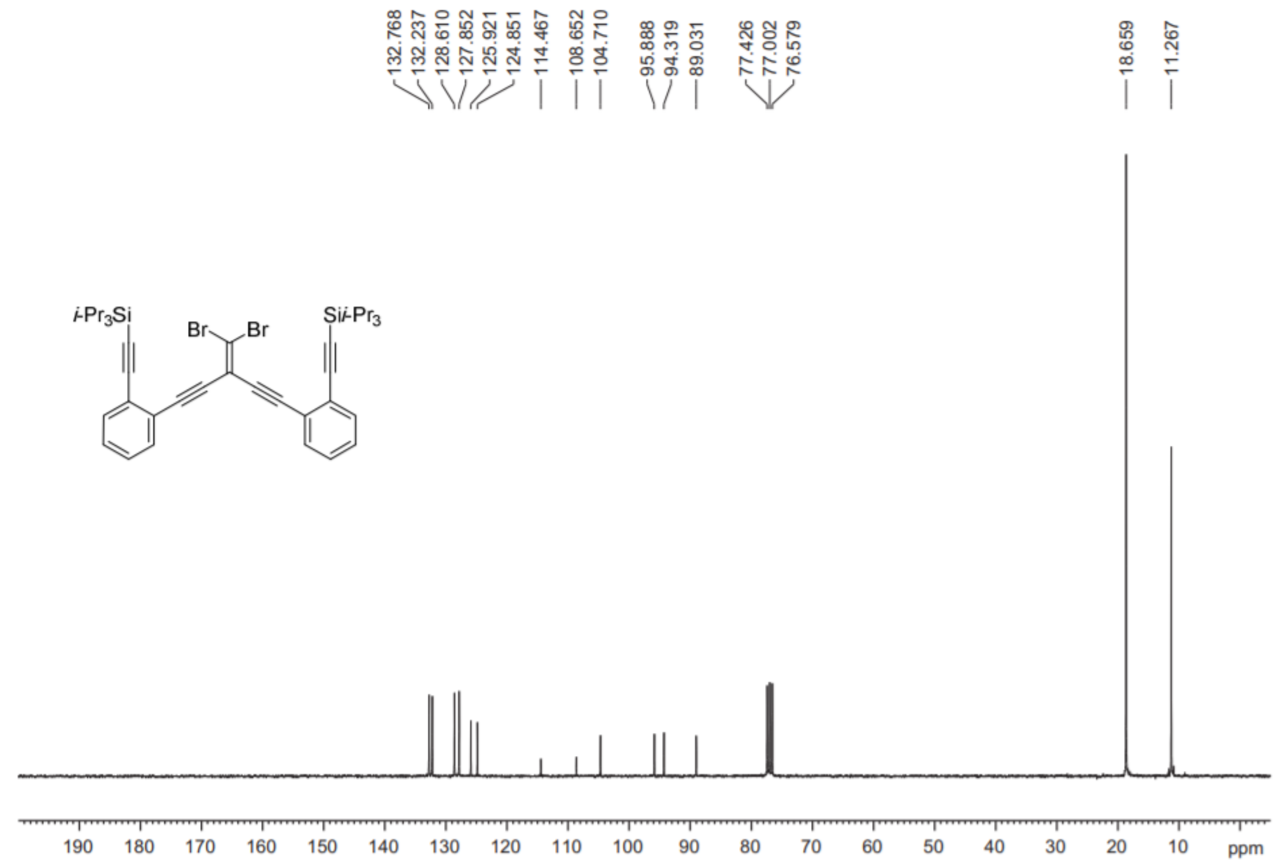

Supplementary Figure 68. ^{13}C-NMR spectrum of **15b** in $CDCl_3$ (75 MHz).

Compound **16a**

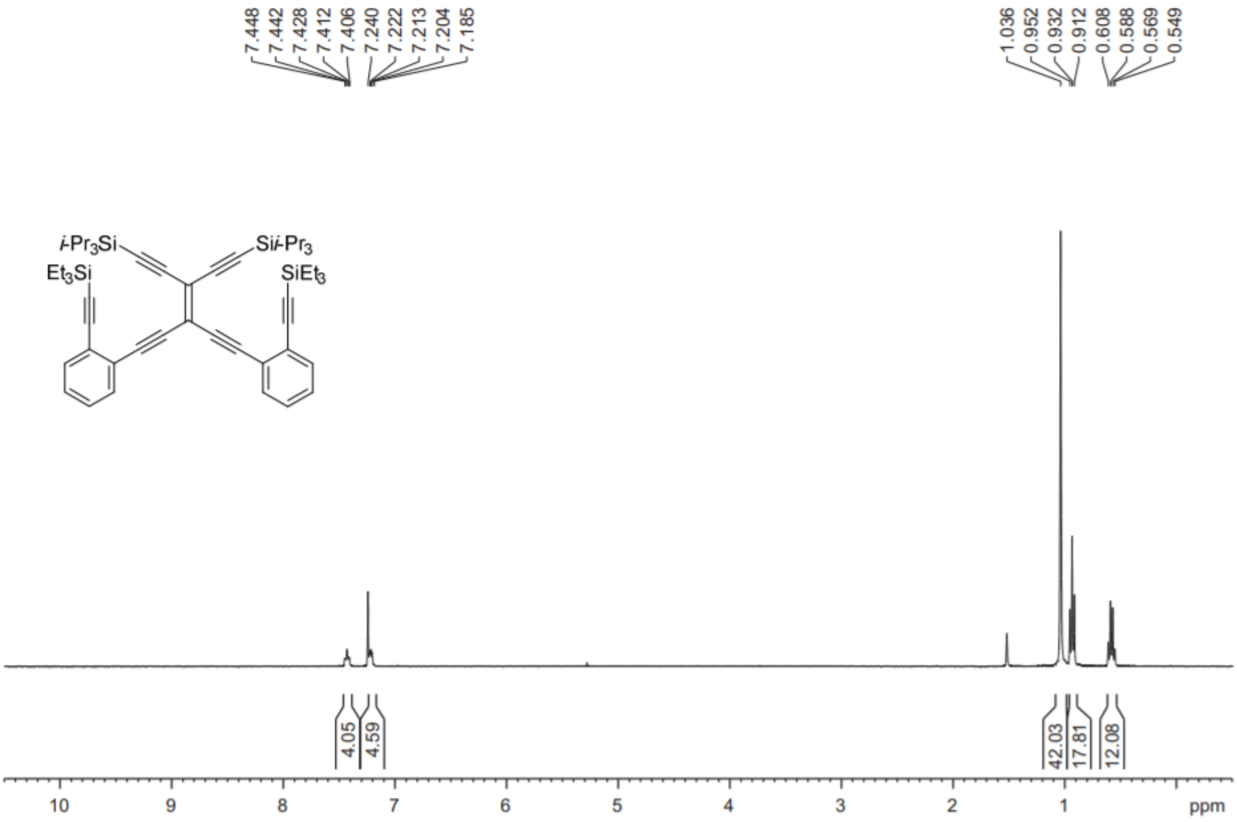

Supplementary Figure 69. ^{1}H-NMR spectrum of **16a** in $CDCl_3$ (300 MHz).

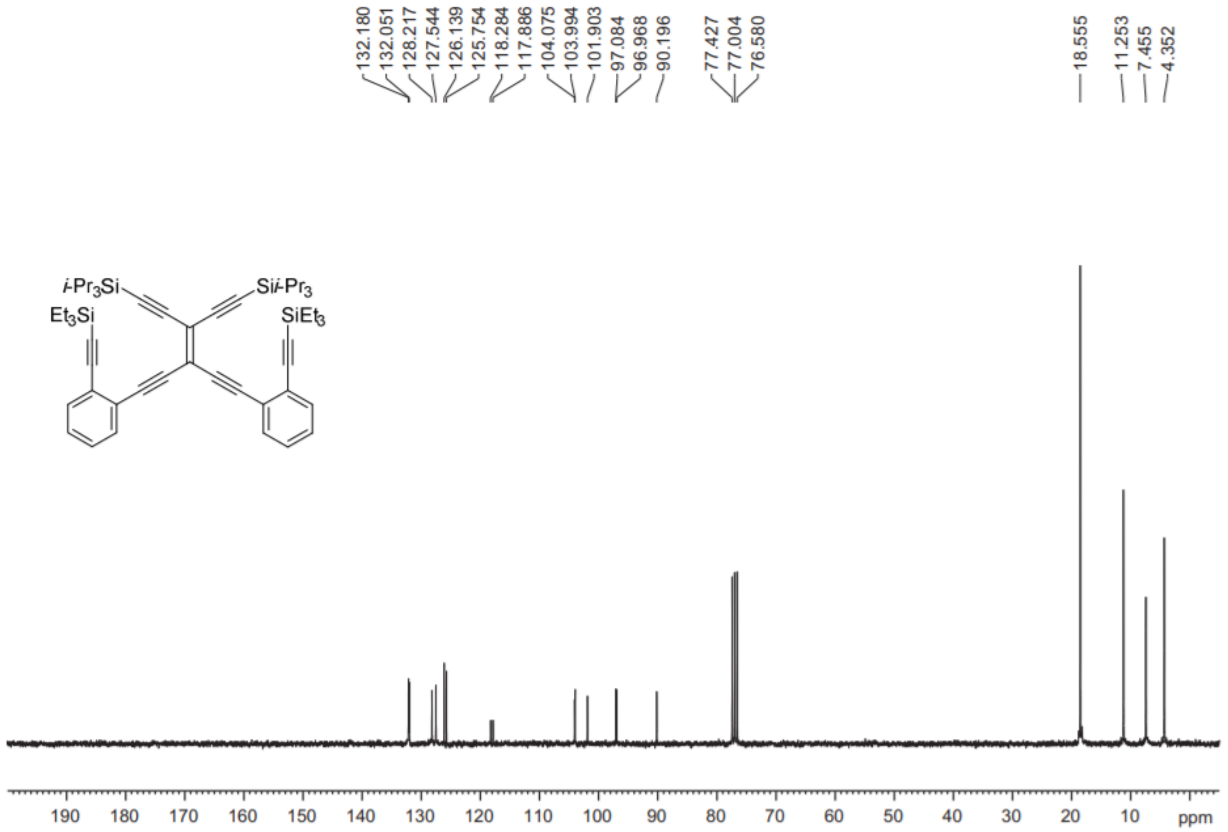

Supplementary Figure 70. ^{13}C-NMR spectrum of **16a** in $CDCl_3$ (75 MHz).

Compound **16b**

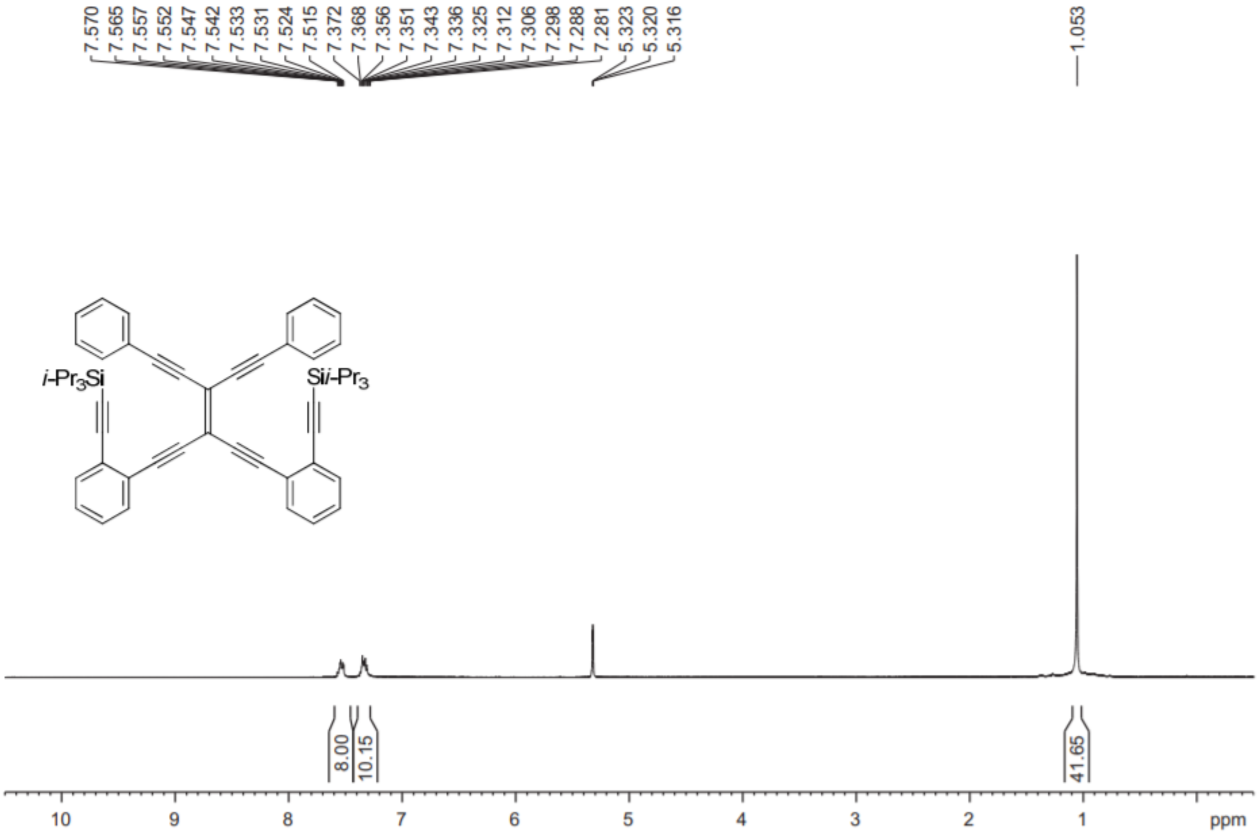

Supplementary Figure 71. ^{1}H-NMR spectrum of **16b** in CD_2Cl_2 (300 MHz).

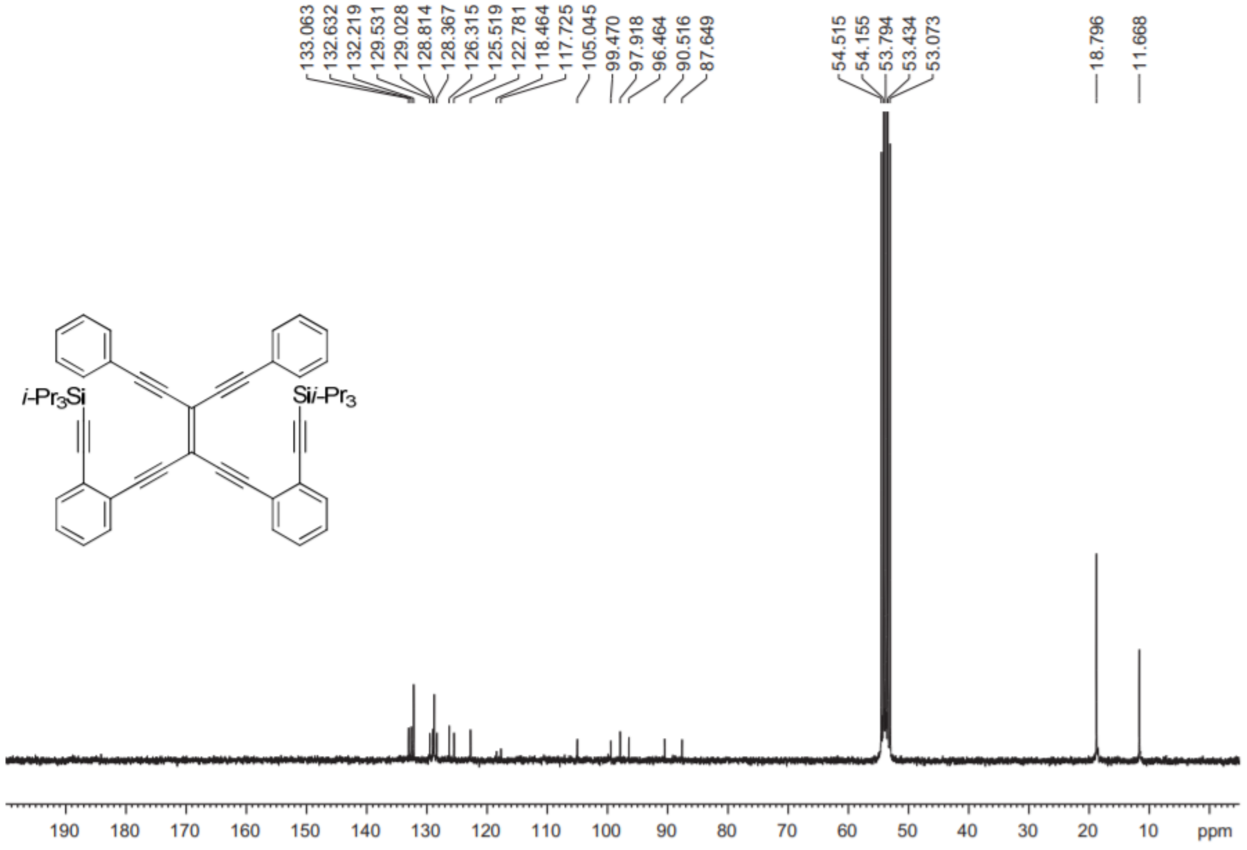

Supplementary Figure 72. ^{13}C-NMR spectrum of **16b** in CD_2Cl_2 (75 MHz).

Compound **17**

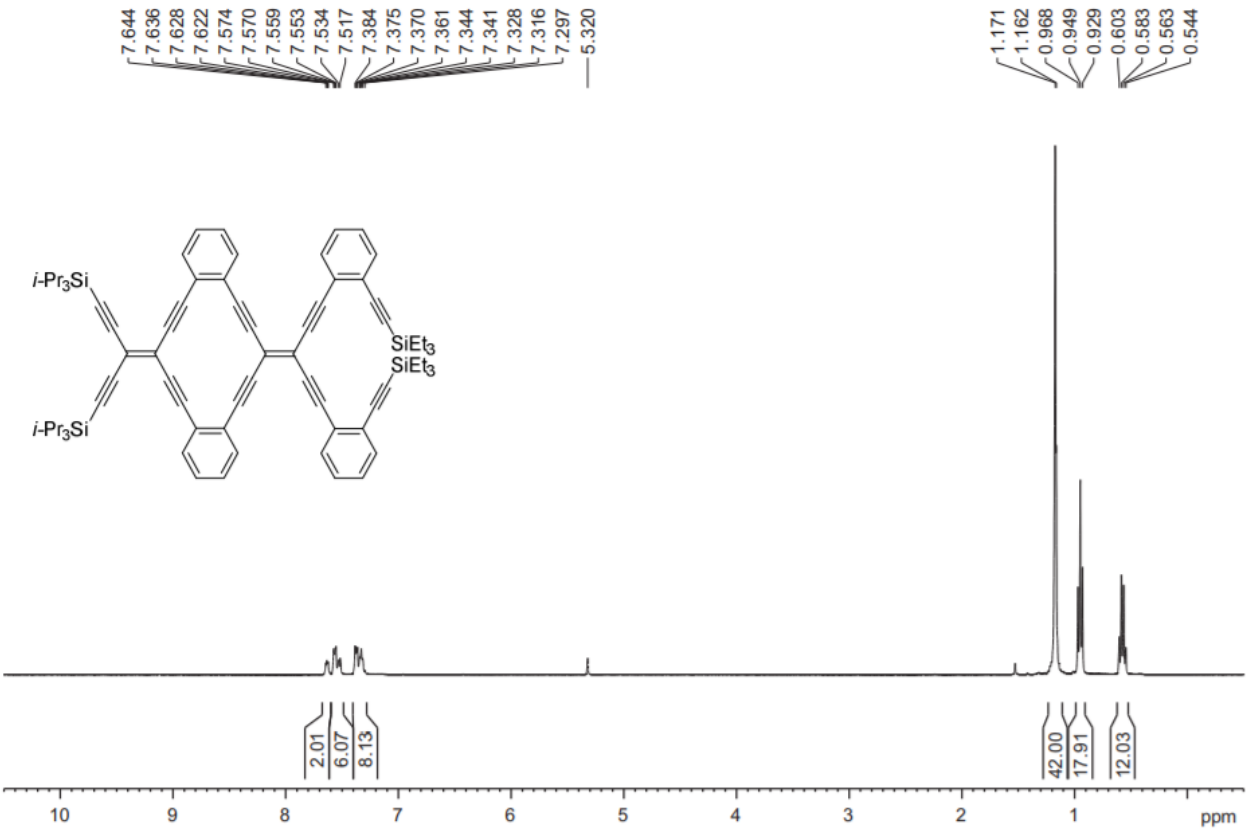

Supplementary Figure 73. ^{1}H-NMR spectrum of **17** in CD_2Cl_2 (300 MHz).

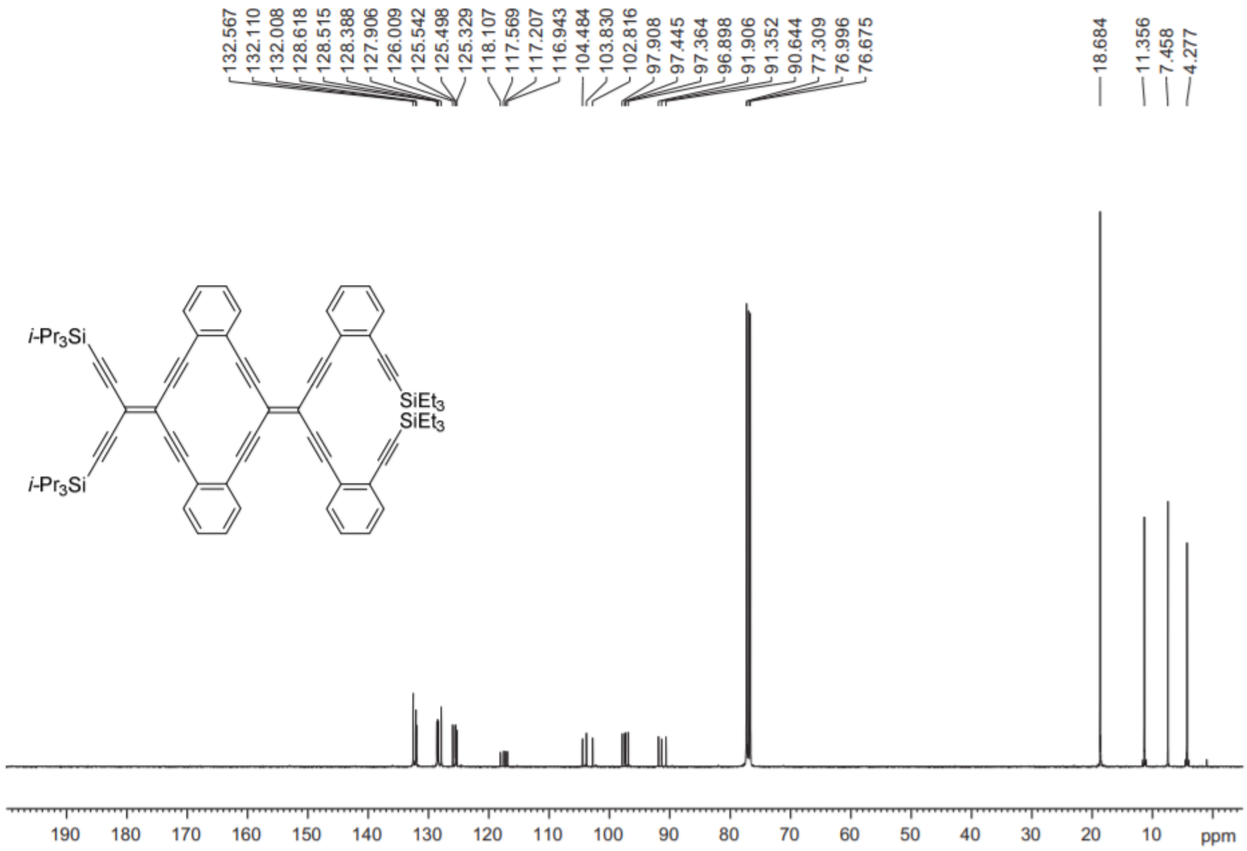

Supplementary Figure 74. ^{13}C-NMR spectrum of **17** in CD_2Cl_2 (75 MHz).

Compound **18**

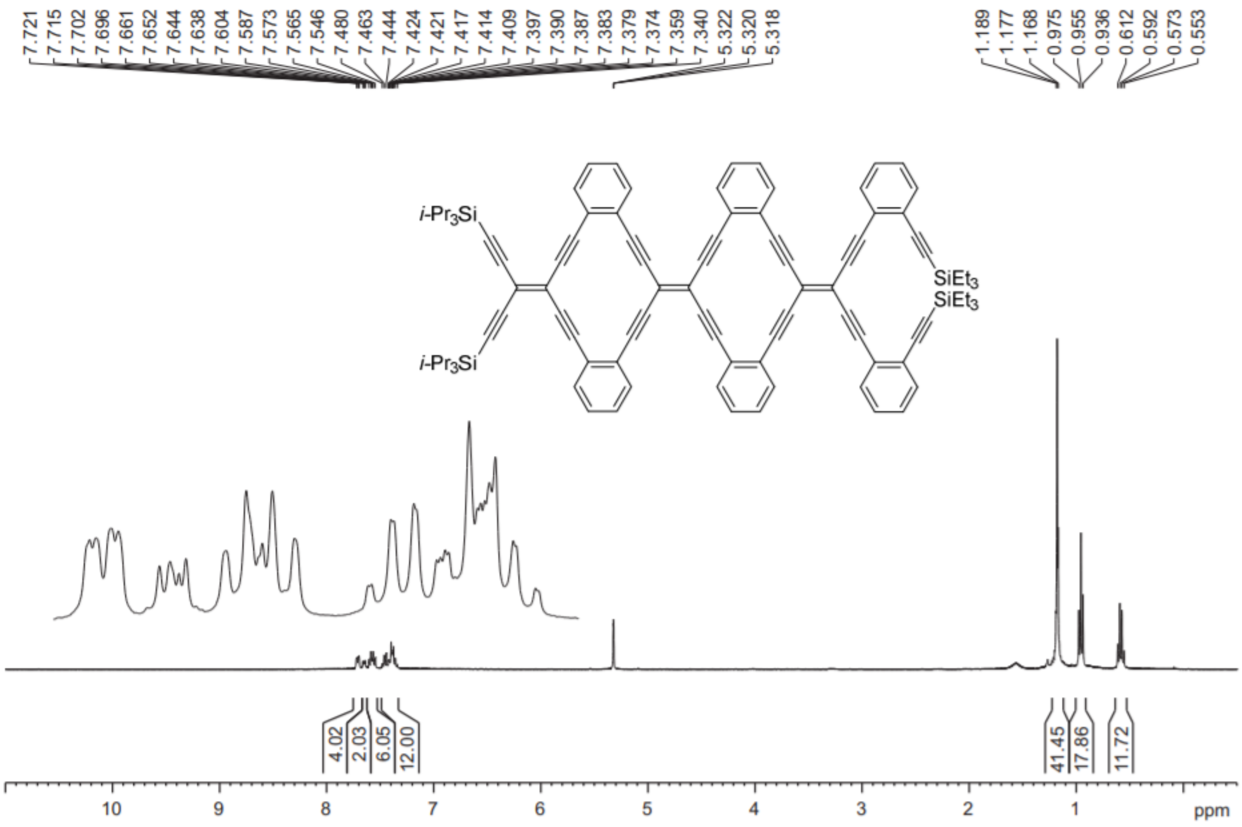

Supplementary Figure 75. ^{1}H-NMR spectrum of **18** in CD_2Cl_2 (300 MHz).

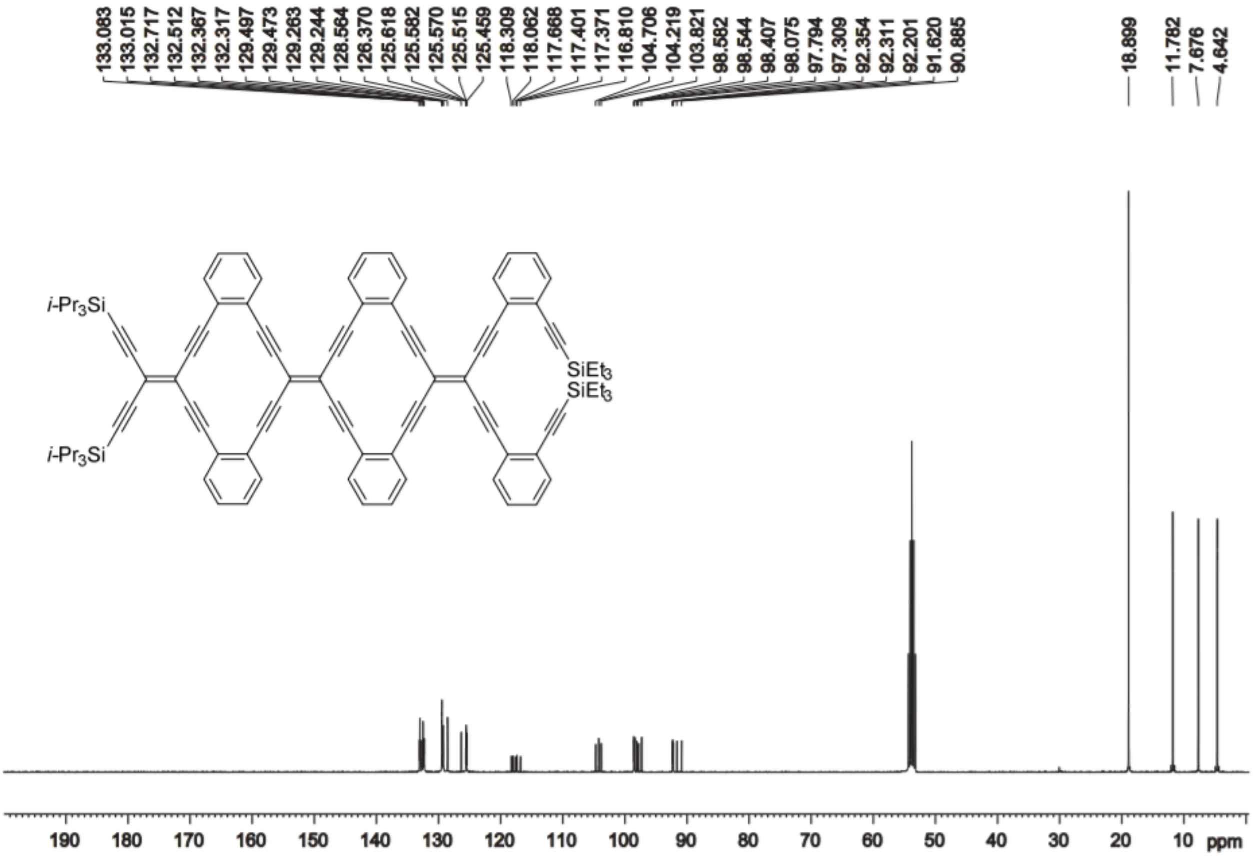

Supplementary Figure 76. ^{13}C-NMR spectrum of **18** in CD_2Cl_2 (75 MHz).

X-Ray crystallographic analysis

Crystals of **S5** were grown from CH_2Cl_2/heptane. The angles for the two different “internal” alkynes are 177.2° (∠ C(sp^2–olefin)–C(sp)–C(sp)) and 167.3° ((∠ C(sp^2–benzene)–C(sp)–C(sp)). Not surprisingly, here the geometry of the internal alkyne being part of the butadiyne bridge deviates significantly from 180°.

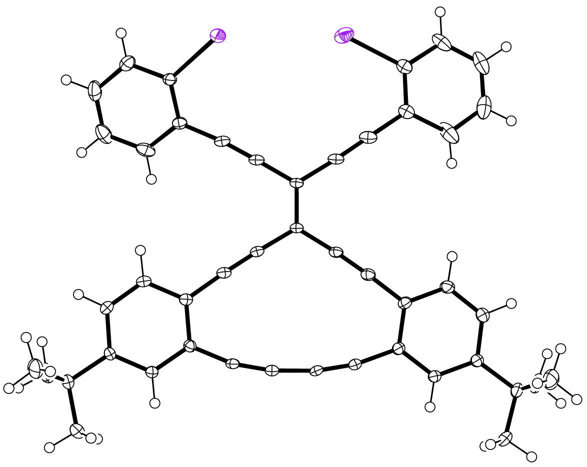

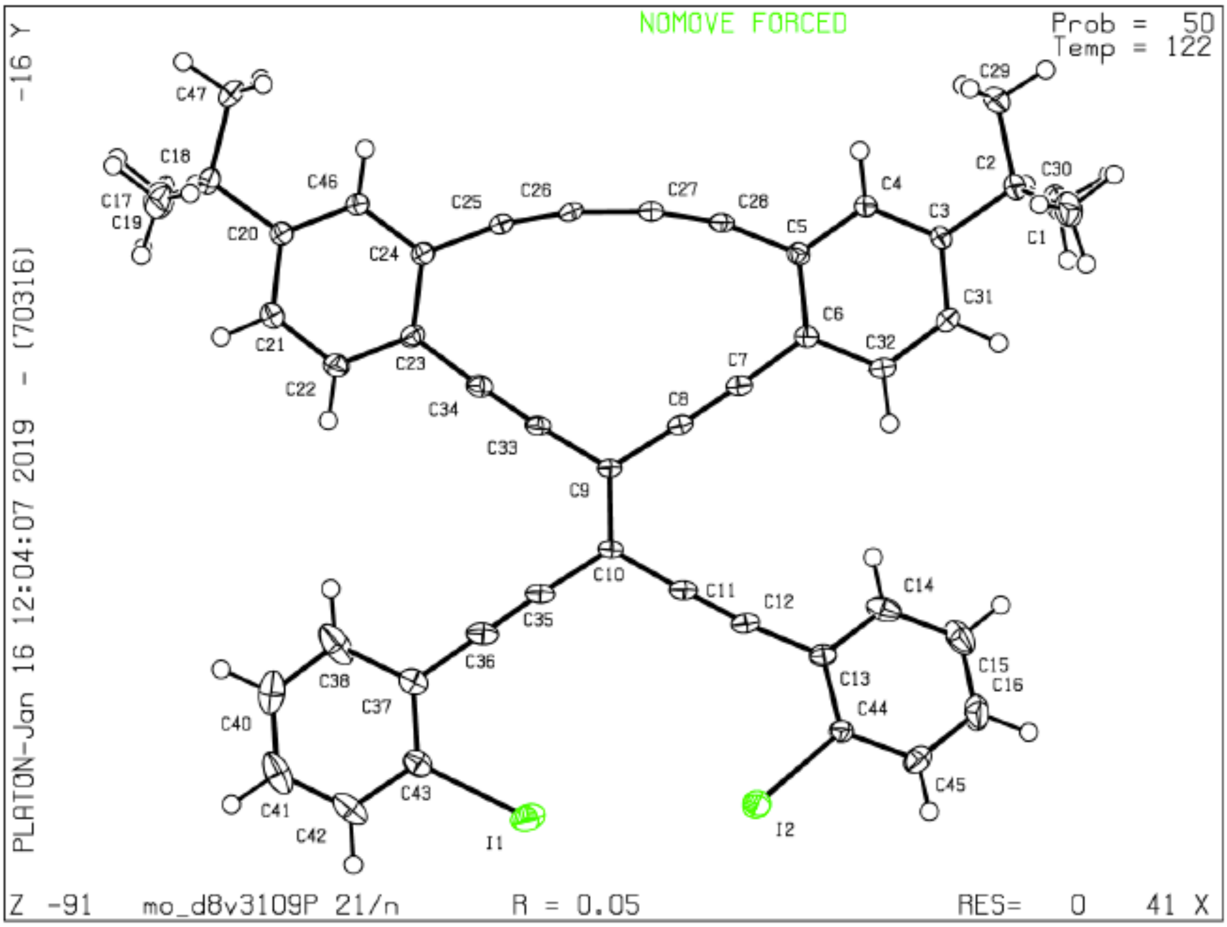

Supplementary Figure 77. ORTEP drawings of molecular structure of **S5**. CCDC 1890763.

Crystal description: orange square (with greenish glue); size 0.066 x 0.246 x 0.337 mm.

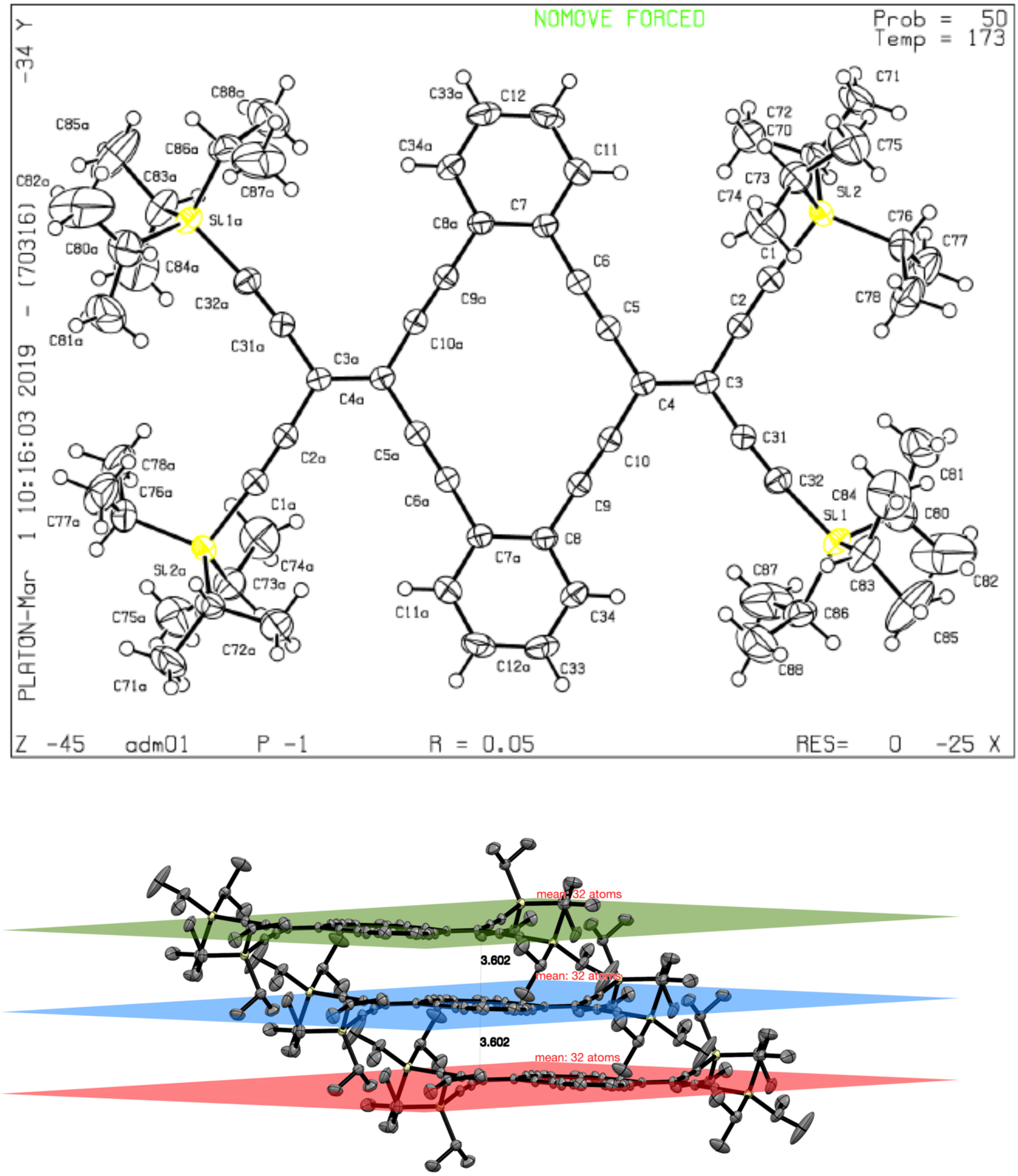

Supplementary Figure 78. Top: ORTEP drawing of molecular structure of **4a**. Bottom: Solid-state packing of **4a** with interplanar distances shown in Å. Each plane calculated from the 32 carbons of the radiaannulene. CCDC 1894937.

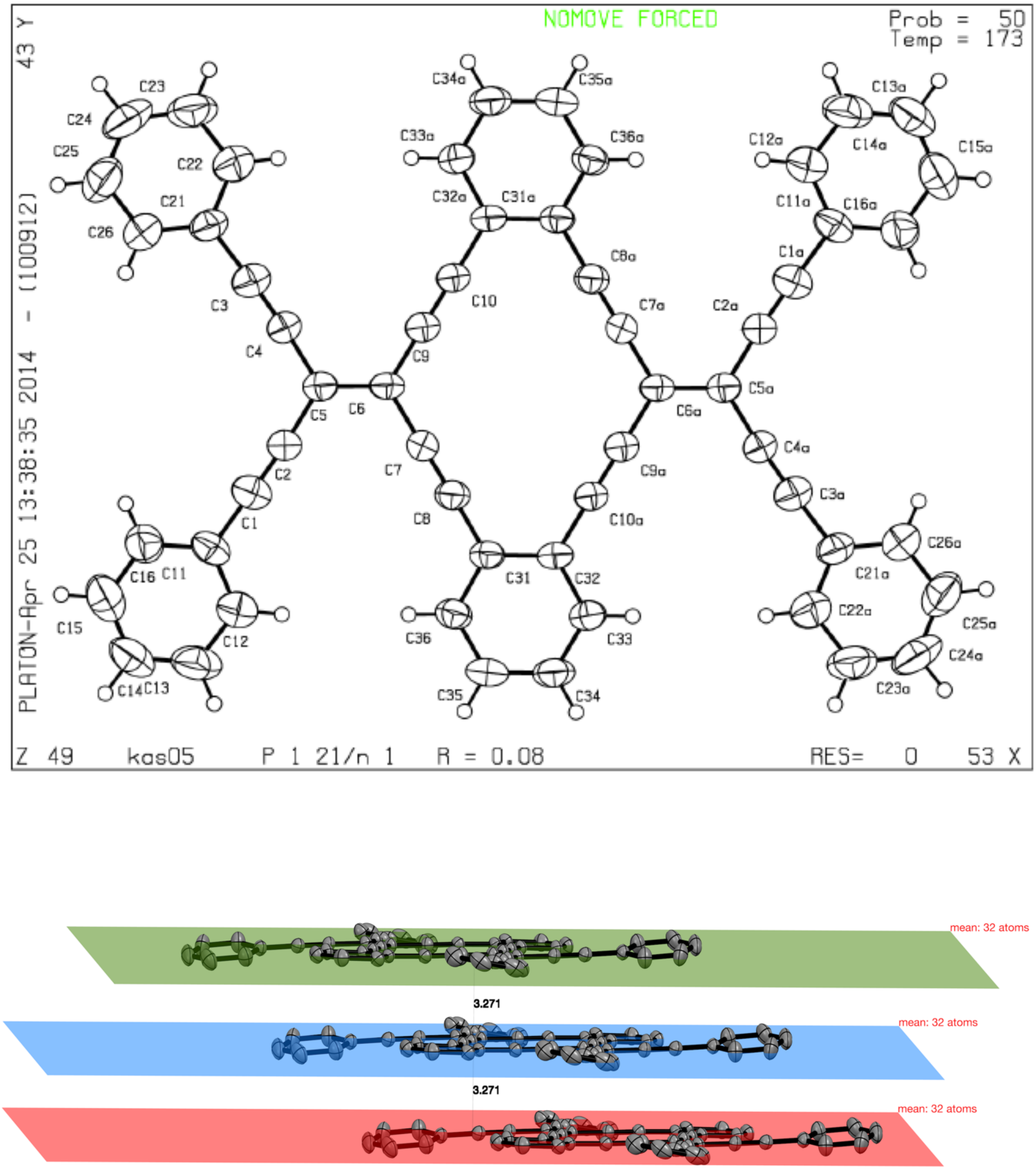

Supplementary Figure 79. Top: ORTEP drawing of molecular structure of **4b**. Bottom: Solid-state packing of **4b** with interplanar distances shown in Å. Each plane calculated from the 32 carbons of the GyNR (excluding the alkylidene phenyl groups). CCDC 1894939.

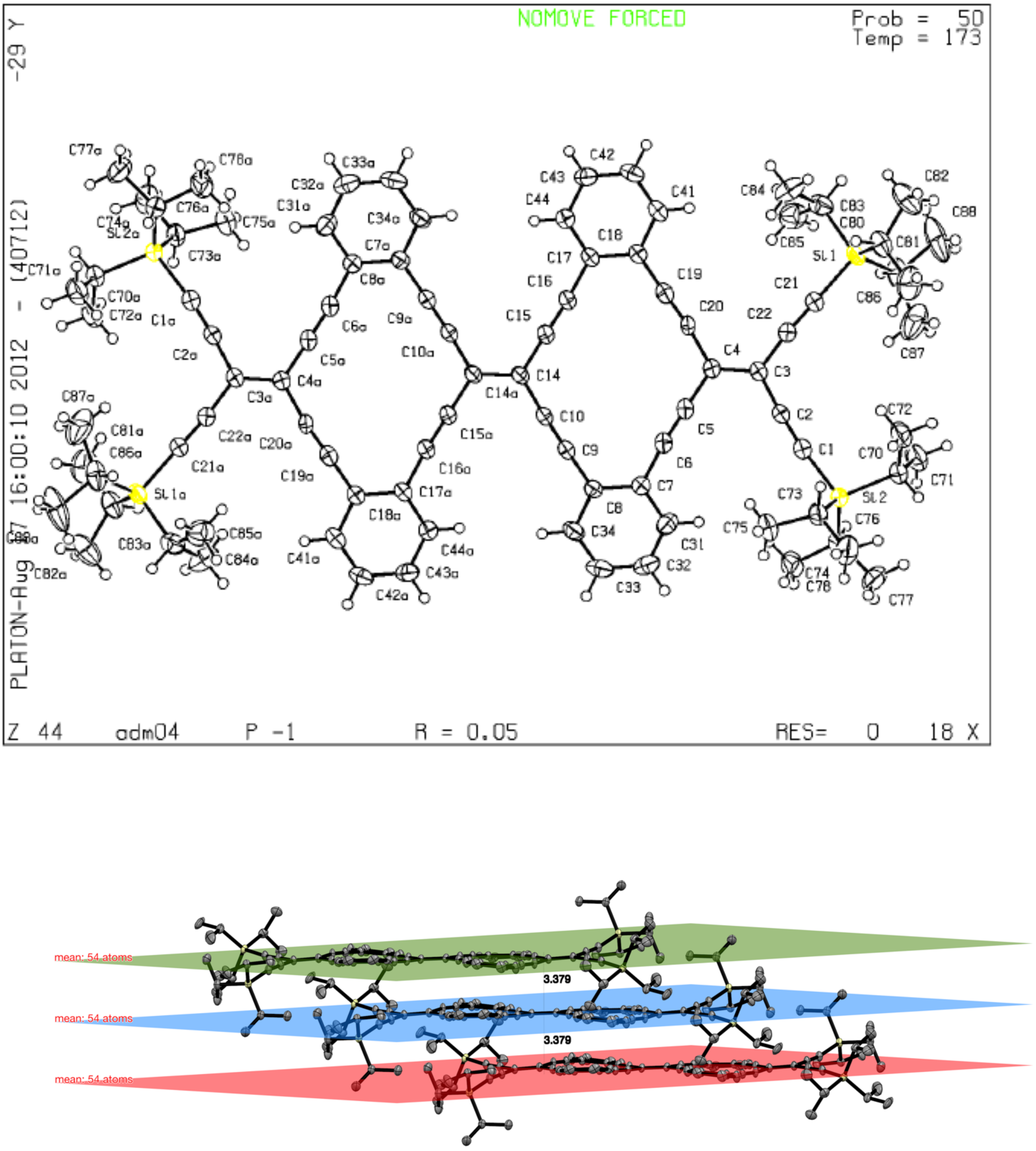

Supplementary Figure 80. Top: ORTEP drawing of molecular structure of **5**. Bottom: Solid-state packing of **5** with interplanar distances shown in Å. Each plane calculated from the 54 carbons of the GyNR. CCDC 1894938.

Comments to Checkcif reports

Structure of S5

EXPT005_ALERT_1_A _exptl_crystal_description is missing

Crystal description: orange square (with greenish glue); size 0.066 x 0.246 x 0.337 mm.

PLAT971_ALERT_2_A Check Calcd Resid. Dens. 0.91A From C38

This peak has been examined and it does not correspond to any atom but rather reflects a high noise level of the residual density.

PLAT230_ALERT_2_B Hirshfeld Test Diff for C37 --C38

This alert could be removed by restraining neighboring bonded temperature parameters to be equal. We have chosen not to do so.

Structure of 4a

PLAT029_ALERT_3_B _diffrn_measured_fraction_theta_full value Low . 0.952 Why?

Poor diffraction at high theta angles is, in our experience and unfortunately, quite characteristic for this class of compounds. Data collection was not continued into this region because there was little to gain. On the other hand, the structure solution and refinement are certainly correct, and the positions and/or distances that would result from analysis in this resolution range (e.g., hydrogen atoms) are calculated in idealized positions and are not discussed in the paper.

PLAT220_ALERT_2_B Non-Solvent Resd 1 C Ueq(max)/Ueq(min) Range 7.0 Ratio

There is no explanation (origin) for this Alert B error that we can determine (perhaps an incorrectly assigned atom doesn't exist, but we cannot tell).

Structure of 4b

THETM01_ALERT_3_B The value of sine(theta_max)/wavelength is less than 0.575

Poor diffraction at high theta angles is, in our experience and unfortunately, quite characteristic for this class of compounds. Data collection was not continued into this region because there was little to gain. On the other hand, the structure solution and refinement are certainly correct, and the positions and/or distances that would result from analysis in this resolution range (e.g., hydrogen atoms) are calculated in idealized positions and are not discussed in the paper.

Structure of 5

PLAT029_ALERT_3_B _diffrn_measured_fraction_theta_full Low 0.948

Poor diffraction at high theta angles is, in our experience and unfortunately, quite characteristic for this class of compounds. Data collection was not continued into this region because there was little to gain. On the other hand, the structure solution and refinement are certainly correct, and the positions and/or distances that would result from analysis in this resolution range (e.g., hydrogen atoms) are calculated in idealized positions and are not discussed in the paper.

PLAT093_ALERT_1_B No su’s on H-atoms, but refinement reported as . mixed

This is a well-known error of the refinement program SHELXL at that time. It represent a formalism without significance to the structure.

PLAT230_ALERT_2_B Hirshfeld Test Diff for C19 -- C20 .. 8.0

To our knowledge, the only rational explanation for this alert is an artifact of the data set during refinement. More, we have not been able to determine.

UV–Vis absorption spectra

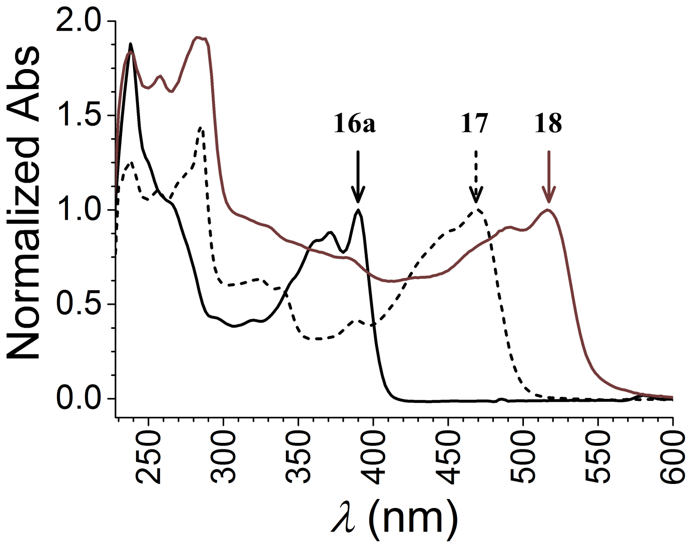

Supplementary Figure 81. UV–Vis absorption spectra of **16a**, **17**, and **18** in THF.

Supplementary Table 1. Absorption maxima (λ_{max}) and molar absorptivities (ε) in brackets of compounds **1**–**3** and **14** recorded in CH_2Cl_2 and of **4a**, **4b**, **5**, **6**, and **16a, 17, 18** recorded in THF. sh = shoulder.

Compound	λ_{max} [nm] (ε [$M^{-1}cm^{-1}$])
**1**	241 (110000), 288 (72700), 313 sh (27400), 351 sh (17900), 470 (31700), 490 (39700)
**2**	292 (100000), 367 (7600), 415 sh (13000), 436 (25200), 453 (33600), 480 (53800)
**3**	293 (107000), 375 (11900), 433 sh (9100), 479 sh (22700), 496 (33000), 530 (55700)
**14**	275 sh (38900), 289 (53800), 320 sh (19400), 351 sh (8500), 431 (23200), 454 (25500)
**4a**	282 (82700), 313 (28200), 330 (32300), 371 (22400), 426 (51800), 450 (65900)
**4b**	255 (31800), 288 (90500), 306 (35100), 331 (23900), 345 (24300), 460 (sh, 41300), 483 (52600)
**5**	287, 343, 415, 485, 504
**6**	284, 490, 522
**16a**	238, 322, 362, 372, 390
**17**	286 (87100), 328 (37800), 338 (35200), 388 (24800), 450 (52300), 470 (59300)
**18**	288, 388, 494, 516

Cyclic voltammetry

Cyclic voltammetry measurements of **1**–**3**, **14**, and **S20** were performed using the Autolab PGSTAT12 instrument and the Nova 1.11 software. The cyclic voltammograms were recorded with scan rate of 0.1 V/s. A silver wire immersed in the solvent-supporting electrolyte mixture and physically separated from the solution containing the substrate by a ceramic frit was used as the reference electrode, a Pt wire was used as the counter electrode, and a glassy-carbon disk electrode (diameter 3 mm) was used as working electrode. The reference electrode potential was referenced to the ferrocene/ferrocenium (Fc/Fc^+) redox couple before and after the experiment. All cyclic voltammograms were recorded in argon atmosphere by purging the solutions with solvent saturated argon before each measurement.

The concentration of the electroactive compounds were: 0.5 mM for **1**, 1 mM for **2**, 0.2 mM for **3**, 0.5 mM for **14**, and 0.5 mM for **S20**. The voltammograms of the compounds were recorded in a dichloromethane (HPLC grade) containing 0.1 M tetrabutylammonium hexafluorophosphate (Bu_4NPF_6) supporting electrolyte. The *i*R-compensation was applied in order to compensate for the solvent resistance.

Cyclic Voltammograms of 1, 2, 3, 14, and S20

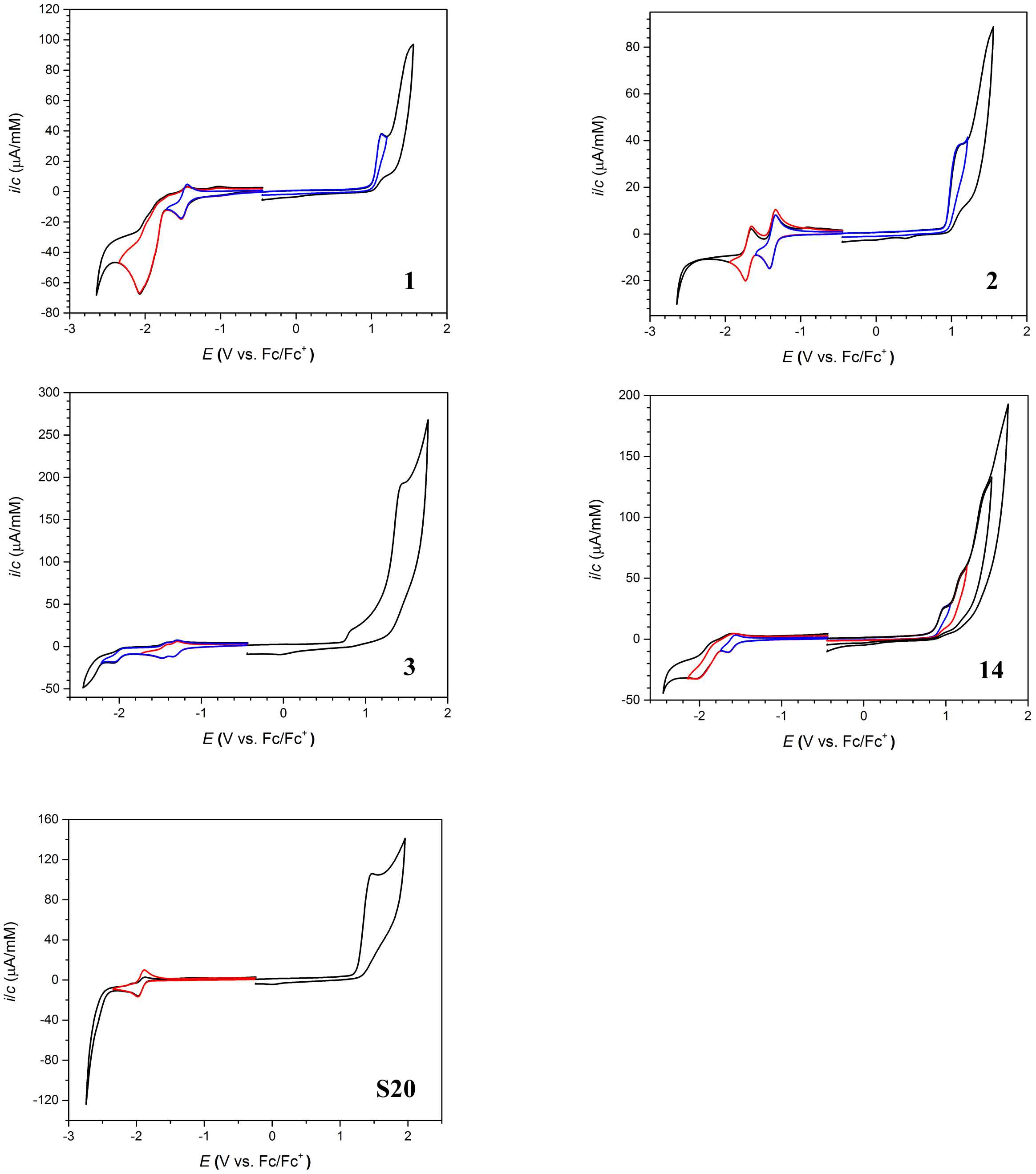

Supplementary Figure 82. Cyclic voltammograms of **1, 2, 3**, **14**, and **S20** recorded in CH_2Cl_2 and Bu_4NPF_6 supporting electrolyte using a Glassy-Carbon working electrode and 0.1 mV/s sweep rate. The potentials are corrected to the formal potential of the Fc/Fc^+ couple.

Cyclic Voltammograms of 17, 18, 4a, and 5

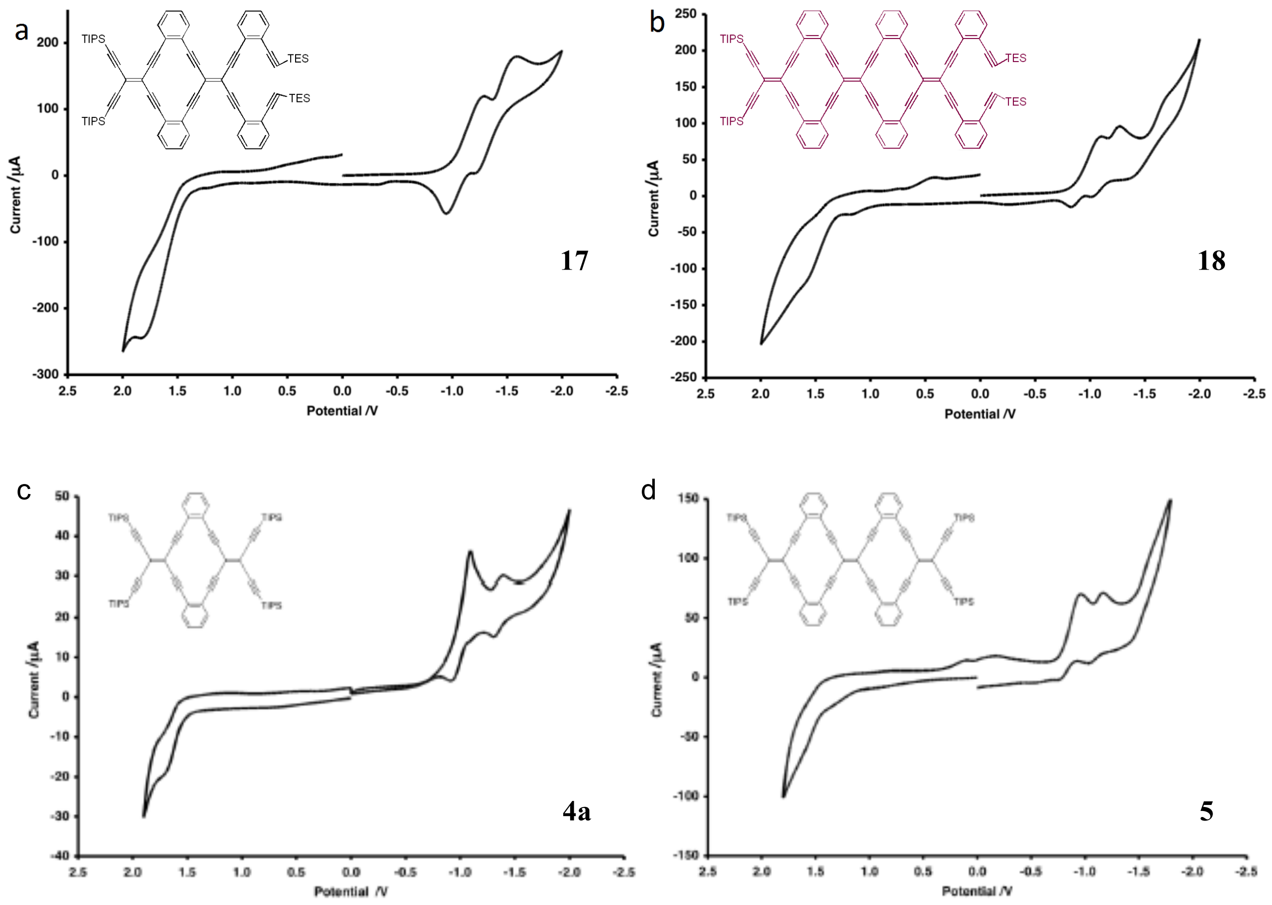

Supplementary Figure 83. Cyclic voltammograms of **17** (a), **18** (b), **4a** (c), and **5** (d) in CH_2Cl_2 (+0.1 M Bu_4NPF_6), scan rate 0.15 V s^{-1}. The first reduction of **4a** is slightly distorted possibly owing to an adsorption phenomenon. The working electrode was a platinum disc, the counter electrode was a platinum wire, and the reference electrode was a non-aqueous Ag/Ag^+ in MeCN. Cyclic voltammetry experiments were done using a Bioanalytical Systems, Inc. (BASi) Epsilon Rotating-Disk Electrode (Model RDE-2). Data was analyzed by BASi Epsilon-EC Ver. 2.00.71-USB, BASi ComServer Ver. 1.03 on a PC computer.

Computational study

NICS calculations on structure 1–3, 4a, 5, 6, 14 – with ^{t}Bu and TIPS groups substituted for hydrogen atoms

All structures were optimized using Density Functional Theory with B3LYP/cc–pVDZ using *Gaussian09*.[8] To confirm that all optimized structures were minimum energy structures, it was confirmed that all values of the Hessian were real. All structures were optimized as neutral structures only, and from these minimum energy structures the NICS values of different charge states were computed. For each structure the NICS values were computed both for ghost atoms, Bq in *Gaussian*, in the plane of the aromatic system (NICS0) and 1Å above the plane (NICS1). For structure **14**, the NICS1 values were computed both one Å above and below the plane since the orientation of the phenyl-acetylene groups might influence the NICS values differently for these two positions. Furthermore, for structure **14** multiple (6^4 = 1296) conformers were created to ensure that the NICS values calculated corresponded to the conformers of lowest energy. These conformers were created by rotating each phenyl-acetylene group 60 degree, a total of 6 position per group, and combining these positions in all possible ways.

Supplementary Table 2. NICS values calculated at position highlighted by colored circle on the structure.

**1**			
Charge	Isotropic	NICSzz	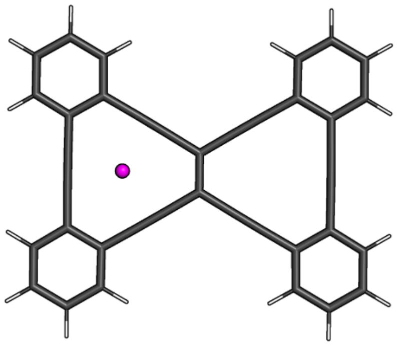
NICS0			
0	5.2023	27.121	
–1	0.2719	12.909	
–2	–4.8638	–2.0963	
NICS1			
0	4.6400	18.795	
–1	0.4793	6.6799	
–2	–3.8972	–6.1517	

Supplementary Table 3. NICS values calculated at position highlighted by colored circle on the structure.

**2**		
Charge	Isotropic	NICSzz
NICS0		
0	0.9959	13.973
–1	–4.1845	–1.0997
–2	–9.6079	–16.814
–3	–6.3648	–6.1434
–4	–3.4696	3.3860
NICS1		
0	1.0326	8.3225
–1	–3.3852	–4.6532
–2	–8.0255	–18.2204
–3	–5.1795	–9.0781
–4	–2.6355	–0.8997

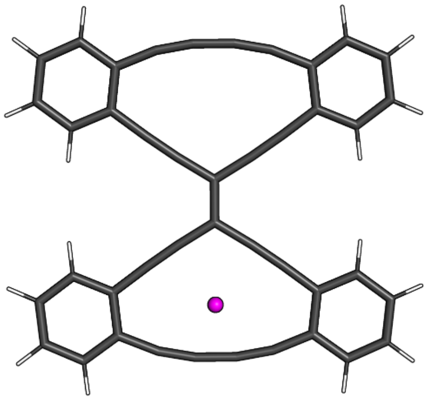

Supplementary Table 4. NICS values calculated at positions highlighted by colored circles on the structures.

**3**				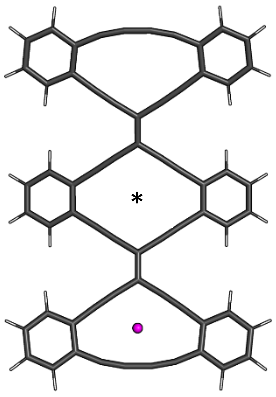
charge	Atom	Isotropic	NICSzz	
NICS0				
0	●	0.8984	13.1853	
0	*	0.4144	9.6872	
–1	●	–2.4099	3.837	
–1	*	–3.2609	–1.1603	
–2	●	–6.1352	–6.6622	
–2	*	–6.4677	–10.6186	
–3	●	–9.3362	–15.5621	
–3	*	–1.9921	2.9956	
–4	●	–12.0273	–22.9626	
–4	*	3.241	18.8977	
–5	●	–4.6361	–0.627	
–5	*	1.1091	12.6789	
–6	●	2.8423	21.8492	
–6	*	0.9514	12.419	
NICS1				
0	●	0.9511	7.8949	
0	*	0.3425	6.0533	
–1	●	–1.8759	–0.1879	
–1	*	–2.8647	–3.3841	
–2	●	–5.0672	–9.2779	
–2	*	–5.6658	–11.6067	
–3	●	–7.7928	–16.9491	
–3	*	–1.662	0.5216	
–4	●	–10.0794	–23.3245	
–4	*	3.0196	14.6845	
–5	●	–3.6514	–4.0956	
–5	*	1.1124	9.0005	
–6	●	2.8522	15.2806	
–6	*	0.9501	8.5432	

Supplementary Table 5. NICS values calculated at positions highlighted by colored circle on the structures.

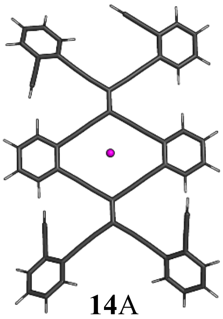
14A

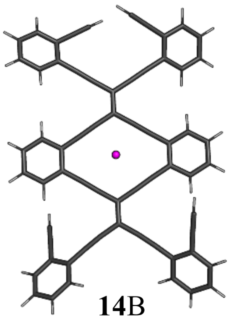
14B

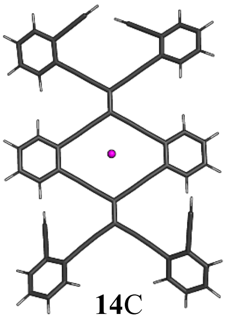
14C

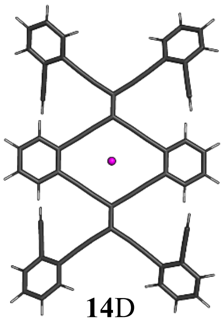
14D

Conf **14**A			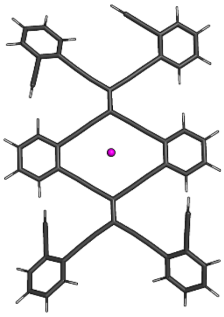
charge	Isotropic	NICSzz	
NICS0			
0	0.4680	9.7628	
–1	–3.8904	–3.0979	
–2	–7.8798	–14.873	
NICS–1			
0	0.3989	5.9011	
–1	–3.3941	–5.3274	
–2	–6.8684	–15.604	
NICS1			
0	0.3931	5.8840	
–1	–3.3883	–5.3400	
–2	–6.8500	–15.622	

Conf **14**B			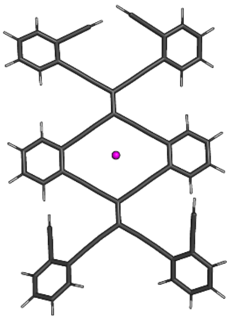
charge	Isotropic	NICSzz	
NICS0			
0	0.4280	9.6484	
−1	−3.9267	−3.1913	
−2	−7.9204	−14.971	
NICS−1			
0	0.3633	5.7962	
−1	−3.4249	−5.4229	
−2	−6.9024	−15.722	
NICS1			
0	0.3637	5.7977	
−1	−3.4249	−5.4227	
−2	−6.9029	−15.723	

Supplementary Table 5 continues on next page

Supplementary Table 5 – *Continued.*

Conf **14**C		
charge	Isotropic	NICSzz
NICS0		
0	0.4252	9.6559
−1	−3.9311	−3.0823
−2	−7.9354	−14.8009
NICS−1		
0	0.3608	5.8399
−1	−3.4297	−5.2988
−2	−6.918	−15.5539
NICS1		
0	0.3603	5.8385
−1	−3.4298	−5.2989
−2	−6.9176	−15.5523

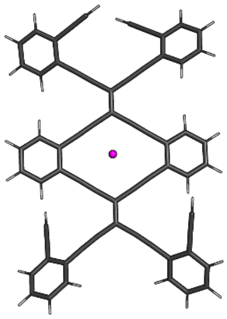

Conf **14**D		
charge	Isotropic	NICSzz
NICS0		
0	0.5172	9.8979
−1	−3.8986	−2.9492
−2	−7.9558	−14.773
NICS−1		
0	0.4333	6.0725
−1	−3.4014	−5.1394
−2	−6.9247	−15.4533
NICS1		
0	0.4333	6.0725
−1	−3.4014	−5.1393
−2	−6.9247	−15.4532

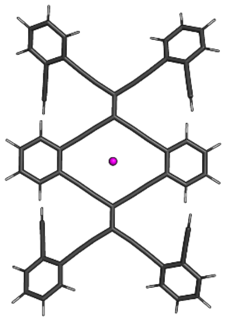

Supplementary Table 5 continues on next page

Supplementary Table 5 – *Continued.*

Average **14**		
charge	Isotropic	NICSzz
NICS0		
0	0.4596	9.74125
–1	–3.9117	–3.080175
–2	–7.92285	–14.854475
NICS–1		
0	0.389075	5.902425
–1	–3.412525	–5.297125
–2	–6.903375	–15.5833
NICS1		
0	0.3876	5.898175
–1	–3.4111	–5.300225
–2	–6.8988	–15.587625

**4a**			
charge	Isotropic	NICSzz	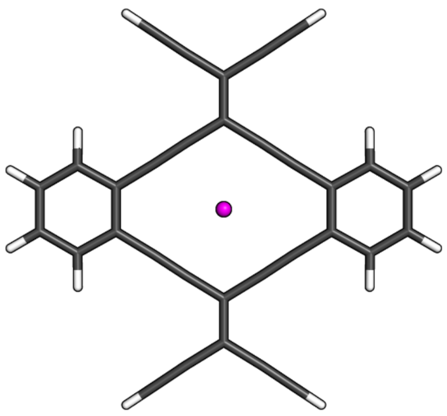
NICS0			
0	0.4455	9.4100	
–1	–4.3585	–4.7057	
–2	–8.2754	–16.1722	
NICS–1			
0	0.3746	5.5284	
–1	–3.8042	–6.8212	
–2	–7.2131	–16.8638	
NICS1			
0	0.3746	5.5284	
–1	–3.8043	–6.8212	
–2	–7.2132	–16.8639	

Supplementary Table 6. NICS values calculated at position highlighted by colored circle on the structure.

**5**		
charge	Isotropic	NICSzz
NICS0		
0	0.4398	9.5936
–1	–2.6630	0.4925
–2	–5.6697	–8.3286
NICS–1		
0	0.3477	5.5012
–1	–2.3423	–2.4486
–2	–4.9498	–10.1608
NICS1		
0	0.3918	6.0234
–1	–2.3132	–1.9453
–2	–4.9406	–9.6747

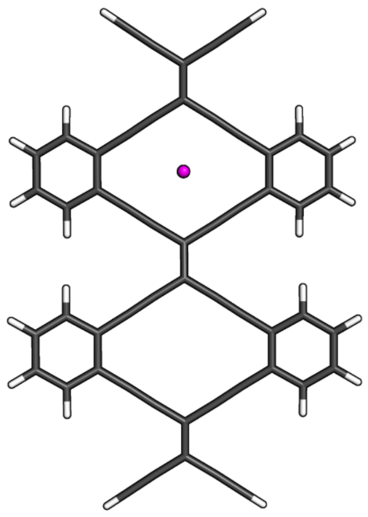

Supplementary Table 7. NICS values calculated at position highlighted by colored circle on the structures.

**6**		
charge	Isotropic	NICSzz
NICS0		
0	0.4388	9.5390
–1	–1.5798	3.6740
–2	–3.9051	–3.1052
NICS–1		
0	0.3903	5.9515
–1	–1.3627	0.8437
–2	–3.3921	–5.0805
NICS1		
0	0.3491	5.4691
–1	–1.4057	0.3344
–2	–3.4269	–5.5949

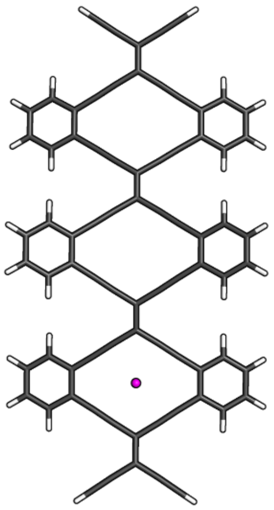

**6**		
charge	Isotropic	NICSzz
NICS0		
0	0.4472	9.8384
–1	–2.5969	0.8366
–2	–4.9340	–6.0656
NICS–1		
0	0.3357	5.5317
–1	–2.3013	–2.3440
–2	–4.3279	–8.3979
NICS1		
0	0.4131	6.5307
–1	–2.2585	–1.3553
–2	–4.3064	–7.3847

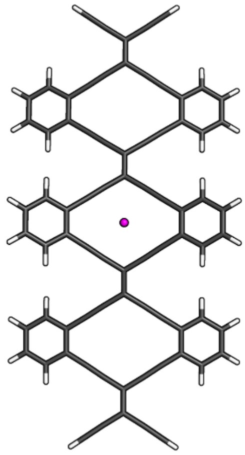

Output data

1


```
1\1\GINC-NODE381\FOpt\RB3LYP\CC-pVDZ\C38H16\ROOT\17-Oct-2017\0\\#opt f
 req b3lyp/cc-pvdz scf(maxcycle=250) CPHF(MaxInv=10000)\\something titl
 e\\0,1\C,-2.9191492446,1.8494766063,-0.30148688\C,-2.1149564503,1.5704
 212134,-1.4111994682\C,-0.8804361173,0.8844563543,-1.2621165546\C,-2.5
 116290124,1.9661267591,-2.7161723308\C,-4.1533805249,2.535977921,-0.45
 05538521\C,-2.5224859328,1.4538022391,1.0034860574\C,0.1798439351,0.29
 37471498,-1.1233592412\C,-2.8618700247,2.3105453828,-3.8346936863\C,-5
 .2135250257,3.1269272992,-0.5893245531\C,-2.1724102253,1.1092365796,2.
 1220146866\C,-3.2861136227,2.719357935,-5.131213533\C,-6.4465084908,3.
 8162673805,-0.7698942411\C,-1.748024537,0.7006309387,3.4185495301\C,1.
 4129043332,-0.3954477942,-0.9427162869\C,1.8322317228,-0.8023811428,0.
 3605037155\C,3.0563596674,-1.4810994575,0.5014548244\C,3.8562346617,-1
 .7576892979,-0.6080014652\C,3.4453113525,-1.3590886484,-1.8843596773\C
 ,2.235418475,-0.6847515802,-2.0480705575\C,-2.5445578584,0.9727544876,
 4.547053316\C,-2.1401463769,0.5777246036,5.8221122604\C,-0.9277009265,
 -0.098850946,5.9925500107\C,-0.1226796564,-0.3787865382,4.887666065\C,
 -0.5101227019,0.0098369744,3.5923364292\C,-6.8657842862,4.223414296,-2
 .0730584515\C,-8.0896855403,4.9025754942,-2.213879633\C,-8.8894411245,
 5.1792694927,-1.1043689229\C,-8.4786022681,4.7803931254,0.1719346277\C
 ,-7.2688744349,4.1057460991,0.3355360329\C,-2.4896594742,2.4469983187,
 -6.2597195472\C,-2.8939656123,2.8421309874,-7.5347799677\C,-4.10616578
 86,3.5191493994,-7.7051998524\C,-4.911079501,3.7993692015,-6.600309824
 4\C,-4.5237943994,3.4105565595,-5.3049941477\C,-5.354833787,3.70248774
 33,-4.1827010711\C,-6.0563264821,3.9459933072,-3.2146332824\C,0.321146
 1695,-0.2815716064,2.4700835164\C,1.022756594,-0.5248546952,1.50204572
 38\H,3.3712538229,-1.7893088814,1.49962625\H,4.8026479771,-2.285799153
 4,-0.474934383\H,4.0683484436,-1.5735973211,-2.7549997656\H,1.90696878
 92,-0.3700649585,-3.0397834437\H,-3.4885794218,1.5009113553,4.40467322
 42\H,-2.7728949392,0.7986404955,6.6841034391\H,-0.6069192938,-0.409997
 7229,6.9888392141\H,0.824133854,-0.9061323186,5.0139505768\H,-8.404491
 0221,5.2110362594,-3.2120015085\H,-9.8356927238,5.7076929039,-1.237339
 2626\H,-9.1015200952,4.9950528231,1.0426230865\H,-6.9404513243,3.79089
 24915,1.3272043499\H,-1.5458047763,1.9185439591,-6.117340364\H,-2.2612
 989427,2.6210115784,-8.3967794347\H,-4.4268273408,3.8304495131,-8.7014
 802607\H,-5.8576704906,4.3271168347,-6.7265814863\\Version=EM64L-G09Re
 vE.01\State=1-A\HF=-1457.4490581\RMSD=7.474e-09\RMSF=1.320e-05\Dipole=
 0.0001393,0.0001275,0.0000765\Quadrupole=5.010199,-19.2849805,14.27478
 15,-18.4488854,-2.5411689,-4.7713217\PG=C01 [X(C38H16)]\\@
```


2


```
1\1\GINC-NODE381\FOpt\RB3LYP\CC-pVDZ\C42H16\ROOT\17-Oct-2017\0\\#opt f
 req b3lyp/cc-pvdz scf(maxcycle=250) CPHF(MaxInv=10000)\\something titl
 e\\0,1\C,1.604952038,-1.6856095237,-0.3898983248\C,1.2444655259,-2.252
 6340747,-1.6199331217\C,1.5332071192,-1.6001501197,-2.8482104828\C,0.5
 751275019,-3.5035781754,-1.6899736978\C,1.3163933738,-2.338195245,0.83
 83683756\C,2.2741201572,-0.4345763829,-0.3198445938\C,1.7533669526,-1.
 0890123009,-3.9377177953\C,-0.0015052144,-4.5751581847,-1.816346248\C,
 1.0964515835,-2.849446846,1.9278664831\C,2.8505074128,0.6371330799,-0.
 193543226\C,-0.6711678774,-5.8148326117,-2.0136348166\C,0.862930327,-
 3.4040947154,3.217051248\C,3.5198568328,1.8769746474,0.0038404371\C,1.
 9870849095,-0.5344621156,-5.2269074286\C,2.6398528015,0.7048137462,-5.
 3769687714\C,2.8674104746,1.2456169633,-6.6408470697\C,2.4465491346,0.
 5582362578,-7.786717873\C,1.7983072617,-0.669776229,-7.6674826446\C,1.
 5573890514,-1.2358957505,-6.4019176467\C,3.8238274386,2.3287561128,1.3
 307886654\C,4.4864263385,3.5580641847,1.5038530346\C,4.8467819944,4.33
 40362481,0.4036436683\C,4.5511660978,3.8953646493,-0.8935902648\C,3.89
 56778544,2.6814890557,-1.0897523202\C,0.2105598765,-4.6435780619,3.367
 1179518\C,-0.0168411888,-5.18443841,4.63099936\C,0.4037709311,-4.49690
```

26635,5.7768696922\C,1.0516397985,-3.2686940475,5.6576287374\C,1.29242
43449,-2.7025319817,4.3920558622\C,-0.9751381319,-6.2665824808,-3.3405
919585\C,-1.6380317934,-7.4957312631,-3.5136746399\C,-1.998687631,-8.2
715681185,-2.4134682042\C,-1.703127871,-7.832895223,-1.1162215747\C,-1
.047360044,-6.6191737469,-0.9200445969\C,1.9478060705,-1.4558753772,4.
2239140932\C,3.4386943898,1.5072260861,2.4210716149\C,0.9016926734,-2.
4823898783,-6.2337857886\C,-0.5897560587,-5.4451629527,-4.4308675077\C
,-0.1667735352,-4.5824667942,-5.1926229037\C,0.3702908084,-3.515557511
5,-5.8418587502\C,3.015878878,0.6444413553,3.1828184997\C,2.4789868706
,-0.4225879786,3.8320025899\H,2.9649691294,1.2348019667,-4.4806936192\
H,3.3752131415,2.2076264022,-6.7349412468\H,2.6251335086,0.9825516736,
-8.7768005586\H,1.467822026,-1.2105903746,-8.5555761876\H,4.713479426,
3.8935236478,2.5169298377\H,5.3604482223,5.2853108625,0.556690036\H,4.
8337147782,4.5037655412,-1.7550971106\H,3.6624904072,2.3340750987,-2.0
971374266\H,-0.1143435028,-5.1736992248,2.470843419\H,-0.5243272425,-6
.1466148197,4.7250977504\H,0.2252801056,-4.9212535446,6.7669542296\H,1
.3819440725,-2.7277623086,6.5457175762\H,-1.8650765406,-7.8311684764,-
4.5267601462\H,-2.5125713401,-9.2227245802,-2.566521005\H,-1.985964553
3,-8.4411633012,-0.2547151816\H,-0.8142491158,-6.2717421847,0.08735266
7\\Version=EM64L-G09RevE.01\State=1-A\HF=-1609.7624326\RMSD=8.174e-09\
RMSF=2.420e-06\Dipole=-0.0000478,0.000019,0.0000044\Quadrupole=-17.152
4734,14.8870446,2.2654288,22.3984257,-7.5829696,-17.0282549\PG=C01 [X(
C42H16)]\\@

3

1\1\GINC-NODE374\FOpt\RB3LYP\CC-pVDZ\C64H24\ROOT\20-Oct-2017\0\\#opt f
req b3lyp/cc-pvdz scf(maxcycle=250) CPHF(MaxInv=10000)\\something titl
e\\0,1\C,-4.2136298681,0.3847486703,-0.1277439319\C,-2.8409076321,0.34
68970127,0.1564700036\C,-2.1466647128,-0.8816753891,0.2905660603\C,-2.
0888776743,1.5354406426,0.3330500684\C,-4.9066609592,1.6173144951,-0.2
609895676\C,-4.9648317471,-0.8077957674,-0.3031948742\C,-1.5087758219,
-1.9158132764,0.4271799245\C,-1.4024528769,2.5324862022,0.5054594394\C
,-5.5581261845,2.644647299,-0.3931132902\C,-5.6646775189,-1.797550419,
-0.4701487635\C,-0.6409735637,3.713262876,0.7098748369\C,-6.3587930053
,3.8095527903,-0.553949746\C,-6.5201245314,-2.9164536024,-0.6706700729
\C,-0.8047130056,-3.1380687201,0.589213809\C,-1.5193804395,-4.34716852
42,0.7267866904\C,-0.8554187935,-5.5594808141,0.8876204103\C,0.5459449
2,-5.5934187741,0.9139609836\C,1.273360935,-4.4148009343,0.77927676\C,
0.6237069552,-3.1726621738,0.6160597444\C,-7.9188975669,-2.718990814,-
0.920075849\C,-8.7487482923,-3.8394972714,-1.1109650507\C,-8.225457315
3,-5.1302141441,-1.0628513306\C,-6.8588338807,-5.3261310603,-0.8242697
756\C,-6.017797026,-4.2321751973,-0.6307605038\C,-5.7940509656,5.09750
08005,-0.4684442079\C,-6.581524652,6.2365838202,-0.6230879542\C,-7.955
711362,6.1148067306,-0.8675194642\C,-8.5401567904,4.8531099128,-0.9604
572972\C,-7.7651466722,3.6881554619,-0.8092830086\C,0.7874489872,3.678
6634525,0.7366883081\C,1.4955235251,4.8818448813,0.9430243318\C,0.8250
867479,6.0883634318,1.119799577\C,-0.5762762295,6.1223050648,1.0935166
219\C,-1.2972239026,4.9494909588,0.8906324687\C,-8.3170080538,2.385156
5814,-0.9054982331\C,-8.4076694415,-1.3884939119,-0.9702192239\C,1.391
6931909,-1.9860611195,0.4816982894\C,1.4980044726,2.4622093807,0.55996
90789\C,2.1413616221,1.4329529776,0.4125640263\C,2.083597641,-0.984160
3716,0.3700337765\C,-8.5875177943,-0.1755736605,-0.9780647124\C,-8.554
926491,1.1832566483,-0.9545157237\C,2.8410604614,0.2092388971,0.263209
2524\C,4.2236970404,0.1802932251,0.0307310714\C,4.9801587279,1.3777173
409,-0.0751522576\C,4.9222797262,-1.0474006349,-0.1176118477\C,5.57908
14523,-2.0700335246,-0.2594151623\C,5.6851140546,2.3721790964,-0.18171
98971\C,6.3863053864,-3.2291915928,-0.4287955109\C,6.54670245,3.496827
7472,-0.3112371913\C,7.9540778497,3.3073366325,-0.5140749553\C,8.78964
30708,4.4333177057,-0.6349433739\C,8.2638868155,5.7218895189,-0.562402
2214\C,6.8890461545,5.910194567,-0.369220549\C,6.0421579417,4.81066014
52,-0.2453676964\C,5.8197562313,-4.5190291914,-0.4090604029\C,6.613543
3339,-5.6525625965,-0.5719558896\C,7.9960305747,-5.5231986189,-0.75894

56485\C,8.5825880611,-4.2593532095,-0.7861837755\C,7.8013546577,-3.099
8506135,-0.6253991607\C,8.4454684901,1.979093742,-0.5910731008\C,8.355
4655623,-1.7946135076,-0.6557721678\C,8.6263893884,0.7669951091,-0.631
8166545\C,8.5940153917,-0.5918369363,-0.6555520666\H,-2.6094338497,-4.
3090661047,0.714028272\H,-1.4289753066,-6.4822790723,0.9956899899\H,1.
0697101145,-6.5427906462,1.0426692466\H,2.3636900729,-4.4294942174,0.8
0750491\H,-9.8112963904,-3.6788100486,-1.2996888994\H,-8.8840413907,-5
.9879067445,-1.2137299877\H,-6.4482911395,-6.3373354957,-0.7902143377\
H,-4.9518642784,-4.3787229082,-0.4508194766\H,-4.7225092666,5.18644387
66,-0.2841698573\H,-6.1230469653,7.2251499149,-0.5539836971\H,-8.57231
10396,7.0078040959,-0.9879128927\H,-9.6089701645,4.7502180416,-1.15409
92948\H,2.5852882743,4.8434766331,0.9708237495\H,1.3934688322,7.006524
5026,1.2814308026\H,-1.1052140219,7.0670458222,1.2345588938\H,-2.38784
53678,4.9639372079,0.8775424996\H,9.8586969461,4.2786850031,-0.7887587
103\H,8.9270348726,6.5839169152,-0.6587211358\H,6.476693333,6.91983926
81,-0.3160804498\H,4.9700521734,4.9514782117,-0.1010561511\H,4.7419858
154,-4.6136778815,-0.2693510535\H,6.1535749438,-6.6426925415,-0.554277
2552\H,8.6175309895,-6.4118820926,-0.8858839848\H,9.6579857647,-4.1504
291867,-0.9348446023\\Version=EM64L-G09RevE.01\State=1-A\HF=-2452.7547
306\RMSD=9.585e-09\RMSF=1.149e-05\Dipole=-0.0028007,-0.0025721,0.14497
88\Quadrupole=-13.6508988,54.205446,-40.5545473,1.6124229,0.544284,1.6
484986\PG=C01 [X(C64H24)]\\@

4a

1\1\GINC-NODE663\FOpt\RB3LYP\CC-pVDZ\C32H12\FREJASTORM\26-Jul-2018\0\\
opt freq=noraman b3lyp/cc-pvdz scf(maxcycle=600) CPHF(MaxInv=10000)\
\something title\\0,1\C,0.6179699353,0.0198940866,0.4915252691\C,1.351
6638077,-1.1939864275,0.4279511494\C,1.3576760426,1.2301147958,0.42779
6258\C,2.0268859907,2.2505064582,0.3701105462\C,2.0157899367,-2.217700
1914,0.3703808031\C,2.7638935705,3.463668796,0.3067686247\C,2.74655161
95,-3.4346244237,0.3068411873\C,4.1692868655,-3.4382644588,0.183753843
4\C,4.8477810621,-4.6735906008,0.1247597058\C,4.14995894,-5.8766000308
,0.1848813145\C,2.753401941,-5.8730272374,0.305746023\C,2.0617452622,-
4.6664624134,0.3658615102\C,2.085408563,4.6990001265,0.3657778021\C,2.
7832536106,5.9020041731,0.305816826\C,4.1798252641,5.8984199754,0.1851
198657\C,4.8714733961,4.6918500953,0.1250079344\C,4.1866433064,3.46001
75554,0.1838496522\C,4.9062744122,-2.2250963076,0.1202726512\C,4.91740
23268,2.2430866979,0.1203838482\C,5.5755371476,-1.2047420917,0.0625604
582\C,5.5814708357,1.2193443263,0.0626695043\H,5.934552244,-4.66501777
8,0.0307308267\H,4.6944038196,-6.8219006092,0.1375674112\H,2.204129876
4,-6.8155301093,0.3530802695\H,0.9750341916,-4.6523260495,0.4599109104
\H,0.9986273828,4.6904327066,0.4596893604\H,2.2388179818,6.8473096534,
0.3531384975\H,4.7291180375,6.8409179023,0.1379270213\H,5.9581974036,4
.6777080956,0.0311062964\C,6.3152069595,0.0055003676,-0.0011287446\C,7
.7038733411,0.0021065451,-0.1212235087\C,8.4497131835,1.2147405461,-0.
1853852703\C,8.4437715716,-1.2141565531,-0.1854910101\C,9.1241002594,2
.2249215205,-0.2434339835\H,9.7147935589,3.1184186013,-0.2942378543\C,
9.1132107242,-2.2276176665,-0.2436387291\H,9.699482328,-3.1240123058,-
0.2946189706\C,-0.7707078798,0.0233464115,0.6115135872\C,-1.5105596633
,1.2396407611,0.6757526865\C,-1.5166095142,-1.1892564916,0.6756273265\
C,-2.1800037131,2.2531040374,0.7338464163\H,-2.7665969965,3.1493020642
,0.7846121799\C,-2.1910960487,-2.1993761975,0.7336255306\H,-2.78241288
41,-3.0924583558,0.7844949738\\Version=ES64L-G16RevA.03\State=1-A\HF=-
1226.1828403\RMSD=8.853e-09\RMSF=3.888e-06\Dipole=-0.0003516,0.000053,
0.0000312\Quadrupole=-2.2011695,25.8993663,-23.6981968,0.071119,-1.871
8826,0.0057972\PG=C01 [X(C32H12)]\\@

5

1\1\GINC-NODE629\FOpt\RB3LYP\CC-pVDZ\C54H20\FREJASTORM\25-Jul-2018\0\\
opt freq=noraman b3lyp/cc-pvdz scf(maxcycle=600) CPHF(MaxInv=10000)\
\something title\\0,1\C,2.7496588751,0.0535115309,-0.1883069357\C,1.99
95661979,1.2528074562,-0.0681272284\C,2.0371359228,-1.1683921935,-0.06
72562103\C,1.318580382,2.2594190876,0.0558578526\C,1.3877148328,-2.195

5602324,0.0574733505\C,0.6776453139,-3.4155374636,0.2167136586\C,0.570
8704128,3.4567896663,0.2144040285\C,1.2431617549,4.6942318916,0.298925
617\C,0.5383672668,5.8823408169,0.4687172905\C,-0.8604004773,5.8605009
68,0.5596528492\C,-1.5459360328,4.6523506686,0.4735544549\C,-0.8551974
822,3.4343552431,0.2969619281\C,-0.7484328628,-3.4374661737,0.29921032
92\C,-1.4009209426,-4.6762684885,0.4765389387\C,-0.6781041149,-5.86243
41836,0.5634077671\C,0.7206701583,-5.8407664681,0.4725303191\C,1.38813
74518,-4.6313934282,0.3020256793\C,-1.5797020401,2.2164911281,0.201565
4216\C,-1.5104983964,-2.2428085757,0.2030396849\C,-2.196138322,-1.2338
490686,0.1173436674\C,-2.233634774,1.1866442021,0.1165204986\C,-2.9482
560538,-0.0349914938,0.0336919237\C,-4.3435051498,-0.0566529894,-0.112
790621\C,-5.0581317497,-1.2782863292,-0.1956231253\C,-5.095621994,1.14
22066672,-0.196419273\C,-5.781192602,2.1512060219,-0.2822020358\C,-5.7
121245949,-2.3080895817,-0.2807394822\C,-6.5432014984,3.3458853097,-0.
3785538003\C,-6.4367002025,-3.5259037764,-0.3762495255\C,-7.8627691624
,-3.5482688221,-0.2936835243\C,-8.5351299822,-4.7856653357,-0.37832957
45\C,-7.8304025922,-5.9737943905,-0.5482512615\C,-6.4316334599,-5.9520
219025,-0.6391945941\C,-5.7460305122,-4.7439194555,-0.5529745192\C,-5.
890659709,4.5846319015,-0.5560781262\C,-6.6134265105,5.7708141178,-0.6
431265441\C,-8.012202365,5.7492202196,-0.5522375027\C,-8.6797207124,4.
5399036045,-0.3815374868\C,-7.9692785278,3.3240312688,-0.2960390627\C,
-8.6104138473,-2.3508757785,-0.1350013496\C,-8.6793930919,2.1041081967
,-0.1365835098\C,-9.2914154982,-1.3442873043,-0.0109180231\C,-9.328767
0708,1.0769134024,-0.0118246758\H,2.3319938451,4.6983254141,0.23210837
4\H,1.0784579155,6.8290188293,0.5339285006\H,-1.4158679724,6.790180819
7,0.6992461594\H,-2.6336358847,4.6260346055,0.5510824568\H,-2.48891632
1,-4.6837841948,0.5540195446\H,-1.204361904,-6.8088721338,0.7035613994
\H,1.2899722409,-6.7701280229,0.5383478343\H,2.4765728309,-4.601622444
3,0.235251284\H,-9.6239619808,-4.7897046627,-0.311505557\H,-8.37054424
19,-6.9204366686,-0.6135584457\H,-5.8762200456,-6.8817185402,-0.778890
7269\H,-4.658330008,-4.7176539216,-0.6305097316\H,-4.8026640418,4.5920
872726,-0.6335641032\H,-6.0871308243,6.7172087375,-0.7834301856\H,-8.5
814635074,6.6785968048,-0.6181958998\H,-9.7681566802,4.5101883155,-0.3
14746944\C,-10.0413967096,-0.1449234668,0.1092657117\C,-11.4186890026,
-0.1660888342,0.321969508\C,-12.1736102252,1.0371923036,0.4359476915\C
,-12.1361542357,-1.3919998977,0.4368632545\C,-12.8541615818,2.03955032
63,0.5398547679\H,-13.4503246352,2.9259642298,0.6329631051\C,-12.78564
32044,-2.4146703854,0.5414859027\H,-13.3543941403,-3.318868101,0.63507
12533\C,4.1269472156,0.0748056055,-0.4010463238\C,4.8442753846,1.30077
53074,-0.5160513736\C,4.8819661077,-1.128401486,-0.5151886933\C,5.4934
881599,2.3236157933,-0.6208201083\H,6.061952267,3.227984134,-0.7144992
058\C,5.5625822213,-2.1306947628,-0.619239034\H,6.1588127208,-3.017060
91,-0.7123676827\\Version=ES64L-G16RevA.03\State=1-A\HF=-2069.1747929\
RMSD=5.568e-09\RMSF=2.864e-06\Dipole=-0.0001088,0.000017,-0.0002649\Qu
adrupole=-4.462269,42.2766848,-37.8144158,-0.7263752,-0.5123948,-0.033
9022\PG=C01 [X(C54H20)]\\@

6

1\1\GINC-NODE627\FOpt\RB3LYP\CC-pVDZ\C76H28\FREJASTORM\26-Jul-2018\0\\
#opt freq=noraman b3lyp/cc-pvdz scf(maxcycle=600) CPHF(MaxInv=10000)\\
something title\\0,1\C,4.1774624159,-0.0125862622,-0.0246224475\C,2.81
29103781,-0.0212378977,0.3007345639\C,2.0873891731,1.1832908312,0.4803
789577\C,2.1039330285,-1.2348162438,0.4854521255\C,4.9011717032,-1.218
9465404,-0.1987561846\C,4.8846067173,1.2028191283,-0.2038441049\C,1.42
2917441,2.1931595005,0.6646992859\C,1.4533469967,-2.2529074831,0.67402
3935\C,5.5580817028,-2.2366835987,-0.366152717\C,5.5275467327,2.228728
6236,-0.3755387267\C,0.7310710528,-3.4541766815,0.9011640191\C,6.28766
28289,-3.440643155,-0.5527956253\C,6.240610112,3.4417314478,-0.5673926
539\C,0.6842357609,3.3853368755,0.886927193\C,1.3628693066,4.607105151
9,1.0841496038\C,0.6635043105,5.7895028011,1.3067354674\C,-0.738161158
5,5.7799563278,1.337544038\C,-1.4304674874,4.5880799855,1.145548584\C,
-0.7445841767,3.3756049719,0.9183338334\C,7.6621791339,3.4507967281,-0

.7093132779\C,8.32574446,4.6834424521,-0.8844210535\C,7.615408159,5.87
95856309,-0.9271129972\C,6.2195780566,5.8709620054,-0.7964152075\C,5.5
432406524,4.6677544717,-0.6180510313\C,5.6071103167,-4.6762842086,-0.5
982120268\C,6.2998337183,-5.8708920688,-0.7714196606\C,7.6956599928,-5
.8610050252,-0.9020698128\C,8.3895864794,-4.6550975338,-0.8644928747\C
,7.7092322045,-3.4308942061,-0.694665709\C,-0.6977497763,-3.4639107545
,0.9325283963\C,-1.3669538596,-4.6847286541,1.1647522986\C,-0.65837832
46,-5.866195132,1.3616717789\C,0.7432881092,-5.856645618,1.33090373\C,
1.4263849107,-4.6656979079,1.1034345551\C,8.4432745831,-2.2148927879,-
0.6731123869\C,8.4127662766,2.24504058,-0.682605476\C,-1.4760173678,2.
1734140722,0.7284236391\C,-1.4455895147,-2.2726582956,0.7376506274\C,-
2.1176134572,-1.2635604291,0.5781607505\C,-2.1341465215,1.1545489202,0
.5731455397\C,9.0937413327,1.2307999144,-0.6757899126\C,9.1103219146,-
1.1914187091,-0.6706319643\C,-2.8504295061,-0.0597715746,0.425131006\C
,-4.2279399852,-0.0697388757,0.1599902187\C,-4.9421482532,-1.285862188
8,0.0173961946\C,-4.9587060855,1.1359043648,0.0123861542\C,-5.62241602
48,2.152954207,-0.1305823125\C,-5.591898174,-2.3124666651,-0.121345505
7\C,-6.3595634624,3.3561557063,-0.2904632604\C,-6.3126066052,-3.526236
7527,-0.276215447\C,-7.7390349941,-3.5357648592,-0.3555722243\C,-8.409
5548533,-4.7691052981,-0.4958141299\C,-7.7016895131,-5.9655209599,-0.5
642209226\C,-6.3014711633,-5.9564825889,-0.4949362496\C,-5.6180451665,
-4.7525641719,-0.3518729694\C,-5.6817490278,4.5914969041,-0.3712014483
\C,-6.3814871268,5.7854027894,-0.5192327925\C,-7.7816959863,5.77510088
56,-0.5885335931\C,-8.4732194524,4.5694575421,-0.5151681137\C,-7.78598
5486,3.3459456917,-0.3698370028\C,-8.4878133202,-2.3298048528,-0.30140
44147\C,-8.5182859057,2.1301425058,-0.3106650318\C,-9.1679101651,-1.31
54599458,-0.2693205547\C,-9.1844942874,1.1067630075,-0.2743778791\H,2.
453737543,4.6030768972,1.070474529\H,1.2098710074,6.7221785231,1.46114
73561\H,-1.2898631675,6.7051529078,1.5160933694\H,-2.520727065,4.56919
62099,1.1798155569\H,9.4115389889,4.6774500468,-0.9895828974\H,8.14837
98828,6.8224392529,-1.0654406186\H,5.6590207921,6.807243535,-0.8352307
535\H,4.4566877237,4.6517924299,-0.5224608611\H,4.5204342112,-4.674758
2136,-0.5027002538\H,5.7521220926,-6.8149017646,-0.806232312 7\H,8.2414
715514,-6.7970728479,-1.0363572897\H,9.4752046405,-4.6347222018,-0.969
6724953\H,-2.4573717776,-4.6806568994,1.1989414016\H,-1.1973505821,-6.
7981250219,1.5440430692\H,1.3023850363,-6.7810947205,1.4891697332\H,2.
5170962233,-4.6467659367,1.089745343\H,-9.4989183261,-4.763453524,-0.5
532600905\H,-8.2401524715,-6.9089215167,-0.6747618405\H,-5.7430864336,
-6.8929993857,-0.5540716018\H,-4.5283455088,-4.7363098482,-0.304125962
6\H,-4.5919310824,4.5902688782,-0.3234018713\H,-5.8358945973,6.7291782
059,-0.5822490232\H,-8.332941571,6.7106219769,-0.7029668259\H,-9.56240
28835,4.548745861,-0.5725715789\C,-9.9164210773,-0.1093676543,-0.24768
25542\C,-11.3099022199,-0.118837709,-0.2142473637\C,-12.0632369208,1.0
907060174,-0.198274693\C,-12.0466164785,-1.338503929,-0.1931911308\C,-
12.7434416167,2.0985630498,-0.1829117595\H,-13.3395050148,2.9897875181
,-0.1678284822\C,-12.7130101303,-2.3554752804,-0.1735633669\H,-13.2968
326492,-3.2546952748,-0.15472773\C,9.8425971805,0.0247420564,-0.681563
7352\C,11.2362019384,0.0342225136,-0.7092951173\C,11.9730055265,1.2538
981786,-0.7260786076\C,11.9896344512,-1.1753124623,-0.720904754\C,12.6
394684528,2.2709108574,-0.7403039823\H,13.2234612851,3.1701520024,-0.7
511421797\C,12.6699640089,-2.1831534229,-0.7308178278\H,13.266225265,-
3.0743454028,-0.7378275901\\Version=ES64L-G16RevA.03\State=1-A\HF=-291
2.1671754\RMSD=9.613e-09\RMSF=1.019e-06\Dipole=-0.0002245,-0.0000154,-
0.0104038\Quadrupole=-8.1199172,59.1151243,-50.9952072,-0.4613581,-0.9
402565,-0.2371432\PG=C01 [X(C76H28)]\\@

14A

1\1\GINC-NODE364\FOpt\RB3LYP\CC-pVDZ\C64H28\ROOT\20-Jan-2018\0\\# opt
b3lyp/cc-pvdz scf(maxcycle=600) CPHF(MaxInv=10000)\\something title\\0
,1\C,-4.1488172003,0.1311231782,-0.0777703374\C,-2.7494235751,0.100846
6975,-0.025290221\C,-1.9897884156,1.2983253629,-0.0465749676\C,-2.0344
16101,-1.1208291189,0.0398443827\C,-4.9367251121,-1.0470837056,0.01114

12243\C,-4.8595407765,1.3489026464,-0.2355063058\C,-1.303612255,2.3102
233995,-0.0398683941\C,-1.373977296,-2.1495380106,0.0632943926\C,-5.69
33176922,-2.0035733249,0.0970645128\C,-5.5331141028,2.3575250927,-0.38
99604082\C,-0.6522840656,-3.3719957596,0.0848363277\C,-6.6252152238,-3
.0781963414,0.1498614578\C,-6.2952587385,3.5417627221,-0.5841797568\C,
-0.5472337223,3.5119695799,-0.0315004422\C,-1.2110788277,4.7574622195,
-0.0308057992\C,-0.4961035617,5.9516804101,-0.0308739811\C,0.905653761
4,5.9299356557,-0.031964237\C,1.5851628058,4.7147823471,-0.0277999589\
C,0.8817909023,3.4907339873,-0.0237998782\C,-7.6943007706,3.5697927564
,-0.3075274275\C,-8.4101746118,4.7632133423,-0.5173797646\C,-7.7723792
888,5.9089458169,-0.9897708163\C,-6.3992387826,5.8827564373,-1.2641000
715\C,-5.6710617661,4.7131942414,-1.0633055208\C,-7.8283527073,-2.9827
096942,-0.582576074\C,-8.7639647979,-4.0134992062,-0.5621610381\C,-8.5
205840338,-5.166156807,0.19543839\C,-7.3436007242,-5.278166502,0.93258
55851\C,-6.3835721138,-4.2480469634,0.9273274045\C,0.7770413297,-3.391
8626925,0.0749489485\C,1.4445469809,-4.6353645136,0.1026582125\C,0.729
308557,-5.829806986,0.1349540397\C,-0.6723374688,-5.8102486978,0.14006
44365\C,-1.3542469858,-4.5963542722,0.1143676956\C,-5.1972382438,-4.38
17508532,1.7134363967\C,-8.3700082266,2.4086958094,0.1812381592\C,1.60
18070188,2.2668891304,-0.0089063392\C,1.5329583586,-2.1903302605,0.035
0311399\C,2.2244077639,-1.1825451425,0.001281586\C,2.2618150956,1.2379
126969,0.0056172368\C,-8.9595585314,1.4326612604,0.6003314967\C,-4.204
989779,-4.5138112725,2.4024454196\C,2.9816366872,0.0161756931,-0.00570
41282\C,4.3813420513,-0.0045280252,-0.0222695858\C,5.1166551848,-1.217
073444,-0.1084661946\C,5.153391269,1.186423549,0.0453973646\C,5.890417
9379,2.15958061,0.116268802\C,5.8241421994,-2.2104982337,-0.1976894115
\C,6.7898902113,3.2625872323,0.1571951125\C,6.6922056244,-3.3371046086
,-0.2626059878\C,6.3536477447,-4.5045034274,-1.007310515\C,7.252610655
3,-5.5880165003,-1.0301707927\C,8.4623085306,-5.5315862888,-0.34124867
21\C,8.800093468,-4.38278905,0.385130783\C,7.9261124903,-3.2998105903,
0.4217862355\C,7.9944737992,3.2004999328,-0.5757613939\C,8.8971742293,
4.2602862743,-0.5639356237\C,8.6183652578,5.4100163153,0.1855635754\C,
7.4386360429,5.4905094114,0.9224164188\C,6.5116290296,4.4306616141,0.9
253485357\C,5.1296235452,-4.5842131398,-1.7410448395\C,5.3202466385,4.
5341802156,1.7083636799\C,4.1008713389,-4.6719579651,-2.3818116233\C,4
.3209004413,4.6419070133,2.391274625\H,-2.3019482061,4.7642781403,-0.0
240475398\H,-1.0310040562,6.903748005,-0.0299380169\H,1.4702103231,6.8
646492261,-0.0338065933\H,2.6750681447,4.6884058734,-0.0189884388\H,-9
.4796717348,4.7746017393,-0.3018575799\H,-8.3470882951,6.824156421,-1.
1457117191\H,-5.8964947213,6.7770937914,-1.6380604893\H,-4.6030913708,
4.6802784471,-1.2841399485\H,-8.0077339791,-2.0814151477,-1.1707494791
\H,-9.6857809129,-3.9199208727,-1.1401135847\H,-9.2511652132,-5.977443
5862,0.2134800884\H,-7.1488167952,-6.1690066133,1.5316643684\H,2.53470
47751,-4.6411255581,0.0906524094\H,1.2665545123,-6.7803727452,0.154572
2094\H,-1.2356620486,-6.745525822,0.1642054166\H,-2.4443572533,-4.5712
987229,0.1225435559\H,-9.4620200701,0.563051373,0.9760189016\H,-3.3243
983682,-4.607727889,3.0072958924\H,6.9837426547,-6.4761004305,-1.60414
18413\H,9.1445714802,-6.3835218597,-0.3724269243\H,9.7477382964,-4.332
6167313,0.9253767486\H,8.1799592347,-2.4019682147,0.9872264638\H,8.202
3666383,2.3022386153,-1.1590055871\H,9.8211664719,4.191201126,-1.14175
71028\H,9.3232971022,6.2437864752,0.1973565936\H,7.2158758432,6.379321
7181,1.5147211563\H,3.188441258,-4.7270516993,-2.9426546748\H,3.434244
8377,4.7144936876,2.9901626458\\Version=ES64L-G16RevA.03\State=1-A\HF=
-2455.108927\RMSD=7.173e-09\RMSF=8.698e-07\Dipole=0.3028449,0.0815057,
-0.143626\Quadrupole=21.6379972,20.324081,-41.9620783,2.4660764,3.7097
505,-2.7326998\PG=C01 [X(C64H28)]\\@

14B

1\1\GINC-NODE356\FOpt\RB3LYP\CC-pVDZ\C64H28\ROOT\20-Jan-2018\0\\# opt
b3lyp/cc-pvdz scf(maxcycle=600) CPHF(MaxInv=10000)\\something title\\0
,1\C,4.0943114986,0.000001744,0.0000265616\C,2.6919760728,-0.000001400

3,0.0000024459\C,1.9545817736,-1.2092101771,-0.0610273831\C,1.95457525
63,1.2092050383,0.0610041635\C,4.8306649307,1.2040027821,0.1438840835\
C,4.8306765617,-1.2039951424,-0.1438057389\C,1.2837428946,-2.231271313
,-0.0853253139\C,1.2837330738,2.2312645786,0.0852760179\C,5.4968431173
,2.219701083,0.2822841773\C,5.496861364,-2.219690897,-0.2821925632\C,0
.5440739703,3.4430022422,0.1101697149\C,6.2355209539,3.4215530771,0.46
30804966\C,6.2355294386,-3.4215473961,-0.4629989258\C,0.5440809669,-3.
4430068241,-0.1102345308\C,1.2245850493,-4.6792408508,-0.1381993454\C,
0.5257473935,-5.882511376,-0.1703707978\C,-0.8761740469,-5.8797479812,
-0.1752091724\C,-1.5719742995,-4.6743099996,-0.1423710134\C,-0.8852420
989,-3.4413532887,-0.1058846277\C,7.6017635405,-3.5180624999,-0.065100
1688\C,8.2894556976,-4.7297617952,-0.2647550409\C,7.6559081373,-5.8275
778237,-0.844357305\C,6.3152986964,-5.7339814616,-1.238610245\C,5.6150
930127,-4.5454908555,-1.0492308986\C,5.6150682746,4.5455325174,1.04922
67034\C,6.3152823617,5.7340200717,1.2385940029\C,7.6559164097,5.827577
1995,0.8444152116\C,8.2894799454,4.7297253642,0.2648983554\C,7.6017796
242,3.5180284147,0.0652569198\C,-0.8852488357,3.4413533179,0.105811751
9\C,-1.5719770329,4.674312185,0.1422853366\C,-0.8761732407,5.879748141
6,0.1751157889\C,0.5257481213,5.8825074175,0.1702814014\C,1.2245821746
,4.67923436,0.1381245679\C,8.2704978073,2.4051565118,-0.5319854008\C,8
.270465226,-2.4052277306,0.5322297232\C,-1.6223069797,-2.2284939306,-0
.0606293019\C,-1.6223167623,2.2284956451,0.0605595463\C,-2.2977649639,
1.210277515,0.019367258\C,-2.2977562609,-1.2102766765,-0.0194327106\C,
8.8451150575,-1.46366664,1.0414192088\C,8.8451618266,1.4635624759,-1.0
410979869\C,-3.0363211396,-0.0000023918,-0.0000280842\C,-4.4362798802,
-0.000008333,-0.0000229472\C,-5.1900889856,1.2030822653,-0.054705163\C
,-5.1900762831,-1.2031061794,0.0546631878\C,-5.9125700605,-2.187749289
3,0.1162778051\C,-5.9125887472,2.1877212415,-0.1163136983\C,-6.7965158
133,-3.303480118,0.1479208852\C,-6.7965078221,3.3034743805,-0.14792603
31\C,-6.4894967501,4.4843869691,-0.8848952566\C,-7.402490827,5.5562762
584,-0.8741996093\C,-8.5958171722,5.4754101518,-0.1596141998\C,-8.9027
205051,4.3132981122,0.559203517\C,-8.0143680828,3.2414158956,0.5627838
187\C,-8.0144419876,-3.2413554372,-0.5626700988\C,-8.9028233197,-4.313
2135575,-0.5590541267\C,-8.5958838241,-5.4753663839,0.1596822777\C,-7.
4024909454,-5.5562988377,0.8741490512\C,-6.4894648138,-4.4844365154,0.
8848042475\C,-5.2833095885,4.589055904,-1.6446689689\C,-5.2832049469,-
4.5891749593,1.6444525291\C,-4.2706384921,4.6981620416,-2.3074194647\C
,-4.2704739717,-4.6983392866,2.3071020843\H,2.3153671945,-4.6720891497
,-0.1279980105\H,1.0735613811,-6.8269640144,-0.1914620303\H,-1.4280261
519,-6.8216185864,-0.20212107\H,-2.6621244758,-4.662786079,-0.13555129
54\H,9.3337247172,-4.7936880865,0.044836882\H,8.2085351902,-6.75791082
19,-0.9903570846\H,5.8162575076,-6.5901535567,-1.6972655189\H,4.573645
231,-4.4596994178,-1.3630485057\H,4.5736004926,4.4597724153,1.36298632
81\H,5.816228291,6.5902205481,1.6971822801\H,8.2085501157,6.7579076383
,0.9904062063\H,9.333767967,4.7936210784,-0.0446357658\H,-2.6621272879
,4.6627917977,0.1354635943\H,-1.4280226791,6.8216206561,0.2020176837\H
,1.0735648611,6.8269586475,0.1913631836\H,2.3153644221,4.6720792912,0.
1279266628\H,9.3255174488,-0.6140607473,1.4857168995\H,9.3255744598,0.
613915003,-1.4853050495\H,-7.1578557919,6.4548762245,-1.4426060225\H,-
9.2894503137,6.3186613784,-0.1648836816\H,-9.837452474,4.243976331,1.1
194354416\H,-8.2441574755,2.3333537417,1.122136659\H,-8.2442600811,-2.
3332612476,-1.1219590771\H,-9.8376067329,-4.2438407605,-1.1191938612\H
,-9.2895406342,-6.3185979904,0.1649810838\H,-7.1578269486,-6.454931538
5,1.4424912705\H,-3.3724438535,4.7723433816,-2.8886600056\H,-3.3722761
29,-4.7725336901,2.8883360841\\Version=ES64L-G16RevA.03\State=1-A\HF=-
2455.1090165\RMSD=7.283e-09\RMSF=3.732e-06\Dipole=-0.608906,0.0000191,
0.0000452\Quadrupole=15.2304967,26.5182606,-41.7487572,-0.0006494,-0.0
005855,5.8720236\PG=C01 [X(C64H28)]\\@

14C

1\1\GINC-NODE356\FOpt\RB3LYP\CC-pVDZ\C64H28\ROOT\21-Jan-2018\0\\# opt

b3lyp/cc-pvdz scf(maxcycle=600) CPHF(MaxInv=10000)\\something title\\0
,1\C,-4.0936377792,-0.0001647764,0.0001289231\C,-2.6913277075,-0.00017
43153,0.0001010211\C,-1.9538748992,1.2070141221,-0.092207404\C,-1.9538
765506,-1.2073643971,0.0924044051\C,-4.8298353599,-1.2127554679,0.0127
429586\C,-4.8298094493,1.2124419633,-0.0124640829\C,-1.2826249148,2.22
36394379,-0.1976956649\C,-1.2825838325,-2.2239614169,0.1978956746\C,-5
.4954218031,-2.2382110411,0.0080067689\C,-5.4953079426,2.237954454,-0.
0077291516\C,-0.5425579246,-3.4279508089,0.3351734158\C,-6.2330901148,
-3.453998025,-0.0141951871\C,-6.2326289217,3.453954012,0.0143661017\C,
-0.542746571,3.4277188227,-0.3349760345\C,-1.2229632334,4.653697359,-0
.4975846996\C,-0.523962767,5.8494667458,-0.6347378529\C,0.8778001972,5
.8492165405,-0.6132514525\C,1.5733859196,4.6529542237,-0.4603828102\C,
0.8865107873,3.4274507872,-0.3204311026\C,-7.5991721209,3.498977698,-0
.391493123\C,-8.2853633178,4.7272246104,-0.3521625857\C,-7.6500423627,
5.8909515321,0.0774798332\C,-6.3090303951,5.8484313643,0.4790913884\C,
-5.610269265,4.644536654,0.4468721832\C,-5.6110930675,-4.6447054138,-0
.4468786235\C,-6.310196708,-5.8483986078,-0.4791990162\C,-7.6511971226
,-5.8905870565,-0.0775137677\C,-8.2861636034,-4.7267311319,0.352303038
7\C,-7.5996224947,-3.4986831981,0.3917375649\C,0.8866988052,-3.4275237
85,0.3205284977\C,1.5737205693,-4.6529445065,0.4604865862\C,0.87827988
86,-5.8492779709,0.6134570058\C,-0.5234813237,-5.8496837506,0.63504247
92\C,-1.222624876,-4.6539985985,0.4978854596\C,-8.2697213988,-2.317833
8215,0.8377276768\C,-8.2696319692,2.3182651946,-0.8373034045\C,1.62346
87796,2.2217072089,-0.1811527202\C,1.623514868,-2.2217052674,0.1811462
596\C,2.2990967292,-1.2076390712,0.0811368834\C,2.2991298306,1.2076859
061,-0.0812244645\C,-8.8458387234,1.3190012639,-1.2187309635\C,-8.8456
209237,-1.3184513613,1.2193086484\C,3.0376693288,0.0000118331,-0.00006
9439\C,4.4377297978,-0.0000088729,-0.0001001948\C,5.1913398224,-1.2043
560448,0.0050815588\C,5.1913763258,1.2043149189,-0.0053167706\C,5.9132
4109,2.1912884563,0.0072048722\C,5.9131744962,-2.1913510607,-0.0074807
808\C,6.7971367947,3.3071500694,-0.0154806266\C,6.7970540738,-3.307226
8762,0.0151444781\C,6.4802146432,-4.5301980439,-0.6451417041\C,7.39412
08775,-5.5995890922,-0.5836200003\C,8.5977007483,-5.4761985827,0.10729
55506\C,8.9140987287,-4.2731883727,0.7507951333\C,8.0250652456,-3.2029
555351,0.7031798619\C,8.0251776888,3.2027990771,-0.7034514371\C,8.9142
295623,4.2730140311,-0.7511212013\C,8.5978206395,5.4760862163,-0.10774
24839\C,7.3942112549,5.5995563479,0.5831071924\C,6.4802858274,4.530184
555,0.6446822689\C,5.2629775183,-4.6802927046,-1.379195234\C,5.2630137
108,4.6803650705,1.3786600909\C,4.2405633811,-4.8288291994,-2.01889862
96\C,4.2405747447,4.8289840673,2.0183046358\H,-2.3135218201,4.64349315
83,-0.5219653212\H,-1.07150921,6.7857326877,-0.7617821201\H,1.42970788
84,6.7853792664,-0.7199578211\H,2.6634365108,4.6429865284,-0.442784387
4\H,-9.3298888358,4.7511491866,-0.6665461923\H,-8.201528719,6.83306485
57,0.100399634\H,-5.808502646,6.7569697817,0.8203950035\H,-4.568283376
7,4.5996472165,0.7673769138\H,-4.5691146249,-4.6000721766,-0.767442592
\H,-5.8099462559,-6.7570380541,-0.820640247\H,-8.2029513792,-6.8325415
819,-0.1005127978\H,-9.3306773673,-4.7503973578,0.6667451546\H,2.66376
89355,-4.642855367,0.4428133924\H,1.4302997431,-6.7853742121,0.7201669
059\H,-1.0709141603,-6.7860050341,0.7621683149\H,-2.3131823922,-4.6439
151664,0.5223468251\H,-9.327654664,0.4194289741,-1.5483585916\H,-9.327
1601532,-0.4187719717,1.5490486381\H,7.1418757795,-6.5306315784,-1.093
479169\H,9.2918394103,-6.3183239919,0.1423964284\H,9.8568092429,-4.170
5719528,1.2922440644\H,8.2622471207,-2.2630789551,1.2038189531\H,8.262
3679719,2.2628741489,-1.2039957734\H,9.8569632487,4.1703351668,-1.2925
179553\H,9.2919735747,6.3181980596,-0.1428866701\H,7.1419565918,6.5306
482684,1.0928713201\H,3.3337588756,-4.9369738527,-2.5810772302\H,3.333
8574791,4.9373075851,2.5805895175\\Version=ES64L-G16RevA.03\State=1-A\
HF=-2455.1090553\RMSD=4.583e-09\RMSF=1.607e-06\Dipole=0.6111994,0.0000
361,0.0000067\Quadrupole=15.4854728,28.1461328,-43.6316056,0.0009011,0
.0011382,3.4694276\PG=C01 [X(C64H28)]\\@

14D

1\1\GINC-NODE356\FOpt\RB3LYP\CC-pVDZ\C64H28\ROOT\21-Jan-2018\0\\# opt
b3lyp/cc-pvdz scf(maxcycle=600) CPHF(MaxInv=10000)\\something title\\0
,1\C,-4.0936377792,-0.0001647764,0.0001289231\C,-2.6913277075,-0.00017
43153,0.0001010211\C,-1.9538748992,1.2070141221,-0.092207404\C,-1.9538
765506,-1.2073643971,0.0924044051\C,-4.8298353599,-1.2127554679,0.0127
429586\C,-4.8298094493,1.2124419633,-0.0124640829\C,-1.2826249148,2.22
36394379,-0.1976956649\C,-1.2825838325,-2.2239614169,0.1978956746\C,-5
.4954218031,-2.2382110411,0.0080067689\C,-5.4953079426,2.237954454,-0.
0077291516\C,-0.5425579246,-3.4279508089,0.3351734158\C,-6.2330901148,
-3.453998025,-0.0141951871\C,-6.2326289217,3.453954012,0.0143661017\C,
-0.542746571,3.4277188227,-0.3349760345\C,-1.2229632334,4.653697359,-0
.4975846996\C,-0.523962767,5.8494667458,-0.6347378529\C,0.8778001972,5
.8492165405,-0.6132514525\C,1.5733859196,4.6529542237,-0.4603828102\C,
0.8865107873,3.4274507872,-0.3204311026\C,-7.5991721209,3.498977698,-0
.391493123\C,-8.2853633178,4.7272246104,-0.3521625857\C,-7.6500423627,
5.8909515321,0.0774798332\C,-6.3090303951,5.8484313643,0.4790913884\C,
-5.610269265,4.644536654,0.4468721832\C,-5.6110930675,-4.6447054138,-0
.4468786235\C,-6.310196708,-5.8483986078,-0.4791990162\C,-7.6511971226
,-5.8905870565,-0.0775137677\C,-8.2861636034,-4.7267311319,0.352303038
7\C,-7.5996224947,-3.4986831981,0.3917375649\C,0.8866988052,-3.4275237
85,0.3205284977\C,1.5737205693,-4.6529445065,0.4604865862\C,0.87827988
86,-5.8492779709,0.6134570058\C,-0.5234813237,-5.8496837506,0.63504247
92\C,-1.222624876,-4.6539985985,0.4978854596\C,-8.2697213988,-2.317833
8215,0.8377276768\C,-8.2696319692,2.3182651946,-0.8373034045\C,1.62346
87796,2.2217072089,-0.1811527202\C,1.623514868,-2.2217052674,0.1811462
596\C,2.2990967292,-1.2076390712,0.0811368834\C,2.2991298306,1.2076859
061,-0.0812244645\C,-8.8458387234,1.3190012639,-1.2187309635\C,-8.8456
209237,-1.3184513613,1.2193086484\C,3.0376693288,0.0000118331,-0.00006
9439\C,4.4377297978,-0.0000088729,-0.0001001948\C,5.1913398224,-1.2043
560448,0.0050815588\C,5.1913763258,1.2043149189,-0.0053167706\C,5.9132
4109,2.1912884563,0.0072048722\C,5.9131744962,-2.1913510607,-0.0074807
808\C,6.7971367947,3.3071500694,-0.0154806266\C,6.7970540738,-3.307226
8762,0.0151444781\C,6.4802146432,-4.5301980439,-0.6451417041\C,7.39412
08775,-5.5995890922,-0.5836200003\C,8.5977007483,-5.4761985827,0.10729
55506\C,8.9140987287,-4.2731883727,0.7507951333\C,8.0250652456,-3.2029
555351,0.7031798619\C,8.0251776888,3.2027990771,-0.7034514371\C,8.9142
295623,4.2730140311,-0.7511212013\C,8.5978206395,5.4760862163,-0.10774
24839\C,7.3942112549,5.5995563479,0.5831071924\C,6.4802858274,4.530184
555,0.6446822689\C,5.2629775183,-4.6802927046,-1.379195234\C,5.2630137
108,4.6803650705,1.3786600909\C,4.2405633811,-4.8288291994,-2.01889862
96\C,4.2405747447,4.8289840673,2.0183046358\H,-2.3135218201,4.64349315
83,-0.5219653212\H,-1.07150921,6.7857326877,-0.7617821201\H,1.42970788
84,6.7853792664,-0.7199578211\H,2.6634365108,4.6429865284,-0.442784387
4\H,-9.3298888358,4.7511491866,-0.6665461923\H,-8.201528719,6.83306485
57,0.100399634\H,-5.808502646,6.7569697817,0.8203950035\H,-4.568283376
7,4.5996472165,0.7673769138\H,-4.5691146249,-4.6000721766,-0.767442592
\H,-5.8099462559,-6.7570380541,-0.820640247\H,-8.2029513792,-6.8325415
819,-0.1005127978\H,-9.3306773673,-4.7503973578,0.6667451546\H,2.66376
89355,-4.642855367,0.4428133924\H,1.4302997431,-6.7853742121,0.7201669
059\H,-1.0709141603,-6.7860050341,0.7621683149\H,-2.3131823922,-4.6439
151664,0.5223468251\H,-9.327654664,0.4194289741,-1.5483585916\H,-9.327
1601532,-0.4187719717,1.5490486381\H,7.1418757795,-6.5306315784,-1.093
479169\H,9.2918394103,-6.3183239919,0.1423964284\H,9.8568092429,-4.170
5719528,1.2922440644\H,8.2622471207,-2.2630789551,1.2038189531\H,8.262
3679719,2.2628741489,-1.2039957734\H,9.8569632487,4.1703351668,-1.2925
179553\H,9.2919735747,6.3181980596,-0.1428866701\H,7.1419565918,6.5306
482684,1.0928713201\H,3.3337588756,-4.9369738527,-2.5810772302\H,3.333
8574791,4.9373075851,2.5805895175\\Version=ES64L-G16RevA.03\State=1-A\
HF=-2455.1090553\RMSD=4.583e-09\RMSF=1.607e-06\Dipole=0.6111994,0.0000
361,0.0000067\Quadrupole=15.4854728,28.1461328,-43.6316056,0.0009011,0

.0011382,3.4694276\PG=C01 [X(C64H28)]\\@

Calculated bond lengths of 1, 2, 3, and 14 – with ^{t}Bu and TIPS groups substituted for hydrogen atoms

Calculated bond lengths in Å of **1**, **2**, **3**, and **14** in the neutral (n), radical anion (ra), and dianion (da) oxidation state. The calculations were performed at the B3LYP/cc–pvdz level of theory using *Gaussian09* as described for the NICS calculations.

Compounds **1**, **2**, and **3** were optimized without including the *tert*–butyl groups in the structures, and the output files are listed in the previous paragraph. Compound **14** was optimized with the bulky TIPS groups using the same procedure as for **1–3**, and the output file is listed below.

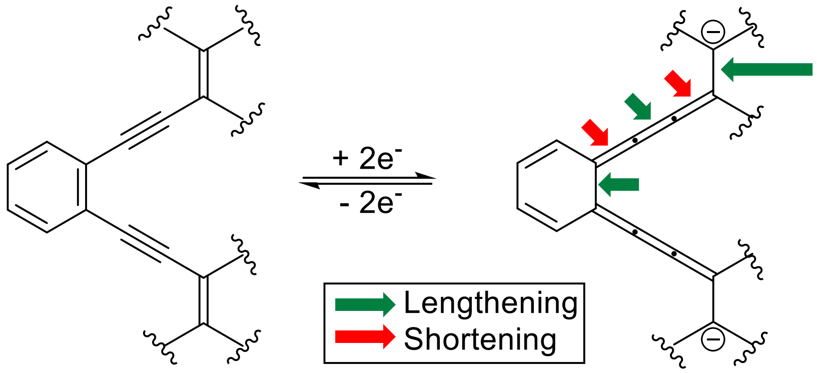

Supplementary Figure 84: The figure illustrates the change in bond length upon reducing **1**, **2**, **3**, and **14** from the neutral to the dianion. For more insight, see Supplementary Table 8 below.

Supplementary Table 8. Calculated bond lengths for neutral (n), radical anion (ra), and dianion (da) species and differences between these. The bonds are labeled in structures to the right.

**1**	n	ra	da	ra-n	da-ra	da-n
a	1.427	1.421	1.414	-0.005	-0.008	-0.013
b	1.220	1.224	1.229	0.004	0.005	0.009
c	1.427	1.421	1.414	-0.005	-0.008	-0.013
d	1.428	1.437	1.452	0.009	0.014	0.024
e	1.424	1.412	1.399	-0.012	-0.013	-0.025
f	1.222	1.229	1.238	0.008	0.008	0.016
g	1.420	1.408	1.398	-0.012	-0.010	-0.022
h	1.399	1.438	1.472	0.040	0.034	0.074

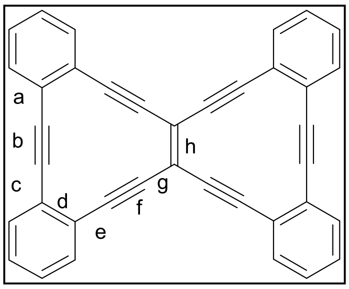

**2**	n	ra	da	ra-n	da-ra	da-n
a	1.360	1.356	1.350	-0.004	-0.006	-0.010
b	1.226	1.231	1.238	0.005	0.007	0.011
c	1.418	1.412	1.403	-0.006	-0.009	-0.016
d	1.434	1.444	1.458	0.010	0.014	0.024
e	1.423	1.410	1.397	-0.013	-0.013	-0.025
f	1.223	1.231	1.239	0.007	0.008	0.015
g	1.420	1.408	1.398	-0.013	-0.010	-0.023
h	1.402	1.441	1.476	0.040	0.034	0.074

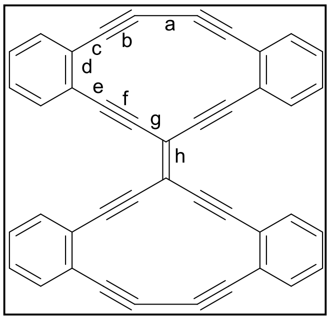

Supplementary Table 8 continues on next page

Supplementary Table 8 – *Continued.*

**14**	n	ra	da	ra-n	da-ra	da-n
a	1.226	1.227	1.229	0.001	0.002	0.003
b	1.428	1.428	1.425	0.000	-0.003	-0.003
c	1.426	1.430	1.437	0.004	0.007	0.011
d	1.424	1.417	1.407	-0.007	-0.010	-0.017
e	1.223	1.228	1.234	0.005	0.006	0.011
f	1.419	1.411	1.402	-0.008	-0.009	-0.017
g	1.400	1.427	1.453	0.026	0.027	0.053
h	1.419	1.409	1.401	-0.010	-0.008	-0.018
i	1.223	1.228	1.233	0.006	0.005	0.011
j	1.421	1.410	1.402	-0.010	-0.009	-0.019
k	1.429	1.438	1.446	0.009	0.009	0.017
l	1.420	1.410	1.402	-0.010	-0.008	-0.019
m	1.222	1.228	1.233	0.006	0.005	0.011
n	1.418	1.409	1.401	-0.010	-0.008	-0.018
o	1.400	1.427	1.453	0.027	0.026	0.053
p	1.423	1.417	1.409	-0.005	-0.009	-0.014
q	1.222	1.226	1.232	0.004	0.006	0.010
r	1.427	1.423	1.414	-0.004	-0.009	-0.013
s	1.425	1.428	1.434	0.003	0.006	0.009
t	1.429	1.429	1.428	0.000	-0.001	-0.002
u	1.227	1.227	1.228	0.000	0.001	0.001

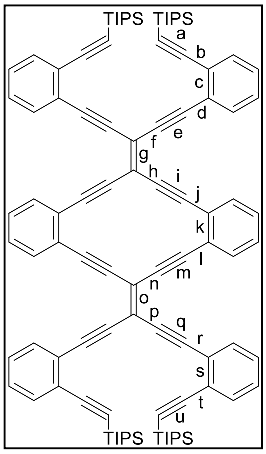

**3**	n	ra	da	ra-n	da-ra	da-n
a	1.359	1.357	1.353	-0.002	-0.004	-0.006
b	1.226	1.229	1.233	0.003	0.004	0.007
c	1.418	1.414	1.408	-0.004	-0.006	-0.010
d	1.434	1.440	1.449	0.006	0.009	0.015
e	1.423	1.415	1.405	-0.008	-0.010	-0.017
f	1.224	1.228	1.234	0.005	0.005	0.010
g	1.420	1.413	1.405	-0.008	-0.008	-0.016
h	1.402	1.428	1.451	0.025	0.024	0.049
i	1.418	1.408	1.401	-0.009	-0.007	-0.017
j	1.223	1.228	1.233	0.005	0.005	0.010
k	1.420	1.410	1.402	-0.010	-0.008	-0.018
l	1.429	1.437	1.445	0.008	0.008	0.016

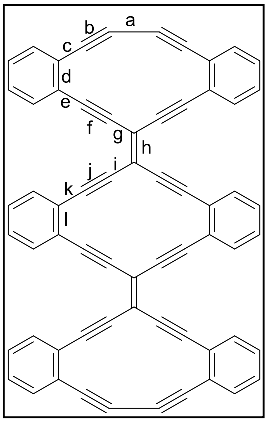

Output file

14 with TIPS groups, but without ^{t}Bu groups


```
1\1\GINC-NODE338\FOpt\RB3LYP\CC-pVDZ\C100H108Si4\ROOT\12-Nov-2017\0\\#
 opt=tight freq=noraman b3lyp/cc-pvdz geom=connectivity\\3 n0 anti\\0,
 1\C,-4.0775918332,0.3496136537,0.3801934887\C,-2.7146998348,0.19440443
 83,0.0982184701\C,-1.942571784,1.2614717283,-0.4291530917\C,-2.0436000
 676,-1.0279365257,0.3562795209\C,-4.8528533983,-0.7321784496,0.8832451
 937\C,-4.7154123956,1.6068885397,0.2222158456\C,-1.2445856865,2.151753
 3913,-0.8927003278\C,-1.4249259052,-2.0526521804,0.6044942461\C,-5.537
 972465,-1.640934639,1.3287329847\C,-5.2557761699,2.7002443961,0.127157
 8352\C,-0.7485901495,-3.2561380029,0.9379422048\C,-6.3387959184,-2.651
 1013287,1.9406436365\C,-5.7536431317,4.0317774495,0.0547013912\C,-0.46
 78745133,3.2114085203,-1.433075932\C,-1.0989228958,4.4026514818,-1.851
 0281621\C,-0.3601471525,5.4584934079,-2.3770875744\C,1.032633217,5.350
 2769932,-2.4965777707\C,1.6786490142,4.1843856806,-2.0950791577\C,0.95
 06250983,3.0979866597,-1.5635559007\C,-7.1358608714,4.3235277784,-0.13
 7841475\C,-7.5461785132,5.6722138168,-0.1798256496\C,-6.6294677262,6.7
 113320103,-0.041458017\C,-5.2706369828,6.4249267042,0.1425856322\C,-4.
 8411977731,5.1020374632,0.1891971919\C,-6.9141122068,-2.3934780439,3.2
 028992838\C,-7.6950336226,-3.3500974189,3.8465519889\C,-7.9219850257,-
 4.5902666202,3.2376302707\C,-7.3688232081,-4.8633020866,1.9890041228\C
 ,-6.5741084402,-3.9109486678,1.317566933\C,0.6696380543,-3.3709007565,
 0.8068115221\C,1.2940050115,-4.5803493728,1.1800032312\C,0.5486605325,
 -5.6523018518,1.6623982219\C,-0.843971589,-5.5423703624,1.7819610649\C
 ,-1.4834563465,-4.3586100528,1.4247443098\C,-6.0466858171,-4.230600520
 9,0.028240613\C,-8.1139349115,3.2959078903,-0.3001597463\C,1.634501431
 4,1.9145149377,-1.177904031\C,1.453255301,-2.2920481792,0.316984431\C,
 2.1578872134,-1.382416267,-0.0962535275\C,2.2609391984,0.9086361715,-0
 .8774749894\C,-9.0040414839,2.4671587199,-0.457614707\C,-5.6405926893,
 -4.5629783619,-1.0805395607\C,2.9414720239,-0.2945502127,-0.5602293655
 \C,4.3281396567,-0.4114186346,-0.7178374945\C,4.9812879716,-1.65283203
 1,-0.5026112517\C,5.1196598754,0.6940695384,-1.1355196536\C,5.82658276
 31,1.6233044634,-1.4971062846\C,5.5352930249,-2.7329865864,-0.35251262
 24\C,6.664084586,2.6594750662,-2.0072449773\C,6.053758604,-4.053023318
 9,-0.2265935411\C,7.3923564432,-4.314005787,0.1883804306\C,7.826538242
 ,-5.652762074,0.2803168517\C,6.9744659628,-6.7115016609,-0.0233616122\
 C,5.6585510234,-6.4553801136,-0.4293491877\C,5.2066072655,-5.142714090
 1,-0.5280543852\C,7.4175553644,2.414630349,-3.1749058013\C,8.237470823
 ,3.3984119617,-3.7216279196\C,8.3252494342,4.6538766334,-3.1078431459\
 C,7.5970071552,4.914171059,-1.949641701\C,6.7607483167,3.9338177271,-1
 .3760724236\C,8.3017960793,-3.2658022825,0.5250623739\C,6.058047144,4.
 2410860295,-0.1699716378\C,9.1310576813,-2.4190089445,0.8398728616\C,5
 .5046833353,4.5668389466,0.8753079238\H,-2.1832979138,4.4779730161,-1.
 7576363286\H,-0.8696683722,6.3701045777,-2.6961005016\H,1.6151953927,6
 .1781034641,-2.9056967987\H,2.7616740441,4.0909858244,-2.1841976533\H,
 -8.605904393,5.8868923952,-0.3252695769\H,-6.9741989913,7.7468589378,-
 0.0772441019\H,-4.5465997578,7.234729716,0.2541022708\H,-3.7869624971,
 4.8671590761,0.3436444392\H,-6.7297497517,-1.4249922307,3.670126591\H,
 -8.1270276608,-3.1283733686,4.8245357866\H,-8.5336602379,-5.3449557835
 ,3.7364247251\H,-7.5458824323,-5.8247476485,1.5045785061\H,2.378342299
 6,-4.6568708596,1.0877686541\H,1.05304914,-6.577967554,1.9472773779\H,
 -1.4316520704,-6.3826980682,2.1568181176\H,-2.5662908894,-4.2632438975
 ,1.5146417619\H,8.8525002301,-5.8438747708,0.5983098264\H,7.3363696028
 ,-7.7387196347,0.0559414541\H,4.985905658,-7.2805739529,-0.6724375712\
 H,4.1865866353,-4.9312674117,-0.8527956149\H,7.339160767,1.434524572,-
 3.6476729965\H,8.8080716927,3.186563345,-4.628149891\H,8.9660619923,5.
 4301514067,-3.5312375648\H,7.6670117956,5.8866925723,-1.4601142401\Si,
```

-5.0919036766,-5.1133182408,-2.7726750201\Si,-10.3837968175,1.24838852
83,-0.7237237926\Si,10.4181181608,-1.1730733216,1.3411557102\Si,4.7363
464822,5.1072691199,2.4830198047\C,6.1446172699,5.260369057,3.80091961
15\H,5.7389748285,5.9876812173,4.5328894391\C,3.4325717815,3.800764241
9,3.0419898389\H,3.3855174275,3.9336937451,4.1419064043\C,3.8958688579
,6.8269111666,2.2031209071\H,3.1149184456,6.8654726612,2.9888543561\C,
-10.9689447238,0.5909020872,0.998359633\H,-12.0263304185,0.3068263362,
0.8239261558\C,-11.8371754642,2.1753805901,-1.6012306402\H,-12.4042948
881,1.3623239674,-2.0975503048\C,-9.7300364297,-0.1971060451,-1.822749
1722\H,-10.4027549161,-1.0427150836,-1.5750583412\C,-4.1603587586,-6.7
979750051,-2.5868389008\H,-3.514947251,-6.8398608426,-3.4871587425\C,-
3.9313526216,-3.7769550025,-3.5371444587\H,-4.0370359259,-3.9343118405
,-4.629711884\C,-6.6685548892,-5.3475170888,-3.8686836928\H,-6.3490943
465,-6.0914782852,-4.6260765675\C,11.3401982733,-0.5721989881,-0.24833
29807\H,12.3254510448,-0.2328502148,0.1303363087\C,11.6523959091,-2.04
46352897,2.5485929731\H,12.0899873043,-1.2061402229,3.1266356857\C,9.5
421574764,0.3090513584,2.2135195298\H,10.2389829154,1.1566125269,2.055
5392566\C,-3.2478165736,-6.8381138127,-1.3472725481\H,-2.5434042379,-5
.9947396207,-1.3040475651\H,-3.8460705242,-6.8123850466,-0.421096107\H
,-2.6522123293,-7.7686432648,-1.3318093366\C,-5.0586637056,-8.04682995
89,-2.6098756021\H,-5.6799808437,-8.10888038,-3.5167648535\H,-4.443965
6229,-8.9638767448,-2.569945478\H,-5.7330064227,-8.0749609729,-1.73698
99713\C,-2.4392666131,-3.9371510461,-3.1960010373\H,-1.8402176669,-3.1
814191314,-3.7338377762\H,-2.251830551,-3.7848231644,-2.1202353081\H,-
2.042774965,-4.9268239267,-3.4724797096\C,-4.389654813,-2.3423072114,-
3.2152062938\H,-4.2968578606,-2.1330153263,-2.1373496819\H,-3.76511271
46,-1.606193365,-3.7513344799\H,-5.436779308,-2.1549893828,-3.49592424
49\C,-7.8574570673,-5.9428795018,-3.0915531396\H,-8.7012653023,-6.1537
749297,-3.7726812152\H,-7.6052317957,-6.8822242904,-2.5773674241\H,-8.
217603896,-5.2366098481,-2.3254132048\C,-7.1245391503,-4.0906048507,-4
.6307911027\H,-7.4651124793,-3.3003633716,-3.9402618191\H,-6.329905671
1,-3.6635911934,-5.261979545\H,-7.9766765919,-4.3300469622,-5.29178377
46\C,-12.8078831508,2.8965596667,-0.6497729631\H,-12.3082243261,3.7227
94185,-0.1158347708\H,-13.6456835982,3.3403945867,-1.2167694226\H,-13.
2432909087,2.2240222806,0.1055197781\C,-11.3578915502,3.1529416296,-2.
6905259757\H,-10.7825187708,3.9827848696,-2.2477807629\H,-10.713231277
1,2.6735084921,-3.4415303662\H,-12.2190208078,3.5933163226,-3.22437763
56\C,-9.8354957694,0.0421444985,-3.3390578405\H,-9.5061154274,-0.85481
33555,-3.8930857132\H,-10.8628444727,0.2700119909,-3.6641958846\H,-9.1
869358683,0.874516359,-3.660853939\C,-8.2923983743,-0.6056445454,-1.45
39438726\H,-7.9830447027,-1.5030231582,-2.0183189329\H,-7.5795946525,0
.200416364,-1.6924496887\H,-8.1723363865,-0.8361402369,-0.3858066499\C
,-10.9438158477,1.6769918362,2.0903739291\H,-11.509406783,2.5784724265
,1.811399408\H,-11.3766642796,1.2920562567,3.0311749342\H,-9.909523034
3,1.9928720731,2.3048025441\C,-10.234866776,-0.6644381694,1.5002962818
\H,-10.2766288176,-1.5001649566,0.7849645998\H,-9.1724240548,-0.456189
6665,1.708628635\H,-10.6838254027,-1.0191121451,2.4452320248\C,7.44714
20454,5.8438682984,3.223055799\H,8.1926312952,6.0000128848,4.023270826
\H,7.8911552777,5.1557519918,2.4848499042\H,7.2957117413,6.8105398099,
2.7202486051\C,6.4538624066,3.9637623877,4.5694288104\H,5.5666546662,3
.5386395297,5.0633057768\H,6.8693449341,3.1896703713,3.9022075746\H,7.
2086661351,4.1525547918,5.3536521355\C,4.8124914127,8.045046481,2.4108
085546\H,4.2371068111,8.9823654768,2.3065280876\H,5.2838955449,8.05953
75501,3.4058794468\H,5.6176326507,8.0777612068,1.6570016798\C,3.195486
5999,6.9289237326,0.8358259428\H,3.9319522238,6.8999728941,0.015452267
1\H,2.4803228651,6.1121695098,0.6604783636\H,2.6408671602,7.8808601433
,0.7520390926\C,3.8803806173,2.3553566728,2.7543521516\H,3.1528293782,
1.6349587992,3.1677873993\H,3.9464372553,2.1733733788,1.6697878116\H,4

.863530822,2.1176286356,3.187013613\C,2.0119812123,4.0253572093,2.4936
956096\H,1.9774343784,3.9035287037,1.3986170504\H,1.3157588261,3.28117
70541,2.9186779856\H,1.6135802985,5.0230607563,2.736932887\C,10.947527
7189,-2.988023902,3.5407095478\H,10.4923264017,-3.8425475263,3.0129351
188\H,11.6693227575,-3.3943447115,4.2716846709\H,10.1468443029,-2.4913
011368,4.1080883944\C,12.8168512484,-2.7859487821,1.8688462592\H,13.50
78065286,-3.1948182637,2.6277189461\H,12.4579164061,-3.6400098523,1.26
93941197\H,13.4085423363,-2.1360304576,1.20553363\C,11.5839948032,-1.7
065430023,-1.2611849167\H,12.1918425622,-1.3460007338,-2.1103162413\H,
12.1089915895,-2.5675217388,-0.8215432954\H,10.6297525792,-2.078142711
6,-1.6696510649\C,10.6859897495,0.6254152538,-0.9589761993\H,10.553424
1646,1.4949529108,-0.2970030091\H,11.3080164878,0.95375622,-1.81089286
03\H,9.6956963173,0.3620808328,-1.3665605805\C,8.1986173388,0.66379404
05,1.551260325\H,7.7746113306,1.5829102447,1.9924674331\H,8.2853045073
,0.8323146376,0.4683615562\H,7.4634963103,-0.1447510348,1.6943107917\C
,9.3519320885,0.1533507413,3.7325731816\H,8.6618131926,-0.6740414682,3
.969290033\H,10.297729892,-0.0329012513,4.2656611127\H,8.9105111812,1.
0714492348,4.1594333955\\Version=EM64L-G09RevE.01\State=1-A\HF=-5033.3
727615\RMSD=7.851e-09\RMSF=1.433e-07\Dipole=0.0174116,-0.0078932,0.017
4366\Quadrupole=-0.462176,23.1551038,-22.6929278,8.0413608,-12.1642774
,-8.5685244\PG=C01 [X(C100H108Si4)]\\@

HOMO–LUMO plots

	HOMO	LUMO
**1**	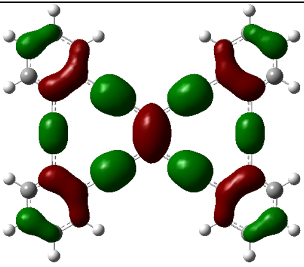	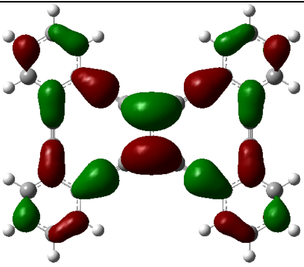
**2**	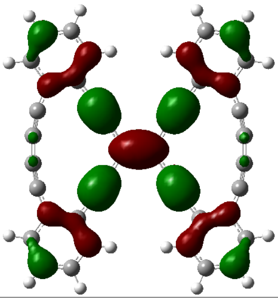	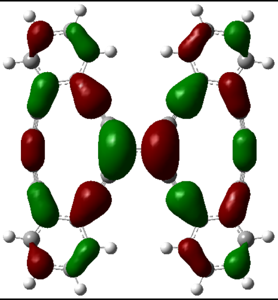
**3**	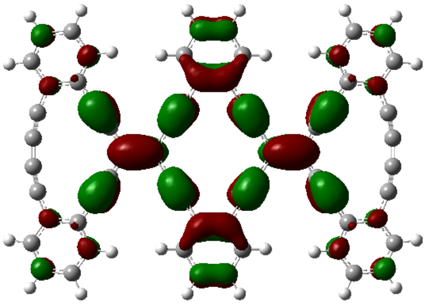	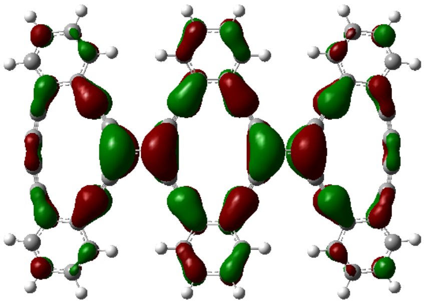
**14**	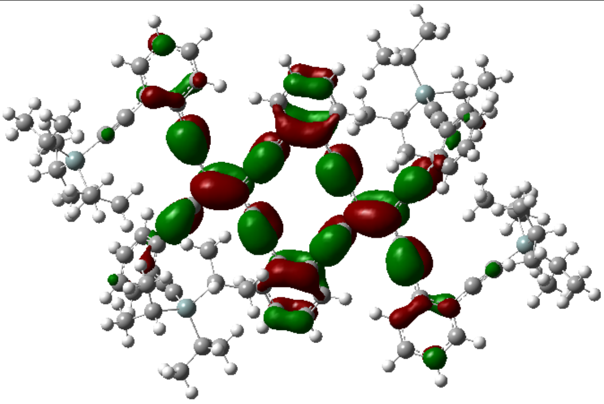	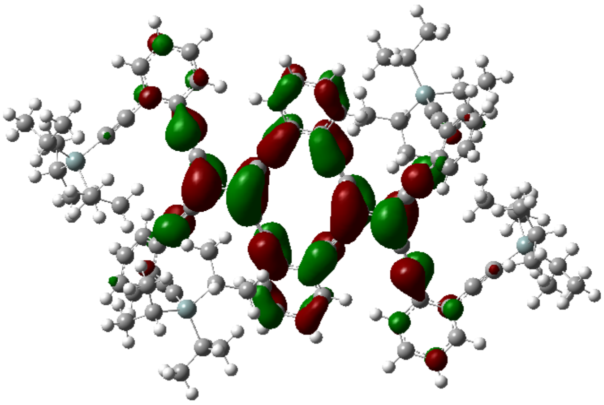

Supplementary Figure 85. Optimized geometries of the neutral compounds **1**–**3** and **14** calculated at the B3LYP/pvdz level of theory, and the visualisation of the frontier molecular orbitals of the neutral compounds calculated at the B3LYP/cc–pvdz//CAM–B3LYP/6–31g(d,p) level of theory. All performed using *Gaussian09*.

Supplementary References

1. Lv, J., Liu, Q., Tang, J., Perdih, F. & Kranjc, K. A facile synthesis of indolo[3,21-*jk*]carbazoles via palladium-catalyzed intramolecular cyclization. *Tetrahedron Lett.* **53**, 5248–5252 (2012).
2. Álvarez, R., Martinez, C., Madich, Y., Denis, J. G., Aurrecoechea J. M. & de Lera, A. R. A General Synthesis of Alkenyl-Substituted Benzofurans, Indoles, and Isoquinolones by Cascade Palladium-Catalyzed Heterocyclization/Oxidative Heck Coupling. *Chem. Eur. J.* **16**, 12746–12753 (2010).
3. Karpov, G. V. & Popik, V. V. Triggering of the Bergman Cyclization by Photochemical Ring Contraction. Facile Cycloaromatization of Benzannulated Cyclodeca-3,7-diene-1,5-diynes. *J. Am. Chem. Soc.* **129**, 3792–3793 (2007).
4. Bell, M. L., Chiechi, R. C., Johnson, C. A., Kimball, D. B., Matzger, A. J., Wan, W. B., Weakley, T. J. R & Haley, M. M. A versatile synthetic route to dehydrobenzoannulenes via in situ generation of reactive alkynes. *Tetrahedron* **57**, 3507−3520 (2001).
5. Gholami, M., Chaur, M. N., Wilde, M., Ferguson, M. J., McDonald, R., Echegoyen, L. & Tykwinski, R. R. Radiaannulenes: synthesis, electrochemistry, and solid-state structure. *Chem. Commun.* 3038−3040 (2009).
6. Philip, D., Gramlich, V., Seiler, P. & Diederich, F. π-Complexes incorporating tetrakis(phenylethynyl)ethane. *J. Chem. Soc., Perkin Trans. 2*, 875−886 (1995).
7. Anthony, J., Boldi, A. M., Rubin, Y., Hobi, R., Gramlich, V., Knobler, C. B., Seiler, P. & Diederich, F. Tetraethynylethenes: Fully cross-conjugated π-electron chromophores and molecular scaffolds for all-carbon networks and carbon-rich nanomaterials. *Helv. Chem. Acta* **78**, 13–45 (1995).
8. *Gaussian G09*, Revision E.01, Frisch, M. J., Trucks, G. W., Schlegel, H. B., Scuseria, G. E., Robb, M. A., Cheeseman, J. R., Scalmani, G., Barone, V., Mennucci, B., Petersson, G. A., Nakatsuji, H., Caricato, M., Li, X., Hratchian, H. P., Izmaylov, A. F., Bloino, J., Zheng, G., Sonnenberg, J. L., Hada, M., Ehara, M., Toyota, K., Fukuda, R., Hasegawa, J., Ishida, M., Nakajima, T., Honda, Y., Kitao, O., Nakai, H., Vreven, T., Montgomery, Jr., J. A., Peralta, J. E., Ogliaro, F., Bearpark, M., Heyd, J. J., Brothers, E., Kudin, K. N., Staroverov, V. N., Keith, T., Kobayashi, R., Normand, J., Raghavachari, K., Rendell, A., Burant, J. C:, Iyengar, S. S., Tomasi, J., Cossi, M., Rega, N., Millam, J. M., Klene, M., Knox, J. E., Cross, J. B., Bakken, V., Adamo, C., Jaramillo, J., Gomperts, R., Stratmann, R. E., Yazyev, O., Austin, A. J., Cammi, R., Pomelli, C., Ochterski, J. W., Martin, R. L., Morokuma, K., Zakrzewski, V. G., Voth, G. A., Salvador, P., Dannenberg, J. J., Dapprich, S., Daniels, A. D., Farkas, O., Foresman, J. B., Ortiz, J. V., Cioslowski, J. & Fox, D. J. *Gaussian, Inc.*, Version E.01, Wallingford CT (2000).
